# Single housing of juveniles accelerates early-stage growth but extends adult lifespan in African turquoise killifish

**DOI:** 10.18632/aging.206111

**Published:** 2024-09-16

**Authors:** Chika Takahashi, Emiko Okabe, Masanori Nono, Saya Kishimoto, Hideaki Matsui, Tohru Ishitani, Takuya Yamamoto, Masaharu Uno, Eisuke Nishida

**Affiliations:** 1 Laboratory for Molecular Biology of Aging, RIKEN Center for Biosystems Dynamics Research (BDR), Hyogo, Japan; 2 Department of Neuroscience of Disease, Brain Research Institute, Niigata University, Niigata, Japan; 3 Department of Homeostatic Regulation, Division of Cellular and Molecular Biology, Research Institute for Microbial Diseases, Osaka University, Osaka, Japan; 4 Center for iPS Cell Research and Application (CiRA), Kyoto University, Kyoto, Japan; 5 Institute for the Advanced Study of Human Biology (WPI-ASHBi), Kyoto University, Kyoto, Japan; 6 Medical-risk Avoidance based on iPS Cells Team, RIKEN Center for Advanced Intelligence Project (AIP), Kyoto, Japan

**Keywords:** lifespan, aging, growth, housing density, African turquoise killifish

## Abstract

Within the same species, individuals exhibiting faster growth tend to have shorter lifespans, even if their fast growth arises from early-life pharmacological interventions. However, in vertebrates, the impact of the early-life environment on the growth rate and lifespan has not been fully elucidated. In this study, by utilizing the short-lived African turquoise killifish, which is suitable for a comprehensive life-stage analysis in a brief timeframe, we explored the effects of housing density during the juvenile stage on holistic life traits. As a result, we found that lower housing densities resulted in faster growth, but led to longer adult lifespan, which was contrary to the common notion. Furthermore, the single-housed adult fish displayed a longer egg-laying period than did their group-housed counterparts. Our transcriptome analysis also demonstrated that, in terms of internal transcriptional programs, the life stage progression and aging process of single-housed fish were slower than those of group-housed fish. Collectively, our results suggest that sharing housing with others in early life might influence whole-life attributes, potentially leading to specific life history traits beyond the typical relationship between the growth rate and lifespan.

## INTRODUCTION

Fast postnatal growth is linked to short lifespans [1–4]. Correlation studies have shown this inverse relationship between the postnatal growth rate and lifespan among mammalian species [5] and within the same species in rats, mice, and dogs [2, 6, 7]. This relationship is often explained from various perspectives, including trade-offs in resource allocation among life history traits [8] and decreased accuracy in DNA synthesis due to the high rate of cell proliferation during growth [2]. In addition, the relationship has been observed not only in wild-type animals but also in mutant and transgenic animals. For example, growth hormone-deficient dwarf mice exhibit slower growth and a longer lifespan [9, 10]. These phenotypes can be partially rescued by early-life growth hormone replacement, indicating that early-life interventions can alter lifespan [11–13]. Even in these rescue experiments, the inverse relationship between the growth rate and lifespan does not change. To our knowledge, an exception to the general negative relationship between the growth rate and lifespan has not been previously reported in vertebrates.

Unlike those of early-life pharmacological interventions, the impacts of early-life environmental conditions, such as housing density, on the relationship between the growth rate and lifespan in vertebrates remain poorly understood. The median lifespan of African turquoise killifish (*Nothobranchius furzeri*) is 4-6 months, which facilitates a comprehensive life-stage analysis in a brief timeframe compared to the longer lifespans (more than two years) of canonical vertebrate model organisms such as mice [14–16]. Here, we explored the effects of housing density in the juvenile stage on whole-life traits, including growth, fecundity, and lifespan, in African turquoise killifish.

## RESULTS

### Juveniles housed at lower densities grow faster

**Figure 1:**
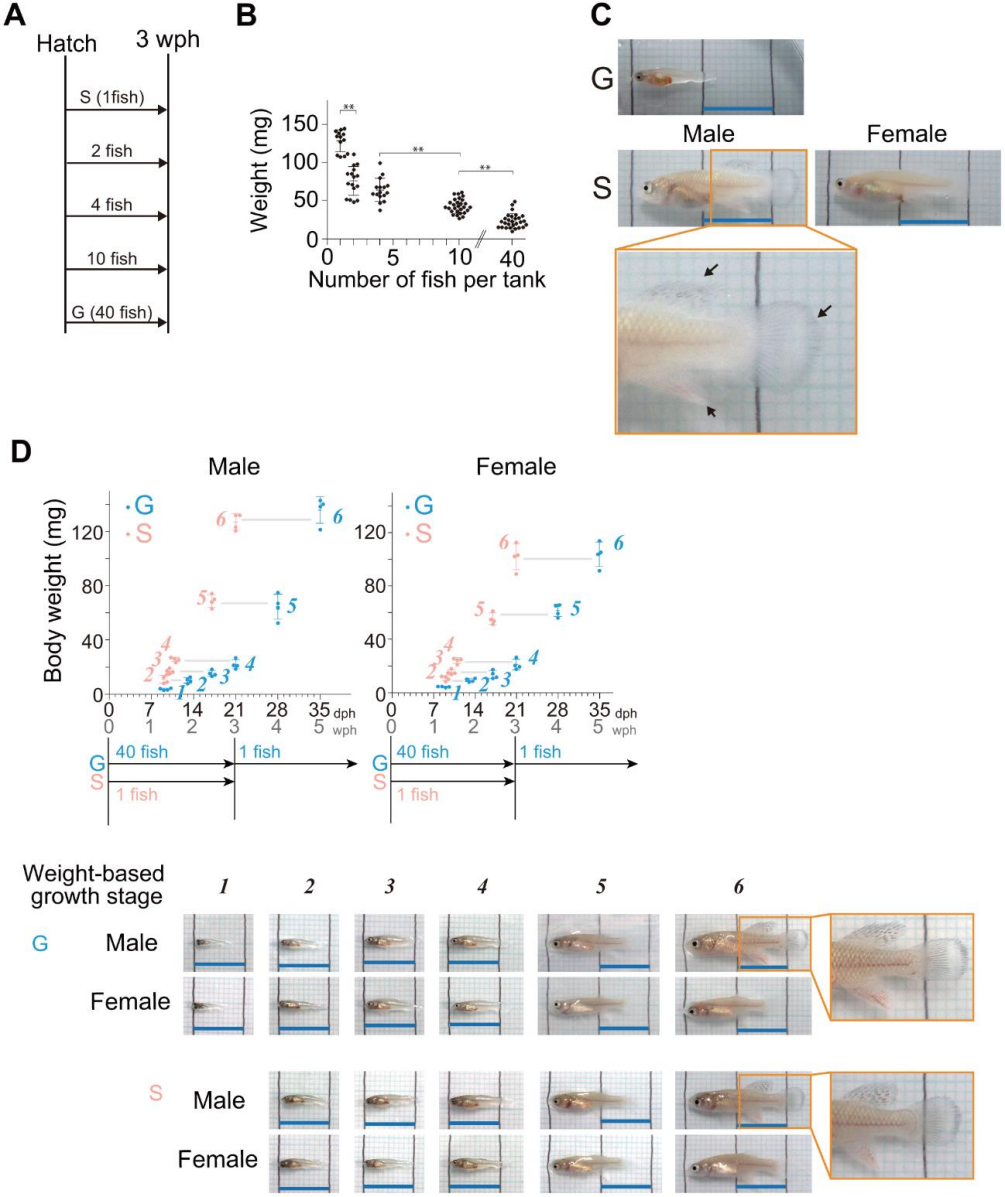
**Lower housing densities in the juvenile stage accelerate body growth.** (**A**) Schematic diagram showing the housing conditions. The hatched fish were kept at five different densities until three wph: 1, 2, 4, 10, and 40 fish per tank. (**B**) The graph shows their body weight at 3 wph under the housing conditions represented in (**A**). Each point represents an individual fish. The data were obtained for 13-28 fish at each density from a single experiment. The data are shown as the mean ± S.D. **, *p* < 0.01 by Tukey test. (**C**) Images are representative images of three-week-old fish under the group housing (40 fish per tank) and single housing (one fish per tank). The lower panels show higher magnification images of the boxed areas in the middle panels. The black arrows indicate fin coloration. The black lines in the images are drawn at 1 cm intervals. Blue scale bars, 1 cm. (**D**) Body weights at six different juvenile growth stages from 9 days posthatching (dph) to the onset of sexual maturity (group-housed fish; 35 dph, single-housed fish; 21 dph). The data were obtained from four samples at each stage. The data are shown as the mean ± S.D. Each point represents an individual fish. The blue and pink italic numbers represent the six weight-based stages of body growth in group- and single-housed fish, respectively (see methods section for detail). Representative images of fish at each weight-based growth stage under group housing (40 fish per tank) and single housing (one fish per tank). The black lines in the images are drawn at 1 cm intervals. Blue scale bars, 1 cm. Sex was confirmed by the expression level of a female-specific gene, *zona pellucida sperm-binding protein 3* (XM_015945764.2). (**A**–**D**) G: group-housed fish, S: single-housed fish.

In many studies using African turquoise killifish, newly hatched fish are initially kept in groups of 4-200 individuals per tank [16–21]. To investigate the effect of housing density on juvenile growth, we reared newly hatched fish at densities of 1, 2, 4, 10, and 40 fish per S-tank (15 × 9 × 10 cm, 0.65 L water) (Figure 1A), according to the protocol described by Dodzian et al. [18] with minor modifications. Because food availability affects growth, all the fish were fed in sufficient amounts until their abdomens turned orange, a sign of full satiation [18]. At three weeks posthatching (wph), we investigated body weight, and found that lower housing densities resulted in faster growth (Figure 1B). The average weight in single housing (one fish per tank) was 5.4 times greater than the average weight in group housing at the highest density (40 fish per tank). The average weight in single housing was also 1.7 times greater than the average weight in group housing at the second-lowest density (two fish per tank). In addition at three wph, single-housed juveniles reached the onset of sexual maturity stage, where male fin coloration was observed (Figure 1C, black arrows in pictures). On the other hand, group-housed fish at the highest density (40 fish per tank) had just reached the middle stage of juvenile growth (Figure 1C). These results suggest that in the juvenile stage, lower housing densities lead to faster growth.

We focused on the two conditions with the largest difference in growth: group housing (40 fish per tank) and single-housing (one fish per tank). In general, group-housed fish are separated individually before reaching the onset of sexual maturity stage because males become more aggressive upon their maturation [18]. Therefore, we reared all the fish at a density of one fish per L-tank (25 × 9 × 15 cm, 2 L water) from three wph onward to death (Supplementary Figure 1). These two housing conditions (40 fish per tank and 1 fish per tank) are hereafter denoted simply as “group housing” and “single housing”.

**Figure 2:**
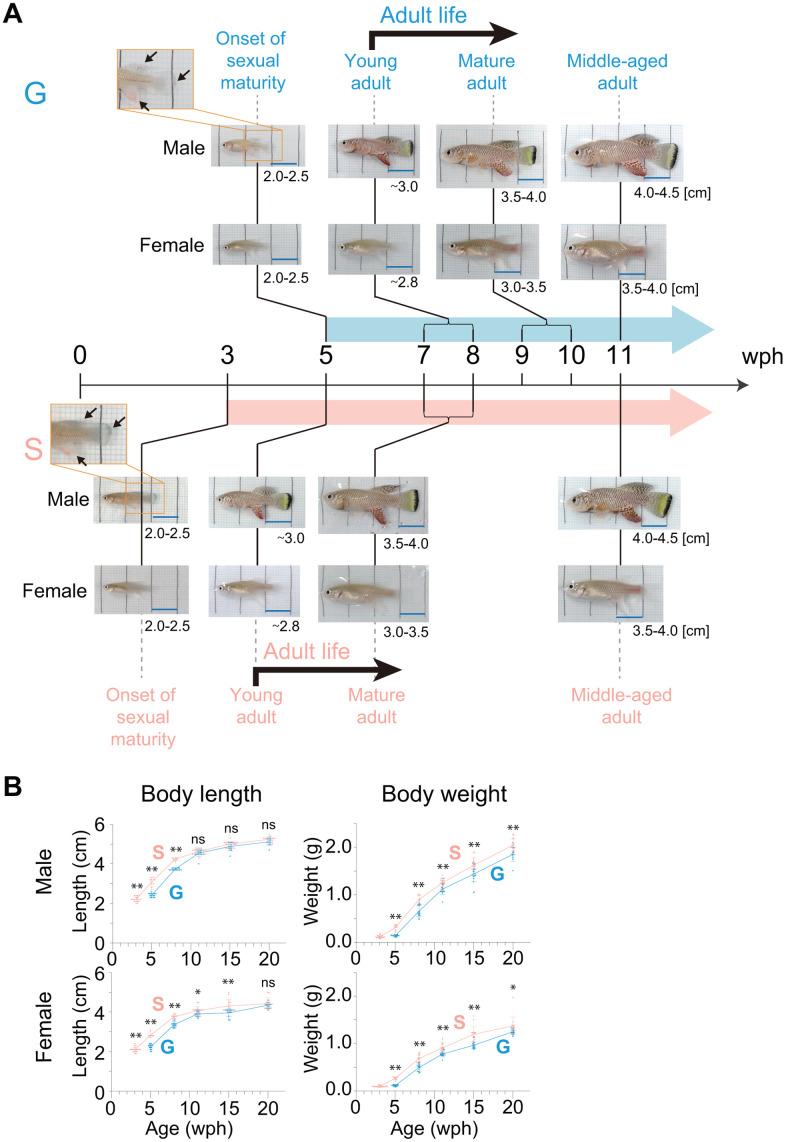
**Body growth from the onset of sexual maturity to the old stage.** (**A**) Representative images of male and female fish at the following four different life stages are shown: onset of sexual maturity, young adult, mature adult, and middle-aged adult. The numbers in the lower right corner of the images indicate the body length of the fish observed at each life stage. The arrows indicate fin coloration. The black lines in the images are drawn at 1 cm intervals. Blue scale bars, 1 cm. (**B**) Body length and body weight at each age after the onset of sexual maturity to the old stage (20 wph). The blue points indicate group-housed fish; the pink points indicate single-housed fish. Each point represents an individual fish. The data were obtained for 9-20 fish at each age from pooled data from one (group housing) and four (single housing) independent experiments. The data are shown as the mean ± S.D. *, *p* < 0.05; **, *p* < 0.01; ns, not significant by the Tukey test. (**A**, **B**) G: group-housed fish, S: single-housed fish.

After three wph, the group-housed fish reached the onset of sexual maturity stage at five wph, two weeks later than did the single-housed fish (Figures 1D, 2A). The appearance of the group-housed fish at this onset of sexual maturity stage was very similar to that of the single-housed fish at the same stage, with no obvious defects (Figure 2A). To compare the growth rate of juveniles in more detail, we examined the temporary changes in body weight from the early juvenile growth stage to the onset of sexual maturity stage in group- and single-housed fish, and then defined weight-based juvenile growth stages *1–6* (Figure 1D, see method section). Sex was confirmed on the basis of the expression level of a female-specific gene, *zona pellucida sperm-binding protein 3* (XM_015945764.2). Regardless of sex, it took 22 days for group-housed fish and 12 days for single-housed fish to grow from stage *2* to stage *6*, indicating that the growth rate of single-housed juveniles in stages *2–6* was 1.8 times faster than that of group-housed juveniles. All the fish were kept individually after stage *4* in the middle stage. However, regardless of sex, it took 14 days for group-housed fish and 10 days for single-housed fish to grow from stage *4* to stage *6*, indicating that the growth rate of single-housed juveniles even in the stages *4–6* was 1.4 times faster than that of group-housed juveniles. These results suggest that single-housed juveniles consistently grow faster than group-housed juveniles until the onset of sexual maturity stage.

Next, to examine whether group or single housing in the juvenile stage affected the body growth during sexual maturation, we observed the temporal growth from the onset of sexual maturity stage (group-housed fish, five wph; single-housed fish, three wph) in group- and single-housed fish (Figure 2A). As sexual maturation progressed, the body coloration of males spread from the fin to the entire body, and the abdomen of females became rounded, in both group- and single-housed fish (Figure 2A). The young adult fish are fully sexually mature and capable of breeding. The group- and single-housed fish reached the young adult stage at seven and five wph, respectively (Figure 2A). The timing of reaching this stage was within the range of the previously reported timings in captivity (4-8 wph) [18, 20–26]. The comparison of group- and single-housed fish at the same life stage from the onset of sexual maturity to middle age indicated that whether group housing or single housing in the juvenile stage had no effect on the change in appearance of fish during sexual maturations (Figure 2A).

Because both the group- and single-housed fish continued to grow until middle-aged adult regardless of sex (Figure 2A), we next examined the temporal changes in body length and weight from the onset of sexual maturity stage until the aged stage (20 wph) (Figure 2B). Regardless of the housing conditions, the rate of increase in body length after sexual maturity slowed, and the differences in body length between group- and single-housed fish of the same age were not significant for both males and females in old adults (20 wph). However, regardless of the housing conditions, the rate of increase in body weight after sexual maturity did not slow, and the differences in body weight between group- and single-housed fish of the same age persisted into old adults. Collectively, these results showed that in old adults, the group- and single-housed fish had similar body lengths but different body weights. This suggests the possibility of differences in body composition, such as in body fat percentage, muscle mass, and bone mass, between group- and single-housed fish in old age. Because the balance of fat mass, muscle mass, and bone mass is associated with health status in old age [27], the health status might differ between old group- and single-housed fish.

### Juveniles housed at lower densities have longer adult lifespan

**Figure 3:**
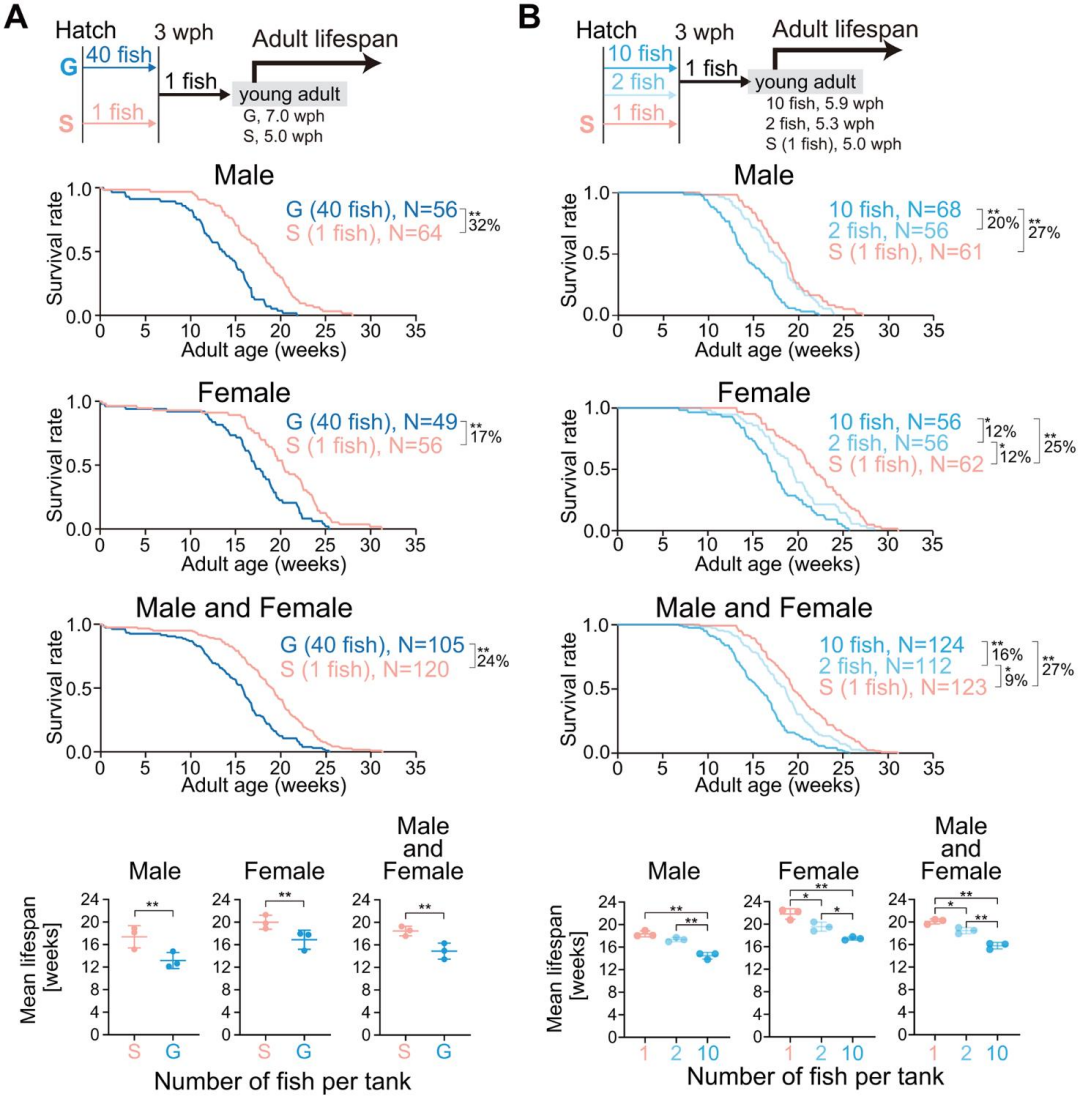
**Lower housing densities in the juvenile stage extend adult lifespan.** (**A**, **B**) The schemes for measuring the adult lifespan from reaching the young adult stage are shown at the top. The hatched fish were reared at a density of 1 or 40 fish per tank (**A**) or 1, 2, or 10 fish per tank (**B**) until 3 wph. After 3 wph, all the fish were reared individually. The age at which the fish reached the young adult stage was defined as zero weeks of adult age. The Kaplan–Meier survival curves (three independent experiments were pooled) of adult males (upper panel), females (middle panel), and both males and females (lower panel) are shown. The percentage indicates the rate of increase in the average lifespan. *, *p* < 0.05; **, *p* < 0.01 by log-rank test. The three lowest graphs show the mean lifespan. Each point represents the mean lifespan in each independent experiment. The data are shown as the mean ± S.D. *, *p* < 0.05; **, *p* < 0.01; ns, not significant by the log-rank test. G: group-housed fish, S: single-housed fish, N: number of fish analyzed.

Single housing in the juvenile stage accelerated the rate of body growth (Figure 1). In general, the growth rate is inversely correlated with lifespan [2]. Thus, we expected that the single-housed fish (fast-growing fish) would live for shorter periods than the group-housed fish (slow-growing fish). Unexpectedly, the mean adult lifespan in single-housed fish was longer than that in group-housed fish in both sexes (Figure 3A, male, 32%; female, 17%; male and female, 24%; Supplementary Figure 2A). Because juvenile growth was dependent on juvenile housing density (Figure 1A, 1B), we suspected whether the adult lifespan also exhibited a dependence on juvenile housing density. To test this hypothesis, we reared newly hatched fish at densities of 1, 2, and 10 fish per S-tank, and then, reared all the fish at a density of one fish per L-tank from three wph onward, and measured the adult lifespan (Figure 3B, upper schematic diagram). Both fish housed at a density of one or two fish per tank exhibited a longer mean adult lifespan than fish housed at a density of 10 fish per tank in both sexes (Figure 3B and Supplementary Figure 2B). These results suggest that lower housing densities in the juvenile stage extend adult lifespan despite fast body growth, which is contrary to the commonly held idea that growth rate and lifespan are inversely correlated.

### Single-housed fish have a longer egg-laying period compared to group-housed fish

**Figure 4:**
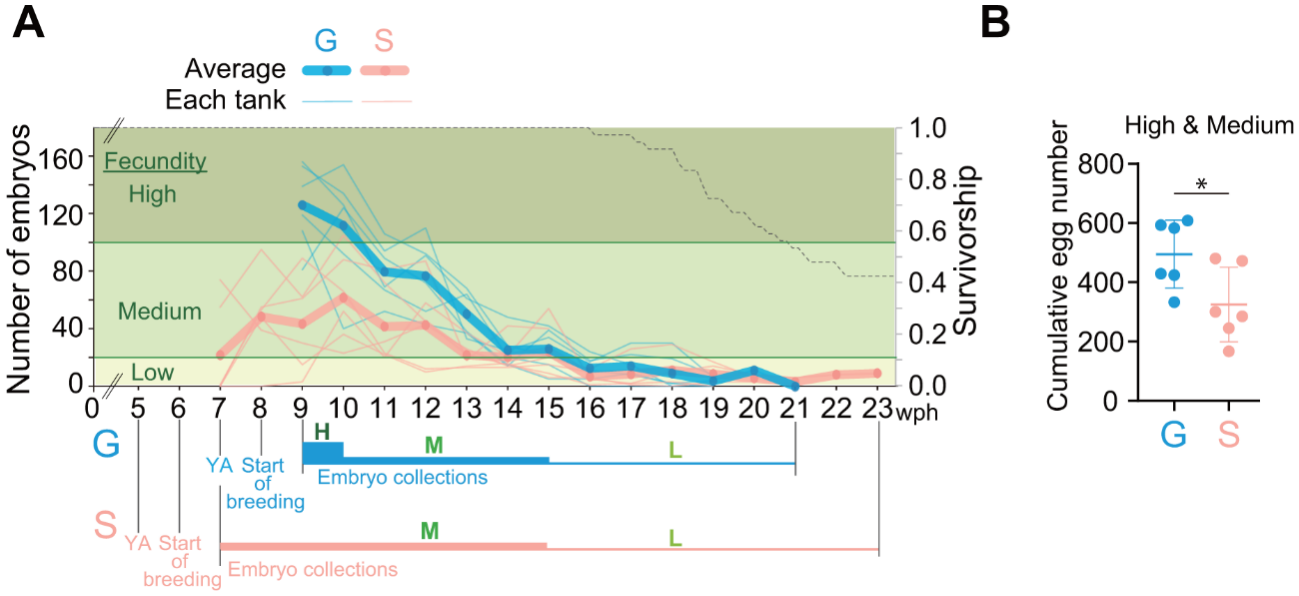
**Single-housed fish have a longer egg-laying period than group-housed fish.** (**A**) Temporal changes in the number of eggs laid by group- and single-housed fish. Six breeding tanks were prepared one week after the fish reached the young adult stage, and the number of embryos was counted once a week. The left vertical axis represents the number of viable embryos. The thick lines represent the average number of embryos in the six experimental tanks; the thin lines represent the number of embryos in each tank. The thick, blue or pink horizontal lines labeled “H” represent a high fecundity period; the medium lines marked “M” represent a medium fecundity period; and the thin lines labeled “L” represent a low fecundity period. The right vertical axis and black dashed line represent the survival rate and the Kaplan–Meier survival curves of all the fish used in this experiment, respectively. (**B**) Cumulative number of live embryos obtained at high and medium fecundity levels. *, *p* < 0.05 by t-test. Each point represents an individual tank. (**A**, **B**) G: group-housed fish, S: single-housed fish.

Adult lifespan of single-housed fish was longer than that of group-housed fish (Figure 3A). Therefore, we suspected that the egg-laying period of single-housed fish was also longer than that of group-housed fish. To test this possibility, we conducted weekly monitoring of temporal changes in the number of eggs laid until the old adult stage (Figure 4A). In group-housed fish, the number of eggs laid was high for the first two weeks, medium for the subsequent five weeks and low thereafter, whereas, in single-housed fish, it was medium for the first nine weeks and low thereafter (Figure 4A). The cumulative number of live embryos in single-housed fish was less than that in group-housed fish (Figure 4B). These results suggest that the egg-laying period of single-housed fish is longer than that of group-housed fish, although the number of eggs laid is not very high.

### The rate of change in the gonadal gene expression profile during sexual maturity in single-housed fish is slower than that in group-housed fish of the same life stage

**Figure 5:**
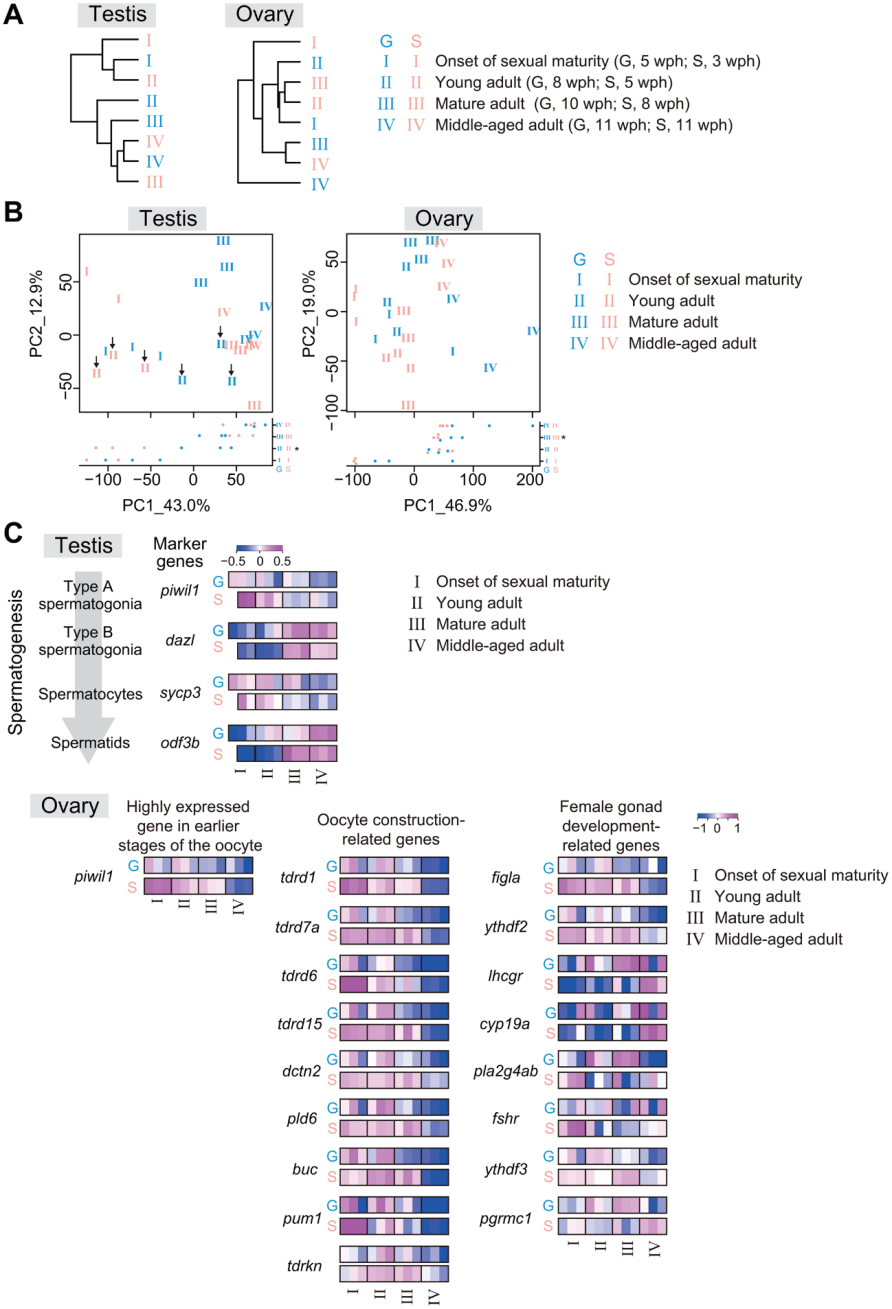
**The rate of gonadal transcriptional change with life-stage progression in single-housed fish is slower than that in group-housed fish.** RNA sequencing of testes and ovaries from group- and single-housed fish at the following four different life stages: onset of sexual maturity, young adult, mature adult, and middle-aged adult. The data were obtained from two or three samples at each life stage. (**A**) The dendrograms represent the hierarchical clustering of the samples based on the similarity of the changes in the average expression at each life stage for gene sets with TPM > 10 in at least one sample. (**B**) PCA plots prepared using VST values of the genes with TPM > 10 in at least one sample. Each number represents an individual sample at each growth stage. The black arrows in the left plot of the testis show the group- and single-housed fish at the young adult stage. *, *p* < 0.05 by t-test and Kruskal-Wallis test. (**C**) Changes in the individual expression of germ cell marker genes at each stage of spermatogenesis, early-oogenesis-related genes, oocyte-construction-related genes and female-gonad-development-related genes. *piwil1*, *piwi-like RNA-mediated gene silencing 1*; *dazl*, *deleted in azoospermia-like*; *sycp3*, *synaptonemal complex protein 3*; *odf3b*, *outer dense fiber of sperm tails 3B*; *tdrd1*, *tudor domain containing 1*; *tdrd7a*, *tudor domain containing 7a*; *tdrd6*, *tudor domain containing 6*; *tdrd15*, *tudor domain containing 15*; *dctn2, dynactin 2*; *pld6*, *phospholipase D family, member 6*; *buc*, *bucky ball*; *pum1*, *pumilio RNA-binding family member 1*; *tdrkn*, *tudor and KH domain containing*; *figla*, *folliculogenesis specific bHLH transcription factor*; *ythdf2*, *YTH N6-methyladenosine RNA binding protein F2*; *lhcgr*, *luteinizing hormone/choriogonadotropin receptor*; *cyp19a1a*, *cytochrome P450, family 19, subfamily A, polypeptide 1a*; *pla2g4ab*, *phospholipase A2, group IVAb*; *fshr*, *follicle stimulating hormone receptor*; *ythdf3*, *YTH N6-methyladenosine RNA binding protein F3*; *pgrmc1*, *progesterone receptor membrane component 1*. (**A**–**C**) G: group-housed fish, S: single-housed fish.

We suspected that the reduction in offspring number and increase in the egg-laying period in single-housed fish may be related to transcriptomic differences in the gonads. Therefore, we performed RNA sequencing analysis of testes or ovaries at four life stages: I, the onset of sexual maturity (G, 5 wph; S, 3 wph); II, young adult (G, 8 wph; S, 5 wph); III, mature adult (G, 10 wph; S, 8 wph); and IV, middle-aged adult (G, 11 wph; S, 11 wph) (Figure 2A). Interestingly, hierarchical clustering showed that single-housed fish tended to exhibit higher similarity to group-housed fish at earlier life stages than to group-housed fish at the same life stage in both the testes and ovaries (Figure 5A). For example, in the testes, single-housed fish at stage II exhibited the highest similarity to group-housed fish at stage I; in the ovaries, single-housed fish at stage II and III exhibited higher similarity to group-housed fish at stage I. These results suggest that the rate of gonadal transcriptional change with life stage progression in single-housed fish is slower than that in group-housed fish.

The samples were plotted along the PC1 axis in a manner reflecting the life stages regardless of housing type in both testes and ovaries in the PCA plots (Figure 5B). We next identified the DEGs between stage I and stage IV in group- and single-housed fish. The ribosome-related genes and the cilium-related genes were also highly enriched in 233 common DEGs with higher expression in the stage I than in the stage IV between group- and single-housed fish, and in 425 common DEGs with higher expression in the stage IV than in the stage I between group- and single-housed fish, respectively (Supplementary Figure 3). These ribosomal biogenesis and cilium organization are associated with the early and final spermatogenesis stages in zebrafish [28, 29], suggesting a link between life stage progression of testes and the progression of spermatogenesis. In the ovaries, the growth-related genes and the translation-related genes were highly enriched in 569 common DEGs with higher expression in the stage I than in the stage IV between group- and single-housed fish, and in 434 common DEGs with higher expression in the stage IV than in the stage I between group- and single-housed fish, respectively (Supplementary Figure 4). These processes are associated with the early oogenesis stage in zebrafish [30] and the aging of oocytes in mice [31], suggesting a link between the life-stage progression of ovaries and the progression of oogenesis and aging. Considering the differences in PC1 values between group- and single-housed fish at several stages (testes; II, ovaries; III, Figure 5B), these results imply that compared to group-housed fish, single-housed fish exhibited a slower progression of gametogenesis and gonadal maturation relative to the progression of life stage.

To verify this speculation, we focused on spermatogenic differentiation marker genes (*piwil1*, *dazl*, *sycp3*, and *odf3b*) [32–35], a gene highly expressed in earlier stages of oocyte development (*piwil1*) [36, 37], oocyte construction-related genes (*tdrd1*, *tdrd7a*, *tdrd6*, *tdrd15*, *dctn2*, *pld6*, *buc*, *pum1*, and *tdrkn*) and female gonad development-related genes (*figla*, *ythdf2*, *lhcgr*, *cyp19a*, *pla2g4ab*, *fshr*, *ythdf3*, and *pgrmc1*), and examined their changes in expression with life stage progression. In the testes and ovaries, the rate of change in the expression of these genes with life stage progression in single-housed fish tended to be lower than that in group-housed fish (Figure 5C). These results suggest that compared to group-housed fish at the same life stage, single-housed fish might have slower progression rates of gametogenesis and gonadal maturation, resulting in a lower proportion of mature sperm and oocytes in gonads. Collectively, from the perspective of the internal transcriptional programs, the results show that the progression of gonadal maturation and ovarian aging in single-housed fish might be slower than that in group-housed fish. This slow progression might explain the medium fecundity observed when we began to count the number of eggs laid and the extended egg-laying period observed in single-housed fish.

### Comparison of liver gene expression profiles between group-housed and single-housed fish in early and late adulthood

**Figure 6:**
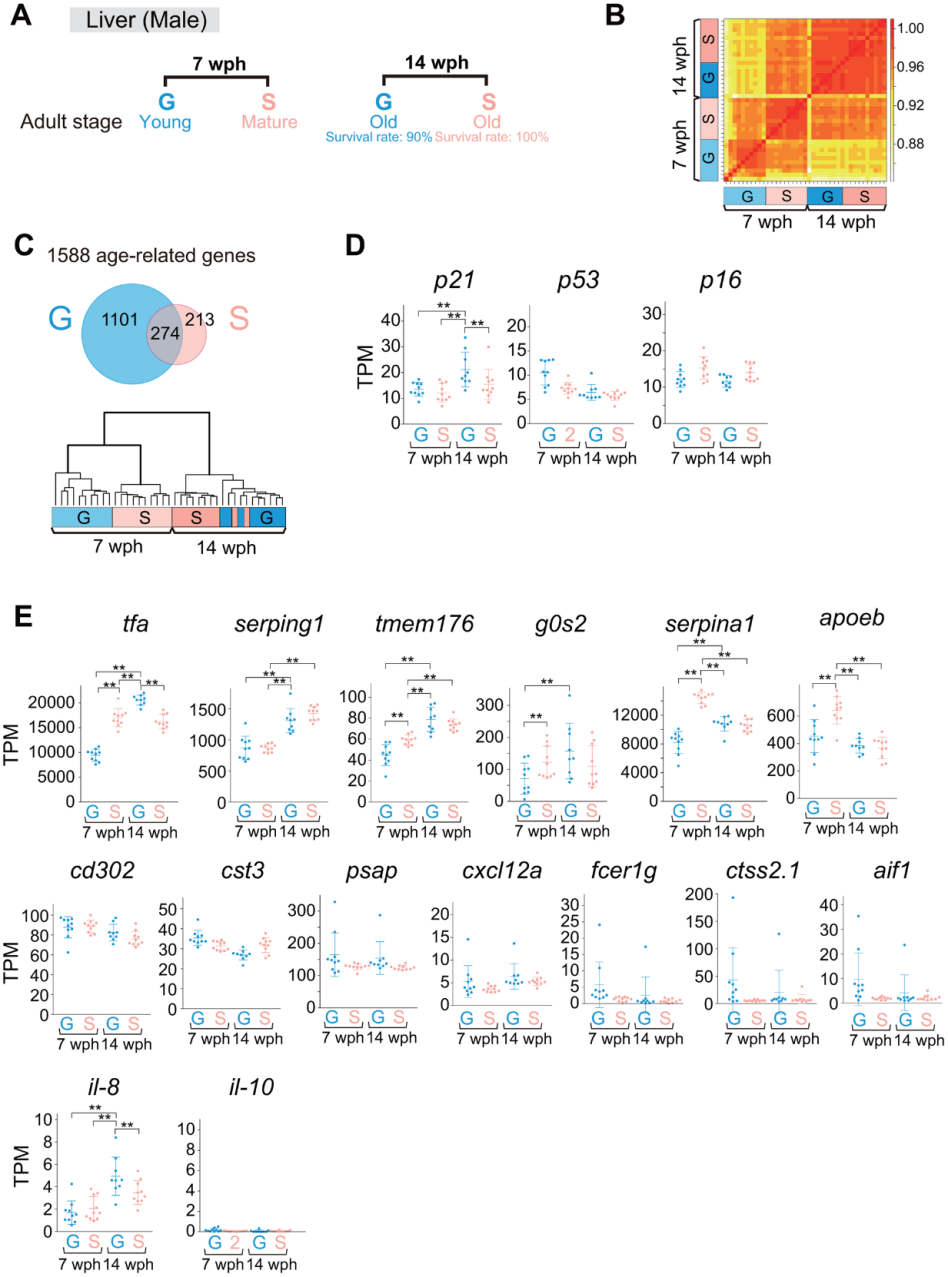
**Comparison of liver gene expression profiles between group- and single-housed fish in early and late adulthood.** (**A**) Schematic diagram showing the sampling for RNA sequencing of livers at 7 and 14 wph. At each age, 9-10 fish were sampled. (**B**) Correlation coefficients between samples in the gene sets with TPM > 10 in at least one sample. (**C**) The upper panel shows a Venn diagram of the DEGs (> 1.5-fold change, *padj < 0.01*) between 7 wph and 14 wph in either group- or single-housed fish (1588 genes). The lower panel shows hierarchical clustering of the samples based on the similarity of the changes in expression of the 1588 age-related genes. (**D**, **E**) TPM values of cell senescence markers (**D**) and SASP factors (**E**). Each circle represents an individual sample. **, *padj < 0.01* by DESeq2. *p21*, *cyclin dependent kinase inhibitor 1A*; *p53*, *tumor protein p53*; *p16*, *cyclin dependent kinase inhibitor 2A*; *tfa*, *transferrin-a*; *serping1*, *serpin family G member 1*; *tmem176*, *transmembrane protein 176*; *g0s2*, *G0/G1 switch 2*; *serpina1, serpin family A member 1*; *apoeb*, *apolipoprotein Eb*; *cd302*, *CD302 molecule*; *cst3*, *cystatin C*; *psap*, *prosaposin*; *cxcl12a, chemokine (C-X-C motif) ligand 12a*; *fcer1g, Fc epsilon receptor IgFc epsilon receptor Ig*; *ctss2.1*, *cathepsin S, ortholog2, tandem duplicate 1*; *aif1*, *allograft inflammatory factor 1-like*; *il-8, interleukin-8*; *il-10, interleukin-10*. (**A**–**E**) G: group-housed fish, S: single-housed fish.

As single-housed adult fish lived longer than group-housed fish, we suspected that the rate of the aging process in single-housed fish was slower than that in group-housed fish. Organismal metabolism changes with age [3], and the liver plays a central role in the organismal metabolic processes. Therefore, we compared the gene expression profiles of livers from group- and single-housed fish at two different ages: 7 wph (early adulthood) and 14 wph (late adulthood) (Figure 6A). Interestingly, the correlation coefficients between all samples showed that group- and single-housed fish at 14 wph were very similar to each other (Figure 6B), despite the 2-week difference in adult age between group- and single-housed fish (Figures 2A, 6A). We identified 1588 age-related differentially expressed genes (DEGs) between 7 wph and 14 wph in either group- or single-housed fish. The hierarchical clustering of all samples based on the similarity of changes in the expression of the 1588 age-related genes also indicated that the expression profiles of the group- and single-housed fish were similar at 14 wph (Figure 6C, lower).

GO enrichment analysis revealed that the most enriched GO term among the 274 common age-related genes between group- and single-housed fish was “alpha amino acid metabolic process” (Figure 6C, upper, and Supplementary Table 1), consistent with previous studies showing GO terms enriched among aging-related genes in mouse livers [38]. The most enriched GO terms among the 213 single-housed fish-specific age-related genes and the 1101 group-housed fish-specific age-related genes were “steroid metabolic process” and “ribosome biogenesis”, respectively (Figure 6C, upper, and Supplementary Table 1). This difference in enriched GO terms among age-related genes (Supplementary Table 1) could be attributed to the difference in life stage between group- and single-housed fish at 7 wph. The single-housed fish at 7 wph were in the mature adult stage during which large amounts of reproductive steroid hormones were likely produced; the group-housed fish were in the young adult stage, during which the fish were likely still actively translating for body growth. These results indicated that the differences in gene expression profiles between group- and single-housed fish at 7 wph decreased by 14 wph, suggesting the possibility that the rate of aging is slower in single-housed fish than in group-housed fish, and the group-housed fish caught up to single-housed fish in terms of the aging process at 14 wph.

Then, we analyzed marker genes for cellular senescence because the senescent cell burden increases with age, and found that the expression of the cell senescence marker *p21* (Figure 6D) and SASP factors *tfa* [39] and *il-8* [40–43] (Figure 6E) was upregulated in group-housed fish at 14 wph compared to other samples. This result suggests the possibility that group-housed fish at 14 wph had more senescent cells than single-housed fish at 14 wph, although the group-housed fish at 14 wph were two weeks younger in terms of adult age compared to the single-housed fish at 14 wph (Figures 2A, 6A). These results raise the possibility that the aging process in single-housed fish may be slower than that in group-housed fish.

### The rate of change in the gene expression profile of whole body during juvenile growth in single-housed fish is slightly slower than that in group-housed fish of the same growth stage

**Figure 7:**
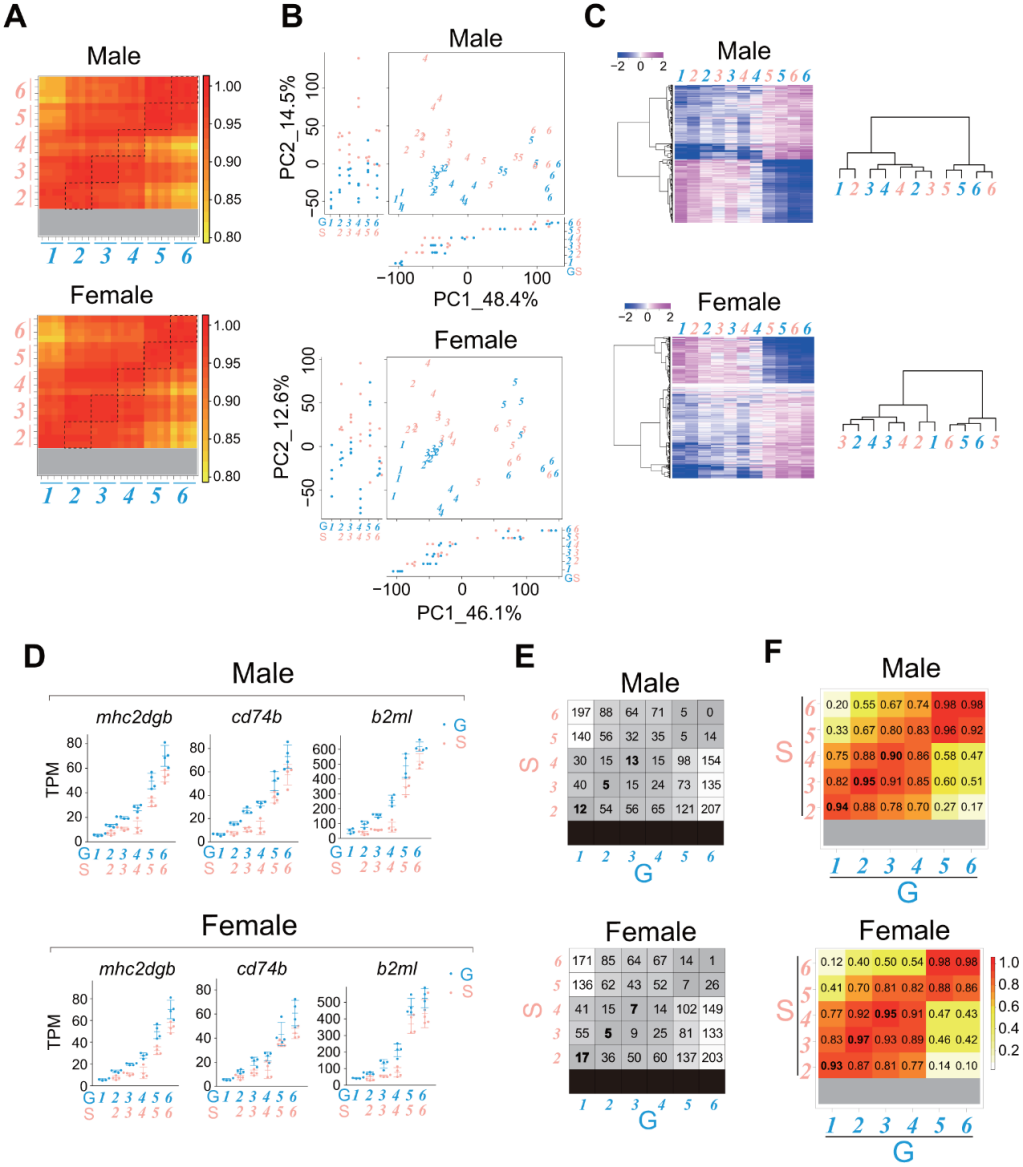
**The rate of whole-body transcriptional change with the progression of the juvenile growth stage in single-housed fish is slightly slower than that in group-housed fish.** RNA sequencing of the whole bodies of the juveniles (stages *1*-*6*) shown in Figure 1D. The data were obtained from four samples at each stage. (**A**) Heatmaps showing the correlation coefficients between all samples for genes with TPM > 10 in at least one sample. The dashed squares show the correlation coefficients between group- and single-housed fish at the same growth stage. (**B**) PCA plots using VST values of the genes with TPM > 10 in at least one sample. Each italicized number represents an individual sample at each growth stage. Each circle represents a value of PC1/2 in an individual sample. (**C**) Heatmaps showing changes in the average expression at each growth stage for the common DEGs (stage *1*/*2* vs. stage *6*) between group- and single-housed juveniles (See Venn diagrams in Supplementary Figures 5-6; males, 714 DEGs; females, 706 DEGs). The right dendrograms represent the hierarchical clustering of the samples based on the similarity of changes in expression (**D**) Examples of the expression of the immune response-related genes that were enriched among the common DEGs (stage *1*/*2* vs. stage *6*) between group- and single-housed juveniles (See Venn diagrams in Supplementary Figures 5-6). The TPM values are shown. Each circle represents an individual sample. *mhc2dgb*, *major histocompatibility complex class II DGB gene*; *cd74b*, *CD74 molecule*; *b2ml*, *beta-2-microglobulin, like*. (**E**) Number of DEGs with > 4-fold (*padj* < 0.01) differences in at least one of the two-group comparisons among the genes with TPM > 50 in at least one sample. (**F**) Heatmaps showing the correlation coefficients between samples in genes whose expression changed substantially with body growth. (**A**–**F**) G: group-housed fish, S: single-housed fish. The blue and pink italic numbers represent the six weight-based stages of body growth in group- and single-housed fish, respectively.

Analysis of the internal transcriptional programs in the gonads and liver suggested that single-housed fish have a slower rate of life-stage progression and aging process than group-housed fish. We wondered whether the rate of change in the gene expression profile of juveniles with growth in single-housed fish might also be slower than that in group-housed fish. To address this question, we conducted time-series sampling across growth stages *1–6* for both males and females (Figure 1D). Then, we compared transcriptomes between group- and single-housed juveniles at the same growth stages. The correlation coefficients between samples showed that the gene expression profiles were quite similar between group- and single-housed juveniles at the same growth stages in both sexes (Figure 7A).

In principal component analysis (PCA), with the PC1 axis explaining over 45% of the variance, the samples were plotted in a manner reflecting the growth stages regardless of housing type for both sexes (Figure 7B). For the common DEGs between stage *1*/*2* and stage *6* in group- and single-housed fish (Supplementary Figures 5, 6), the temporal changes in expression with body growth were roughly similar between the group- and single-housed fish of both sexes (Figure 7C, heatmaps). For example, cellular anatomical entity morphogenesis-related genes and regulation of RNA splicing-related genes, which were enriched in the common DEGs with higher expression in the stage *1*/*2* than in the stage *6* between group- and single-housed fish (Supplementary Figures 5, 6), also showed similar decreases in expression with body growth (Supplementary Figure 7A). It has been reported that during mouse embryonic development, the frequency of alternative splicing increases to allow the expression of specific gene isoforms at different developmental stages [44]. Based on our results, the frequency of alternative splicing may be high at the embryonic stage and decrease with growth after hatching regardless of housing conditions. In the GO terms enriched among the DEGs with higher expression in the single-housed fish than in the group-housed fish in stages *1-4*, during which housing conditions (group or single) differed, several terms were likely associated with the body growth rate in the juvenile stage (Supplementary Tables 2, 3). For example, the expression of muscle structure development/pyruvate metabolic process-related gene in stages *2-4* in single-housed fish tended to be higher than that in stages *1-4* in group-housed fish of both sexes (Supplementary Figure 7B). Higher expression of these genes in single-housed fish might lead to increased energy production, resulting in a higher growth rate. Collectively, these results suggest that although there are the DEGs between group- and single-housed fish during stages *1-4*, the gene expression profiles at each weight-based growth stage until the onset of sexual maturity were roughly similar between group- and single-housed juveniles. Therefore, both group- and single-housed juveniles would undergo roughly similar growth processes, despite having different growth rates.

However, the analysis of the similarity between samples using hierarchical clustering of the common DEGs (stage *1/2* vs. stage *6*) between group- and single-housed fish (Figure 7C, right dendrograms) showed that in both sexes, single-housed fish tended to exhibit higher similarity to group-housed fish at the earlier growth stage than to group-housed fish at the same growth stage. In zebrafish, the immune system develops both morphologically and functionally after hatching [45]. Our results showed that the expression of immune response-related genes increased more gradually in single-housed fish than in group-housed fish in both sexes (Figure 7D). In addition, the analysis of both the number of DEGs between samples (Figure 7E) and the correlation coefficient between samples for the DEGs (Figure 7F) indicated that single-housed fish tended to exhibit higher similarity to group-housed fish at the earlier life-stage than to group-housed fish at the same life-stage in both sexes. Taken together, these results suggest that from the perspective of internal transcriptional programs, single-housed juveniles have a slightly slower rate of growth stage progression than group-housed juveniles in both sexes, consistent with our hypothesis. Notably, at 17 and 21 dph, in the comparisons of group-housed and single-housed fish of the same age, the single-housed fish were more advanced in terms of both growth stage and progression of the gene expression profile compared to group-housed fish.

## DISCUSSION

Our studies suggest that lower housing densities result in faster growth and longer adult lifespans, contrary to the common notion that faster growth is linked to shorter lifespans. There are two conceivable speculations for this phenomenon. In general, juvenile growth and development are presumed to be enhanced by cell proliferation and expansion [46]. Thus, cell proliferation and cell expansion may be more accelerated in lower-density housed juveniles than in higher-density housed juveniles of the same age. High cell proliferation rates due to rapid growth increase DNA replication stress and DNA replication errors, the accumulation of which is thought to shorten lifespan [2]. However, lower housing densities lead to faster growth but longer lifespan. Lower housing densities may have benefits that outweigh the negative effects of increased DNA replication stress due to rapid growth. For example, higher housing densities may result in decreased oxygen, increased contamination, and increased chronic stress from interactions with other individuals than lower housing densities. Therefore, lower housing densities may be a healthier environment compared to higher housing densities, leading to fast growth and longer lifespan. If only the growth of juveniles housed at lower densities could be artificially restricted without changing the housing environment by some methods such as genetic engineering, it may become clear how much the low-density environments contribute to lifespan extension. The other conceivable explanation is based on evolutionary adaptations to thrive in their respective environments. Under more unfavorable environments for African turquoise killifish, each individual might need to enhance their survival advantages. Because unfavorable environments generally have lower population densities, our single-housing scenario might mimic unfavorable environments. Therefore, single housing of juveniles might enhance survival advantages in adults, resulting in fast growth and a long lifespan.

Although single-housed fish produced offspring for a longer period than group-housed fish, the total number of eggs laid in the single-housed fish was lower than that in the group-housed fish. In terms of increasing the number of offspring, the single-housing condition may not provide significant advantages compared to the group-housing condition. African turquoise killifish inhabit ephemeral ponds where water is present only during the short rainy season in southeast Africa [47–50], and appear to live at low population densities [51]. In this specific wild environment, African turquoise killifish might have been evolutionarily driven to grow faster, begin spawning earlier, and continue to live and spawn as long as possible until the pond dries out, even at the expense of the number of offspring.

We also show that lower housing densities during only the juvenile stage lead to a longer egg-laying period, a longer lifespan, and a slower life stage progression rate in terms of internal transcriptional programs. Based on our findings, it is speculated that the life traits and the progression rate of internal transcriptional programs over the whole lifespan are influenced, at least in part, by housing density during early life. Studies of developmental origins of health and disease (DOHaD) have shown that prenatal and early postnatal environmental conditions are associated with health and disease risk in later life stages through epigenetic modification [52, 53]. However, there has been a lack of research reporting the relationship between early-life environmental conditions and lifespan in vertebrates to date [4]. Our study broadens the horizons of the DOHaD research field. By our juvenile RNA sequencing analysis, we detected the DEGs between group- and single-housed fish in weight-based growth stages *1-4*, during which housing conditions (group or single) differed. These genes might be key to understanding the differences in growth rate, fecundity, and lifespan between group- and single-housed fish. Further analyses of DEGs between group- and single-housed juveniles and epigenetic modifications to elucidate the underlying molecular mechanisms will provide a deeper understanding of the impact of early-life environmental conditions on whole life.

In summary, juvenile single housing leads to faster growth, longer egg laying periods, and longer lifespan compared to group housing, which is contrary to the common notion regarding the relationship between growth and lifespan. The whole-life history traits might be influenced by the degree of early-life cohabitation with others. Our findings provide new insights for a better understanding of aging and the DOHaD theory and contribute to the further advancement of various research fields, including aging.

## MATERIALS AND METHODS

### Fish maintenance

All experiments were performed using the GRZ strain of African turquoise killifish, which was a gift from Dr. Adam Antebi. Fish care protocols were based on the protocol described by Dodzian et al. [18] with minor modifications. The fish were housed at 28° C with a 12/12 h light/dark cycle. For hatching, the embryos were put into a 50 ml tube containing 20 ml of 1 g/L humic acid solution (Sigma, #53680) and shaken vigorously for 1 min. The embryos and solution were transferred to a 90-mm plastic petri dish and kept under optical illumination for 2-3 hours without a lid. Healthy hatched fish were selected and transferred to an S-tank (15 × 9 × 10 cm) filled with 1 g/L humic acid solution to a depth of approximately 2 cm. When reared for mating, the hatched fish were kept at a density of 30-40 fish per S-tank. The solution was diluted to half its concentration with autoclaved fish breeding system water every day, maintaining a depth of 2 cm. After 7 dph, the fish were reared in an S-tank (15 × 9 × 10 cm, 0.65 L water) with a water recirculation system (IWAKI, Japan). The conductivity and pH of the water in the system were maintained at 6,000-7,500 μS/cm and 6.0-7.5, respectively. From and after 21 days posthatching (dph), all the fish were individually housed in an L-tank (25 × 9 × 15 cm, 2 L water) because the males showed more aggressive behaviors as they grew. The hatched fish were fed freshly hatched brine shrimp (Great Salt Lake Brine Shrimp EGGS-90, Kitamura & Co Ltd., Japan) twice a day. All juveniles were fed in sufficient amounts until their abdomens turned orange, a sign of full satiation [18], at all housing densities. When the colored males reached 3 cm in body length, they were considered young adults. After reaching the young adult stage, the fish were fed freshly hatched brine shrimp and frozen bloodworms (Kyorin, Japan) twice a day. For mating, one young adult male and two young adult females were kept in a breeding tank (23 × 16 × 20 cm, 5 L water). After 3 days, sand trays were placed in the breeding tanks as spawning sites. The embryos collected weekly were cultured in Yamamoto’s Ringer solution (128 mM NaCl, 2.7 mM KCl, 2.5 mM CaCl_2_, 0.02 mM NaHCO_4_) [54] supplemented with 0.01% methylene blue at 28° C. Every day, dead embryos were removed, and the medium was replaced. The developed embryos with black eyes were transferred to sterile dry peat moss (Antonio, Japan) and incubated at 28° C. Within one month after egg collection, most of the eggs were ready to hatch.

### Measurements of body length and weight

All measurements were performed before feeding in the morning. The fish were anesthetized with 0.1% 2-phenoxyethanol in the system water or on crushed ice for 15-60 seconds, and transferred to a 90 mm plastic petri dish containing cooled system water. The dish was placed on graph paper, and pictures were taken with a mirrorless interchangeable lens camera (EOS M100/200 EF-M15-45 IS STM lens kit) to measure body length. The fish were transferred to a paper towel to remove the water and weighed quickly. For Figure 1A, 1B, we prepared 13 tanks with one fish, eight tanks with two fish, four tanks with four fish, three tanks with ten fish, and one tank with 40 fish each. The data were obtained for 13-28 fish at each density from a single experiment. The images in Figure 1C are representative images obtained from two independent experiments. For Figure 1D, we prepared 100 tanks with one fish each and five tanks with 40 fish each. For group-housed fish up to 21 dph, one tank was randomly selected on each measurement day (9, 13, 17, and 21 dph), and all fish in that tank were weighed and euthanized after the measurement. In the group-housed fish at 28/35 dph and all single-housed fish at 9, 10, 11, 17 and 21 dph, 16-18 tanks were randomly selected on each measurement day, and all the fish in that tank were weighed and euthanized after the measurement. To confirm the sex by the expression level of the female-specific gene, *zona pellucida sperm-binding protein 3* (XM_015945764.2), total RNA was extracted from each fish, and cDNA was synthesized. Real-time quantitative RT-PCR analyses were subsequently performed with a QuantStudio 3 real-time PCR system (Applied Biosystems), PowerUp SYBR Green Master Mix (Applied Biosystems), and a primer set (5’-GTATGGGACCAGGAGGCAAC-3’ (forward) and 5’-TCCTCTGCAGGTAGAGGTCC-3’ (reverse)). In group-housed juveniles, we selected four juveniles which were close to the average weight on each sampling date. Juvenile stages *1-6* were defined based on the weight of these four juveniles. Sampling of single-housed juveniles was conducted at the age when they reached each of the stages *2* through *6* in average body weight in the preliminary experiment. We selected four juveniles which were close to the average weight on each sampling date in single-housed juveniles. The weights of these selected juveniles are shown in Figure 1D. These samples shown in Figure 1D were used for whole body RNA sequencing of the juveniles (Figure 7). The data were obtained from a single experiment. For Figure 2A, we randomly selected four fish at each life stage, and measured their body length, and then sampled their gonads for RNA sequencing (Figure 5). The images in Figure 2A are representative images obtained in a single experiment. In Figure 2B, after the measurements, we returned the fish to the tank, confirmed that they were swimming vigorously within a few minutes, and continued rearing them. The data were obtained from 9-20 fish at each age from one (group housing) and four (single housing) pooled independent experiments.

### Sample preparation and data processing for RNA sequencing

Sampling was always performed before feeding in the morning. The fish were euthanized with 0.1% 2-phenoxyethanol in the system water. The fish were dissected on ice under a stereomicroscope (Leica M80). For whole liver sampling, RNA later (Sigma, #R0901) was used. The liver data (Figure 6) were obtained from 9-10 fish at each age from a single experiment. The harvested whole juveniles, testes, ovaries, and whole livers were quickly washed with PBS and immediately frozen in liquid nitrogen. Total RNA was isolated with TRIzol reagent (Thermo Fisher Scientific) and subjected to DNase treatment with a TURBO DNA-free kit (Invitrogen #AM1907) according to the manufacturer’s instructions. The quality of the total RNA was assessed using an Agilent Bioanalyzer 2100 and Agilent RNA 6000 nano kit. For the analysis of ovaries and testes, both library preparation using the TruSeq stranded mRNA Library (Illumina, CA, USA) and sequencing using NovaSeq 6000 with 100 bp paired-end reads (R1, ~20 million; R2, ~20 million) were performed by Macrogen (Kyoto, Japan). For analysis of whole juvenile fish and livers, library preparation using the NEBNext Poly(A) mRNA Magnetic Isolation Module (E7490), NEBNext Ultra II Directional RNA Library Prep Kit (E7760), NEBNext Multiplex Oligos for Illumina (96 Unique Dual Index Primer Pairs) (E6440), Biomek i5 Automated Workstation (Beckman Coulter, Indianapolis, United States) and sequencing using the NovaSeq 6000 S4 Reagent Kit v1.5 (1 lane) with 150 bp paired-end reads (juvenile fish: R1, ~40 million; R2, ~40 million; liver: R1,~20 million; R2, ~20 million) were performed by WPI-ASHBi SignAC (Kyoto, Japan). The quality check of the raw sequencing data was performed using FastQC v0.11.8 [55]. The raw data were processed using Trim-Galore v0.6.4 [56]. The processed reads were mapped to the *N. furzeri* reference genome Nfu_20140520 using HISAT2 2.1.0 [57]. The mapped reads were counted using featurecounts v.1.5.3 [58]. Transcripts per million (TPM) values were calculated using R (ver. 4.0.2). The subsequent analysis used genes with TPM > 10 in at least one sample. Principal component analysis (PCA) was performed using the VST values and the R package prcomp. The value obtained by taking the base 2 logarithm of the ratio of TPM+1 to the mean of all samples for each gene was used to assess the similarity of changes in gene expression with growth/life stage progression. The enrichment analysis was performed with the *Danio rerio* (zebrafish) source in the gene ontology (GO) biological processes (BP) gene sets by using Metascape (https://metascape.org/). For the expression analysis in Figure 5C, we used African turquoise killifish homologs of zebrafish spermatogenic differentiation marker genes, an oogenesis-related gene, and GO-term-related genes (GO: 0007308, oocyte construction; GO:0008585, female gonad development). Differentially expressed genes (DEGs) were identified using DESeq2 1.28.1 [59]. The corresponding data are available in GEO under accession number GSE245483. We described all the scripts and text files in Supplementary Materials 1–3.

### Lifespan measurements

Every day, fish survival was checked at the time of feeding, dead fish were removed from the tanks, and the date of death was recorded. Lifespan analyses were performed using the Kaplan-Meier estimator (three independent experiments were pooled in Figure 3). The log-rank test was used to compare survival curves between different housing conditions.

### Counting the number of eggs laid

For this experiment, we prepared six breeding tanks. We applied the same breeding protocol to both group- and single-housed fish. One young adult male and two young adult females were kept in a 5 L breeding tank (23 × 16 × 20 cm). After 3 days, the sand trays used as spawning sites were placed in the breeding tank. We collected the eggs in the sand trays once a week. Embryo collection in each tank was terminated when one of the three fish died of old age. The mean number of live embryos collected from each of the six tanks per condition was evaluated at three levels: low (0-20 embryos), medium (20-100 embryos), and high (> 100 embryos). The data were obtained from a single experiment.

### Statistical analysis

In order to determine whether the means of two independent groups are significantly different, we used SPSS (IBM). We first assessed the data normality using the Shapiro-Wilkes test. If data is non-normality, Kruskal Wallis tests were performed; otherwise, the homogeneous variance was assessed using Levene-tests. If data is homogeneous variance, t-test was performed; otherwise, Welch test was performed. The values of significance probability in the results of their tests were shown in Supplementary Table 4. Tukey tests were performed using SPSS (IBM). The results were considered significant when *p* was < 0.05. The log-rank test was performed using Oasis 2 [60].

### Data availability

The corresponding data are available in GEO under accession number GSE245483.

## Supplementary Material

Supplementary Figures

Supplementary Table 1

Supplementary Tables 2-4

Supplementary Material 1

Supplementary Material 2

Supplementary Material 3
