# Supplementary material for: Single housing of juveniles accelerates early-stage growth but extends adult lifespan in African turquoise killifish
Source: Aging (Albany NY). 2024 Sep 16;16(18):12443–72. doi: 10.18632/aging.206111 (PMC11466477; doi:10.18632/aging.206111)
Supplement: Supplementary Figures [file aging-16-206111-s001.pdf]

SUPPLEMENTARY FIGURES

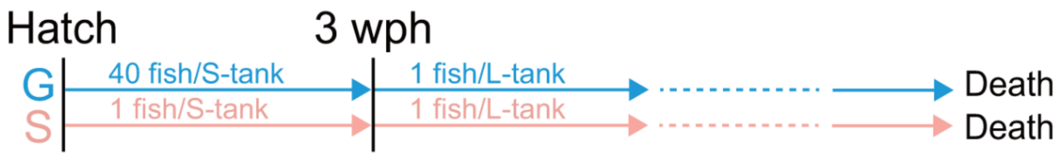

Supplementary Figure 1. The schematic diagram of group- and single-housing conditions. G: group-housed fish, S: single-housed fish. The hatched fish were kept at two different densities until three wph: 1 and 40 fish per S-tank. After three wph, all the fish were kept individually in L-tank.

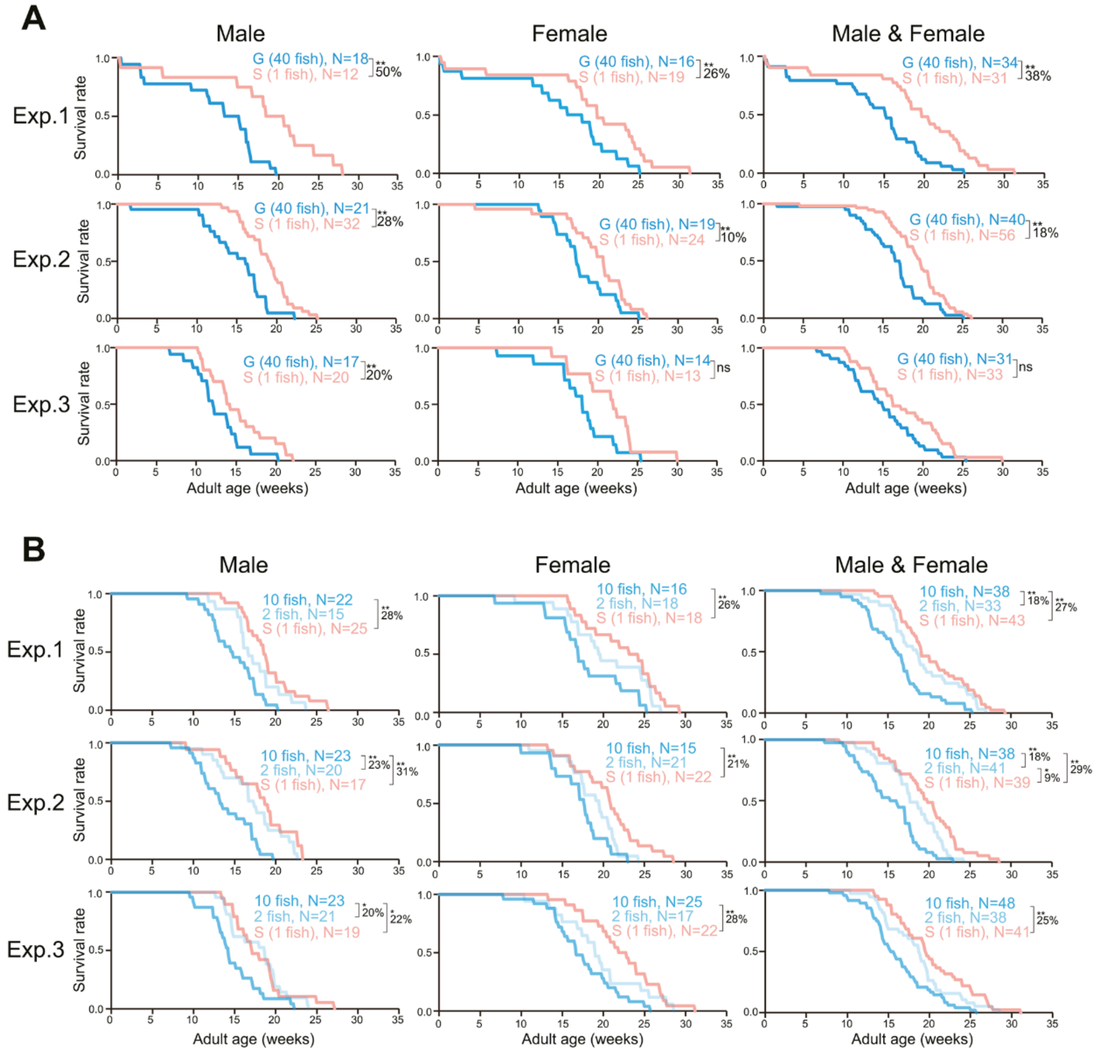

Supplementary Figure 2. Kaplan–Meier survival curves for each of the three independent lifespan measurements. The hatched fish were reared at a density of 1 or 40 fish per tank (**A**) or 1, 2, or 10 fish per tank (**B**) until 3 wph. After 3 wph, all the fish were reared individually. The age at which the fish reached the young adult stage was defined as zero weeks of adult age. The percentage indicates the rate of increase in the average lifespan. *, $p < 0.05$; **, $p < 0.01$ by log-rank test. G: group-housed fish, S: single-housed fish, N: the number of fish analyzed.

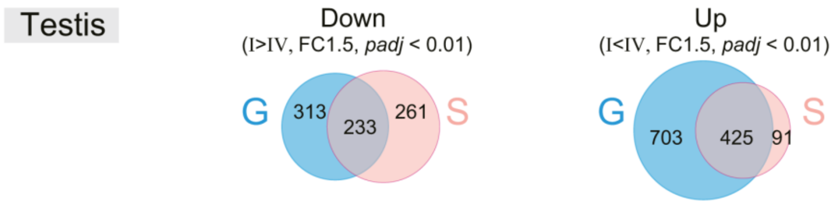

233 common down-regulated genes				
**Term**	**Description**	**LogP**	**Counts**	**Symbols**
GO:0042273	ribosomal large subunit biogenesis	-7.90	14	rplp0,npm1a,snu13b,ftsj3,rrp15,heatr3,rbm34,rsl1d1,ddx28,utp25,tsr1,dkc1,thumpd1,farsa
GO:0061061	muscle structure development	-5.56	16	dag1,mybphb,postnb,csrp1a,csrp2,pxna,fhl1a,itga6a,vcla,fermt2,pgm5,smarcd3b,col6a2,ogg1,jam2a,myom1b
GO:0031099	regeneration	-4.35	13	cdh2,serpinh1b,hmgb1a,tgif1,csrp1a,mycb,vdac3,dpysl2b,bmpr2b,kdm6bb,utp25,col4a5,rpl22l1
GO:0051146	striated muscle cell differentiation	-4.07	22	dag1,mybphb,csrp1a,csrp2,vcla,pgm5,smarcd3b,ogg1,jam2a,myom1b,col6a2,epb41a,cnn1b,cdh2,etv5b,col4a5,sema7a,ap4s1,islr2,dpysl2b,rplp0,ddx28
GO:0044770	cell cycle phase transition	-3.70	5	meis1b,ccnp,skp2,cdk21,cdk6
GO:0048729	tissue morphogenesis	-3.38	23	cdh2,sox9a,meis1b,fabp3,dag1,quo,apoa2,acta2,sdc3,fyna,csrp1a,pdgfra,fermt2,bmpr2b,ezrb,rpl22l1,fhl1a,cldn5a,vcla,smarcd3b,kdm6bb,seraf,ogg1
GO:0060216	definitive hemopoiesis	-3.34	5	lpla,meis1b,mycb,dkc1,rpl22l1
GO:0030198	extracellular matrix organization	-3.32	8	serpinh1b,col4a5,postnb,crtap,col4a1,col18a1a,col5a2a,col5a1
GO:0048568	embryonic organ development	-2.87	16	nog3,cdh2,sox9a,meis1b,fabp3,scarb2a,utp25,fstl1b,pdgfra,pacsin3,bmpr2b,cavin1b,kdm6bb,pbx1b,ogg1,mfap5
GO:0031102	neuron projection regeneration	-2.67	3	hmgb1a,csrp1a,dpysl2b
GO:0035118	embryonic pectoral fin morphogenesis	-2.62	4	cdh2,sox9a,hoxc8a,etv5b
GO:0051338	regulation of transferase activity	-2.57	14	adcyap1b,fyna,pdgfra,prrc1,dnmt3ab,ccnp,dbndd1,serpinh1b,scarb2a,fshr,tbc1d4,rangap1a,nfkbiaa,fxyd6l
GO:0006631	fatty acid metabolic process	-2.47	15	lpla,echdc2,acaa2,pck2,olah,acot17,zgc:158482,prtfdc1b,bhmt,nags,dnph1,gamt,fdxr,hibadhb,crtap
GO:0006694	steroid biosynthetic process	-2.36	9	star,hsd3b1,cyp11c1,star2,fdxr,igf2bp3,fshr,cdk21,zglp1
GO:0009116	nucleoside metabolic process	-2.28	4	prtfdc1b,tk1,dnph1,bhmt
GO:0031647	regulation of protein stability	-2.18	3	meis1b,utp25,bag2
GO:1901532	regulation of hematopoietic progenitor cell differentiation	-2.06	3	pdgfra,nfkbiaa,cdca7a
**425 common up-regulated genes**				
**Term**	**Description**	**LogP**	**Counts**	**Symbols**
GO:0007017	microtubule-based process	-11.68	47	gas8,ccdc65,tuba1b,poc1b,ssx2ipa,efhc1,pafah1b1b,dzip1,cfap206,usp33,spag6,gle1,lrrc23,spire2,odad1,kif6,armc2,daw1,ttll9,ak9,rpgrb,mapre3a,tekt4,clasp1a,ttll7,bbs1,kif26ba,kif21b,rfx3,lca5,cfap221,tbcelb,dnah3,kif16ba,ttc9c,cep89,clrn1,cep104,cep290,cfap126,wdpcp,iqub,arhgef9b,fxr1,agfg1a,si:ch211-260e23.9,smyd1b
GO:0007018	microtubule-based movement	-10.50	23	gas8,poc1b,ssx2ipa,pafah1b1b,cfap206,spag6,gle1,lrrc23,odad1,kif6,armc2,ak9,rpgrb,tekt4,bbs1,kif26ba,kif21b,rfx3,lca5,cfap221,dnah3,kif16ba,cep290
GO:0001539	cilium or flagellum-dependent cell motility	-7.83	21	gas8,ccdc65,efhc1,cfap206,spag6,armc2,daw1,tekt4,rfx3,dzip1,odad1,fxr1,agfg1a,buc,pafah1b1b,paqr5b,cfap221,spire2,spmap2,cd9a,dio2
GO:0032474	otolith morphogenesis	-5.33	22	gas8,ttc9c,lrrc23,daw1,cep290,wwc1,igfbp3,clrn1,ush1ga,whrna,poc1b,bcl6aa,dzank1,gle1,ubr3,rpgrb,ninl,dzip1,sec24d,sar1b,cep104,bbs1
GO:0070647	protein modification by small protein conjugation or removal	-4.60	31	arih1,klhl17,rnf41l,josd2,desi1a,otud3,zgc:66427,ube2r2,peli2,zgc:101783,ubr3,otud5a,sh3rf1,daw1,spsb4a,rnf19a,socs5b,kbtbd8,pias1b,ankib1a,asb15b,vcpip1,rnf25,rnf6,plaa,usp33,usp38,dis3l2,vps37c,ptpn23a,fzr1b
GO:0018107	peptidyl-threonine phosphorylation	-3.06	4	hipk2,hipk3a,hipk3b,hipk1a
GO:0002093	auditory receptor cell morphogenesis	-2.98	3	clrn1,ush1ga,whrna
GO:0003352	regulation of cilium movement	-2.79	3	ccdc65,cfap206,daw1
GO:0018200	peptidyl-glutamic acid modification	-2.79	3	ttll9,ttll7,ttll10
GO:0038127	ERBB signaling pathway	-2.70	5	cpne3,ptk2ba,socs5b,sh3rf1,fzr1b
GO:0042461	photoreceptor cell development	-2.57	8	dzank1,rpgrb,cep290,ninl,bbs1,lca5,poc1b,pafah1b1b
GO:0009896	positive regulation of catabolic process	-2.50	11	cth1,fxr1,upf1,sh3rf1,socs5b,dis3l2,fzr1b,pnpla2,tent5c,zc3h14,apoc1
GO:0070646	protein modification by small protein removal	-2.29	5	josd2,desi1a,otud3,otud5a,vcpip1
GO:0015849	organic acid transport	-2.03	9	slc6a19b,slc43a1a,pla2g10,slc1a1,fabp4b,slc1a3b,slc16a3a,slc38a2,slc16a4
GO:0048870	cell motility	-2.02	19	gas8,ccdc65,efhc1,cfap206,usp33,spag6,rnd1b,armc2,sh3rf1,itgb8,daw1,tekt4,lpxn,wwc1,apoc1,rfx3,ackr4b,zgc:174863,ccl35.2

Supplementary Figure 3. Venn diagrams of the DEGs (> 1.5-fold change, *padj* < 0.01) between stage I and stage IV, and GO terms enriched in the common up/down-regulated genes in testes. G: group-housed fish, S: single-housed fish.

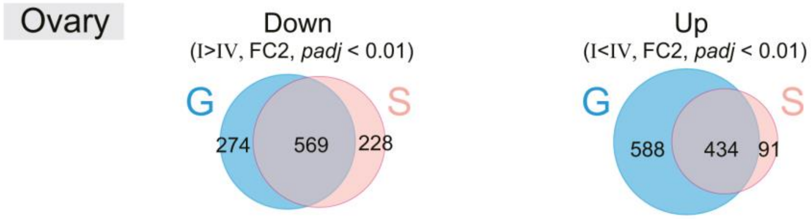

Down-regulated genes				
**Term**	**Description**	**LogP**	**Counts**	**Symbols**
GO:1904888	cranial skeletal system development	-4.07	18	s1pr2,bptf,usp36,ptpn11a,ptpn11b,mapk3,nek1,taok2a,furina,hdac4,chd7,vcpip1,josd1,usp37,si:dkey-88l16.3,taok2b,mosmoa,map2k7
GO:0098657	import into cell	-4.06	23	scarb2a,itsn1,necap1,slc12a2,gapvd1,slc7a3a,eea1,csnk1g1,slc27a4,dennd1a,dnm2a,epn2,slc38a2,inpp5f,epn1b,slc9a6a,epn3a,slc9a1b,eps15,si:dkey-88l16.3,rab22a,nalf1a,pla2r1
GO:0032869	cellular response to insulin stimulus	-4.02	6	cpeb1b,insig1,lpin2,si:ch73-335l21.1,pik3r3b,irs4a
GO:0050732	negative regulation of peptidyl-tyrosine phosphorylation	-3.63	3	cbl,cadm4,zfyve28
GO:0034248	regulation of amide metabolic process	-3.58	19	cpeb1b,igf2bp3,casc3,ormdl3,kbtbd8,larp4b,ddx6,stk35,pdik1l,ddx61,samd4a,larp4aa,tent5c,cnot3b,pum1,rc3h1b,tnrc6c1,celf2,rbfox2
GO:0051128	regulation of cellular component organization	-3.48	37	notch3,axin2,abhd17aa,trim37,gna13a,man1a2,baiap2l1a,c2cd5,rhogd,mapk3,clasp2,extl3,arhgap17a,msnb,cttnbp2,tbc1d25,sema6cb,bicd2,sptbn2,col15a1b,pik3ca,fignl2,clasp1a,camsap2a,plekhg2,cemip2,pld1b,dync1li2,limk1a,si:ch211-159i8.4,tgfa,plxnb2b,si:ch211-176g13.8,ssh1a,slc12a2,nde1,myo5b
GO:0008610	lipid biosynthetic process	-3.41	29	tlcd3ba,insig1,elovl7a,abhd2a,ptdss1a,serinc5,ormdl3,scdb,acer3,lpcat1,selenoi,lpin2,efr3bb,pik3ca,gnpat,pld1b,agpat2,pik3r3b,cds1,gal3st3,plcg1,slc7a3a,mtmr9,plce1,plpp5,slc27a4,fam135a,mboat1,sgpp1b
GO:0051056	regulation of small GTPase mediated signal transduction	-3.18	25	akap13,cadm4,arhgap17a,stard13a,dennd4c,ralgapb,sipa1l3,arhgef18b,sipa1l1,cdc42se1,il17rd,waca,ptpn11a,tsc1a,prkcbb,gpr137c,taok2a,sh3rf1,rictora,uacab,dlg5a,cds1,zdhhc17,taok2b,tsc1b
GO:0032924	activin receptor signaling pathway	-3.18	4	acvr1ba,smad2,prex1,tgfbr1b
GO:0043087	regulation of GTPase activity	-2.90	25	ralgps2,tbc1d16,tbc1d25,vav2,ralgapb,prex1,rab3gap1,ralgds,agap1,tbc1d2b,plxnb2b,hdr,uacab,scarb2a,axin2,trim37,cbl,si:dkey-7n6.2,ccny,dot1l,crtc3,zfyve28,ccnjl,stac,bcl10
GO:0016579	protein deubiquitination	-2.78	25	usp36,mindy2,stambpl1,vcpip1,josd1,usp37,zbtb16a,trim37,klhl17,ube2h,cdc34a,prkcbb,wdr32,uhrf1,rbbp6,cbl,rnf157,sh3rf1,dcun1d4,rnf19a,rc3h1b,kbtbd8,herc56.1,smurf2,ube2g1b
GO:0022600	digestive system process	-2.61	4	uhrf1,dot1l,myo5b,chd7
GO:0006468	protein phosphorylation	-2.56	20	acvr1ba,rps6kal,rps6ka3a,prkcbb,stk10,stk24b,csnk1g1,taok2a,wnk1b,prkacbb,hipk3a,lmtk2,cdc42bpab,stk35,pdik1l,tgfbr1b,taok2b,srms,mast3b,wnk2
GO:0003094	glomerular filtration	-2.49	5	myh9a,col4a4,tbc1d8b,farp1,plce1
GO:0060627	regulation of vesicle-mediated transport	-2.46	10	notch3,c2cd5,syt11a,arhgap1,msnb,rims1b,inpp5f,rab27a,rab8a,pld1b
GO:0034764	positive regulation of transmembrane transport	-2.43	4	c2cd5,wnk1b,stac,wnk2
GO:0051254	positive regulation of RNA metabolic process	-2.37	30	srfa,smad2,tefa,ncoa2,ell2,waca,cnot3b,ccnt1,smarcc1a,thrap3b,znf296,zgc:110158,rc3h1b,lpin2,tnrc6c1,tfeb,znf827,mllt1a,maml1,arid1b,crtc3,samd4a,hmbox1b,atad2b,lef1,igf2bp3,zbtb16a,tent5c,larp4b,larp4aa
GO:0015914	phospholipid transport	-2.35	6	pitpnm3,atp11c,mfsd2ab,atp11a,pitpnc1a,pitpnab
GO:0003002	regionalization	-2.34	26	acvr1ba,nr6a1a,smad2,lef1,hey1,axin2,il17rd,rbm14a,bptf,ptpn11a,man1a2,tsc1a,tle5,pum1,rbm14b,pnhd,lmo7b,frmd8,map2k7,rbpjb,furina,cemip2,chd7,llgl1,cdc42se1,ahcyl1
GO:0040007	growth	-2.26	15	acvr1ba,smad2,lef1,tlcd3ba,ap1g1,gna13a,ptpn11a,plk4,kif1b,plcg1,col4a6,myo9ab,kdm6bb,myo5b,mgat5
**Up-regulated genes**				
**Term**	**Description**	**LogP**	**Counts**	**Symbols**
GO:0006412	translation	-12.8	38	eef1a1l1,rack1,rpl3,rpl10a,rpl23a,rplp2,eef1a1a,rps14,rpl9,rpl6,rps15,rps27a,rpl13,faua,rps19,rpsa,rpl18a,rps24,rps2,rps11,rpl37,rpl39,rpl35a,rps6,rpl18,eif3ha,zgc:114188,rps27.1,rpl32,rps16,rps8b,gpx1a,chac1,ece1,sptlc2b,tlcd3bb,galcb,acot17
GO:0006119	oxidative phosphorylation	-10.6	18	CYTB,ND1,ND2,ND3,ND6,COX1,COX2,COX3,cox4i2,ndufb6,cox5ba,cox8a,sdhaf2,atp5f1e,ugp2b,aldocb,hk2,rpl37
GO:0000028	ribosomal small subunit assembly	-5.4	21	rps14,rps15,rps19,rpsa,rps27.1,rpl23a,rpl6,rps16,rps8b,rpl10a,rpl7a,rpl35a,gata4,csf1ra,sparc,arpc1b,rpl18,ada2b,slc7a7,eif3ha,rac2
GO:0002376	immune system process	-5.0	33	psmb8a,stat1a,pycard,csf1ra,psmb13a,sparc,ctsla,rpl10a,arpc1b,ifi30a,rps14,ctss2.2,mhc2dgb,rps19,irf9,rac2,rpl35a,ada,ccr10,rpl18,c1qc,ch25h,c1qa,rps27.1,cfp,mhc2d8.46a,cd276,b2ml,tnfsf14,ccl38.6,ninj1,ccl34a.4,ccl19a.2
GO:1901658	glycosyl compound catabolic process	-3.76	5	ada,fuca2,ada2b,gusb,aprt
GO:0030595	leukocyte chemotaxis	-3.30	36	csf1ra,rac2,ch25h,ccl38.6,ninj1,ccl34a.4,ccl19a.2,pycard,sgk1,fn1b,gmfb,ccr10,lrrc15,c5ar1,stat1a,anxa1a,il12bb,nfe2l2a,cpne3,cyp7a1,sh3bp4a,akr1a1a,grb10b,ifngr2,crfb2,cd276,hmox1a,asip1,nupr1b,serp2,rbp4,COX1,dio2,gadd45ba,chac1,si:ch1073-406l10.2
GO:0009145	purine nucleoside triphosphate biosynthetic process	-3.26	15	nme2b.1,nme7,ATP6,COX1,atp5f1e,aldocb,hk2,aprt,ada,adss2,ada2b,ugp2b,uap1l1,rbks,acot17
GO:2000027	regulation of animal organ morphogenesis	-2.98	4	csf1ra,klf2b,churc1,rspo3
GO:0051336	regulation of hydrolase activity	-2.59	13	pycard,scarb2a,ctsla,ctss2.2,mcl1b,kng1,serpinb1l1,arhgef19,si:ch211-247j9.1,apoc1,ccl38.6,ccl34a.4,ccl19a.2
GO:0010876	lipid localization	-2.53	13	rbp4,apoeb,fabp7a,fabp6,apoa4a,slc5a8l,esyt1a,spns3,pla2g1b,plin2,gltpd2a,npc1l1,aqp7
GO:0002181	cytoplasmic translation	-2.51	8	rplp2,rpl6,rpsa,rpl35a,eif3ha,rpl10a,rpl23a,rpl7a
GO:1901615	organic hydroxy compound metabolic process	-2.50	13	apoeb,aldh1a2,dio2,rdh1,dhcr7,cyp7a1,tpk1,ch25h,apoa4a,sptlc2b,comtb,ece1,hsd17b14
GO:0015671	oxygen transport	-2.46	3	si:ch211-5k11.8,cygb2,hbbe1.3
GO:0002399	MHC class II protein complex assembly	-2.46	3	mhc2dgb,mhc2d8.46a,b2ml
GO:0051014	actin filament severing	-2.32	10	gsna,capgb,gmfb,pfn2b,sptan1,hcls1,si:ch73-95l15.5,zgc:154093,arhgef19,rhof
GO:0000041	transition metal ion transport	-2.05	5	slc39a13,tcn2,fthl27,slc31a2,hephl1b
GO:0002521	leukocyte differentiation	-2.00	6	csf1ra,rpl10a,arpc1b,rac2,ada2b,slc7a7

Supplementary Figure 4. Venn diagrams of the DEGs (> 2-fold change, *padj* < 0.01) between stage I and stage IV, and GO terms enriched in the common up/down-regulated genes in ovaries. G: group-housed fish, S: single-housed fish.

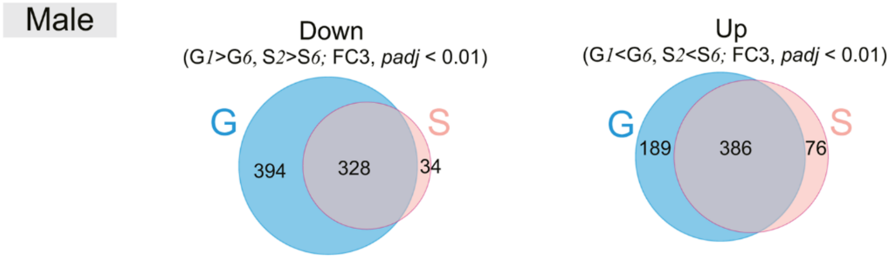

common down-regulated genes				
**Term**	**Description**	**LogP**	**Counts**	**Symbols**
GO:0007420	brain development	-7.96	27	dla,dlb,neurog1,pou3f3b,pou3f3a,pou3f1,eya1,ascl1a,ascl1b,otx2b,sox11a,robo2,pax6b,sox19b,tfap2a,ptprsa,meis2a,neurod4,tal2,pou4f2,her12,lhx9,lhx2b,notch1b,foxn4,ncanb,chd7
GO:0032989	cellular anatomical entity morphogenesis	-7.86	32	desma,mef2d,hsp90aa1.1,elavl4,scn8aa,robo2,ttn.2,tnika,lamb2,tardbpa,lhx9,myo18ab,kif5aa,wfs1a,plxna3,chd7,flncb,col19a1,lhx2b,si:ch211-159i8.4,notch1b,ttn.1,cyfip2,ptprdb,neurog1,ascl1a,neurod4,fryb,map4l,cdh15,cdh26.1,sox11a
GO:0071698	olfactory placode development	-6.73	11	neurog1,tfap2a,neurod4,tfap2c,lrrn1,chd7,eya1,wfs1a,dla,pax6b,sox19b
GO:0007626	locomotory behavior	-6.21	13	desma,scn8aa,slc1a2b,tardbpa,slc6a9,wfs1a,sv2a,flncb,gabra1,flnca,chata,chd7,lhx2b
GO:0031034	myosin filament assembly	-5.76	21	mef2d,hsp90aa1.1,ttn.2,ttn.1,desma,lamb2,tardbpa,mymk,mylpfb,itga6a,myo18ab,popdc2,mat2aa,flncb,si:dkeyp-69b9.3,limch1a,xirp2a,scn4ba,flnca,xirp2b,kifc1
GO:0050767	regulation of neurogenesis	-5.60	15	neurog1,ascl1a,ascl1b,elavl3,robo2,neurod4,hes6,her12,ncanb,plxna3,chd7,mymk,thrab,apc2,igf2bp3
GO:0042391	regulation of membrane potential	-5.46	11	scn8aa,slc6a9,gabrg2,gna11a,scn4ba,sv2a,grin1a,gabra1,glra2,popdc2,ank3a
GO:0007268	chemical synaptic transmission	-4.81	18	gria2b,gria3b,syt1a,slc6a9,gabrg2,syt2a,sv2a,slc12a5a,grin1a,gabra1,glra2,slc17a6b,chata,gjc4b,gja2,gje1a,dot1l,apc2
GO:0045944	positive regulation of transcription by RNA polymerase II	-4.70	19	neurog1,foxn4,ascl1a,ascl1b,mef2d,sox11a,pax6b,sox19b,meis2a,neurod4,abraa,thrab,kmt2cb,si:dkeyp-69b9.3,hsp90aa1.1,igf2bp3,celf3a,scamp5a,eya1
GO:0006836	neurotransmitter transport	-4.41	22	syt1a,slc6a1b,slc6a9,syt2a,sv2a,slc6a1a,slc17a6b,scn8aa,gabrg2,slc8a3,cacna2d1a,kcnma1a,slc12a5a,grin1a,gabra1,glra2,lrrc38b,cacng6b,trpm4a,gria2b,gria3b,scn4ba
GO:0021772	olfactory bulb development	-4.35	8	neurog1,robo2,ptprsa,lhx2b,lrrn1,chd7,notch1b,tfap2a
GO:0007423	sensory organ development	-3.87	21	dla,neurog1,foxn4,eya1,ascl1a,ascl1b,otx2b,sox11a,pax6b,tfap2a,meis2a,neurod4,lamb2,rab11fip4a,tfap2c,igf2bp1,wnk1b,wfs1a,chd7,lhx2b,cyfip2
GO:0021514	ventral spinal cord interneuron differentiation	-3.82	10	dla,foxn4,tal2,notch1b,dlb,her12,otud7b,neurog1,robo2,otx2b
GO:0008016	regulation of heart contraction	-3.63	7	desma,foxn4,ank2b,sorbs2a,popdc2,trdn,mymk
GO:0006865	amino acid transport	-3.48	7	slc1a2b,slc6a1b,slc6a9,slc6a1a,slc7a5,slc17a6b,slc38a3b
GO:1901888	regulation of cell junction assembly	-3.24	4	robo2,limch1a,macf1a,si:ch211-159i8.4
GO:0030902	hindbrain development	-3.07	8	neurog1,ascl1a,ascl1b,otx2b,pax6b,tfap2a,notch1b,neurod4
GO:0048488	synaptic vesicle endocytosis	-3.06	9	syt1a,si:ch211-200p22.4,syt2a,dnm1a,kif5aa,slc17a6b,scamp5a,slc1a2b,cpne5a
GO:0043484	regulation of RNA splicing	-2.97	6	celf3a,hmga1a,nova2,ahnak,znf638,prx
GO:0034762	regulation of transmembrane transport	-2.93	12	desma,scn8aa,slc1a2b,wnk1b,cacna2d1a,scn4ba,kcnma1a,cacng6b,ank3a,scamp5a,syt1a,syt2a
**common up-regulated genes**				
**Term**	**Description**	**LogP**	**Counts**	**Symbols**
GO:0006956	complement activation	-10.75	42	cfb,c8g,c8a,c1qc,c1qa,c7a,c7b,c4b,cd74b,cd74a,mhc2dgb,fadd,mhc2d8.46a,b2ml,rac2,cxcr3.2,mmp9,gata1a,lgals9l3,wasb,grnb,cxcl8a,gapdh,f2,ctss2.2,fga,gh1,ctsl.1,tm4sf21b,ccl25b,c5ar1,ccl38.6,ccl35.2,psmb8a,psmb13a,alas2,ifi44f1,zgc:174904,casp3b,pglyrp6,mhc1uka,si:ch1073-280e3.1
GO:0050878	regulation of body fluid levels	-7.47	19	cel.1,f2,fgb,serpind1,fga,fgg,prozb,cel.2,f9b,serpina10b,f7l,mmp9,rac2,cxcl8a,cd74b,cd74a,zp2l1,cxcr3.2,spire2
GO:0044419	biological process involved in interspecies interaction between organisms	-6.71	35	cfb,ncf1,ldlra,mmp9,rac2,c8a,mogat2,c7a,zgc:174904,lect2.1,c7b,marco,pglyrp6,cxcr3.2,ccl25b,cxcl8a,c1ql4l,ccl38.6,ccl35.2,asgr1a,f2,wasb,chad,gh1,gcga,tcap,bglap,c5ar1,cd74b,cd74a,si:ch1073-280e3.1,si:ch211-160b11.4,si:ch1073-406l10.2,si:dkey-9i23.4,si:dkey-9i23.4
GO:0042632	cholesterol homeostasis	-6.54	27	fabp2,ldlra,lipca,cyp7a1,cetp,abcg5,abcg8,apoa1a,apoea,dhrs9,cyp27a1.1,cyp8b1.1,srd5a2a,cahz,fth1a,hpxa,slc34a2b,fthl27,slc34a1a,adh8a,dio2,miox,crym,gata1a,alas2,rac2,casp3b
GO:0019752	carboxylic acid metabolic process	-6.04	55	agxtb,gpib,gapdh,aldob,bhmt,aldoaa,alox5ap,eno3,acad11,lipca,hpda,cyp7a1,amdhd1,agxta,zgc:92040,pkmb,aldh8a1,haao,cbsa,scdb,zgc:153372,hal,si:ch211-217a12.1,aldh1l1,cyp2x8,cel.1,adh8a,gpx1a,dnase1,hbba2,neu3.2,si:ch211-5k11.8,cel.2,nme2b.1,nme4,psmb8a,psmb13a,psma6l,ctss2.2,ctsc,ctsba,chia.3,ctsl.1,pglyrp6,ugp2b,amy2al1,fbp2,slc3a1,gyg2,si,chst6,cox4i2,tcap,cox8b,cox7a1
GO:0015669	gas transport	-4.86	7	hbba1,cahz,mb,hbba2,si:ch211-5k11.8,gpx1a,ncf1
GO:0170033	L-amino acid metabolic process	-4.20	16	agxtb,bhmt,hpda,amdhd1,agxta,zgc:92040,aldh8a1,cbsa,hal,si:ch211-217a12.1,haao,adh8a,acad11,cyp7a1,miox,aldh1l1
GO:0019882	antigen processing and presentation	-4.06	12	cd74b,cd74a,mhc2dgb,mhc2d8.46a,b2ml,gapdh,f2,grnb,fga,acanb,mep1a.2,agrp
GO:0006641	triglyceride metabolic process	-3.86	5	lipca,mogat2,cetp,agpat9l,apoc1
GO:0006575	cellular modified amino acid metabolic process	-3.47	8	bhmt,ckmt2a,gpx1a,dio2,gstt1a,crym,chdh,aldh1l1
GO:0000302	response to reactive oxygen species	-3.41	12	gpx1a,mb,apoda.1,apoda.2,adh8a,ldlra,cyp7a1,gh1,scdb,sult2st3,bglap,cxcl8a
GO:0048251	elastic fiber assembly	-3.40	8	mfap4.1,mfap4.12,mfap4.6,loxa,mmp9,paplnb,dpt,col10a1b
GO:0070887	cellular response to chemical stimulus	-3.38	30	agxtb,ucp1,cpb1,adh8a,hpxa,wasb,ldlra,amy2al1,mb,cyp7a1,slc13a2,nr5a5,gh1,rac2,cyp2x8,zgc:153372,crfb2,cxcr3.2,ccl25b,cxcl8a,ccl38.6,agrp,ccl35.2,serp2,fabp2,dio2,lipca,scdb,sult2st3,bglap
GO:0044283	small molecule biosynthetic process	-3.21	13	apoa1a,agxtb,gpib,bhmt,alox5ap,lipca,cyp7a1,cyp27a1.1,agxta,fbp2,haao,cbsa,scdb
GO:0050994	regulation of lipid catabolic process	-3.14	3	gh1,apoc1,si:ch1073-406l10.2
GO:0002697	regulation of immune effector process	-2.85	6	cd74b,cd74a,mmp9,cxcl8a,cxcr3.2,gh1
GO:0052547	regulation of peptidase activity	-2.83	11	psme2,ctss2.2,serpind1,ctsl.1,serpinb1,serpina7,serping1,apoc1,ccl25b,ccl38.6,ccl35.2
GO:0042445	hormone metabolic process	-2.77	6	dhrs9,dio2,aldh8a1,srd5a2a,crym,nmbb
GO:0016042	lipid catabolic process	-2.63	9	cel.1,pla1a,acad11,lipca,cyp7a1,pla2g10,neu3.2,cel.2,pla2g1b

Supplementary Figure 5. Venn diagrams of the DEGs (> 3-fold change, *padj* < 0.01) between stage *1/2* and stage *6*, and GO terms enriched in the common up/down-regulated genes in male juveniles. G: group-housed fish, S: single-housed.

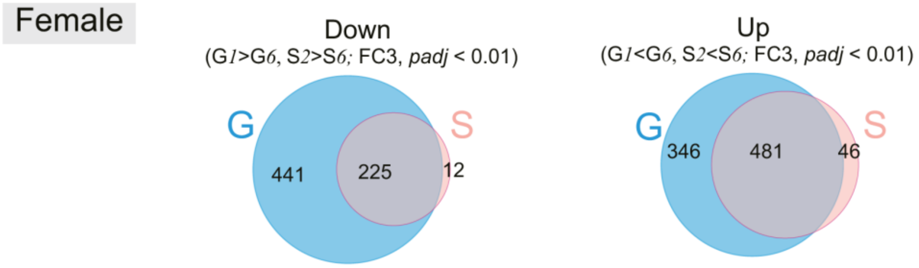

common down-regulated genes				
**Term**	**Description**	**LogP**	**Counts**	**Symbols**
GO:0061061	muscle structure development	-8.69	22	desma,myog,mef2d,hsp90aa1.1,ttn.2,mat2aa,lamb2,tardbpa,rbfox1l,mylpfb,itga6a,flncb,si:dkeyp-69b9.3,nrap,popdc2,mef2cb,ttn.1,limch1a,xirp2a,scn4ba,flnca,xirp2b
GO:0032989	cellular anatomical entity morphogenesis	-7.79	24	desma,mef2d,hsp90aa1.1,elavl4,scn8aa,ttn.2,lamb2,tardbpa,lhx9,wfs1a,plxna3,flncb,col19a1,nrap,lhx2b,notch1b,ttn.1,ptprdb,her4.2,cdh15,cdh26.1,neurog1,neurod4,sox11a
GO:0071698	olfactory placode development	-7.69	16	neurog1,tfap2a,neurod4,tfap2c,lrrn1,eya1,wfs1a,sox11a,meis2a,col27a1b,plxna3,popdc2,mef2cb,grhl2b,pax6b,rab11fip4a
GO:0007420	brain development	-5.04	20	neurog1,pou3f1,eya1,ascl1b,otx2b,sox11a,pax6b,tfap2a,ptprsa,meis2a,neurod4,lhx9,lhx2b,notch1b,grhl2b,lamb2,rab11fip4a,tfap2c,igf2bp1,wfs1a
GO:0007626	locomotory behavior	-5.04	9	desma,scn8aa,tardbpa,wfs1a,flncb,nrap,flnca,chata,lhx2b
GO:0043484	regulation of RNA splicing	-5.01	7	celf3a,hmga1a,rbfox1l,nova2,ahnak,znf638,prx
GO:0045944	positive regulation of transcription by RNA polymerase II	-4.60	19	myog,neurog1,ascl1b,mef2d,sox11a,pax6b,meis2a,neurod4,thrab,si:dkeyp-69b9.3,mef2cb,elavl3,hes6,plxna3,her4.2,hsp90aa1.1,celf3a,larp4b,eya1
GO:0021536	diencephalon development	-4.42	8	neurog1,eya1,pax6b,neurod4,lhx9,lhx2b,ptprsa,lrrn1
GO:0048738	cardiac muscle tissue development	-4.03	13	ttn.2,mat2aa,rbfox1l,nrap,mef2cb,ttn.1,mef2d,dnmt3ba,thrab,kdm6bb,plxna3,flncb,popdc2
GO:0034765	regulation of monoatomic ion transmembrane transport	-3.82	14	desma,scn8aa,cacna2d1a,scn4ba,kcnma1a,cacng1b,scn4aa,cacng6b,ano1a,slc6a1b,gabrg2,glra2,gria2b,gria3b
GO:0008016	regulation of heart contraction	-3.61	5	desma,ank2b,sorbs2a,popdc2,trdn
GO:0048934	peripheral nervous system neuron differentiation	-3.37	4	neurog1,notch1b,her4.2,tfap2a
GO:0110020	regulation of actomyosin structure organization	-3.20	3	thrab,limch1a,nrap
GO:0042391	regulation of membrane potential	-2.83	6	scn8aa,gabrg2,scn4ba,scn4aa,glra2,popdc2
GO:0034330	cell junction organization	-2.82	13	ptprsa,tardbpa,gabrg2,cdh15,cdh26.1,ppfia4,ptprdb,notch1b,si:ch73-74h11.1,lamb2,itga6a,frem3,col28a2a
GO:0006936	muscle contraction	-2.15	4	desma,gja2,scn4ba,myhb
GO:1902476	chloride transmembrane transport	-2.11	3	ano1a,gabrg2,glra2
GO:0030198	extracellular matrix organization	-2.09	5	lamb2,col27a1b,adamts12,col28a2a,colq

common up-regulated genes				
**Term**	**Description**	**LogP**	**Counts**	**Symbols**
GO:0010876	lipid localization	-11.05	28	apoa1a,fabp2,fabp10a,ldlra,pla2g12b,fabp6,pla2g10,cd36,fabp4a,cetp,apoa4a,apoea,abcg5,vtg6,vtg2,ldah,rbp2a,afp4,apoc2,rbp7b,apoa4b.2,pla2g1b,apol,abcg8,apoda.2,star2,slc13a2,slc25a5
GO:0071391	cellular response to estrogen stimulus	-9.61	33	ucp1,esr1,fbp1b,ssr1,amy2al1,aqp12,vtg6,vtg2,nupr1b,fam20cl,cpb1,adh8a,wasb,rhogb,ldlra,mb,slc13a2,lta4h,nr5a5,cyp2x8,nt5c2l1,cyp2n13,cyp2x9,crfb2,prdx2,hmox1a,cxcr3.2,ccl25b,ccl38.6,ccl35.2,serp2,dio2,cd36
GO:0006956	complement activation	-8.27	50	cfb,c8g,c8a,c1qc,c1qa,c7a,c4b,gata1a,cd74b,psmb8a,psmb13a,alas2,alas1,wasb,arpc1b,ifi30a,ctss2.2,mhc2dgb,vtnb,lta4h,cd36,ctsl.1,ada,zgc:110239,ifi44f1,wfdc2,slc15a2,zgc:174904,casp3b,mhc2d8.46a,pglyrp6,mhc1uka,prdx2,b2ml,cxcr3.2,irf1b,ccl25b,ccl38.6,ccl35.2,fgl1,lgals9l3,grnb,mmp9,hmox1a,gapdh,f2,tm4sf21b,prl,c5ar1,si:ch211-5k11.8
GO:0055088	lipid homeostasis	-7.74	40	apoa1a,fabp2,ldlra,pla2g12b,pnpla3,cetp,apoa4a,apoea,abcg5,apoa4b.2,tm6sf2b,abcg8,hsd17b1,faxdc2,cyp8b1.1,hsd3b1,apof,star2,cahz,fth1a,nherf1a,fthl27,fthl31,prl,slc34a1a,fah,adh8a,dio2,tpi1b,crym,gata1a,alas2,alas1,casp3b,prdx4,prdx2,afp4,apoc2,apoc1,apol
GO:0044248	cellular catabolic process	-7.13	83	pklr,gpib,gapdh,aldob,bhmt,fah,cel.1,adh8a,aldoaa,gpx1a,eno3,upp2,hpda,amdhd1,hao1,amdhd2,hoga1,lta4h,uox,pnpla3,cd36,ada,dnase1,hbba2,pkmb,aldh8a1,rfesd,urad,tpi1b,tat,cel.2,prdx4,prdx2,hmox1a,aldh1l1,psmb8a,psmb13a,psma6l,ctss2.2,ctsc,lgmn,ctsba,chia.3,ctsl.1,ctsz,zgc:110239,apoc2,pglyrp6,papss2b,alox5ap,cyp2x8,agxta,cyp2n13,scdb,acot17,cyp2x9,ugp2b,cox4i2,tcap,gyg2,cox8b,cox7a1,ccn6,nme2b.1,fbp1b,amy2al1,b3gat1a,si,uap1,pnp4b,ckmt1,ckmt2a,pla2g12b,pla2g10,gpat3,cetp,pla2g1b,rarres3,plaat1l,itih3a.1,ogal,fut9a,si:ch211-5k11.8
GO:0044282	small molecule catabolic process	-4.69	15	fah,adh8a,upp2,hpda,amdhd1,hao1,amdhd2,hoga1,uox,ada,aldh8a1,urad,tpi1b,tat,aldh1l1
GO:0042632	cholesterol homeostasis	-4.65	6	fabp2,ldlra,pla2g12b,cetp,abcg5,abcg8
GO:0044419	biological process involved in interspecies interaction between organisms	-4.35	34	cfb,rhogb,ldlra,mmp9,lta4h,cd36,c8a,mogat2,wfdc2,slc15a2,c7a,zgc:174904,pglyrp6,cxcr3.2,irf1b,ccl25b,c1ql4l,ccl38.6,ccl35.2,asgr1a,dap,wasb,chad,gcga,tcap,prl,bglap,nupr1b,c5ar1,f2,si:ch1073-406l10.2,si:dkey-9i23.4,si:dkey-9i23.4,si:ch1073-280e3.1
GO:0015669	gas transport	-4.34	13	hbba1,cahz,mb,hbba2,gpx1a,prdx4,prdx2,gpx4a,msrb2,hmox1a,apoda.2,adh8a,si:ch211-5k11.8
GO:0006575	cellular modified amino acid metabolic process	-4.12	10	ckmt1,bhmt,ckmt2a,gpx1a,dio2,gstt1a,crym,aldh9a1a.1,chdh,aldh1l1
GO:0050878	regulation of body fluid levels	-3.78	11	f7i,cel.1,f2,cel.2,f9b,cpb2,fgl1,f7l,mmp9,lgmn,b3gat1a
GO:0030199	collagen fibril organization	-3.63	20	loxa,mmp9,dpt,col2a1a,loxl4,mibp2,mfap4.1,paplnb,col10a1b,mfap4.12,lcp1,wasb,arpc1b,rhogb,evlb,scinlb,spire2,tcap,smyd1b,nanog
GO:0009410	response to xenobiotic stimulus	-3.56	8	esr1,cpb1,adh8a,cyp2x8,nt5c2l1,cyp2n13,cyp2x9,hmox1a
GO:0052547	regulation of peptidase activity	-3.51	13	psme2,ctss2.2,ctsl.1,serpinb1,zgc:110239,serpina7,serping1,si:ch211-262h13.5,apoc1,ccl25b,ccl38.6,ccl35.2,si:ch211-5k11.8
GO:0006641	triglyceride metabolic process	-3.36	10	pnpla3,gpat3,mogat2,cetp,apoc1,si:ch1073-406l10.2,cd36,ldlra,thrsp,tm6sf2b
GO:0006880	intracellular sequestering of iron ion	-3.23	6	fth1a,fthl27,fthl31,cd36,ldah,meltf
GO:0046348	amino sugar catabolic process	-3.08	4	amdhd2,hoga1,chia.3,uap1
GO:0001775	cell activation	-2.86	10	zp2.5,cd74b,f2,arpc1b,ada,zp2l1,prdx2,cxcr3.2,fgl1,zp3a.2
GO:0030388	fructose 1,6-bisphosphate metabolic process	-2.73	3	fbp1b,aldob,aldoaa
GO:0019882	antigen processing and presentation	-2.56	4	cd74b,mhc2dgb,mhc2d8.46a,b2ml

Supplementary Figure 6. Venn diagrams of the DEGs (> 3-fold change, *padj* < 0.01) between stage *1/2* and stage *6*, and GO terms enriched in the common up/down-regulated genes in female juveniles. G: group-housed fish, S: single-housed.

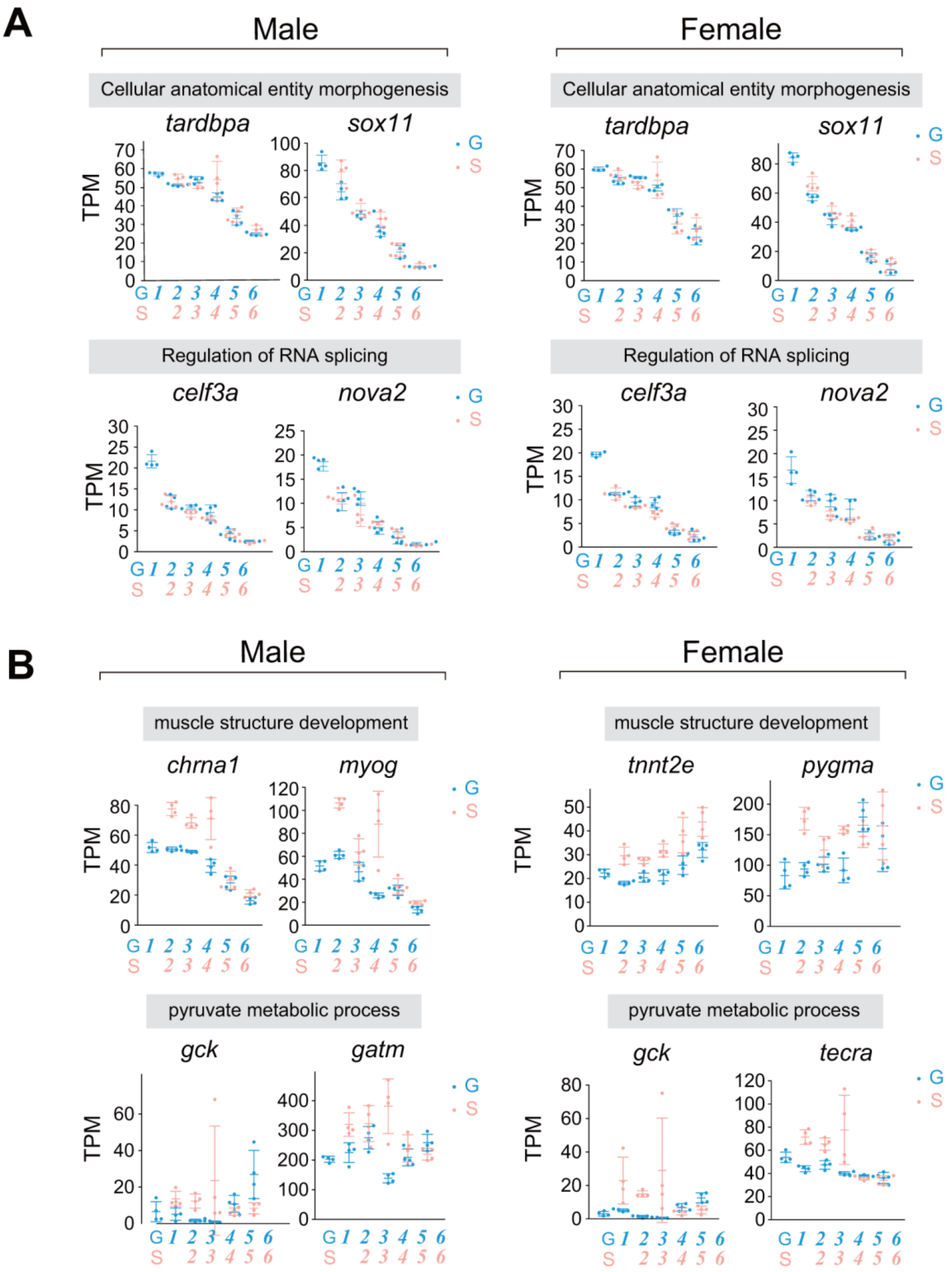

Supplementary Figure 7. Changes in the expression of genes associated with GO terms that are enriched in the DEGs between group- and single-housed fish. (**A**) Example of expression of the cellular anatomical entity morphogenesis/regulation of RNA splicing-associated genes that were enriched among the common DEGs (stage *1*/*2* vs. stage *6*) between group- and single-housed fish. TPM values are shown. *tardbpa*, *TAR DNA binding protein a*; *sox11*, *SRY-box transcription factor 11*; *celf3a*, *Elav-like family member 3a*; *nova2*, *NOVA alternative splicing regulator 2* (**B**) Example of the expression of muscle structure development/pyruvate metabolic process-associated genes that were enriched among the DEGs with higher expression in the single-housed fish than in the group-housed fish in stages *1-4*. TPM values are shown. *chrna1*, *cholinergic receptor, nicotinic, alpha 1*; *myog*, *myogenin*; *tnnt2e*, *troponin T2e*; *pygma*, *phosphorylase, glycogen, muscle A*; *gck*, *glucokinase*; *gatm*, *glycine amidinotransferase*; *tecra*, *trans-2,3-enoyl-CoA reductase a.* (**A**, **B**) G: group-housed fish, S: single-housed fish.
