# Supplementary material for: Single housing of juveniles accelerates early-stage growth but extends adult lifespan in African turquoise killifish
Source: Aging (Albany NY). 2024 Sep 16;16(18):12443–72. doi: 10.18632/aging.206111 (PMC11466477; doi:10.18632/aging.206111)
Supplement: Supplementary Table 1 [file aging-16-206111-s002.docx]

Supplementary Table 1: GO term enrichment analysis of age-related genes in livers.

| **274 common genes** | |  |  |  |
| --- | --- | --- | --- | --- |
| **Term** | **Description** | **LogP** | **Counts** | **Symbols** |
| GO:1901605 | alpha-amino acid metabolic process | -5.61 | 24 | cbsb,ctps1a,bhmt,adi1,pah,got1,amt,pfas,oat,glula,prodha,eno1a,cyp2p6,dbi,amdhd2,cyp2x8,scdb,acsl1a,abcd1,gch2,hsd17b3,hprt1,tm7sf2,mat1a |
| GO:0019218 | regulation of steroid metabolic process | -4.09 | 6 | fgf1a,scap,fgfr4,apoa4b.2,pnpla3,cbsb |
| GO:0015711 | organic anion transport | -4.07 | 11 | slc25a5,slc43a1a,slc13a3,slc51a,slc35b1,slc13a5a,slc6a6a,slc16a6b,abcd1,slc16a4,rbp2a |
| GO:0006629 | lipid metabolic process | -3.45 | 26 | bco1,hsd11b2,hsd17b3,dbi,faxdc2,pnpla3,bpnt1,mogat2,tm7sf2,fdps,hsd3b1,scdb,acsl1a,pcyt1bb,scap,gba2,abhd15a,pigl,abcd1,apoba,agpat2,gch2,ctps1a,hprt1,ppcs,pfas |
| GO:0009064 | glutamine family amino acid metabolic process | -3.16 | 5 | ctps1a,pfas,oat,glula,prodha |
| GO:0044248 | cellular catabolic process | -2.94 | 17 | bco1,bhmt,adh8a,pah,cdab,amdhd2,mao,pnpla3,entpd1,amt,sec16b,prdx5,gba2,abcd1,pde8b,oat,prodha |
| GO:0015850 | organic hydroxy compound transport | -2.92 | 10 | slc51a,abcd1,apoba,aqp9b,apoa4b.2,pltp,scap,rbp2a,afp4,vps13c |
| GO:1901136 | carbohydrate derivative catabolic process | -2.74 | 12 | nccrp1,gpd1b,cdab,amdhd2,gba2,pde8b,eno1a,amy2al1,ppp1r3cb,khk,b4galt1l,si:ch73-91k6.2 |
| GO:0045834 | positive regulation of lipid metabolic process | -2.59 | 6 | fgf1a,pnpla3,apoa4b.2,angptl4,apoba,mogat2 |
| GO:0009719 | response to endogenous stimulus | -2.56 | 21 | tbx2b,thrb,smad2,dio2,timp2a,pah,amy2al1,fgf1a,khk,ghrb,chrm3a,fgfr4,ptger4b,nccrp1,cyp1a,adh8a,calr,hspa5,entpd1,scdb,dnajb9a |
| GO:1901615 | organic hydroxy compound metabolic process | -2.43 | 9 | gch2,adh8a,hsd11b2,dio2,hsd17b3,mao,faxdc2,tm7sf2,hsd3b1 |
| GO:0072009 | nephron epithelium development | -2.40 | 4 | tbx2b,slc43a1a,fgf1a,brca2 |
| GO:0007626 | locomotory behavior | -2.36 | 6 | fhl1a,bag3,ca8,scdb,abcd1,cln5 |
| GO:0030433 | ubiquitin-dependent ERAD pathway | -2.27 | 4 | nccrp1,calr,hspa5,dnajb9a |
| GO:0055123 | digestive system development | -2.12 | 6 | sdc2,utp25,bhmt,rps6ka3a,pllp,cldn15a |
| GO:0009116 | nucleoside metabolic process | -2.12 | 3 | cdab,hprt1,pnp5a |
|  |  |  |  |  |
| **213 single-housed fish specific age-related genes** | |  |  |  |
| **Term** | **Description** | **LogP** | **Counts** | **Symbols** |
| GO:0008202 | steroid metabolic process | -7.26 | 34 | apoeb,insig1,dhcr7,sc5d,mvk,dhcr24,rdh5,hmgcra,cyb5r2,cyp27b1,gatm,fbp1b,ip6k2a,dpydb,ada,csad,zgc:153031,aasdhppt,mtmr3,faah2a,st3gal8,smpd1,pparaa,acot17,fmo5,pik3r3b,hdhd5,pnpla2,fah,nr1d2b,alas2,uck1,gnpnat1,taf7 |
| GO:0044282 | small molecule catabolic process | -3.77 | 25 | apoeb,gldc,fah,gpt,hoga1,dpydb,tdh,ada,si:ch211-217a12.1,gatm,csad,zgc:153031,pparaa,acot17,aasdhppt,gck,si:ch211-5k11.8,prdx1,smpd1,atp13a2,ulk2,psmb8a,psmb13a,sat1a.2,psme4a |
| GO:0032922 | circadian regulation of gene expression | -3.46 | 4 | per3,id2a,bhlhe40,per1b |
| GO:0019755 | one-carbon compound transport | -2.99 | 13 | slc4a1a,rhag,slc4a4b,atp6ap1a,ccnd1,alas2,angptl4,prdx1,abcc6a,atp13a2,gck,rh50,zgc:162608 |
| GO:0032870 | cellular response to hormone stimulus | -2.90 | 16 | nr1d2b,ucp1,fbp1b,insig1,grb10b,pparaa,pik3r3b,id2a,fgf19,htr1ab,sesn2,egln3,prdx1,atp13a2,ppifb,crfb2 |
| GO:0045834 | positive regulation of lipid metabolic process | -2.88 | 5 | pparaa,zgc:162608,pnpla2,egln3,cptp |
| GO:0001878 | response to yeast | -2.88 | 3 | sc5d,xpo1b,cyb5r2 |
| GO:0015711 | organic anion transport | -2.87 | 12 | slc4a1a,rbp5,mfsd2ab,fabp4a,slc16a12a,slc4a4b,slc25a29,cptp,apoeb,plin2,zgc:162608,slc37a4b |
| GO:0009225 | nucleotide-sugar metabolic process | -2.83 | 4 | ugp2b,gne,gnpnat1,hoga1 |
| GO:0042398 | cellular modified amino acid biosynthetic process | -2.78 | 5 | gatm,zgc:153031,ckba,rdh5,cyp27b1 |
| GO:0097435 | supramolecular fiber organization | -2.62 | 13 | tmod4,col1a1a,dnajb6b,mfap4.1,rhoaa,rhoac,rhobtb4,shroom1,sh3d21,tnnt3b,svilc,si:ch211-266i6.3,pdlim4 |
| GO:0018208 | peptidyl-proline modification | -2.52 | 3 | egln3,p4ha3,ppifb |
| GO:0015850 | organic hydroxy compound transport | -2.44 | 5 | apoeb,aqp7,aqp9b,zgc:162608,mfsd2ab |
| GO:0001666 | response to hypoxia | -2.41 | 4 | hbba1,alas2,igfbp1a,egln3 |
| GO:0035249 | synaptic transmission, glutamatergic | -2.40 | 3 | gria1b,grik4,grid1a |
| GO:0019637 | organophosphate metabolic process | -2.31 | 20 | fbp1b,ip6k2a,mtmr3,ada,uck1,mvk,smpd1,hmgcra,ckba,acot17,gck,pik3r3b,hdhd5,ugp2b,gne,dpydb,gnpnat1,hoga1,st3gal8,b3gnt3.4 |
| GO:0010506 | regulation of autophagy | -2.21 | 4 | mtmr3,cptp,sesn2,ulk2 |

(continued)

| **1101 group-housed fish specific age-related genes** | |  |  |  |
| --- | --- | --- | --- | --- |
| **Term** | **Description** | **LogP** | **Counts** | **Symbols** |
| GO:0042254 | ribosome biogenesis | -32.8 | 104 | pes,mak16,npm1a,sdad1,ddx54,airim,wdr43,nifk,ppan,snu13b,ddx18,nop2,nop14,ftsj3,nsa2,gnl3,rpl7l1,rpl10a,mphosph10,tsr1,utp15,esf1,rrp15,gtpbp4,ebna1bp2,rrs1,ddx27,ngdn,eif6,utp6,pno1,wdr46,heatr3,gar1,bysl,rps21,nop56,bxdc2,rsl24d1,fbl,abce1,rpf2,nsun5,rbm34,gtpbp10,gnl3l,grwd1,nop10,polr1d,utp3,nop58,nmd3,rrp7a,mrto4,tma16,trmt112,noc4l,naf1,bud23,emg1,ncl,gnl2,pdcd11,rrp8,nhp2,eif3ea,eif2s3,eif3s10,eif3m,eif3d,eif3g,ptges3a,eif3ha,snrpd2,eif3k,eif3ba,pelo,polr1e,ssb,aars1,sars2,rpp21,tars1,tsen54,thumpd3,qng1,nars1,lars1b,ints9,prpf19,prpf40a,hnrnpa0l,ddx46,rnps1,trmt2a,ssu72,srsf7a,rbm4.3,srsf2a,snrnp70,rbm39b,tbrg4,si:dkey-251i10.3,si:ch211-256m1.8 |
| GO:0043603 | amide metabolic process | -12.5 | 72 | cers2a,eif2s1b,cel.1,rpl3,rpl10a,aars1,eif3ea,eif2s3,eif3s10,eif3m,eif3d,drg1,rps27a,eif6,eif3g,rpl28,pelo,mrpl14,asns,sars2,rps21,sucla2,abce1,galcb,aimp2,rps11,eif4a2,mrrf,mrps15,rwdd1,mrpl11,acss2,aclya,mrps23,eif3ha,ass1,tars1,mrps16,ormdl3,mvda,eif4eb,mrpl10,smpdl3a,eif3k,mrpl12,gm2a,mrpl13,mrps17,urad,cerk,acaca,pcsk5b,gfm1,mrps5,cel.2,eif2ak1,eef1da,tsfm,nars1,lars1b,eif2a,rpl22l1,rarres3,gal3st1a,si:ch211-256m1.8,eif3ba,mrpl51,eif4ebp1,pmvk,gls2a,mrpl22,shmt2 |
| GO:0006629 | lipid metabolic process | -10.4 | 82 | lpla,ppardb,fads2,cers2a,rdh8a,apobb.1,cel.1,hsd17b12b,inppl1a,tecrb,lpcat4,pla1a,eci1,gnpat2,hadhb,dhcr7,pck1,bco1l,acadl,pla2g12a,mttp,acaa2,galcb,ptges3a,msmo1,pi4k2a,pnpla3,sccpdha.1,acss2,aclya,hsd17b14,acox1,cpt1b,mogat2,ormdl3,slc7a3a,mvda,slc27a1a,smpdl3a,abhd4,pcyt1aa,gm2a,lratb.1,cyp46a1.1,apoea,amacr,lpcat2,scdb,elovl8b,pik3r1,plpp3,cerk,abhd6b,acaca,rft1,cpt1ab,cel.2,dgat2,cyp27a3,agpat9l,apoc1,pla2g1b,inpp5l,cish,rarres3,ebpl,hsd17b7,gal3st1a,hadhab,ptena,fmo5,dpm2,lpin1a,ehhadh,sftpbb,pmvk,cyp24a1,ccdc57,gpam,pnpla2,si:ch211-117n7.7,si:ch211-256m1.8 |
| GO:0043436 | oxoacid metabolic process | -9.64 | 73 | lpla,ppardb,agxtb,papss2b,ugdh,fads2,gatm,hsd17b12b,aars1,tecrb,eci1,gnpat2,hadhb,il4i1,kmo,pck1,abat,pycr1a,idh3a,hibadhb,asns,sars2,acadl,nit2,acaa2,got1,hk2,mdh2,ptges3a,tha1,acss2,aclya,me2,ass1,sdhaf3,tars1,acox1,cpt1b,slc7a3a,accs,amacr,scdb,elovl8b,agmat,acaca,cpt1ab,qdpra,nars1,lars1b,zgc:153372,hadhab,lpin1a,ehhadh,gls2a,idh1,aspg,shmt2,ccdc57,gpam,aldh1l1,cyp2x8,cyp2u1,ada2a,dhcr7,aprt,pcbd1,msmo1,mvda,pdxkb,cyp27a3,hsd17b7,pmvk,si:ch211-256m1.8 |
| GO:0006457 | protein folding | -8.32 | 36 | calr3a,hsp90ab1,dnajc3b,cct6a,cct7,cct2,cct3,dnajc3a,hspa4b,dnaja3a,fkbp5,hspa9,hsp90b1,dnajb11,cct4,erp44,nudc,cct8,dnajc2,txndc5,ptges3a,p4hb,canx,tor2a,hspa8,mesd,tbcelb,fgb,fga,phex,lgmn,pcsk5b,afg3l1,bmp1a,si:ch211-256m1.8,isca2 |
| GO:0016042 | lipid catabolic process | -8.22 | 66 | lpla,cel.1,pla1a,eci1,hadhb,pck1,bco1l,acadl,pla2g12a,acaa2,galcb,pnpla3,hsd17b14,acox1,cpt1b,smpdl3a,gm2a,cyp46a1.1,apoea,abhd6b,cpt1ab,cel.2,pla2g1b,si:ch211-117n7.7,hadhab,lpin1a,ehhadh,cyp24a1,pnpla2,adh8a,il4i1,kmo,ada2a,abat,hibadhb,rbks,tha1,uox,urad,qdpra,tkfc,gls2a,shmt2,aldh1l1,urod,snd1,cnp,vmp1,pelo,sqstm1,snx7,lamp2,mrto4,dnph1,enpp1,tp53inp1,prdx4,hspa8,hmox1a,sting1,inppl1a,plpp3,inpp5l,ptena,si:ch211-256m1.8,si:ch211-260e23.9 |
| GO:0006641 | triglyceride metabolic process | -7.71 | 25 | lpla,apobb.1,gnpat2,pck1,pnpla3,mogat2,slc7a3a,dgat2,agpat9l,apoc1,lpin1a,gpam,abhd6b,inppl1a,pi4k2a,slc27a1a,abhd4,pcyt1aa,pik3r1,inpp5l,cish,rarres3,ptena,dpm2,si:ch211-117n7.7 |
| GO:0006413 | translational initiation | -7.33 | 29 | eif2s1b,eif3ea,eif2s3,eif3s10,eif3m,eif3d,eif6,eif3g,abce1,eif4a2,eif3ha,eif4eb,eif3k,eif2ak1,eif2a,eif3ba,eif4ebp1,drg1,rps21,rwdd1,rpl22l1,ppan,bxdc2,ptges3a,rpf2,rrp7a,snrpd2,mrto4,naf1 |
| GO:0000154 | rRNA modification | -6.78 | 27 | nop2,ftsj3,gar1,fbl,nsun5,nop10,trmt112,bud23,emg1,nhp2,prmt1,suv39h1b,trmt2a,pcmtl,eef1akmt1,dph5,prmt3,kmt2ca,thumpd3,comtd1,ezh1,pmt,rrp8,shmt2,alkbh1,qng1,naf1 |
| GO:0000460 | maturation of 5.8S rRNA | -6.11 | 12 | pes,mak16,ftsj3,nsa2,gnl3,rrp15,rrs1,eif6,rps21,gnl2,tsr1,cdc14b |
| GO:0042274 | ribosomal small subunit biogenesis | -5.04 | 13 | npm1a,wdr43,snu13b,tsr1,utp15,rrs1,ngdn,utp6,pno1,wdr46,rps21,utp3,rrp7a |
| GO:0019216 | regulation of lipid metabolic process | -4.94 | 14 | ppardb,pik3ip1,ldlra,acaa2,pnpla3,ormdl3,slc27a1a,cpt1ab,thrsp,apoc1,tm6sf2b,srebf1,zgc:162608,pnpla2 |
| GO:0019637 | organophosphate metabolic process | -4.37 | 88 | nme2b.1,gart,papss2b,ATP6,ATP8,atic,paics,nt5c3a,inppl1a,gpd1b,cnp,lpcat4,gnpat2,impdh1b,kmo,pfkfb4b,aprt,pla2g12a,sucla2,hk2,cmpk,pi4k2a,rbks,acss2,aclya,mvda,slc27a1a,smpdl3a,abhd4,pcyt1aa,lpcat2,pik3r1,plpp3,acaca,npr1a,prps1b,dnph1,enpp1,nadkb,pdxkb,agpat9l,g6pd,pla2g1b,inpp5l,cish,rarres3,ptena,dpm2,pmvk,idh1,npr2,gpam,ugdh,ada2a,uox,gmppb,urad,shmt2,urod,ppardb,cebpb,rdh8a,esr2a,hnf4a,hsd17b12b,dhcr7,ssu72,pcbd1,pycr1a,nr1d2a,polr1f,ptges3a,msmo1,rxrgb,nr1h4,nop10,polr1d,polr1e,nr1h5,cyp27a3,qdpra,ncl,hsd17b7,irf1b,polr1g,nhp2,ttf1.2,si:ch211-117n7.7 |
| GO:1901615 | organic hydroxy compound metabolic process | -4.35 | 27 | rdh8a,adh8a,dio2,dhcr7,pck1,pcbd1,idh3a,msmo1,mvda,lratb.1,cyp46a1.1,apoea,amacr,rft1,pdxkb,dgat2,cyp27a3,qdpra,ebpl,hsd17b7,tkfc,dpm2,pmvk,cyp24a1,idh1,hsd17b12b,hsd17b14 |
| GO:0007005 | mitochondrion organization | -4.15 | 42 | mtx2,tomm40,tomm34,bnip3la,rab5if,dnaja3a,bnip3,higd1a,mcl1b,timm17a,ndufaf8,timm50,slc25a33,sqstm1,snx7,timm13,slc25a36a,tomm20b,cox17,sdhaf3,tomm5,ssbp1,cluha,oxa1l,ttc19,adck1,polg2,vdac2,micu2,slc25a22a,slc25a38b,sec61a1a,tram1,heatr3,kpna2,lamp2,srpra,kpnb3,kpnb1,srp14,sec61b,naca |
| GO:0061959 | response to (R)-carnitine | -4.13 | 4 | lpla,cpt1b,acaca,dgat2 |
| GO:0042742 | defense response to bacterium | -4.08 | 21 | tfa,cfb,pycard,mpeg1.1,hsp90b1,hamp,sqstm1,rac2,saa,dram1,pglyrp6,leap2,acod1,bpifcl,ly97.3,cebpb,hnf4a,ldlra,tlr3,cpt1b,tnip1 |
| GO:0043627 | response to estrogen | -3.88 | 8 | hsp90ab1,agxtb,esr2a,hpxa,ssr1,aqp12,lman1,nupr1b |
| GO:0046942 | carboxylic acid transport | -3.84 | 23 | fabp10a,slc26a5,pla2g12a,mttp,slc38a5b,mfsd2ab,fabp4a,cpt1b,slc7a3a,slc7a2,slc43a2b,slc27a1a,slc16a10,slc38a2,slc25a22a,abcb11b,pla2g1b,slc25a38b,slc16a7,slc3a2a,slc16a1a,slc22a6l,slco1e1 |
| GO:0055088 | lipid homeostasis | -3.73 | 11 | apobb.1,ldlra,mttp,pnpla3,acox1,ormdl3,abhd4,dgat2,tm6sf2b,zgc:162608,adck1 |
