# Supplementary material for: Single housing of juveniles accelerates early-stage growth but extends adult lifespan in African turquoise killifish
Source: Aging (Albany NY). 2024 Sep 16;16(18):12443–72. doi: 10.18632/aging.206111 (PMC11466477; doi:10.18632/aging.206111)
Supplement: Supplementary Tables 2-4 [file aging-16-206111-s003.pdf]

Supplementary Table 2. GO term analysis for DEGs (> 1.5-fold change, *padj* < 0.01) between group- and single-housed juveniles during stages *1-4* in males.

446 down-regulated genes (G*1-4* > S*2-4*, FC1.5, *padj* < 0.01)				
**Term**	**Description**	**LogP**	**Counts**	**Symbols**
GO:0007601	visual perception	-34.62	68	opn1lw1,opn1sw2,opn1sw1,prph2a,crybb1,gnat1,gnat2,opn1mw2,pdca,pde6ha,rom1a,pde6c,rom1b,cryba2b,crygn2,crygm5,crygn1,crygm3,crygs1,cryba1l1,rgra,crygs3,cryba4,crybb2,crybb3,crybb1l1,crybgx,crygm2b,crygm6,crygmxl1,crygmxl2,crygm2f,prph2b,grk1a,crygm2e,unc119b,arr3b,cryaa,slc17a6b,penka,lhfpl3,ppef2a,scn8aa,glrbb,slc6a9,gabrg2,sv2a,gabra1,glra2,vip,plaat1,mipa,adcyap1b,rbp4l,ppm1na,cdk5r1b,cdhr1a,cdk5r2a,ankrd33aa,igf2bp1,cadm1a,lbhl,impdh1a,rab11fip4a,cd74b,aldh1l1,si:dkey-57a22.15,si:ch73-167i17.6
GO:0099536	synaptic signaling	-7.53	43	snap25a,dlg1a,gria2b,penka,gria3b,syt1a,glrbb,slc6a9,gabrg2,cplx2b,dtnbb,syt2a,sv2a,slc12a5a,syn2b,grin1a,cplx4a,gabra1,glra2,slc17a6b,chata,ptprna,ptprn2,gabbr1a,gje1a,vip,scn8aa,slco1d1,slc5a1,slc13a2,slc6a1b,atp2b2,atp2b3b,slc39a4,scn3b,kcnma1a,atp13a2,slc4a7,slc6a1a,slc4a10a,lrrc38b,si:ch211-117c9.5,si:ch211-251b21.1
GO:0006836	neurotransmitter transport	-6.47	28	snap25a,syt1a,slc6a1b,slc6a9,cplx2b,syt2a,sv2a,syn2b,slc6a1a,cplx4a,slc17a6b,ptprna,ptprn2,vip,scamp5a,scg5,chga,stx1b,dnm2a,scn8aa,slc1a2b,pacsin1a,flot1a,scn3b,kcnma1a,unc119b,dpp6a,adcyap1b
GO:0009583	detection of light stimulus	-4.97	19	opn1lw1,opn1sw2,opn1sw1,gnat1,gnat2,opn1mw2,rgra,rcvrn2,grk1b,scn8aa,ucp1,igfbp1a,ddit4,kcnma1a,cryaa,si:dkey-1k23.3,cadm1a,ppef2a,si:ch73-167i17.6
GO:0006030	chitin metabolic process	-4.24	3	chs1,chia.2,chia.3
GO:0071248	cellular response to metal ion	-4.11	9	slc13a2,syt1a,cpne4a,syt2a,cpne5a,atp13a2,tap2a,slc1a2b,chga
GO:1990778	protein localization to cell periphery	-3.90	10	stx1b,prph2a,dlg1a,rom1a,rom1b,ank2b,prph2b,flot1a,arl13a,sgtb
GO:0008277	regulation of G protein-coupled receptor signaling pathway	-3.81	6	pdca,pde6ha,grk1a,rgs9a,saga,arr3b
GO:0019882	antigen processing and presentation	-3.73	8	cd74b,mhc2dgb,mhc2d8.46a,b2ml,marchf1,flot1a,zgc:123297,lgals9l3
GO:0007626	locomotory behavior	-3.49	13	snap25a,scn8aa,slc1a2b,camk1gb,slc6a9,sv2a,atp13a2,thnsl2,gabra1,chata,serpini1,bmerb1,vip
GO:0010817	regulation of hormone levels	-3.41	9	ptprna,ptprn2,dio2,scg5,chga,lratb.2,vip,pcsk2,slco1d1
GO:0046394	carboxylic acid biosynthetic process	-3.22	12	agxtb,alox5ap,gad1b,elovl5,got1,agxta,asah2,gad2,abhd2b,thnsl2,hpda,prodha
GO:0015849	organic acid transport	-3.16	16	fabp2,slco1d1,slc1a2b,slc13a2,slc6a1b,slc6a9,slc3a1,slc6a1a,slc7a5,slc17a6b,si:ch211-117c9.5,slc16a1a,scn8aa,slc5a1,scn3b,slc2a1a
GO:0007254	JNK cascade	-3.06	3	mapk8ip2,si:ch211-45c16.2,mapk10
GO:0035970	peptidyl-threonine dephosphorylation	-3.06	3	ppm1na,ppm1kb,ppm1nb
GO:0050908	detection of light stimulus involved in visual perception	-2.94	3	gnat1,gnat2,si:ch73-167i17.6
GO:0060078	regulation of postsynaptic membrane potential	-2.87	15	slc6a9,gabrg2,gabra1,glra2,clstn3,glrbb,slc4a7,slc12a5a,slc4a10a,slc17a6b,gabbr1a,scn8aa,scn3b,sv2a,grin1a
GO:0002455	humoral immune response mediated by circulating immunoglobulin	-2.73	8	cfb,c9,si:ch1073-280e3.1,cd74b,mhc2dgb,cadm1a,mhc2d8.46a,b2ml
GO:0035249	synaptic transmission, glutamatergic	-2.45	4	gria2b,gria3b,si:ch211-251b21.1,slc17a6b
GO:1901890	positive regulation of cell junction assembly	-2.27	3	clstn3,flot1a,lrrc4bb

117 up-regulated genes (G*1-4* < S*2-4*, FC1.5, *padj* < 0.01)				
**Term**	**Description**	**LogP**	**Counts**	**Symbols**
GO:0030198	extracellular matrix organization	-10.73	15	serpinh1b,col1a2,col1a1a,loxa,lum,col11a1b,col10a1a,col5a2a,p3h4,adamts12,col5a3a,col16a1,hsp90aa1.1,ttn.2,igfn1.4
GO:0061061	muscle structure development	-5.92	11	myog,hsp90aa1.1,chrna1,ttn.2,loxa,mymk,pygma,synpo2la,pgam2,myl2b,igfn1.4
GO:0006457	protein folding	-5.24	7	hsp90aa1.1,fkbp10b,hsc70,fkbp9,ppil1,ptges3l,hspb9
GO:0006090	pyruvate metabolic process	-3.93	6	ldha,bpgm,pgam2,gck,pygma,gatm
GO:0031099	regeneration	-2.69	7	myog,serpinh1b,col1a1a,col10a1a,gck,chrna1,dio2

Supplementary Table 3. GO term analysis for DEGs (> 1.5-fold change, *padj* < 0.01) between group- and single-housed juveniles during stages *1-4* in females.

Term	Description	LogP	Counts	Symbols
**276 down-regulated genes (G*1-4* > S2-*4*, FC1.5, *padj* < 0.01)**				
**Term**	**Description**	**LogP**	**Counts**	**Symbols**
GO:0007601	visual perception	-27.07	47	opn1lw1,opn1sw2,opn1sw1,prph2a,crybb1,gnat1,gnat2,pde6ha,rom1a,cryba2b,crygn2,crygm5,crygn1,crygm3,crygs1,cryba1l1,crygs3,cryba4,crybb3,crybb1l1,crybgx,crygm2b,crygm6,crygmxl1,si:ch73-167i17.6,grk1a,crygm2e,si:dkey-57a22.15,slc17a6b,scn8aa,glrbb,slc6a9,gabrg2,sv2a,gabra1,adcyap1b,ppm1na,cdhr1a,cdk5r2a,ankrd33aa,igf2bp1,cadm1a,ndr1,lbhl,pax10,impdh1a,rab11fip4a
GO:0048477	oogenesis	-5.47	10	zp3b,buc,zar1,zp3a.1,zp3a.2,zar1l,zglp1,fkbp6,dio2,cadm1a
GO:0006836	neurotransmitter transport	-4.59	15	snap25a,syt1a,slc6a9,syt2a,sv2a,syn2b,slc17a6b,gria2b,glrbb,gabrg2,slc12a5a,gabra1,ptprna,ptprn2,scn8aa
GO:0009583	detection of light stimulus	-4.50	11	opn1lw1,opn1sw2,opn1sw1,gnat1,gnat2,rcvrn2,scn8aa,ucp1,ucp3,cadm1a,si:ch73-167i17.6
GO:0008345	larval locomotory behavior	-4.33	8	scn8aa,slc1a2b,thnsl2,gabra1,snap25a,camk1gb,slc6a9,sv2a
GO:0032940	secretion by cell	-4.08	10	snap25a,stx1b,scamp5a,ptprna,ptprn2,syt1a,syt2a,syn2b,ndr1,slc1a2b
GO:0001678	intracellular glucose homeostasis	-3.77	4	ptprna,ptprn2,hk2,ndr1
GO:0099003	vesicle-mediated transport in synapse	-3.37	7	snap25a,syt1a,dnm2a,si:ch211-200p22.4,syt2a,slc17a6b,kif5aa
GO:0031175	neuron projection development	-2.75	15	elavl4,scn8aa,ncam1b,adcyap1b,gpm6aa,nrn1a,stmn4l,b3gat1a,cdk5r2a,pacsin1a,si:ch211-113g11.6,kif5aa,si:ch211-195b13.1,map4l,ndrg4
GO:1990778	protein localization to cell periphery	-2.33	5	stx1b,prph2a,rom1a,ank2b,arl13a
GO:0003333	amino acid transmembrane transport	-2.16	4	slc1a2b,slc6a9,slc7a5,slc17a6b
**115 up-regulated genes (G*1-4* < S2-*4*, FC1.5, *padj* < 0.01)**				
**Term**	**Description**	**LogP**	**Counts**	**Symbols**
GO:0030198	extracellular matrix organization	-13.47	17	serpinh1b,col1a2,col1a1a,postnb,postna,loxa,lum,zgc:113232,dpt,col10a1a,p3h4,adamts12,col5a3a,col16a1,ttn.2,tnnt2e,igfn1.4
GO:0006090	pyruvate metabolic process	-6.76	14	ldha,gapdh,aldoaa,bpgm,pgam2,gck,pygma,gpd1b,ppp1r3ca,slc25a55b,gbgt1l1,gatm,tecra,rfesd
GO:0061061	muscle structure development	-4.28	10	myog,ttn.2,postnb,loxa,pygma,tnnt2e,pgam2,myl2b,igfn1.4,tnni4b.2
GO:0071391	cellular response to estrogen stimulus	-3.46	3	hpxa,vtg6,vtg2
GO:0031099	regeneration	-2.71	5	myog,serpinh1b,col1a1a,col10a1a,gck
GO:0007517	muscle organ development	-2.71	5	myog,ttn.2,postnb,loxa,pygma

Supplementary Table 4. Results of statistical analysis.

		Significance probability
**Figure**	**Data**	**Shapiro-Wilk-test**
Figure 4B	Group-housed fish	0.346
	Single-housed fish	0.190
Figure 5B_Testis	Group-housed fish_Stage II	0.404
	Group-housed fish_Stage III	0.249
	Group-housed fish_Stage IV	0.837
	Single-housed fish_Stage II	0.656
	Single-housed fish_Stage III	0.767
	Single-housed fish_Stage IV	0.210
Figure 5B_Ovary	Group-housed fish_Stage I	0.323
	Group-housed fish_Stage II	0.976
	Group-housed fish_Stage III	0.976
	Group-housed fish_Stage IV	0.915
	Single-housed fish_Stage I	0.415
	Single-housed fish_Stage II	0.931
	Single-housed fish_Stage III	0.034
	Single-housed fish_Stage IV	0.754

		Significance probability			
**Figure**	**Comparison**	**Kruskal-Wallis test**	**Levene-test**	**t-test (homogeneous variance)**	**Welch-test (unequal variance)**
Figure 4B	Group-housed fish vs Single-housed fish	-	0.970	0.035	-
Figure 5B_Testis	Stage I_Group-housed fish vs Single-housed fish	-	0.850	0.289	-
	Stage II_Group-housed fish vs Single-housed fish	-	0.845	0.011	-
	Stage III_Group-housed fish vs Single-housed fish	-	0.605	0.077	-
	Stage IV_Group-housed fish vs Single-housed fish	-	0.233	0.340	-
Figure 5B_Ovary	Stage I_Group-housed fish vs Single-housed fish	-	0.025	-	0.168
	Stage II_Group-housed fish vs Single-housed fish	-	0.213	0.886	-
	Stage III_Group-housed fish vs Single-housed fish	0.014	-	-	-
	Stage IV_Group-housed fish vs Single-housed fish	-	0.123	0.109	-
