# Supplementary material for: Single housing of juveniles accelerates early-stage growth but extends adult lifespan in African turquoise killifish
Source: Aging (Albany NY). 2024 Sep 16;16(18):12443–72. doi: 10.18632/aging.206111 (PMC11466477; doi:10.18632/aging.206111)
Supplement: Supplementary Material 1 [file aging-16-206111-s004.docx]

**Supplementary Material 1. Script for gonads analysis (Figure 5).**

**(A) Linux commands**

#0 Analysis environment

Ubuntu 18.04.6 LTS

#1. Making directories

workDir=/mnt/d/takahashi/Gonads

mkdir $workDir

scDir=/mnt/d/takahashi/Gonads/00_reference

mkdir $scDir&& cd $_

#Copying sample information file to 00_reference

cp /mnt/d/takahashi/sample.csv $_

#Making directories from the information of the sample file

cat sample.csv | awk -F "," '{print $2}'  > filename.txt

cat sample.csv | awk -F "," '{print $3}'  > workDir.txt

paste -d "/" workDir.txt filename.txt > FilenameDir.txt

cat FilenameDir.txt | {

while read dir1

  do

      mkdir -p $dir1 && cd $_

      mkdir 01_raw-seq 02_fastqc 03_valid-seq 04_abundant-check 05_mapping 06_count 07_clustering 08_DESeq2

  done

}

cd $workDir

tree

#2. Making index for HISAT2

cd $scDir

wget ftp://ftp.ensembl.org/pub/release-105/fasta/nothobranchius_furzeri/dna/Nothobranchius_furzeri.Nfu_20140520.dna.toplevel.fa.gz

gzip -d Nothobranchius_furzeri.Nfu_20140520.dna.toplevel.fa.gz

hisat2-build Nothobranchius_furzeri.Nfu_20140520.dna.toplevel.fa Nothobranchius_furzeri.Nfu_20140520.dna.toplevel

cd $scDir

hisat2-build nfu.abundant.fa nfu.abundant

#3-1. Download of Genome Data from Ensembl

cd $scDir

wget ftp://ftp.ensembl.org/pub/release-105/gtf/nothobranchius_furzeri/Nothobranchius_furzeri.Nfu_20140520.105.gtf.gz

gzip -d Nothobranchius_furzeri.Nfu_20140520.105.gtf.gz

#3-2. Making homologues list

cd /mnt/d/takahashi

mkdir blast

cd /mnt/d/takahashi/blast

# downloading blast souce file and compiling

wget ftp://ftp.ncbi.nih.gov/blast/executables/blast+/LATEST/ncbi-blast-2.13.0+-src.tar.gz

tar xzvf ncbi-blast-2.13.0+-src.tar.gz

cd ncbi-blast-2.13.0+-src

cd c++

./configure

make

cd /mnt/d/takahashi/blast

#downloading of Zebrafish genome

wget ftp://ftp.ensembl.org/pub/release-108/fasta/danio_rerio/pep/Danio_rerio.GRCz11.pep.all.fa.gz

gunzip Danio_rerio.GRCz11.pep.all.fa.gz

#downloading of Nothobranchius genome

wget ftp://ftp.ensembl.org/pub/release-105/fasta/nothobranchius_furzeri/pep/Nothobranchius_furzeri.Nfu_20140520.pep.all.fa.gz

gunzip Nothobranchius_furzeri.Nfu_20140520.pep.all.fa.gz

#downloading of medaka genome

wget ftp://ftp.ensembl.org/pub/release-108/fasta/oryzias_latipes/pep/Oryzias_latipes.ASM223467v1.pep.all.fa.gz

gunzip Oryzias_latipes.ASM223467v1.pep.all.fa.gz

#making database with makeblastdb

makeblastdb -in Danio_rerio.GRCz11.pep.all.fa -dbtype prot -out GRCz11_ensembl

makeblastdb -in Nothobranchius_furzeri.Nfu_20140520.pep.all.fa -dbtype prot -out Nfu_20140520_ensembl

makeblastdb -in Oryzias_latipes.ASM223467v1.pep.all.fa -dbtype prot -out medaka_ensembl

blastp -query Nothobranchius_furzeri.Nfu_20140520.pep.all.fa -db GRCz11_ensembl -max_target_seqs 1 -outfmt "6 qseqid sseqid pident length mismatch gapopen qstart qend sstart send evalue bitscore stitle" -evalue 1e-10 -num_threads 8 > nfu_zeb_result.txt

blastp -query Nothobranchius_furzeri.Nfu_20140520.pep.all.fa -db Nfu_20140520_ensembl -max_target_seqs 1 -outfmt "6 qseqid sseqid pident length mismatch gapopen qstart qend sstart send evalue bitscore stitle" -evalue 1e-10 -num_threads 8 > nfu_nfu_result.txt

blastp -query Nothobranchius_furzeri.Nfu_20140520.pep.all.fa -db medaka_ensembl -max_target_seqs 1 -outfmt "6 qseqid sseqid pident length mismatch gapopen qstart qend sstart send evalue bitscore stitle" -evalue 1e-10 -num_threads 8 > nfu_medaka_result.txt

#making gene_symbol.txt

cat nfu_zeb_result.txt | awk -F "gene_symbol:" '{print $2}' | awk -F " " '{print $1}' > zeb_gene_symbol.txt

cat nfu_nfu_result.txt | awk -F "gene_symbol:" '{print $2}' | awk -F " " '{print $1}' > nfu_gene_symbol.txt

cat nfu_medaka_result.txt | awk -F "gene_symbol:" '{print $2}' | awk -F " " '{print $1}' > medaka_gene_symbol.txt

#making description.txt

cat nfu_zeb_result.txt | awk -F "description:" '{print $2}' | awk -F "Source" '{print $1}' | sed -e 's/ \[//g' > zeb_description.txt

cat nfu_nfu_result.txt | awk -F "description:" '{print $2}' | awk -F "Source" '{print $1}' | sed -e 's/ \[//g' > nfu_description.txt

cat nfu_medaka_result.txt | awk -F "description:" '{print $2}' | awk -F "Source" '{print $1}' | sed -e 's/ \[//g' > medaka_description.txt

#making geneID-matched-proteinID

#making geneID.txt

cat nfu_zeb_result.txt | awk -F "gene:" '{print $2}' | awk -F " " '{print $1}' > zeb_gene_ID.txt

cat nfu_nfu_result.txt | awk -F "gene:" '{print $2}' | awk -F " " '{print $1}' > nfu_gene_ID.txt

cat nfu_medaka_result.txt | awk -F "gene:" '{print $2}' | awk -F " " '{print $1}' > medaka_gene_ID.txt

#removing ver information

cat nfu_zeb_result.txt | awk -F "gene:" '{print $2}' | awk -F " " '{print $1}' | awk -F "." '{print $1}' > zeb_gene_ID_noVer.txt

cat nfu_nfu_result.txt | awk -F "gene:" '{print $2}' | awk -F " " '{print $1}' | awk -F "." '{print $1}' > nfu_gene_ID_noVer.txt

cat nfu_medaka_result.txt | awk -F "gene:" '{print $2}' | awk -F " " '{print $1}' | awk -F "." '{print $1}' > medaka_gene_ID_noVer.txt

cat nfu_zeb_result.txt | awk '{print $2}' | awk -F "." '{print $1}'> zeb_protein_ID_noVer.txt

cat nfu_nfu_result.txt | awk '{print $2}' | awk -F "." '{print $1}'> nfu_protein_ID_noVer.txt

cat nfu_medaka_result.txt | awk '{print $2}' | awk -F "." '{print $1}'> medaka_protein_ID_noVer.txt

#completing of geneID-proteinID

paste -d "\t" zeb_gene_ID_noVer.txt  zeb_protein_ID_noVer.txt > zeb_geneID_protienID.txt

paste -d "\t" nfu_gene_ID_noVer.txt  nfu_protein_ID_noVer.txt > nfu_geneID_protienID.txt

paste -d "\t" medaka_gene_ID_noVer.txt  nfu_protein_ID_noVer.txt > medaka_geneID_protienID.txt

paste -d "\t" nfu_zeb_result.txt  zeb_gene_symbol.txt zeb_description.txt zeb_gene_ID_noVer.txt > nfu_zeb_BlastResultFinal2.txt

paste -d "\t" nfu_nfu_result.txt  nfu_gene_symbol.txt nfu_description.txt nfu_gene_ID_noVer.txt > nfu_nfu_BlastResultFinal2.txt

paste -d "\t" nfu_medaka_result.txt  medaka_gene_symbol.txt medaka_description.txt medaka_gene_ID_noVer.txt > nfu_medaka_BlastResultFinal2.txt

nn=/mnt/d/takahashi/blast/nfu_nfu_BlastResultFinal2.txt

nz=/mnt/d/takahashi/blast/nfu_zeb_BlastResultFinal2.txt

nm=/mnt/d/takahashi/blast/nfu_medaka_BlastResultFinal2.txt

wdir=/mnt/d/takahashi/blast/

R --no-save --args $nn $nz $nm $wdir < /mnt/d/takahashi/blast/blast4.R

#4. check of raw data in 01_rawdata

workDir=/mnt/d/takahashi/Gondads

scDir=/mnt/d/takahashi/Gonads/00_reference

cd $scDir

cat FilenameDir.txt | {

while read dir1

  do

    cd $dir1/01_raw-seq

    for d in `find ./ -type d`;

        do echo $dir1,`ls "$d" | wc -l`;

    done

    md5sum Lib*gz > sum.txt

  done

}

#5. RUN FastQC

workDir=/mnt/d/takahashi/Gonads

scDir=/mnt/d/takahashi/Gonads/00_reference

cd $scDir

cat FilenameDir.txt | {

  while read dir1

  do

      fastqc -t 8 --nogroup $dir1/01_raw-seq/Lib*.gz -o $dir1/02_fastqc

      multiqc $dir1/02_fastqc

      mv ./multiqc_report*.html $dir1/02_fastqc/

  done

}

#6. Processing  sequence data with Trim galore

workDir=/mnt/d/takahashi/Gonads

scDir=/mnt/d/takahashi/Gonads/00_reference

cd $scDir

cat FilenameDir.txt | {

  while read dir1

  do

    MAX=$((`find $dir1/01_raw-seq -type f | wc -l`/2))

    for i in `seq -f "%02g" $MAX`

    do

    trim_galore -q 30 --length 35 \

    --paired $dir1/01_raw-seq/Lib${i}*_1.fastq.gz $dir1/01_raw-seq/Lib${i}*_2.fastq.gz \

    -o $dir1/03_valid-seq \

    &> $dir1/03_valid-seq/fastq${i}.trim-galore.log;

    done

  done

}

#7 genome mapping with HISAT2

workDir=/mnt/d/takahashi/Gonads

scDir=/mnt/d/takahashi/Gonads/00_reference

cd $scDir

cat FilenameDir.txt | {

  while read dir1

  do

  MAX=$((`find $dir1/01_raw-seq -type f | wc -l`/2))

  for i in `seq -f "%02g" $MAX`

  do

    hisat2 -p 8 -x $scDir/nfu.abundant \

      -1 $dir1/03_valid-seq/Lib${i}_*val_1.fq.gz \

      -2 $dir1/03_valid-seq/Lib${i}_*val_2.fq.gz \

      -S $dir1/04_abundant-check/Lib${i}.abundant.sam \

      --no-unal --no-hd \

      2>$dir1/04_abundant-check/Lib${i}.abundant.log;

  done

  done

}

workDir=/mnt/d/takahashi/Gonads

scDir=/mnt/d/takahashi/Gonads/00_reference

cd $scDir

cat FilenameDir.txt | {

  while read dir1

  do

    echo -e "library\tchrM\teGFP\tphiX174\trRNA\tmRNA" > $dir1/04_abundant-check/abundant.summary.log;

    MAX=$((`find $dir1/01_raw-seq -type f | wc -l`/2))

    echo $MAX;

  done

}

cat FilenameDir.txt | {

  while read dir1

  do

    MAX=$((`find $dir1/01_raw-seq -type f | wc -l`/2))

    for i in `seq -f "%02g" $MAX`

    do

    paste <(echo $dir1/04_abundant-check/Lib${i}) \

    <(grep -c chrM    $dir1/04_abundant-check/Lib${i}.abundant.sam) \

    <(grep -c eGFP    $dir1/04_abundant-check/Lib${i}.abundant.sam) \

    <(grep -c phiX174 $dir1/04_abundant-check/Lib${i}.abundant.sam) \

    <(grep -c EU780557    $dir1/04_abundant-check/Lib${i}.abundant.sam) \

    <(sed -n 3P $dir1/04_abundant-check/Lib${i}.abundant.log | cut -f 5 -d ' ') \

    >> $dir1/04_abundant-check/abundant.summary.log;

    done

  done

}

cat FilenameDir.txt | {

  while read dir1

  do

    R --no-save --args $dir1/04_abundant-check/abundant.summary.log $dir1/04_abundant-check/abundant < $scDir/logPlot.R

  done

}

workDir=/mnt/d/takahashi/Gonads

scDir=/mnt/d/takahashi/Gonads/00_reference

cd $scDir

cat FilenameDir.txt | {

  while read dir1

  do

    MAX=$((`find $dir1/01_raw-seq -type f | wc -l`/2))

    for i in `seq -f "%02g" $MAX`

    do

    hisat2 -p 8 -x $scDir/Nothobranchius_furzeri.Nfu_20140520.dna.toplevel --rna-strandness RF \

    -1 $dir1/03_valid-seq/Lib${i}*_1_val_1.fq.gz -2 $dir1/03_valid-seq/Lib${i}*_2_val_2.fq.gz \

    --dta \

    -S $dir1/05_mapping/Lib${i}.sam 2> $dir1/05_mapping/Lib${i}.mapping.log;

    done

  done

}

workDir=/mnt/d/takahashi/Gonads

scDir=/mnt/d/takahashi/Gonads/00_reference

cd $scDir

cat FilenameDir.txt | {

  while read dir1

  do

    MAX=$((`find $dir1/01_raw-seq -type f | wc -l`/2))

    for i in `seq -f "%02g" $MAX`

    do

    samtools sort -@ 8 $dir1/05_mapping/Lib${i}.sam > $dir1/05_mapping/Lib${i}.bam;

    samtools index -@ 8 $dir1/05_mapping/Lib${i}.bam;

    done

  done

}

workDir=/mnt/d/takahashi/Gonads

scDir=/mnt/d/takahashi/Gonads/00_reference

cd $scDir

cat FilenameDir.txt | {

  while read dir1

  do

    echo -e "library\tunmapped\tmulti-mapped\tuniq-mapped" > $dir1/05_mapping/mapping.summary.log;

    MAX=$((`find $dir1/01_raw-seq -type f | wc -l`/2))

    for i in `seq -f "%02g" $MAX`

    do

        paste <(echo $dir1/05_mapping/Lib${i}) \

        <(sed -n 3P $dir1/05_mapping/Lib${i}.mapping.log | cut -f 5 -d ' ') \

        <(sed -n 5P $dir1/05_mapping/Lib${i}.mapping.log | cut -f 5 -d ' ') \

        <(sed -n 4P $dir1/05_mapping/Lib${i}.mapping.log | cut -f 5 -d ' ') \

        >> $dir1/05_mapping/mapping.summary.log;

    done

    R --no-save --args $dir1/05_mapping/mapping.summary.log $dir1/05_mapping/mapping <  $scDir/logPlot.R #H-1 script

    done

}

#9.Counting with fetureCounts

workDir=/mnt/d/takahashi/Gonads

scDir=/mnt/d/takahashi/Gonads/00_reference

cd $scDir

cat FilenameDir.txt | {

    while read dir1

    do

    featureCounts -T 8 -p -B -C -t exon -g gene_id -a $scDir/Nothobranchius_furzeri.Nfu_20140520.105.gtf -o $dir1/06_count/all.featurecounts.txt $dir1/05_mapping/Lib*.bam;

  done

}

#Manually delete the first line of the text file "all.featurecounts.txt",  and rename it to "all.featurecounts2.txt".

workDir=/mnt/d/takahashi/Gonads

scDir=/mnt/d/takahashi/Gonads/00_reference

cd $scDir

cat FilenameDir.txt | {

    while read dir1

    do

    R --no-save --args $dir1/06_count/all.featurecounts2.txt $dir1/06_count/ $dir1/06_count/colnames.txt <  $scDir/counts_to_tpm2.R #H-2 script

    R --no-save --args $dir1/06_count/TPMfeaturecounts.txt $dir1/06_count $dir1/06_count/colnames.txt<  $scDir/TH.R #H-3 script

    R --no-save --args /mnt/d/takahashi/blast/ncbi-blast-2.13.0+-src/c++/nfu_zeb_medaka.txt $dir1/06_count/TPMfeaturecounts.txt $dir1/06_count/ <  /mnt/d/takahashi/blast/ncbi-blast-2.13.0+-src/c++/blast3.R #H-12 script

  done

}

#10. clustring (For Fig.5A)

workDir=/mnt/d/takahashi/Gonads/testis

cd $workDir

R --no-save --args $workDir/06_count/TPMfeaturecounts_max10.txt $workDir/07_clustering < $workDir/07_clustering/Fig5A-testis.R #H-4 script

workDir=/mnt/d/takahashi/Gonads/ovary

cd $workDir

R --no-save --args $workDir/06_count/TPMfeaturecounts_max10.txt $workDir/07_clustering < $workDir/07_clustering/Fig5A-ovary.R #H-5 script

#11. PCA & DEseq2 (For Fig.5B)

#Manually delete ' of the text file "newTPMfeaturecounts.txt" in Excel (microsoft),  and rename it to "newTPMfeaturecounts2.txt".

workDir=/mnt/d/takahashi/Gonads

scDir=/mnt/d/takahashi/Gonads/00_reference

wdir=$workDir/testis/08_DESeq2/

data=$workDir/testis/06_count/all.featurecounts2.txt

INDEX=$workDir/testis/08_DESeq2/samplenames.txt

GTF=$scDir/Nothobranchius_furzeri.Nfu_20140520.105.gtf

default=default

TPMdata=$workDir/testis/06_count/TPMfeaturecounts_max10.txt

newTPMdata=$workDir/testis/06_count/newTPMfeaturecounts2.txt

THtpm=10

ID=/mnt/d/takahashi/blast/ncbi-blast-2.13.0+-src/c++/nfu_zeb_medaka.txt

R --no-save --args $wdir $data $INDEX $GTF defalt $TPMdata $newTPMdata $THtpm $ID< $workDir/testis/08_DESeq2/DESeq2_M.R #H-6 script

workDir=/mnt/d/takahashi/Gonads

scDir=/mnt/d/takahashi/Gonads/00_reference

wdir=$workDir/ovary/08_DESeq2

data=$workDir/ovary/06_count/all.featurecounts2.txt

INDEX=$workDir/ovary/08_DESeq2/samplenames.txt

GTF=$scDir/Nothobranchius_furzeri.Nfu_20140520.105.gtf

default=default

TPMdata=$workDir/ovary/06_count/TPMfeaturecounts_max10.txt

newTPMdata=$workDir/ovary/06_count/newTPMfeaturecounts2.txt

THtpm=10

ID=/mnt/d/takahashi/blast/ncbi-blast-2.13.0+-src/c++/nfu_zeb_medaka.txt

R --no-save --args $wdir $data $INDEX $GTF defalt $TPMdata $newTPMdata $THtpm $ID< $workDir/ovary/08_DESeq2/DESeq2_F.R #H-7 script

#11. Heatmap of gene expression change (Fig.5C)

workDir=/mnt/d/takahashi/Gonads/testis/06_count

cd $workDir

R --no-save --args ./spermatogenesis.csv spermatogenesis-TPM.txt ./newTPMfeaturecounts2.txt ./ <  ./ID-TPMv2.R #H-8 script

R --no-save --args ./spermatogenesis-TPM.txt ./ < ./Fig5C-testis.R #H-9 script

workDir=/mnt/d/takahashi/Gonads/ovary/06_count

cd $workDir

R --no-save --args ./ovaryGOterms-nfu.txt ovaryGOterms-nfu-TPM.txt ./newTPMfeaturecounts2.txt ./ <  ./ID-TPMv3.R #H-10 script

R --no-save --args ./ovaryGOterms-nfu-TPM.txt ./ < ./Fig5C-ovary.R #H-11 script

**(B) sample.csv**

1,testis,/mnt/d/takahashi/Gonads,

2,ovary,/mnt/d/takahashi/Gonads,

**(C) nfu.abundant.fa**

>phiX174

GAGTTTTATCGCTTCCATGACGCAGAAGTTAACACTTTCGGATATTTCTGATGAGTCGAAAAATTATCTTGATAAAGCAGGAATTACTACTGCTTGTTTACGAATTAAATCGAAGTGGACTGCTGGCGGAAAATGAGAAAATTCGACCTATCCTTGCGCAGCTCGAGAAGCTCTTACTTTGCGACCTTTCGCCATCAACTAACGATTCTGTCAAAAACTGACGCGTTGGATGAGGAGAAGTGGCTTAATATGCTTGGCACGTTCGTCAAGGACTGGTTTAGATATGAGTCACATTTTGTTCATGGTAGAGATTCTCTTGTTGACATTTTAAAAGAGCGTGGATTACTATCTGAGTCCGATGCTGTTCAACCACTAATAGGTAAGAAATCATGAGTCAAGTTACTGAACAATCCGTACGTTTCCAGACCGCTTTGGCCTCTATTAAGCTCATTCAGGCTTCTGCCGTTTTGGATTTAACCGAAGATGATTTCGATTTTCTGACGAGTAACAAAGTTTGGATTGCTACTGACCGCTCTCGTGCTCGTCGCTGCGTTGAGGCTTGCGTTTATGGTACGCTGGACTTTGTGGGATACCCTCGCTTTCCTGCTCCTGTTGAGTTTATTGCTGCCGTCATTGCTTATTATGTTCATCCCGTCAACATTCAAACGGCCTGTCTCATCATGGAAGGCGCTGAATTTACGGAAAACATTATTAATGGCGTCGAGCGTCCGGTTAAAGCCGCTGAATTGTTCGCGTTTACCTTGCGTGTACGCGCAGGAAACACTGACGTTCTTACTGACGCAGAAGAAAACGTGCGTCAAAAATTACGTGCGGAAGGAGTGATGTAATGTCTAAAGGTAAAAAACGTTCTGGCGCTCGCCCTGGTCGTCCGCAGCCGTTGCGAGGTACTAAAGGCAAGCGTAAAGGCGCTCGTCTTTGGTATGTAGGTGGTCAACAATTTTAATTGCAGGGGCTTCGGCCCCTTACTTGAGGATAAATTATGTCTAATATTCAAACTGGCGCCGAGCGTATGCCGCATGACCTTTCCCATCTTGGCTTCCTTGCTGGTCAGATTGGTCGTCTTATTACCATTTCAACTACTCCGGTTATCGCTGGCGACTCCTTCGAGATGGACGCCGTTGGCGCTCTCCGTCTTTCTCCATTGCGTCGTGGCCTTGCTATTGACTCTACTGTAGACATTTTTACTTTTTATGTCCCTCATCGTCACGTTTATGGTGAACAGTGGATTAAGTTCATGAAGGATGGTGTTAATGCCACTCCTCTCCCGACTGTTAACACTACTGGTTATATTGACCATGCCGCTTTTCTTGGCACGATTAACCCTGATACCAATAAAATCCCTAAGCATTTGTTTCAGGGTTATTTGAATATCTATAACAACTATTTTAAAGCGCCGTGGATGCCTGACCGTACCGAGGCTAACCCTAATGAGCTTAATCAAGATGATGCTCGTTATGGTTTCCGTTGCTGCCATCTCAAAAACATTTGGACTGCTCCGCTTCCTCCTGAGACTGAGCTTTCTCGCCAAATGACGACTTCTACCACATCTATTGACATTATGGGTCTGCAAGCTGCTTATGCTAATTTGCATACTGACCAAGAACGTGATTACTTCATGCAGCGTTACCATGATGTTATTTCTTCATTTGGAGGTAAAACCTCTTATGACGCTGACAACCGTCCTTTACTTGTCATGCGCTCTAATCTCTGGGCATCTGGCTATGATGTTGATGGAACTGACCAAACGTCGTTAGGCCAGTTTTCTGGTCGTGTTCAACAGACCTATAAACATTCTGTGCCGCGTTTCTTTGTTCCTGAGCATGGCACTATGTTTACTCTTGCGCTTGTTCGTTTTCCGCCTACTGCGACTAAAGAGATTCAGTACCTTAACGCTAAAGGTGCTTTGACTTATACCGATATTGCTGGCGACCCTGTTTTGTATGGCAACTTGCCGCCGCGTGAAATTTCTATGAAGGATGTTTTCCGTTCTGGTGATTCGTCTAAGAAGTTTAAGATTGCTGAGGGTCAGTGGTATCGTTATGCGCCTTCGTATGTTTCTCCTGCTTATCACCTTCTTGAAGGCTTCCCATTCATTCAGGAACCGCCTTCTGGTGATTTGCAAGAACGCGTACTTATTCGCCACCATGATTATGACCAGTGTTTCCAGTCCGTTCAGTTGTTGCAGTGGAATAGTCAGGTTAAATTTAATGTGACCGTTTATCGCAATCTGCCGACCACTCGCGATTCAATCATGACTTCGTGATAAAAGATTGAGTGTGAGGTTATAACGCCGAAGCGGTAAAAATTTTAATTTTTGCCGCTGAGGGGTTGACCAAGCGAAGCGCGGTAGGTTTTCTGCTTAGGAGTTTAATCATGTTTCAGACTTTTATTTCTCGCCATAATTCAAACTTTTTTTCTGATAAGCTGGTTCTCACTTCTGTTACTCCAGCTTCTTCGGCACCTGTTTTACAGACACCTAAAGCTACATCGTCAACGTTATATTTTGATAGTTTGACGGTTAATGCTGGTAATGGTGGTTTTCTTCATTGCATTCAGATGGATACATCTGTCAACGCCGCTAATCAGGTTGTTTCTGTTGGTGCTGATATTGCTTTTGATGCCGACCCTAAATTTTTTGCCTGTTTGGTTCGCTTTGAGTCTTCTTCGGTTCCGACTACCCTCCCGACTGCCTATGATGTTTATCCTTGAATGGTCGCCATGATGGTGGTTATTATACCGTCAAGGACTGTGTGACTATTGACGTCCTTCCCCGTACGCCGGGCAATAACGTTTATGTTGGTTTCATGGTTTGGTCTAACTTTACCGCTACTAAATGCCGCGGATTGGTTTCGCTGAATCAGGTTATTAAAGAGATTATTTGTCTCCAGCCACTTAAGTGAGGTGATTTATGTTTGGTGCTATTGCTGGCGGTATTGCTTCTGCTCTTGCTGGTGGCGCCATGTCTAAATTGTTTGGAGGCGGTCAAAAAGCCGCCTCCGGTGGCATTCAAGGTGATGTGCTTGCTACCGATAACAATACTGTAGGCATGGGTGATGCTGGTATTAAATCTGCCATTCAAGGCTCTAATGTTCCTAACCCTGATGAGGCCGCCCCTAGTTTTGTTTCTGGTGCTATGGCTAAAGCTGGTAAAGGACTTCTTGAAGGTACGTTGCAGGCTGGCACTTCTGCCGTTTCTGATAAGTTGCTTGATTTGGTTGGACTTGGTGGCAAGTCTGCCGCTGATAAAGGAAAGGATACTCGTGATTATCTTGCTGCTGCATTTCCTGAGCTTAATGCTTGGGAGCGTGCTGGTGCTGATGCTTCCTCTGCTGGTATGGTTGACGCCGGATTTGAGAATCAAAAAGAGCTTACTAAAATGCAACTGGACAATCAGAAAGAGATTGCCGAGATGCAAAATGAGACTCAAAAAGAGATTGCTGGCATTCAGTCGGCGACTTCACGCCAGAATACGAAAGACCAGGTATATGCACAAAATGAGATGCTTGCTTATCAACAGAAGGAGTCTACTGCTCGCGTTGCGTCTATTATGGAAAACACCAATCTTTCCAAGCAACAGCAGGTTTCCGAGATTATGCGCCAAATGCTTACTCAAGCTCAAACGGCTGGTCAGTATTTTACCAATGACCAAATCAAAGAAATGACTCGCAAGGTTAGTGCTGAGGTTGACTTAGTTCATCAGCAAACGCAGAATCAGCGGTATGGCTCTTCTCATATTGGCGCTACTGCAAAGGATATTTCTAATGTCGTCACTGATGCTGCTTCTGGTGTGGTTGATATTTTTCATGGTATTGATAAAGCTGTTGCCGATACTTGGAACAATTTCTGGAAAGACGGTAAAGCTGATGGTATTGGCTCTAATTTGTCTAGGAAATAACCGTCAGGATTGACACCCTCCCAATTGTATGTTTTCATGCCTCCAAATCTTGGAGGCTTTTTTATGGTTCGTTCTTATTACCCTTCTGAATGTCACGCTGATTATTTTGACTTTGAGCGTATCGAGGCTCTTAAACCTGCTATTGAGGCTTGTGGCATTTCTACTCTTTCTCAATCCCCAATGCTTGGCTTCCATAAGCAGATGGATAACCGCATCAAGCTCTTGGAAGAGATTCTGTCTTTTCGTATGCAGGGCGTTGAGTTCGATAATGGTGATATGTATGTTGACGGCCATAAGGCTGCTTCTGACGTTCGTGATGAGTTTGTATCTGTTACTGAGAAGTTAATGGATGAATTGGCACAATGCTACAATGTGCTCCCCCAACTTGATATTAATAACACTATAGACCACCGCCCCGAAGGGGACGAAAAATGGTTTTTAGAGAACGAGAAGACGGTTACGCAGTTTTGCCGCAAGCTGGCTGCTGAACGCCCTCTTAAGGATATTCGCGATGAGTATAATTACCCCAAAAAGAAAGGTATTAAGGATGAGTGTTCAAGATTGCTGGAGGCCTCCACTATGAAATCGCGTAGAGGCTTTGCTATTCAGCGTTTGATGAATGCAATGCGACAGGCTCATGCTGATGGTTGGTTTATCGTTTTTGACACTCTCACGTTGGCTGACGACCGATTAGAGGCGTTTTATGATAATCCCAATGCTTTGCGTGACTATTTTCGTGATATTGGTCGTATGGTTCTTGCTGCCGAGGGTCGCAAGGCTAATGATTCACACGCCGACTGCTATCAGTATTTTTGTGTGCCTGAGTATGGTACAGCTAATGGCCGTCTTCATTTCCATGCGGTGCACTTTATGCGGACACTTCCTACAGGTAGCGTTGACCCTAATTTTGGTCGTCGGGTACGCAATCGCCGCCAGTTAAATAGCTTGCAAAATACGTGGCCTTATGGTTACAGTATGCCCATCGCAGTTCGCTACACGCAGGACGCTTTTTCACGTTCTGGTTGGTTGTGGCCTGTTGATGCTAAAGGTGAGCCGCTTAAAGCTACCAGTTATATGGCTGTTGGTTTCTATGTGGCTAAATACGTTAACAAAAAGTCAGATATGGACCTTGCTGCTAAAGGTCTAGGAGCTAAAGAATGGAACAACTCACTAAAAACCAAGCTGTCGCTACTTCCCAAGAAGCTGTTCAGAATCAGAATGAGCCGCAACTTCGGGATGAAAATGCTCACAATGACAAATCTGTCCACGGAGTGCTTAATCCAACTTACCAAGCTGGGTTACGACGCGACGCCGTTCAACCAGATATTGAAGCAGAACGCAAAAAGAGAGATGAGATTGAGGCTGGGAAAAGTTACTGTAGCCGACGTTTTGGCGGCGCAACCTGTGACGACAAATCTGCTCAAATTTATGCGCGCTTCGATAAAAATGATTGGCGTATCCAACCTGCA

>eGFP

ATGGTGAGCAAGGGCGAGGAGCTGTTCACCGGGGTGGTGCCCATCCTGGTCGAGCTGGACGGCGACGTAAACGGCCACAAGTTCAGCGTGTCCGGCGAGGGCGAGGGCGATGCCACCTACGGCAAGCTGACCCTGAAGTTCATCTGCACCACCGGCAAGCTGCCCGTGCCCTGGCCCACCCTCGTGACCACCCTGACCTACGGCGTGCAGTGCTTCAGCCGCTACCCCGACCACATGAAGCAGCACGACTTCTTCAAGTCCGCCATGCCCGAAGGCTACGTCCAGGAGCGCACCATCTTCTTCAAGGACGACGGCAACTACAAGACCCGCGCCGAGGTGAAGTTCGAGGGCGACACCCTGGTGAACCGCATCGAGCTGAAGGGCATCGACTTCAAGGAGGACGGCAACATCCTGGGGCACAAGCTGGAGTACAACTACAACAGCCACAACGTCTATATCATGGCCGACAAGCAGAAGAACGGCATCAAGGTGAACTTCAAGATCCGCCACAACATCGAGGACGGCAGCGTGCAGCTCGCCGACCACTACCAGCAGAACACCCCCATCGGCGACGGCCCCGTGCTGCTGCCCGACAACCACTACCTGAGCACCCAGTCCGCCCTGAGCAAAGACCCCAACGAGAAGCGCGATCACATGGTCCTGCTGGAGTTCGTGACCGCCGCCGGGATCACTCTCGGCATGGACGAGCTGTACAAG

>EU650204.1 Nothobranchius furzeri strain GRZ mitochondrion, complete genome

GTTAACGTAGCTTAAATAAAGCATGACACTGAAGCTGTTAAGATAAACCTTAGCCTGGTTTCGTAAACACAAAAGTTTGGTCCTAACTTCACTATCAGTTTTAACTAAAATTATACATGCAAGTATCCGCATTCCCGTGAGATTTCCTTAAGTTTTCTTTTGAATTCTAAGAAATGATATCAGGCACAATAAATTTTTAGCCCAAAACATCAGGCTTAGCCACACCCCCAAGGGAATTCAGCAGTGAGAAATATTAAGCTATAAGCGAAAGCTTGACTTAGTTATAGTTAATAGGGCCGGTTAAACTCGTGCCAGCCACCGCGGTTATACGAGAGGCTCAAATTGATCGTTAACGGCGTAAAGCGTGTTTAAAGTACAAATAAACTAAAGCAGAATCCACCCAAAACTGTTATACGTTATTGGGTCAAAGAAAAACTTTTACGTAAGTAGCTTTAACTATCCTGAATACACGAGAGCTGTGAAACAAACTGGGATTAGATACCCCACTATGCACAGTCCTAAACTTTAATATCAAAATACATGTACATTCGCCAGAGTACTACGAGCATTAGCTTAAAACCCAAAGGACTTGGCGGTGCTTTAGCCCCACCTAGAGGAGCCTGTTCTATAACCGATAACCCCCGTTAAACCTTACCTTTTCTTGTTCTTCCCGCCTATATACCGCCGTCGTCAGCTCACCCTGTGAAGGATACTCAGTAAGCAAAGACAATATAATTTAAAACGTCAGGTCGAGGTGTAGCATATGAAAAGGAAAGAAATGGGCTACATTTCCTATGTCAGGATATTACGGACAACATTATGAAAATAAGAATCAAAGGAGGATTTAGCAGTAAGCAGGGACCAGCACACCCCGCTGAAGCTGGCCCTGAAGCACGCACACACCGCCCGTCACTCTCCCTAAACACCACACCCTAAATATATAATAACAATCTAAAACAAAAGGGAGGCAAGTCGTAACATGGTAAGTATACCGGAAGGTGTACTTGGCTAACCAGAGTATAGCTAAATTAGGAAAGCATTTCACTTACACCGAAAAGTTATCCGCGCAAATCGGGTTACTCTGATACTTAAAAGCTAGCTACACATAATAACCAACAAACAACTATTAATACACATTAATATACTAATAAACCTAATTAAACCATTTTTCATATCTCAGTATAGGCGATAGAAAAGGAAATTTAAGCTACAGATAAAGTACCGCAAGGGAATGCTGAAAGAGAAGTGAAATAACACAGTAAAGTATAATAAAGCAGAGATAAACACTCGTACCTTTTGCATCATGATTTAGCAAGAATAGACTAAGCAAAATGTATTCTAGTTTATCACCCCGAAACTAAGTGAGCTACTCCAAGACAGCCTAAAAAAGGGCAAACACGTCTCTGTGGCAAAAGAGTGTGAAGATCTTTGAGTAGAGGTGACAGACCTACCGAACTTAGTTATAGCTGGTTGTCTGGGACTTAGATAGGAGTTTAGCCTTTAACTTTCTTAAACTTAATTCTGACTAATGATCAAGATAACAAAAGAATGTTAAGGAGTTATTCGTAAGGGGTTCAGCCCTTATGAAAAAGGATACAACCTTGTCAGGTGATCAAAGAACATATACACACAAGGTTAATGTTTTAGTGGGCTTAAAAGCAGCCACCTAGGGAAAAAGCGTTAAAGCTCAAACATTACCACCACTTTTAATAAAGATTTTTTATCTCACATCCCCAAAATTACCAGACCTCTCCATCTTATTATGGAAGAGATCATGCTAATATGAGTAATAAGAGACCCTTCTCCTAGCACCCCTGTAATTAGGAGCGGAAAATCCTCCTAAAATTATCGGCCCCAATAAAAGAGGGAATAAAATAACAAAGTAATAACAAGAAAACTGTTTAAACATAACCGTTAACCCAACACCGGAGTGCATTATAGGAAAGATAAAAAGAAAAAGAAGGAATTCGGCAAATATACTATAAAGCCTCGCCTGTTTACCAAAAACATAGCTTCTTGAATTCTCAATATAAGAAGTCCCGCCTGCCCCATGACAAAGTTTAATGGCCGCGGTATTTTGACCGTGCAAAGGTAGCGCAATCACTTGTCTTTTAAATGAAGACCTGTATGAATGGCATAACGAGGGCTTAACTGTCTCCTTTTTCTCATCAATGAAATTGATCTTCTCGTGCAGAAGCGGGAATAAAAACATAAGACGAGAAGACCCTATGGATCTTTAATCGCAAGAATAGAGCATATTCAAATTTCTCAAACTAATGAGTATAATATAATGAGAACTATTCAAGTGACTTTGGTTGGGGCGACCGTGGAGAAAAATAAAACCTCCCTGAGGAATGAAGTAACTTCTTCCAATCCCAGAGTGACAACTCTAAGAAATAGTAATTCTAACCGCAATGATCCGGCAAAGCCGATCAACGAACCAAGTTACCCTAGGGATAACAGCGCAATCCTCTTCTAGAGCCCATATCGACAAGGGGGTTTACGACCTCGATGTTGGATCAGGACATCCTAATGGTGCAGCAGCTATTAAGGGTTCGTTTGTTCAACGATTAAAGTCCTACGTGATCTGAGTTCAGACCGGAGTAATCCAGGTCAGTTTCTATCTATGATATTTTCTACTCTAGTACGAAAGGACCGAGTAAAAAAGGCCAATAATAAATTAAGCCTTCCTCTTTACTTATGAAAACAAATAAATAAGAAAAAGATAATACTAATTAGGAAAAGATAATTCCACGCATTAGAATAACAAAACAAAATACTGGCTCAACTACCCCTATTAGGGTGGTTGAGCCTTTTTTGCCGACTCAACCACAGAGTTAGCATGGCAGAACTAGGTATAGCAAGAGGCCTAAGCCCTTTTTATGGAAGTTCAAATCTTCCTTCTAACTATGTCTCATCAAATTTTATTATATATTATTAACCCATTACTAATAGCAGTGTTTGTCTTATTGGCAGTTGCATTATTAACACTAATTGAACGAAAAGTACTAGGTTATATACAATTACGAAAGGGTCCTAATATTGTAGGGCCTTACGGCCTAGTACAACCCATCGCAGATGGTGTTAAACTATTTTTAAAAGAACCCGTATGACCCTCTACCTCCTCCCCCCTACTCTTCCTAGCTATACCCACCATTGCTCTTGCTTTAGCCTTAATTCTATGAGTTCCCATACCGATGCCCTCCCCTTTAGCGAACCTAAATCTCGGCCTTTTGTTTATCCTAGCCTTCTCCAGCTTAGCAGTATATTCAATCCTAGGCTCAGGTTGAGCTTCCAACTCCAAATATGCACTCATCGGAGCCCTACGGGCCGTGGCTCAAACAATTTCATATGAAGTAAGCCTAGGATTAATTCTTCTTAATACTATCCTTTTCACTGGGGGCTTCACGCTACAAGTTTTCAGTACAGCTCAAGAAAATACTTGACTAGTATTCACAATATGGCCTCTAGCTACAATATGGTATGTATCGACACTAGCAGAAACAAATCGAGCACCCTTTGATTTAACAGAAGGGGAATCAGAACTAGTATCAGGCTTTAACATCGAGTATTCTGGAGGACCTTTTGCATTGTTTTTTCTAGCAGAATATGCAAACATCATCCTTATAAACATACTATCTGCTATTTTGTTCATAAGCACAAATCTACTTTATAATAATCTAAACAACGAAACAATAGTTATTATAATCAAAACAACCCTATTAAGCATAGTCTTCCTATGAGTTCGGACGTCCTACCCACGTTTTCGATATGACCAACTAATACACCTTGTATGAAAAAACATTCTTCCAATTACTATGGCATTTTTAATATGGCACCAAGCCATTCTTGTTTCCTTTGCAGGTCTTCCCCCTCAAATCTAGAGGAGCTGTGCCTGAATAAAGGATTACTTTGATAGAGTAAAAAATAAGGGTTAAAACCCCTTCAACTCCTTAGAAAAAGAGGATTTGAACCTCTACTTAAGAGATCAAAACCCTTCGTGCTCCCATTACACCACTCTCTAGTAGAGTCAGCTAATATAAGCTTTTGGGCCCATACCCCGAACACGTTGGTTAAAGTCCTTCCTCTACTAATGAGCCCTTTGACCCTTCTAACTATAGCATTCACCTTAATACTTGGAACCACCATTACACTCATAAGTACCCATTGGTTACTAGCCTGGATGGGCTTAGAAATTAACACATTGGCAATCATCCCCTTAATAATTAAGCAAAATCACCCCCGAGCTGTAGAAGCCACTACCAAGTACTTCCTAATTCAAGCTACCGCAGCCACAACATTACTGTTTGCTGCCACAACAAATGCCTGACTCACAGGAGCATGAGAAATCTCACAAGTAATACACCCCCTACCCACAATTATTATCACAATGGCCCTTTCCCTAAAAATAGGCCTAGCACCTATGCACTCTTGACTCCCAGATGTTATGCAAGGCCTCGAATTGAAAGTAGGGTTAGTCCTGGCCACCTGACAAAAACTAGCCCCCTTTTCTTTACTGTGTCAAATTCCCAACAGCAACTTTACAATTTTATTAGGATTAACATCTATCATCCTAGGAGGATGAGGAGGTTTAAATCAAACTCAATTACGAAAAATTATAGCCTATTCCTCAATCGCTCATCTAGGCTGAATACTTTTAGTAATACAACTACTCCCTTCATTATCCCTAATAACTTTATTAGTTTATATCATCATAACATTTTCATTATTTAACATCTTCATGATTATAAGACAACCAATATTAACTCCTTATCAACCTCCTCCATAAAAGTACCCGTCTTAACTACCCTTACTCCATTGATCTTACTATCGCTGGGAGGTCTTCCTCCCTTAACCGGATTTTTACCAAAATGATTAATCCTACAGGAAATAATTAAACAAAATTTTTTTATAACAGCAACCATAGCGGCTCTATCAGCCCTACTCAGCTTGTACTTCTACCTACGATTGTCTTATAACATGACTTTTACCATTGCTCCTAATACATCAACAAACACACTATCCTGACGTCTAAACAACAAAAATATCATTCTACCAACCGCTACCTCAATAACTATAACCCTGCTTATACTCCCCCTTCTTCCCCTTGCAGCAATACCTTTATTTTAAGAGTTTAGGTTAACATCAAACCCAAAGCCTTCAAAGCTTTATATAAGAGTGCAAATCTCTTAACCCTTAAGACCTACAGGATTCTAACCCACATCTTCTGCATGCAAAACAGACACTTTTATTAAGCTAAAGCCTTCTAGACGAGTAGGCCTCGATCCTACAAACTTTTAGTTAACAGCTAAATACTCAAACCAGCGAGCATCCGTCTACTTTCCCCGCCTAGTCGGGGGAGAAATAGGCGGGGAAAGCCCCGGCAGACGATAATCTACCACTCACGGTTTGCAACCATGCATGTACACACCTCAGGGCTGGGGACTGGGAGAAAGAGGACTCAAACCTCTATTTAGGGAGTTACAATCCACCACTATTTCAGTCATTCTACCTGTGACCATCACTCGTTGATTTTTCTCCACTAATCATAAAGATATCGGCACCCTTTATTTAATCTTTGGTGCCTGAGCCGGAATGGTAGGCACAGCCTTAAGCCTCCTGATCCGAGCAGAATTAAGTCAGCCCGGGTCTCTTCTAGGTGACGACCAAATCTACAATGTCATCGTTACAGCACATGCCTTTGTTATAATTTTCTTTATAGTAATACCAATCATAATTGGCGGTTTCGGAAATTGACTAGTACCCCTCATAATCGGAGCACCTGATATAGCTTTTCCCCGAATAAACAACATAAGCTTTTGACTTCTACCCCCCTCCTTCCTTCTTCTATTAGCCTCCTCAGGGGTTGAAGCAGGCGCAGGGACAGGCTGGACAGTCTATCCCCCCTTAGCAGGAAACATAGCTCATGCAGGAGCCTCTGTAGACCTAACAATCTTCTCCCTCCATTTGGCAGGTGTTTCTTCAATTCTAGGGGCCATTAACTTTATCACAACTATTATTAATATAAAACCACCAGCTATTTCACAATATCAAACCCCTTTATTCGTTTGAGCCGTCTTAATCACGGCTGTACTACTTCTACTTTCACTGCCTGTCTTGGCCGCAGGTATTACAATACTACTCACAGATCGAAATTTAAATACGACTTTCATTGACCCAGCTGGAGGAGGAGATCCAATTCTTTATCAACATTTATTTTGATTTTTTGGTCACCCAGAAGTTTACATTCTAATTCTACCAGGTTTTGGCATAATTTCTCATATTGTAGCCTACTATTCAGGTAAAAAAGAACCTTTTGGCTACATGGGTATAGTCTGAGCAATGATAGCAATCGGCTTACTCGGCTTTATTGTTTGGGCTCACCACATATTTACCGTTGGTATAGACGTGGACACCCGAGCTTACTTTACATCTGCAACCATAATCATCGCTATTCCCACAGGGGTGAAAGTATTCAGTTGATTAGCTACCCTTCATGGAGGCGCAATTAAATGAGAAACACCCTTACTTTGGGCTCTTGGTTTTATTTTCTTGTTTACAGTAGGCGGCCTAACGGGGATTGTTCTAGCCAACTCATCTCTTGATATTGTTCTTCATGACACCTACTATGTAGTAGCACACTTCCACTATGTCTTATCAATAGGGGCAGTATTTGCCATCATTGCCGCATTTATTCACTGATTCCCCTTATTTTCAGGCTACACGCTTCATGAAACATGAGCTAAAATCCACTTTGGTGTAATATTTTTAGGAGTAAACCTTACATTCTTCCCACAGCATTTCCTAGGCTTAGCAGGAATACCTCGACGCTATTCAGACTACCCAGATGCCTACACAGTTTGAAATACTGTTTCTTCTATAGGCTCCTTAATCTCTTTAATTGCCATAATCATATTCCTATTTATTATCTGAGAAGCATTTTCATCAAAACGTGAAGTTCTTTCAGTAGAATTAACTCAGACAAATGTTGAATGGCTTCATGGCTGTCCTCCTCCTTATCACACTTTTGAAGAACCTGCATCTGTTCGATGCAATTAATACCGAGAAAGGAAGGAATTGAACCCCCATAATTTGGTTTCAAGCCAATTACATAACCGATCTGTCACTTTCTTAATAAGACCCTAGTAAAATATTACCTTGTCTTGTCAAGACAAAATTGTGGGTTAAAACCCCGCGAGTCTTAAAAATGGCACATCCCTCCCAACTAGGTTTTCAAGATGCAACTTCTCCTGTAATAGAAGAACTTCTTCACTTTCATGATCACACTTTAATAATTGTTTTTTTAATCAGCACGTTAGTACTATACATCATTATTGCAATAGTAACCACCAAACTTACTAATAAGTTTATCCTTGACTCACAAGAAATTGAAATTATTTGAACATTACTTCCAGCCATGATTTTAATCTTAATCGCTCTTCCATCGCTTCGTATCCTGTACCTAATAGATGAAATTAGCAACTCTCATCTCACTATTAAAACCATTGGGCACCAATGATATTGAAGCTATGAATATACAGATTATGAAAACATCACATTCGACTCCTATATAACTCCTACCCAAGATCTAATGCCTGGTCAGTTCCGATTACTAGAAACAGATCACCGAATAGTAGTTCCTATGGAGTCACCTACCCGAATCCTCGTTTCTGCAGACGACGTTTTACACTCGTGAGCTGTTCCAAGCCTTGGCATTAAAATAGATGCAGTACCAGGACGGTTAAACCAAACAGCATTTATTGTCTCGCGACCAGGTGTATATTACGGCCAGTGCTCTGAAATTTGTGGAGCTAACCATAGTTTTATACCCATTGTTGTTGAAGCTGTTCCACTAAAATACTTTGAAGATTGATCTTCCCTAATACTCCAAGATGCCTCGCTAGAAAGCTAAATGGGTGTAAGCATTAGCCTTTTAAGCTAAACATGGTGCCTCCCAACCACCTCTAGCGAAATGCCACAACTCAACCCCAATCCCTGATTTATAATCATAACCTTCGCCTGATTACTTTTCCTGATTGTTATTCCCCCAAAAGTATTGGCACATACAACTCCTAATAATGCTTTACCACAAAAAGTTGTATGTTCTCGTACAGAGTCTTGAGTCTGACCATGATATTAAATTTATTTGATCAATTCATAAGTCCCACACTATTAGGAATCCCACTAATACTATTAGCTTTATCCCTACCTTGAGTTTTGTACCCGAAACCTCTTAAACGCTGACTTAATAACCGAACTTTAACAATTCAAAGTTGGTTTATTAATAACTTTATTAAACAAATTTTTTTACCTATAAACACAGAAGGTCATAAATGAGCAATATTACTAACATCCCTACTAATTTTCTTAATCACACTTAATCTTCTAGGACTCCTCCCATATACTTTTACACCAACTACGCAACTCTCCCTTAACATAGCATTCGCAACTCCTATCTGACTTACTACTGTGATCTTAGGCATACGAAAACAGCCCACCCACTCTTTAGGCCACCTACTACCAGAAGGCACCCCAACCCTGCTAATCCCTATTTTAGTTGTTATTGAAACTATTAGCCTGTTTATCCGACCTCTAGCTTTAGGTGTCCGACTTACAGCCAATCTAACAGCAGGACACCTTTTAATCCAACTAATTGCCTCAGGAGCCTATTTCCTGTTCCCCCTCATACCCGCAGTAGCATTGCTTACTTCTACACTCCTCTTTCTTTTAACCCTCCTAGAAGTAGCTGTTGCTATAATCCAGGCATACGTCTTTGTTCTTCTGCTAAGCCTCTACTTACAAGAAAACATCTAATGGCCCACCAAGCACATGCGTATCATATAGTAGACCCAAGCCCATGACCCCTTACAGGTGCAGTTGCCGCTTTACTCCTAACATCCGGCTTAGCAATTTGAATACATTTTAACTCTATATTCCTCTTAATAATTGGTTTAACTCTCATACTGTTAACCATAACCCAATGATGACGGGATGTTATTCGAGAAGGCACATTTCAAGGACACCATACACTACCTGTTCAGAAAGGACTTCGTTATGGGATAATCTTATTTATCACCTCCGAAGTCTTTTTCTTCTTAGGCTTCTTCTGAGCATTCTACCACTCAAGTCTAGCTCCCACCCCCGAACTTGGCGGGTGTTGACCACCAACAGGCATTAGTACACTCAACCCATTTGAAGTCCCACTTCTAAACACAGCAGTTCTTCTTGCTTCAGGTGTAACAGTTACCTGAGCACATCACAGTATTATAGAAGGTCAACGAAAACAAGCAATTCAATCATTAACTCTGACTATTATTCTTGGATTTTACTTTACTATCCTTCAAGCAATAGAGTACTATGAGGCACCTTTTACAATCGCAGACGGTGTCTATGGCTCAACTTTCTTTGTAGCCACTGGTTTTCACGGCCTGCATGTAATTATTGGATCTTCATTTTTAATAGTATGCTTATTGCGACAAATTAATTTTCACTTTACTTCACAACACCACTTTGGATTTGAAGCTGCAGCCTGATATTGACACTTCGTTGATGTAGTGTGACTTTTCCTCTATGTTTCTATTTATTGATGAGGATCTTATCTTTCTAGTATAGGTAATACTAGTGACTTCCAATCACTTAACCCTAGTTAAATTCTAGGGAAAGATAATGAATATAATCTCTACTACATTGCCCATGTTCCTAATAATTTCAACAATTCTTGCCATCTTAGCATTTTGGTTACCCTCGTTAAACTCTAATTATGATAAATTATCCCCTTATGAGTGCGGTTTTGACCCTTTAGGCTCAGCTCGACTTCCTTTCTCAATACGATTCTTTCTAATCGCTATCCTATTCATCCTCTTTGATTTAGAAATTGCCTTGTTACTCCCTCTCCCTTGGGGAAACCAACTTCCTGATCCCCTGACCTCGTTCTCATGAATATCAGCAGTATTAACACTTCTAACCCTTGGATTAATTTATGAGTGAACCCAAGGAGGCCTAGAATGAGCAGAGTAGGTTATTAGTTTAATAAAAACATTTGAATTCGGCTTAAAAAATTGTGGTTAAACTCCATAATAACCCTGATGATTTTTACATTAATAACTTTTTCATTTATATTCGTCTTAGGCCTGATAGGCCTAACATTTTACCGAACACACTTACTCTCTGCACTTCTATGTCTTGAAGGTATGATGCTATCTATTTTCATAGCTTTTTCCCTATGAGTCCTCCAAATGGAGTCAGACAACTTCTCTACACCTCCTTTGCTACTTCTTACCTTTTCGGCTTGTGAAGCAAGTATTGGTCTTGCTTTACTAATTGCTATCGCACGAACCCACGGCTCAGATCGCATTAACACCCTAAACCTGTTACAATGTTAAAAGTTATTTTACCCACACTCATATTAATTTCATCAACCTGGGTTTCACCCTACAAAAACATATGATCCTCCATTCTTTTGTATAGTTTTATTATTGCTTCGACTAGCCTTGGTTGAATTTCTTATTCAACCATCACTAACTGAACTACAACTAATACCCTTATAAGTACAGACTCTTTATCATCACCCCTATTAATCCTTACCTGCTGACTCCTTCCACTAATGATTTTAGCAAGTCAAAAACATGTTTTAAATGAGCCTGTAAATCGACAACGAGTCTATCTTACCTTACTGATCTCCCTACAATTCTTCTTAATTCTAGCTTTTAGCGCTACAGAAACCATTATGTTCTATGTTATATTTGAAGCCACCCTCATCCCAACATTAATACTTATTACACGATGGGGCAACCAAAAAGAACGGTTAAACGCAGGCACTTATTTTTTATTTTACACCCTAGCAAGCTCACTACCCCTTCTGGTAGCTCTACTCATTCTACATAGCTCAACAGGAACATTATCCTTCTTTACAATAAGCTATTTAAGTTCCCCCACCCTGACCCCTATTTCACATCAACTATGATGACTCGGATGTCTCATAGCTTTTTTAGTAAAAATACCTCTTTACGGGGTTCACTTATGGTTACCCAAAGCGCACGTAGAAGCACCTATTGCTGGATCAATAGTACTTGCTGCTGTGTTATTAAAACTAGGAGGTTATGGTCTTATGCGAATTATAACAATCCTAGAACCCTTAACCAAAAACATAGCTTACCCCTTTATCATCTTGGCACTATGAGGAGTAATTATAACAGGATTAATCTGTCTACGACAAACTGATCTAAAATCTCTAATTGCCTACTCATCAGTAAGCCACATAGGTCTCGTCGCAGCAGGGATCTTAATTCAAACTTCATGAGGATTCTCAGGAGCATTAATCCTAATAATTGCACACGGCCTAACCTCATCAGCCCTATTTTGTTTAGCTAACACTAACTATGAGCGAACACATAGCCGAACAATGATCATTACCCGAGGATTACAAAGCATCTTTCCCTTAATAGCTGCCTGGTGATTTATTTCAAGTTTAGCAAATCTTGCTCTTCCTCCACTTCCCAATCTCTTGGGAGAATTACTAATCACCTCCTCATTATTCAATTGATCCGTTTTCTCGCTTATCTTTACAGGTTTGGGAACCTTAATTACAGCTAGTTATTCACTACACATATTCCTTACAGCACAACGAGGAACACCTCCCAACCACATAATACTTTTAAGCCCCACATACACACGAGAACACCTACTTATCTTTCTGCATATCATACCTCTTGTCATATTAATAATGAAACCAGAACTAGTTTGAGGATGAACTACATGTGGCTATAATTTAGACAAAATATAAGATTGTGATTCTTACAACAAAGGTTAAAACCCTTTTAGCCACAGAGAGAGGCATGATCGTAGCGATGACTGCTAATTTTCGCACTCTTGGTTTGAATCCGGGACTCTCTCAAACTTCTAAAGGATAATAGCTGATCCGTTGGCCTTAGGAGCCAAAAACTCTTGGTGCAACTCCAAGTAGAAGTTATGCACAACTTATCCTTAATATTTTCATCCACTATATTTTGACTTTTATTAATTCTTATTTATCCTCTATTAGACACTTTAAAACCTCGACTACCATCAGACTGATCCCGCAAAAAAGTCAAAACAGCTGTAAAATTTACATTTTTTACAGCATTATTCCCCTTAATACTTTTCATTGACCAGGGCCTGGAAACAATTATCACTACATGAGCATGGACCGACACACTATTTAACATTAATCTAAGTTTTAAATTTGATTCTTACTCTTCAATCTTTTTGCCCATCGCCCTTTATGTAACTTGATCAATCTTAGAATTTGCTCTATGATACATGCACTCAGATGCATATATTAATAAATTCTTTAAAAACCTTTTAATTTTTTTAATCGCCATAATTGTTCTTGTAACTGCTAATAATCTGTTTCAACTTTTTATTGGTTGAGAAGGTGTAGGAATTATGTCATTTATCTTAATTGGGTGATGATATGGCCGAGCTGATGCCAACACAGCTGCTCTGCAAGCAGTGATTTACAACCGAGTGGGAGATATCGGCTTAATTCTAGCTATGGCTTGAATAGCAATAAACCTTACCTCATGAGAAATTCATCAAATCTTCATAACCTCACAACTAACAACCTCAACGCTCCCACTCTTGGGTCTAATCCTTGCTGCAACCGGCAAATCAGCCCAGTTTGGGCTTCACCCTTGATTACCCTCAGCCATAGAAGGACCTACCCCTGTTTCAGCATTGCTTCACTCAAGTACTATAGTCGTTGCTGGTGTATTTTTATTAATTCGGTTTAGCCCTCTAATAGATAATGACCCAATTGCTCTTTCTACATGTCTCTGTCTAGGGTCTATTACAGCACTATTTGCCGCTATCTGTGCCCTCACACAAAATGACATTAAAAAAATTATTGCATTCTCCACTTCAAGCCAACTAGGCTTAATAATAGTTACCGTCGGCCTGAACCAACCTCAATTAGCCTTCATTCACATCTGCACTCACGCCTTTTTTAAAGCTATATTGTTTTTGTGTTCAGGATCCATTATTCACGCTATAAAAGATGAACAAGATATTCGAAAAATAGGAGGCTTAAACCATTTACTGCCTGTAACCTCTTCTTGCCTAACAATCGGCAGCTTAGCCCTATTAGGTATACCCTTCTTAGCAGGATTCTTTTCAAAAGATGCCATCATTGAGGCCATAAACACATCACACTTAAACGCCTGAGCCCTAATCATTACTCTAATTGCAACCTCTTTTACTGCAGCCTACAGCTTTCGAATCGTGTACTACGTTTCAATAGGGTACCCCCGATTTAACACCTTGTCTCCCATTAATGAAAATAATAATGCTATCATCAACCCTTTAAAACGGTTAGCATGAGGAAGTATTTTAGCAGGACTTTTAATTTTTTCTGACACCTTACCTTTCAAAAACCCAATTCTCACTATACCCACAAGCATTAAGATAGCCGCTATCTTGGTTACTCTTTTAGGCTTTTTAACTGCTATAGAAATATCCATCATAATATCTAAACAGCTGAAACTAACCCCTAAACTTACACCCTTTAACTTTTCAAATATACTGGGTTACTTCCCGACAGTGATCCATCGAGTTTTTACTAAAATGTCTCTTTTAATAGGACAACACATCTCTAATCAAACAATTGATCAAACTTGACTAGAAAAAATTGGTCCTAAAACAGTCTCTACAATAAGTAACAAACCCATCCACCTAGTAGAAAACATACAGAAAGGTTTAATTAAAACATATTTATCCATTTTCTTCATCTCTACAGTAATCTTTACCTACCTCTTTTCACTAAACTGGGCGTAGTACTGAAAAGCTTATGCCTCAAGTTAATTTAAATACAACAAATAATGCTAGAAGTAATACTAAAGCCCCTAATACCAATAAGACCCCACCACATAAATATATGAGCGCTACTCCCCATGCATCCTTTTGAACTATATTAAAACTAGAAATCTCACCTAGTAAATGTAATGGCCCATCATAAAAATGCAAAACCATAGCAGCAGTAAAAAATACTAAACACATATAAGAAATTATTAAACCCAGCACATAACCACTCCCCAAACTTTCAGGAAAAGGCTCAGCAATTAACGCTGCCGAATACCCAAAAACAACCATTATACCACCTAAATAGATTCAAAACAAGATCAAAGAGAAAAAAGGACCACTTAAAATTGCTAAAGCAAGACAACCTACCCCAGCAACTAATACAAGACCTAATGCAGTAAAATAAGGAGGGGGGTTTGAAGCCACAAATGCCAAACCCAAAATCAAGAGAACTAAACTCAAACACATTATAATTGACATAAATCTTACCTGGGTTTTAACCAGGCTCACTAACCCAAAAACTACCGTTGTATTCAACTATTAAAATAAATGGCCAACCTGCGAAAAACACATCCTTTAATCAAAATTGCAAATGACTCCCTAATTGACCTTCCCACCCCATCTAATATTTCAGCATGATGAAATTTTGGCTCCTTATTAGGTCTCTGCTTAATTAGCCAAATTATCACTGGATTATTCTTAGCCATACATTACACCTCGGATATTTCAACTGCTTTCTCTTCAGTAGTACATATTTGCCGAGATGTCAACTACGGATGACTCATCCGCAATATTCATGCTAACGGAGCCTCATTCTTTTTCATCTGCATTTACCTTCATGTGGGGCGAGGGCTTTATTATGGGTCCTACCTTTATAAAGAGACATGAAATCTAGGCGTAATCTTATTACTTCTCCTTATAATAACAGCATTTGTAGGGTATGTACTTCCATGAGGACAAATATCATTTTGAGGTGCTACAGTAATCACTAACCTTCTCTCAGCAATCCCATACATTGGAAACAACCTTGTCCAATGGATTTGAGGGGACTTCTCAGTTAATAACGCCACTCTCACCCGCTTTTTTGCTTTCCATTTTTTGCTTCCTTTTATTATTTTAGCAACAACTATAATTCACCTTATCTTCCTACATGAAACGGGCTCTAATAACCCCACGGGTATCACAACGGACTCGGATAAAATCACCTTTCACCCCTACTTCTCATATAAAGATCTATTAGGTTTTGCTATTTTAATCATACTTCTAGTATCACTATCCCTTTTTGCTCCTAACCTTCTAGGGGACCCTGAAAACTTTACCCCTGCTAATCCTCTAGTCACACCACCCCACATTAAACCAGAATGGTATTTTCTGTTTGCCTATGCTATCCTCCGATCTATTCCTAATAAACTAGGGGGAGTTATTGCCCTCTTTGCCTCCATCCTAGTTTTATTCTGTGTCCCTCTCCTCCACACCTCGAAACAACGATCCCTCACATTCCGCCCCCTTGGGCAGGTCTTGTTTTGACTATTAGTGGGAGATATTATCACCCTAACATGAATTGGAGGAATACCTGTCGAACACCCTTACATCATCATTGGACAAATTGCATCACTAATCTACTTTATAATCTTACTAATTTTCATACCCGCTCTTAACTTGTATGAAAATAAAATTTGATGATAAGTACTCATAGCTTAAATAAAGCATTAGTTTTGTAAACTAAAAATGAAGGTTAAACCCCATCTTAGTACTAAACAGAAGTTTTAATCTTGTCTTTGTACTCAAAACCAAAATTCACCCAACCCACCCCATATACTGGGGTCTAAATATCTGAAAGCTTTCAGCTACAAACATATCAAACTTCACCCATCTCTGCCAGGGGGGGGGGGGGGGGGGCTTGCAGCTGGTGGAGCGAGACACCCCCCTCCGCCCCAAATCTGCCAGCAATTTTACTCTCCCAAGACCTCTTATGATTCGCCTACCTCCGAGTTCGAAAAGGCCGCCAGAATCCGCCATAAAAAGGGGGGAAAAGGTTACCTAAGAAAAAATCTTTTACTTTTTTTTACTTTTTTTGTTAGGGTTTTCCTCTTGGCCTCGGAAGGGGACTTCCTATTTTTCGCAGGATTCTGCCAATTGGGTCTAGGCGTCAAGTACTTGTATGTCATATCATCATTAAATGATATGCATCATTTA

AACGATGGTGCTATAAAAGCACCATAGAATAATCCCTACCTAACTATTTTTACCCATTCAGGCGACAAAACTGGATGCAACAAGCACGGGCGCCTGATTTGTTTAATCCTTGTTCTACCTATTGGGCTGTGCGTTTAATTCACATATACCAGGACTCAAGCACTCTGCAAGTCAGTACCGTTGCACAGTAAGAAACCAGCAACAGGTTGATTTCTTAAGGTGAAACGTCATTGATGGTCAGGGATCGAACTCGTGGGGGTCGCTCTTTCTCACATTTTTCCTAGCATTTGGTTCCTACTTCAGGGCCATTGATTGATAATCACTCCCCTACTCAATGCCTATCCAAGAGGCATTTGATTAATGGCGGTATACCACGGCGGGAGCACCCCCCAAGCCGGGCGTTCTTTTCCAGTGGCTATAGTTCTTTTTTCTTTTTTTTTCCTTTCTATAGACTTCTCACAGTGTCAGTAATCTATGATTGAAAGGTGGAACTAATCCTAGGAATTAAATGTTATAGGTGAGAGTTGGAATGGATGTCTTTGAAGGTTTGCTTAATTGATATTTAAAGCATAACAAGTGCTCTATCTAGACGAGTTTCCTTGAAGTGAGTTTCAGAGAATCCTATTGGGTTTTCCCCCCCTTCCCCCCCAATTCCAAATAAACCATTCATCACACACCCTATGAAAATAAAATCTACAATTTCGAAAAGAAATAAAATTTTAATCTAAAATCTTAATGCTAAACATTAAAAAATCGCACTTAAATAAAAATCTTAAAAATGAGGTAGCCTACAAATTTATCAGTACAGAACTTGGAAATACCTCACACCCTAAAACAGAATAAATGAGCGCAAGCAAGTTAAAAATCCACAATTTTAAAAAACTTTACAAGACTGAAATCTGAAAAAACACCTCATCACCTACAACTACACCCCCCCCATAACTGTCATTTTTAACACATATTAACAAAATTTTCAAGTAATTTTCAACCGCCTCCCTGCTACTCCAGTAAATTAACACATCAGATCATATTGTTTCTTAAATCCTTGCTCTAGTTAATCATGATCACAATTGCATTTAACATCAGACCATTAAGAAGAACTACTTTAGAATACCTCAACAGTTGCAACCTCCTTCAAAACCTACCTAGCACCAATAATTTAAAAGACCTTTTCACTCACATTCTCACACATCCATTTTGCACCCTCTCTCGCAATCTTTTACTACTTTAACAACAACTATAACCCACCTCTTCTCCCCTAACAGATCAACAACCCCTACAAACCAAAACGATGTCTTCTATCACATTAACCCTTCAACCACGACAATGACCAGAGACCACTTCTCTCAGCCCTAAATTTGCCTTATACCTGTCTTTCCTCTACACCTCAAGACAACAATCCTACACTCTACCCCAGCAAGTGGTACTTACTTCTAATAAACGTATACTTTCCAGTATAAATCAAATTCACATCTACCATACACCCTTATATGAATATAATTACATTCTACCGGATGAACTACATCACCAAATCATTTCACACCCCCACTATTATACGCTCACTCTTGACCTGTAAAAGGCCCTCAAAAATCTACATTAATAATTTAACGGTAAAAAAATGCTTTATGATACCTCAAACCCCGCTTACAATCAGAAAAAAAGAATTTTAATCTCTATCCCTGACTCCCAAAGCCAGAATTTTACTTAAACTATTTTCTGCCATCGGGGGGGGGGGGGGGCTTGCAGCTGGTGGAGCGAGACACCCCCCTCCACCCCAAATCTGCCAGCAATTTTACTCTCCCGAGACCTCTTATGATTCGCCTACCTCCGAGTTCGAAAAGGCCGCCAGAATCCGCCATAAAAAGGGGGGAAAAGGTTACCTAAGAAAAAATCTTTTACTTTTTTTTACTTTTTTTGTTAGGGTTTTCCTCTTGGCCTCGGAAGGGGACTTCCTATTTTTCGCAGGATTCTGCCAATTGGGTCTAGGCGTCAAGTACTTGTATGTCATATCATCATTAAATGATATGCATCATTTAAACGATGGTGCTATAAAAGCACCATAGAATAATCCCTACCTAACTATTTTTACCCATTCAGGCGACAAAACTGGATGCAACAAGCACGGGCGCCTGATTTGTTTAATCCTTGTTCTACCTATTGGCTGTGCGTTTAATTCACATATACCAGGACTCAAGCACTCTGCAAGTCAGTACCGTTGCACAGTAAGAAACCAGCAACAGGTTGATTTCTTAAGGTGAAACGTCATTGATGGTCAGGGATCGAACTCGTGGGGGTCGCTCTTTCTCACATTTTTCCTAGCATTTGGTTCCTACTTCAGGGCCATTGATTGATAATCACTCCCCTACTCAATGCCTATCCAAGAGGCATTTGATTAATGGCGGTATACCACGGCGGGAGCACCCCCCAAGCCGGGCGTTCTTTTCCAGTGGCTATAGTTCTTTTTTCTTTTTTTTTCCTTTCTATAGACTTCTCACAGTGTCAGTAATCTATGATTGAAAGGTGGAACTAATCCTAGGAATTAAATGTTATAGGTGAGAGTTGGAATGGATGTCTTTGAAGGTTTGCTTAATTGATATTTAAAGCATAACAAGTGCTCTATCTAGACGAGTTTCCTTGAAGTGAGTTTCAGAGAATCCTATTGGGTTTTCCCCCCCTGGCCCCCCCCCCAAAATACTACCATCTATGAAAACTAGAAGCAAAATCTTCAGTTTTCAGAACAAAAGGACAGAAAAATAAGTCAATTAACCCGATAAATCAAAATAGAACCTCACACGTGAATAAAATATGCAAAATAAGCACCTGTAAATTAAGCAAGCATGCTGAACCTCTTTTCCACACCATCAACTAAATCTGATATAAAAATAAACAATTGAATACTCCAAATTCCTGCCAGGACTTTAACCAGGACCAGCAATTTGAAAAACTACCGTTGTATTCAACTACACGAACGAGTGATATGCGTAAAATGCATCCCTTACACCCTCCCTTTTCTCTCCACTGGTAATAAAACTTAAGCTGTTTTATAAACAAACCGCCTACATTTAAAACATATTAAAAATCTTTATTCTTCAAAAACTACCCAAATTTAATAGTAATGTCAGGGCCCCTAAAAATATAATCACCTCAATATGTACAGTTTTATTTTCAAATTACACAACTGTGTCACGTAAATCACCATTTGCAATTTACAATTTTTGCATTTGCAATGATGACATTACACACCATAAATCACAATTTTTTTTTTAAAAAACATAGTAATGTCAGGGCCTTAAAGCCAAACTACTGTAAAATATAATTTTATCTTTCAAATGTAATACATCAGTCACACTTACAGGTACTAATTTGTAGCTACAATCATGATTTATACATTATGCAATTTAACTTTTAAACACACACTCAGCACCAAATGCCAGAATATTAAATACCTTACAATATCCTATCTTCACGATGTAATTAACACCCTTTTAAAAGATGAG

>EU780557.1 Nothobranchius furzeri 5' external transcribed spacer, partial sequence; 18S ribosomal RNA gene, internal transcribed spacer 1, 5.8S ribosomal RNA gene, internal transcribed spacer 2, and 28S ribosomal RNA gene, complete sequence; and 3' external transcribed spacer, partial sequence

GGCAATAACCTGGTAGAACGACCAAAGTGCTCTACTCGGGCCAGACTCGGACCCACGACCCGGGGTGAGAACCACAACTACTGGTCATCCTTTAGACCTCCAGGGGGCCCGGCACAGCCTCCCTAGTCCGACACAAGTGCTCCACTTGGGCTATACTCTGACCCAAGTCCCGGGGCGAGAACCACAACTACTGGTCATCCTTTAGACCTCCAGGGGGACCGGCACAGCCTCCCTAGACCGACACAAGTGCACCACTTGGGCCATACTCTGACCCAAGTCCCGGGGCGAGAACCACAACTACTGGTCATCCTTTTGACCTCATGGTCATCCTTTAGACCTCAAGGGGGCACGGCAGAGCCTCCCTAGTCCGACACAAGTGTCCCACTTGGGCTATACTATGACCCAAGTCCCGGGGCGAGAACCACAACTACTGGTCATCCTTTAGACCTCAAGGGGGCACGGCAGAGCCTCCCTAGTCCGACACAAGTGTCCCACTTGGGCCATACTCAGACCCACGGCCCGGGGCGAGAACCACAACTACTGGTCATCCTTTAGACCTCCAGGGGGCCCGGCACAGCCTCCCTAGTCCGACGCAAGTGCTCCACTTGGGCTGTACTCTGACCCAAGTCCCGGGGCGAGAACCACAACTACTGGTCATCCTTTAGACCTCCAGGGGGCACGGCACAGCCTCCCTAGTCCGACACAAGTGCTCCACTTGGGCTATACTCTGACCCAAGTCCCGGGGCGAGAACCACAACTACTGGTCATCCTTTAGACCTCCAGGGGGCACGGCACAGCCTCCCTAGTCCGACACAAGTGCTCCACTTGGGCTATACTCTGACCCAAGTCCCGGGGCGAGAACCACAACTACTGGTCATCCTTTTGACCTCATGGTCATCCTTTAGACCTCCAGGGGGCCCGGCACAGCCTCCCTAGGCCGACACAAGTGCTCCACTTGGGCTATACTCTGACCCAAGTCCCGGGGCGAGAACCACAACTACTGGTCATCCTTTAGACCTCCAGGGGGCACGGCACAGCCTCCCTAGTCCGACACAAGTGCTCCACTTCGGCTGTACTCTGACCCAAGTCCCGGGGCGAGAACCACAACTAATGGTCATCCTTTAGACCTCCATGGCAACAGGGGGATGTCAGACCCCAGCTCATCCCAGGTTGATGCCTCAATAGTAGCTTAGGGTCACGGCAGTCCCACCGTGATCCACCCTCTTGTCCTCTCTCCACAGGATGACCAGAGTGTCGTGTTTATTTTCAAAGTGTCCTCGTAGGATGACCATGAGTGCAGGAAAATTTTCAAAGTCCCTCTGTCGGATGACCATGAGTGCAGAAAAAATTTCAAAGTCCCCCCTTGGGATTACCAGACGTTCGAGATTTCGGCTGAAAAATTTTCAAAGTGCTGCCGAGAGCCTGCGCTAGTTGCTTAAGGCTTGAGGAGATCCGCCTTATGGTAAGTAAACGAAAAGTGCCTGCGCCCCTGGAGGTTTTGGAAGGTGCGAGCGATGACCATGCTCGGGTTAGTAGGGAAGCTCATCGTCGAACCAGAGATGGGTAAGGGGCGAACTGGCAGATGTCTTCCCACCGTCGAGCAGCATTCCGGGCTTCACATCGGAGGGCTCCAGCCGGCCCCGGTTCCGAGAACCGGCGCGCGGAAGGTGGCGCCTCCGAACCCGAAGCCAGCCTCTAGGCACGGTCGCAAAGGTGACAGACGCCCCGCCGCCTGCCTCCACAGCACCGTGGCCGCCTCCGGGTGACGAGACTGAGGCGCCCCGTCCGTCTCAGAGGTCCAGAAACGGAGCCCGCCGCGGCGGGGACGCGCCTTCGAAAGCGTCCGCCGGCCCATCCGCGGAGGTGCCCTCCGGCGAGCACGTGCTTCTCAGGAGAGCCCGAGAGTCCGTTCACCCCTCCGGTCAAGATGATGCTTTGAGTGGGAGCCGAGCCGAGCGGGGCGGCTCCGGCGGGGAGGTTGGGAGGCGGCCGTTTGCCTTGCTGCAGCGGCCGTCGCCCCCGCCTGCCCCCCGGTCGCCGTCCCGAACGACTCCTCTTCCCCACTCTCCCAGCACCCACCCCCCCTGTCGGTGGCGGCCGGCTCCGGTGCTGGCGGTCGCGCCTCCGGGCGACCCGTCTGCAGCGCCCGGCCTTCTCCACGGGGACTACCTGGTTGATCCTGCCAGTAGCATATGCTTGTCTCAAAGATTAAGCCATGCAAGTCTAAGTACACACGGTCGGTACAGTGAAACTGCGAATGGCTCATTAAATCAGTTATGGTTCCTTTGATCGCTCCAACGTTACTTGGATAACTGTGGCAATTCTAGAGCTAATACATGCAAACGAGCGCTGACCTCCGGGGATGCGTGCATTTATCAGACCCAAGACCCTCGCGGGGATGCCTCTCGGGGCGCCCCGGTTGCTTTGGTGACTCTAGATAACCTCGAGCCGATCGCTGGCCCACCGTGGCGGCGACGTCTCATTCGAATGTCTGCCCTATCAACTTTCGATGGTACTTTAAGTGCCTACCATGGTGACCACGGGTAACGGGGAATCAGGGTTCGATTCCGGAGAGGGAGCCTGAGAAACGGCTACCACATCCAAGGAAGGCAGCAGGCGCGCAAATTACCCACTCCCGACTCGGGGAGGTAGTGACGAAAAATAACAATACAGGACTCTTTCGAGGCCCTGTAATTGGAATGAGTACACTTTAAATCCTTTAACGAGGATCTATTGGAGGGCAAGTCTGGTGCCAGCAGCCGCGGTAATTCCAGCTCCAATAGCGTATCTTAAAGTTGCTGCAGTTAAAAAGCTCGTAGTTGGATCTCGGGATCGAGCTGACGGTCCGCCGCGAGGCGAGCTACCGTCTGTCCCAGCCCCTGCCTCTCGGCGCCCCCTCGATGCTCTTAGCTGAGTGTCCCGCGGGGTCCGAAGCGTTTACTTTGAAAAAATTAGAGTGTTCAAAGCAGGCCCGGTCGCCTGAATACCGCAGCTAGGAATAATGGAATAGGACTCCGGTTCTATTTTGTGGGTTTTCTCTGAACTGGGGCCATGATTAAGAGGGACGGCCGGGGGCATTCGTATTGTGCCGCTAGAGGTGAAATTCTTGGACCGGCGCAAGACGGACGAAAGCGAAAGCATTTGCCAAGAATGTTTTCATTAATCAAGAACGAAAGTCGGAGGTTCGAAGACGATCAGATACCGTCGTAGTTCCGACCATAAACGATGCCAACTAGCGATCCGGCGGCGTTATTCCCATGACCCGCCGGGCAGCGTCCGGGAAACCAAAGTCTTTGGGTTCCGGGGGGAGTATGGTTGCAAAGCTGAAACTTAAAGGAATTGACGGAAGGGCACCACCAGGAGTGGAGCCTGCGGCTTAATTTGACTCAACACGGGAAACCTCACCCGGCCCGGACACGGAAAGGATTGACAGATTGATAGCTCTTTCTCGATTCTGTGGGTGGTGGTGCATGGCCGTTCTTAGTTGGTGGAGCGATTTGTCTGGTTAATTCCGATAACGAACGAGACTCCGGCATGCTAACTAGTTACGCGGCCCCGTGTGGTCGGCGTCCAACTTCTTAGAGGGACAAGTGGCGTTCAGCCACACGAGATTGAGCAATAACAGGTCTGTGATGCCCTTAGATGTCCGGGGCTGCACGCGCGCCACACTGAGTGGATCAGCGTGTGTCTACCCTTCGCCGAGAGGCGTGGGTAACCCGCTGAACCCCACTCGTGATAGGGATTGGGGATTGCAATTATTTCCCATCAACGAGGAATTCCCAGTAAGCGCGGGTCATAAGCTCGCGTTGATTAAGTCCCTGCCCTTTGTACACACCGCCCGTCGCTACTACCGATTGGATGGTTTAGTGAGGTCCTCGGATCGGCCCCGCCGGGGTCGGCCACGGCCCTGGCGGAGCGCCGAGAAGACGATCAAACTTGACTATCTAGAGGAAGTAAAAGTCGTAACAAGGTTTCCGTAGGTGAACCTGCGGAAGGATCATTACCGGTTTCGTCCCAAGTCTGGTGGCCGCAAACACGCTCCAAGCCCCGGGAGGACGGGCTGGTGGAGGGGCGTCGGAGCGGCGGGCCAACCCCACCGGCGACGGTGCGCGTCCGGGAGAGGGACCGGGAGGCGTCACGGCCTCCCCCTCTCTCCCGAGGCGACTCTGCGCGTCGGTGAGGACCTGGTACCCGTCGCTGCGCTCCGCCCCTCCACCTATCACCACCCGCCCTCCCGAGGCTCCAAGGGCGGCAGGGTGCCGCCGGGCTTCCGCCGTGCCCCGTACGCCCTCGACCTGCTCGGCCTTCGGGCCGGGGAGGCTGGGATGCGGGACACAACGGCGCGGTCGTCCCGACCCCCCTGCCGTCTGTCCGAAAGCGCCGGAGGCACGCCGAGCCGACCCGACTCCGTGCGCCCGTAGCTCGCCGAACCCCCGTTACCCTGTGCGCCCCGTCGGTCCGAAACTGCACCGCACCTATATAGCGACCCCCACCCTAGACAGGGGGGGTCGTGGTGACGGGGCTGCGGACGGCCGGCGGGACCGGGGTTACGGCTGGGAAGGGAGGTGCGGGACGCGGAGAGGCCCGGCGTGTGCCTCGCGCCGAGCCAAACTCCGTGCGCCCGTAGCTCGCCGAACCCCCCGTTACCCTGTGCGCCCCGTCGGTCCGAAGCTGCCCAGCACCTATATAGCGACCCCCACCCTAGACAGGGGGGGTCGTGGTGACCGGGCTGTGGACGGCCGGCGGGACCGGGGTTACGGGGGGGGGAAGGGAGGTGCGGGACGCGGAGAGGCCCGGCGCGTGCTCCGAGCCAAACTCCGTACGCCCGTAGCTCGCTGCCCCCCGTTACACTGTGCGCCCCGTCGGTCCGAAGCTGCCCAGCACCTATATAGCGACCCCCACCCTAGACAGGGGGGGGTCGTGGTGACCGGGTTGTGGACGGCCGGCGGGACCGGGGTTACGGGGGACGGAGGTGCGGGACGCGGAAGAGGCCCGGTGCGCTCCTCCGACGCCCTAGGACCCTCGAACCTCCTAGTCCGGGCCCGGCTTCCCCGCCGACAGGTGCGTTCCCTTCCCCCGGCTCTCTCTCCTTTCCTCCGTCAGCGCGACGTCCCGTCGGGGTTCGACCCGAGGGCTGACGGGCCGCAGGCCCGGCGGGCGGCGCGTGGAGGAATCACCAAGGGGAGAGGGTCCTCGTGTGGGGACGGGTGCTCGCCACGTCGACGGACCGAACGGACCGCGGCCCGACCCTCGGAACACACTGACCAGCACGGCGCGTCGGCCTCGCCCTGGCCGCGTGCCGTGTGCCGCTCGGGTACCCCGCAAGGGGTTCAAAGCCTCCCCGGAGCGCCCGGGCGGTCTACTCTGTAAACCCCAGGTTCTCTGATCCAGTCGACCCACAAACAAAAAAACTGGACAACTCTTAGCGGTGGATCACTCGGCTCGTGCGTCGATGAAGAACGCAGCTAGCTGCGAGAACTAATGTGAATTGCAGGACACATTGATCATCGACACTTCGAACGCACCTTGCGGCCCCGGGTTCCTCCCGGGGCTACGCCTGTCTGAGGGTCGCTTTCCAAATCAATCGGGAGAGGCCTCCTCTCCCGCGGTTGGGGCTGTCGCAGGCCTCGGTCGACTCACGCCGACCAGGGCCTTCGTCCCCCTAAGTGCAGACTGCTGGATGCCCGTCGCGACGGACCCACCTCGGGCCCGGCGCTGCCGCCGTCCTCCGGTTCTCCCGACACAGCCGTCGTCCCTCCTCCGTTTCCCCACCTCCGACGCTCCTCCGCGGGCGCCGGTGGACCGGGGGCGCGGAGGGGGCGGCCGTCTCCGCCGAGCCCCGCACGGTTGCGGGCGCGGCTGCCGGTGCGGACACTCTCTCGAGAGGTCTCATCCGAGCTGCCCGCGTCCGTGCCGCGCGCCCAGGGGCTCACACGGCGGAGGCGGACGCCTCCAGCGGGGGACGGCGGTAGGGAGGCTCGGCCCGGACGACGCGCCGGCGTCGGACCCGAGCTCGGACGTCCGCCGCGGCGGGGTACCCGCCCTGAACTGAGCCGGCGAGCCTCCGCCACCCCCCCTCTCTCCTCGGAGTGTGGGGGGGGGCGCGGAGCCGCACCCTTGCCATCCCATCGGCCCCACCCCGACGCCCACCACCGGTGGGAAGACGGGGGGGGACGTTGGGGGGGGCAGCAGCATCCGACTACGACCTCAGATCAGACGAGACAACCCGTGAATTTAAGCATATTACTAAGCGGAGGAAAAGAAACTAACAAGGATTCCCTCAGTAGCGGCGAGCGAAGAGGGAAGAGCCCAGCGCCGAATCCCCGTCCGACTGGCGGGCGTGGGAAATGTGGCGTACAGAAGACCGCCTGCCCGGTGTCGCTCGGGGGCCTGAGTCCTCCTGATCGAGGCTCATCCCATGGACGGTGTGAGGCCGGTAACGGCCCCCGTCGCGCCGGGGCTCGGTCTTCTCGGAGTCGGGTTGTTTGGGAATGCAGCCCAAAGCGGGTGGTAAACTCCATCTAAGGCTAAATACCGGCACGAGACCGATAGTCGACAAGTACCTTAAGGGAAAGTTGAAAAGAACTTTGAAGAGAGAGTTCAAGAGGGCGTGAAACCGTTAAGAGGTAAACGGGTGGGGTCCGCGCAGTCCGCCCGGGGGATTCAACTCGGCAGGTCAGGGACGGCCGCTCGGCGCGGGAGGATCCCCTCCGTGGGAACTCCCCGCCGGTTGGCTGGCCCCCGCCGGGCGCATTTCCTCCGCCGGTGGTGCGCCGCGACCGACTCTGGATCGGCCAGGAAGGGCTCGGGGCGAAGGTGGCTCGCGGCTCCGGCCGCGAGCTTTACAGCGACCCAACGCCTGGACCTCGCCGCTTTCCGGGGTCGTGGAATCAGTACTCACTGCGCCTTCTCTCCTCCGCCTCGCGCCTCCGTCCCCCTCCTCGTGGGGGGGGGCGGGGGACTGGGCGGCCCACGGGAGGGACGGGGCCCCCTCGCCCCCGGCGCGACTGTCGACCGGAGCGGACTGTTCTCAGTGCGCTCCGACCGCGTCGCGCCGCCCGGGCGGGGACCGGCTCACGTACACAGGGCGCAAGGGGTCTGCGGCGATGTCGGCTACCCACCCGACCCGTCTTGAAACACGGACCAAGGAGTCTAACGCACGCGCGAGTCAGAGGGTCCTACTCGAAACCCCGTGGCGCAATGAAAGTGAAGGCCGGCGCGCGCCGGCCGAGGTGGGATCCCGGGCCCCTCGCGGTTCCCGGGCGCACCACCGGCCCGTCTCGCCCGCTCCGTCGGGGAGGTGGAGCTAGAGCGCGTGCGATAGGACCCGAAAGATGGTGAACTATGCCTGGGCAGGGCGAAGCCAGAGGAAACTCTGGTGGAGGCCCGTAGCGGTCCTGACGTGCAAATCGGTCGTCCGACCTGGGTATAGGGGCGAAAGACTAATCGAACCATCTAGTAGCTGGTTCCTTCCGAAGTATCCCTCAGGACAGCTGGCGCTCAGAGTCTCGCAGTTTTATCTGGTAAAGCGAATGATTAGAGGTCTTGGGGCCGAAACGATCTCAACCTATTCTCAAACTTTAAATGGGTAAGAAGCCCGGCTCGCTGGCATGGAGCCGGGCGTGGAATGCGAGCCGCCCAGTGGGCCACTTTTGGTAAGCAGAACTGGCGCTGCGGGATGAACCGAACGCCGGGTTAAGGCGCCCGATGCCGACGCTCATCAGACCCCAGAAAAGGTGTTGGTTGATATAGACAGCAGGACGGTGGCCATGGAAGTCGGAATCCGCTAAGGAGTGTGTAACAACTCACCTGCCGAATCAACTAGCCCTGAAAATGGATGGCGCTGGAGCGTCGGGCCCATACCCGGCCGTCGCCGGCAGCAGGAGCCGCGAGGGCTATGCCGCGACGAGTAGGAAGGCCGCCGCGGTGAGCACGGAAGCCTAGGGCGCGAGCCCGGGTGGAGCCGCCGCGGGTGCAGATCTTGGTGGTAGTAGCAAATATTCAAACGAGAACTTTGAAGGCCGAAGTGGAGAAGGGTTCCATGTGAACAGCAGTTGAACATGGGTCAGTCGGTCCTAAGGGATGGGCGAACGCCGTTCGGAAGCGCGGGGCGATGGCCTACGTCGCCCCCCCGGCCGATCGAAAGGGAGTCGGGTTCAGATCCCCGAACCTGGAGTGGCGGAGACAGGCGCCGCGAGGCGTCCAGTGCGGTAACGCAAACGAACTCGGAGAAGCTGGCGGGAGCCCCGGGGAGAGTTCTCTTTTCTTTGTGAAGGGCAGGGCGCCCTGGAATGGGTTCGCCCCGAGAGAGGGGCCCGTGCCCTGGAAAGCGTCGCGGTTCCGGCGGCGTCCGGTGAGCTCTCGCTGGCCCTTGAAAATCCGAGGGAGAAGGTGTAAATCTCGCGCCAGGCCGTACCCATATCCGCAGCAGGTCTCCAAGGTGAACAGCCTCTGGCGTCTTAGAAGAAGGGAGTGTAAGGGAAGTCGGCAAGTCAGATCCGAAACTTCGGGATAAGGATTGGCTCAAAGGGCTGGGTCGGTCGGGCTGGGGTGCGAAGCGAGGCTGGGCTCGTGCCGCGGCTGGGGGAGCAGTCGCCCCGTCGCCCTCCCCTCTCCGCCGCCTTGAAGCCCGGTTGCCGGCCCGGCTCGTGGTGGGGCCCCCTTCGTCCGTCGCGCCTCGCGCGTCGGCGGGCGGTGGGAGTCTTTGCTGCGAGCCGGTGTCCGACGCCGGGTGGATGGCGGGTCGTGGGAGGAGATGCGGTCGGCGGGTGCGGCGGCGACTCTGGACGCGCGCCGGGCCCTTCTCGCGGATCTCCCCAGCTGCGGCGCCCTTGGGGTGGGTGTCGTCCGTTCACGCGGGCGGCCCTGCCCCTCGGGTTGCCTCGGCTGGCGCCTAGCAGCTGACTTTGAACTGGTGCGGACCAGGGGAATCCGACTGTTTAATTAAAACAAAGCATCGCGAAGGCCCACGGGGGGTGTTGACGCGATGTGATTTCTGCCCAGTGCTCTGAATGTCAAAGTGAAGAAATTCAATGAAGCGCGGGTAAACGGCGGGAGTAACTATGACTCTCTTAAGGTAGCCAAATGCCTCGTCATCTAATTAGTGACGCGCATGAATGGATGAACGAGATTCCCACTGTCCCTACCTCCTATCTAGCGAAACCACAGCCAAGGGAACGGGCTTGGCAGAATCAGCGGGGAAAGAAGACCCTGTTGAGCTTGACTCTAGTCTGGCACCGTGAAGAGACATGAGAGGTGTAGAATAAGTGGGAGGCCTCACGGTCGACGGTGAAATACCACTACTCTTATCGTTTTTTCACTTACCCGGTGAGGCGGGGAGGCGAGCCCCGAGTGGGCTCTCGGTTCTGGTGTCAAGCGCCCGGCGCGTGCCGGGCGTGACCCGCTCCGGGGAAAGTGGCAGGTGGGGAGTTTGACTGGGGCGGTACACCTGTCAAACTGTAACGCAGGTGTCCTAAGGCGAGCTCAGGGAGGACAGAAACCTCCCGTGGAGCAGAAGGGCAAAAGCTCGCTTGATCTTGATTTTCAGTATGAATACAGACCGTGAAAGCGGGGCCTCACGATCCTTCTGACTTTTTGGGTTTTAAGCAGGAGGTGTCAGAAAAGTTACCACAGGGATAACTGGCTTGTGGCGGCCAAGCGTTCATAGCGACGTCGCTTTTTGATCCTTCGATGTCGGCTCTTCCTATCATTGTGAAGCAGAATTCACCAAGCGTTGGATTGTTCACCCACTAATAGGGAACGTGAGCTGGGTTTAGACCGTCGTGAGACAGGTTAGTTTTACCCTACTGATGATGTGTTGTTGCAATAGTAATCCTGCTCAGTACGAGAGGAACCGCAGGTTCAGACATTTGGTGTATGTGCTTGGCTGAGGAGCCAATGGGGCGAAGCTACCATCTGTGGGATTATGACTGAACGCCTCTAAGTCAGAATCCCGCCTAGACGTAATGATACCGTAGCGCCGCGAATCTTCGGTTGGTCCCGGATAGCTGGCCCTCGGGCCGGTGCGGAGAGCCGTTCGTGACTGGGCTGGGGTGCGGCCGAATGATGGCTGCCCCTCTCCAATTGCGCACTGCACGTTTGTGGAGAACGTGGTGCTAAATGACTTGCAGACGACCTGATTCTGGGTCAGGGTTTCGTGCGTGGCAGAGCAGCTACCTCGCTGCGATCCATTGAAAGTCAGCCCTCGATCCAAGTTTTTGTCGGGGTCCTAGCCCCCGTACCTCCCACCCTCCTCCGCATCCACCAAACGGGAAGACCAGTCGCGGAGGTGGGTGGAACTCGGTGGCCCAGCAATGCAACCCCCGGACCTCCGGGGCCGGTCCCAAGTCCGGATCAATGCAGAGGGATGAGCCACTGCCTGAAGCCGAGGTGTCAGAAATTTTCTAAGTGTTGAACTTTTTCTAAGTGTCAGCACGCAGGAGCTGGAAATTTTCTAAGTGTTGAACTTTTTCTAAGTGTCAGCACGCAGGAGCTGAAAATTTTCTAAGTGTTGAACTTTTTCTAAGTGTCAGCACGCAGGAGCTGGAAATTTTCTAAGTGTTGAACTTTTTCTAAGTGTTGAACTTTTTCTAAGTGTCAGCACGCAGGAGCTGGAAATTTTCTAAGTGTTACTTAGGATTACCAGTGTGCGAAAATAATTTCTAAGTGTTGAACTTTTTCTAAGTGTCAGCACACAGAAGCTGGAAATTTTCTAAGTGTTAATTTGGATTACCAGTGTGCGAAAATATTTTTCTAAGTGTTACTTAGGATGACCAGACGTACGAAATTGGATTTGGATGACCAGGCTGCTGGAGGTCCAGCCGGCGTGGACTAGGGTCTTTAACCCAGGGGAGGGTGCTTAATAGTGGGCCGCAG

**(D) colnames.txt**

**(D-1) testis>06_count> colnames.txt**

geneID, GeneLength, G5w_1, G5w_2, G5w_3, G8w_1, G8w_2, G8w_3, G10w_1, G10w_2, G10w_3, G11w_1, G11w_2, G11w_3, S3w_1, S3w_3, S5w_1, S5w_2, S5w_3, S8w_1, S8w_2, S8w_3, S11w_1, S11w_2, S11w_3

**(D-2) ovary>06_count> colnames.txt**

geneID, GeneLength, G5w_1, G5w_2, G5w_3, G8w_1, G8w_2, G8w_3, G10w_1, G10w_2, G10w_3, G11w_1, G11w_2, G11w_3, S3w_1, S3w_2, S3w_3, S5w_1, S5w_2, S5w_3, S8w_1, S8w_2, S8w_3, S11w_1, S11w_2, S11w_3

**(E) samplenames.txt**

**(E-1) testis>06_count> samplenames.txt**

G5w,G5w,G5w,G8w,G8w,G8w,G10w,G10w,G10w,G11w,G11w,G11w,S3w,S3w,S5w,S5w,S5w,S8w,S8w,S8w,S11w,S11w,S11w

**(E-2) ovary>06_count> samplenames.txt**

G5w,G5w,G5w,G8w,G8w,G8w,G10w,G10w,G10w,G11w,G11w,G11w,S3w,S3w,S3w,S5w,S5w,S5w,S8w,S8w,S8w,S11w,S11w,S11w

**(F)spermatogenesis.csv**

ENSNFUG00015024780

ENSNFUG00015024090

ENSNFUG00015022180

ENSNFUG00015018707

**(G)** **ovaryGOterms-nfu.txt**

ENSNFUG00015024780

ENSNFUG00015019763

ENSNFUG00015021052

ENSNFUG00015001640

ENSNFUG00015021955

ENSNFUG00015009599

ENSNFUG00015017953

ENSNFUG00015012154

ENSNFUG00015018013

ENSNFUG00015001916

ENSNFUG00015008988

ENSNFUG00015018207

ENSNFUG00015012265

ENSNFUG00015012937

ENSNFUG00015024259

ENSNFUG00015006897

ENSNFUG00015017788

ENSNFUG00015005163

ENSNFUG00015021048

ENSNFUG00015007607

ENSNFUG00015023830

ENSNFUG00015009294

ENSNFUG00015007513

ENSNFUG00015008412

ENSNFUG00015005352

**(H) R scripts**

**(H-1) logPlot.R**

library(reshape2)

library(ggplot2)

comm <- commandArgs(trailingOnly = T);

infile <- comm[1];

outfile <- comm[2];

d1 = read.table(infile, header = T, sep="\t", quote="", comment.char="")

d2 = melt(d1, id.vars = "library")

g = ggplot(d2, aes( x = factor(library), y = value, fill = variable,))

g = g + geom_bar( stat = "identity") + xlab("Sample Name") + ylab("number of read-pairs") + labs(fill = "Category")

pdf(paste(outfile,".pdf", sep = ""))

plot(g)

dev.off()

**(H-2) counts_to_tpm2.R**

# calculating TPM

comm <- commandArgs(trailingOnly = T);

infile   <- comm[1];

outdir <- comm[2];

out_f <- paste(outdir, "TPMfeaturecounts.txt", sep="/");

colname <- comm[3];

colname <-read.table(colname, header=F, sep=",")

data <- read.table(infile, header=TRUE, row.names=1, sep="\t", quote="")

ncol(data)

head(data)

head(data[,6:ncol(data)])

sum(data[,6:ncol(data)])

nf_RPK <- 1000/data[,5]

RPK <- data[,6:ncol(data)] * nf_RPK

head(RPK)

TPM <- sweep(RPK * 1000000, 2,colSums(RPK),"/")

head(TPM)

tmp <- cbind(rownames(data), data[,5], TPM)

colnames(tmp)<- colname

write.table(tmp, out_f, sep="\t", append=F, quote=F, row.names=F)

**(H-3) TH.R**

#threshold of TPM value

args <- commandArgs(T)

data <- args[1];

outdir <- args[2];

colname <- args[3];

colname <-read.table(colname, header=F, sep=",")

data <- read.delim(data, header=T, sep="\t");

out_f <- paste(outdir, "TPMfeaturecounts_max5.txt", sep="/");

data1 <-data[,3:ncol(data)]

data1 <- data[(apply(data1,1,max,na.rm=F) >= 5),]

colnames(data1)<- colname

write.table(data1, out_f, sep="\t", append=F, quote=F, row.names=F, col.names=T)

out_f <- paste(outdir, "TPMfeaturecounts_max10.txt", sep="/");

data2 <-data[,3:ncol(data)]

data2 <- data[(apply(data2,1,max,na.rm=F) >= 10),]

colnames(data2)<- colname

write.table(data2, out_f, sep="\t", append=F, quote=F, row.names=F, col.names=T)

out_f <- paste(outdir, "TPMfeaturecounts_min5.txt", sep="/");

data1 <-data[,3:ncol(data)]

data1 <- data[(apply(data1,1,min,na.rm=F) >= 5),]

colnames(data1)<- colname

write.table(data1, out_f, sep="\t", append=F, quote=F, row.names=F, col.names=T)

out_f <- paste(outdir, "TPMfeaturecounts_min10.txt", sep="/");

data1 <-data[,3:ncol(data)]

data1 <- data[(apply(data1,1,min,na.rm=F) >= 10),]

colnames(data1)<- colname

write.table(data1, out_f, sep="\t", append=F, quote=F, row.names=F, col.names=T)

**(H-4) Fig5A-testis.R**

#clustering

library(lattice)

library(latticeExtra)

library(gplots)

library(matrixStats)

library(genefilter)

library(dplyr)

my.col1 <- colorRampPalette(c("blue","white","magenta"))

comm <- commandArgs(trailingOnly = T);

infile1 <- comm[1];

outdir <- comm[2];

data<-read.delim(infile1,header=T, sep="\t")

x<-data[,3:25]

x<-x+1

x <- as.matrix(x)

m1<-rowMeans(x[,1:3])

m2<-rowMeans(x[,4:6])

m3<-rowMeans(x[,7:9])

m4<-rowMeans(x[,10:12])

m5<-rowMeans(x[,13:14])

m6<-rowMeans(x[,15:17])

m7<-rowMeans(x[,18:20])

m8<-rowMeans(x[,21:23])

mx<-cbind(m1,m2,m3,m4,m5,m6,m7,m8)

mx_a<-mx/rowMeans(mx)

log_mx_a<-log(mx_a, base=2)

outfile <- paste(outdir, "Fig5A-testis.pdf", sep="/");

pdf(file=outfile,onefile=FALSE,paper="special",height=15,width=15,family="Helvetica",pointsize=20)

heatmap.2(as.matrix(log_mx_a),col=my.col1(269),scale="none",key=TRUE, symm=F,symkey=F,symbreaks=T, breaks=c(seq(-1,1,length=270)), trace="none",distfun = function(x) {dist(x, method="euclidean")},hclustfun = function(x) {hclust(x, method="ward.D2")})

dev.off();

**(H-5) Fig5A-ovary.R**

#ovary-clustering

library(lattice)

library(latticeExtra)

library(gplots)

library(matrixStats)

library(genefilter)

library(dplyr)

my.col1 <- colorRampPalette(c("blue","white","magenta"))

comm <- commandArgs(trailingOnly = T);

infile1 <- comm[1];

outdir <- comm[2];

indata <- read.delim(infile1, header=T, sep="\t");

x<-indata[,3:26]

x<-x+1

x <- as.matrix(x)

m1<-rowMeans(x[,1:3])

m2<-rowMeans(x[,4:6])

m3<-rowMeans(x[,7:9])

m4<-rowMeans(x[,10:12])

m5<-rowMeans(x[,13:15])

m6<-rowMeans(x[,16:18])

m7<-rowMeans(x[,19:21])

m8<-rowMeans(x[,22:24])

mx<-cbind(m1,m2,m3,m4,m5,m6,m7,m8)

mx_a<-mx/rowMeans(mx)

log_mx_a<-log(mx_a, base=2)

outfile <- paste(outdir, "Fig5A-ovary.pdf", sep="/");

pdf(file=outfile,onefile=FALSE,paper="special",height=15,width=15,family="Helvetica",pointsize=20)

heatmap.2(as.matrix(log_mx_a),col=my.col1(269),scale="none",key=TRUE, symm=F,symkey=F,symbreaks=T, breaks=c(seq(-1,1,length=270)), trace="none",distfun = function(x) {dist(x, method="euclidean")},hclustfun = function(x) {hclust(x, method="ward.D2")})

dev.off();

**(H-6) DESeq2_M.R**

#PCA(VST)&DESeq2

options(warn=1)

options(scipen=100)

options( java.parameters = "-Xmx64g" )

library( DESeq2 )

packageVersion("DESeq2")

library(xlsx)

library("genefilter")

library("gplots")

my.col1 <- colorRampPalette(c("blue","white","magenta"))

args <- commandArgs(T)

workDir   <- args[1]

data      <- args[2]

name    <- args[3]

gtfFile   <- args[4]

gene_type <- args[5]

subName   <- ifelse(gene_type=="default","",paste0(".",gene_type))

markers   <- c()

TPMdata <- args[6]

newTPMdata<-args[7]

THtpm <- args[8]

infile3 <- args[9];

outdir <- workDir

outDir    <- file.path(workDir)

outFile   <- file.path(outDir, paste0("TPM",".xlsx"))

outPCA    <- file.path(outDir, paste0("PCA",subName,".xlsx"))

outBAR    <- file.path(outDir, paste0("barplot",subName,".pdf"))

saveData  <- file.path(outDir, paste0("DESeq2_HISAT2",subName,".Rdata"))

name1 <- read.delim(name,header=F, sep=",")

name1<-as.vector(name1)

Group <- data.frame(con = factor(name1))

#TPM>10

data<-read.table(data,header=T,row.names=1,sep="\t")

TPMdata<-read.delim(TPMdata,header=T,sep="\t")

TPMdata1<-as.matrix(TPMdata)

TPMmax10<-TPMdata1[,1]

print(head(TPMdata1))

print(head(TPMmax10))

L1 <- length(TPMmax10)

L1

t5 <- c()

t4 <- subset (data, row.names(data) == TPMmax10[1])

t4 <- t4

for (i in 2:L1) {

  t5 <- subset (data, row.names(data) ==  TPMmax10[i], )

  t4 <- rbind (t4, t5)

}

data<-t4

data1<-row.names(data)

data2<-data[6:ncol(data)]

data3<-cbind(data1, data2)

#DESeq2

dds <- DESeqDataSetFromMatrix(countData=data2, colData=Group, design=~ con)

genelength <- matrix( unlist(data [5]), ncol = 1)

mcols(dds)$basepairs <- as.numeric(genelength)

dds <- estimateSizeFactors(dds)

dds <- estimateDispersions(dds)

dds <- nbinomWaldTest(dds)

#vst_value_PCA_plot(Fig.5B)________________________

vst <- vst(dds, blind=FALSE)

head(assay(vst), 3)

res <- prcomp(t(assay(vst)), scale=T)

v <- res$sdev^2

v <- v / sum(v)

v[1]<-round(v[1]*100,1)

v[2]<-round(v[2]*100,1)

v[3]<-round(v[3]*100,1)

v[4]<-round(v[4]*100,1)

v[5]<-round(v[5]*100,1)

v[6]<-round(v[6]*100,1)

v[7]<-round(v[7]*100,1)

v[8]<-round(v[8]*100,1)

v[9]<-round(v[9]*100,1)

v[1]

v[2]

v[3]

v[4]

v[5]

PC1 <- res$x[, 1]

PC2 <- res$x[, 2]

PC3 <- res$x[, 3]

PC4 <- res$x[, 4]

PC5 <- res$x[, 5]

PC6 <- res$x[, 6]

PC7 <- res$x[, 7]

PC8 <- res$x[, 8]

PC9 <- res$x[, 9]

PC1_9<- res$x[,1:9]

outfile <- paste(outdir, "Score_VST_PCA_prcomp.txt", sep="/")

write.table(PC1_9, file=outfile, sep="\t")

col <- c("skyblue3","skyblue3","skyblue3","skyblue3","skyblue3","skyblue3","skyblue3","skyblue3","skyblue3","skyblue3","skyblue3","skyblue3","palevioletred1","palevioletred1","palevioletred1","palevioletred1","palevioletred1","palevioletred1","palevioletred1","palevioletred1","palevioletred1","palevioletred1","palevioletred1")

pch <- c(0, 0, 0, 0, 0, 0, 0, 0, 0, 0, 0, 0,0, 0, 0, 0, 0, 0, 0, 0, 0, 0, 0, 0, 1, 1, 1, 1, 1, 1, 1, 1, 1, 1, 1, 1, 1, 1, 1, 1, 1, 1, 1, 1, 1, 1, 1, 1)

label3 <- c("I", "I", "I", "II", "II", "II", "III", "III","III", "IV", "IV", "IV", "I", "I", "II", "II", "II", "III", "III", "III", "IV", "IV", "IV")

outfile <- paste(outdir, "VST_PCA_prcomp_PC1-2_Fig.5B.pdf", sep="/");

pdf(file=outfile,onefile=FALSE,paper="special",height=15,width=15,family="Times",pointsize=20)

xname <- paste("PC1_", v[1], "%", sep="");

yname <- paste("PC2_", v[2], "%", sep="");

plot(PC1, PC2, pch=1, cex=6, col = col,xlab=xname, ylab=yname, main="VSA_PCA_prcomp_PC1-PC2")

text(PC1, PC2, label3, col = col, cex=2.5)

dev.off();

#loading factor

fc.l2<-sweep(res$rotation, MARGIN=2, res$sdev, FUN="*")

newTPMdata<-read.delim(newTPMdata,header=T,sep="\t")

colname <- newTPMdata[,1];

colname <- as.matrix(colname)

nfu_G <- data3[,1]

nfu_zeb<-read.delim(infile3, sep="\t",header=F);

nfu_zeb <- as.matrix(nfu_zeb)

L1 <- length(nfu_G)

L2 <- L1+1

L2

t5 <- c()

t4 <- subset (nfu_zeb, nfu_zeb[,1] == nfu_G[1], 4)

t4 <- t4[1]

for (i in 2:L1) {

  t5 <- subset (nfu_zeb, nfu_zeb[,1] ==  nfu_G[i], 4)

  t6 <- t5[1]

  t4 <- c (t4, t6)

}

zebraID <- t4

zebraID <- as.matrix(zebraID)

colnames(zebraID)<-c("zebra_geneID")

length(zebraID)

t5 <- c()

t4 <- subset (nfu_zeb, nfu_zeb[,1] == nfu_G[1], 5)

t4 <- t4[1]

for (i in 2:L1) {

  t5 <- subset (nfu_zeb, nfu_zeb[,1] ==  nfu_G[i], 5)

  t6 <- t5[1]

  t4 <- c (t4, t6)

}

zebraGName <- t4

zebraGName <- as.matrix(zebraGName)

colnames(zebraGName)<-c("zebra_geneName")

t5 <- c()

t4 <- subset (nfu_zeb, nfu_zeb[,1] == nfu_G[1], 6)

t4 <- t4[1]

for (i in 2:L1) {

  t5 <- subset (nfu_zeb, nfu_zeb[,1] ==  nfu_G[i], 6)

  t6 <- t5[1]

  t4 <- c (t4, t6)

}

zebraGene <- t4

zebraGene <- as.matrix(zebraGene)

colnames(zebraGene)<-c("zebra_gene")

fc.l<-cbind(data3,zebraID,zebraGName,zebraGene,fc.l2)

outfile <- paste(outdir, "FactorLoadings_VST_PCA_prcomp.txt", sep="/")

write.table(fc.l, file=outfile, sep="\t")

#______________________

#DEGs(For Supplementary Fig.2)

norm.dt   <- data.frame(counts(dds, normalized=T), check.names=F)

fpkm.dt   <- data.frame(fpkm(dds, robust=F), check.names=F)

tpm.dt    <- data.frame(sweep(fpkm.dt*1e6, 2, colSums(fpkm.dt),"/"), check.names=F)

cpm.dt    <- data.frame(fpm(dds, robust=F), check.names=F)

outfile <- paste(outDir, "DESeq2_results.Rdata", sep="/")

wb <- createWorkbook(type="xlsx")

sheet  <- createSheet(wb, sheet="TPM")

row.names(tpm.dt)<-row.names(data)

colnames(tpm.dt)<-name1

addDataFrame(tpm.dt, sheet, row.names=T)

saveWorkbook(wb, file=outFile)

outfile <- paste(outDir, "TPM.txt", sep="/")

write.table(tpm.dt, file=outfile, row.names=T)

dds <- DESeqDataSetFromMatrix(countData=data2, colData=Group, design=~ con)

genelength <- matrix( unlist(data [5]), ncol = 1)

mcols(dds)$basepairs <- as.numeric(genelength)

dds <- DESeq(dds)

terms<-c("S11w","S8w","S5w","S3w","G11w","G10w","G8w","G5w")

terms2<-combn(terms,2)

terms2

n<-ncol(terms2)

newTPMdata<-as.matrix(newTPMdata)

L1 <- length(TPMmax10)

L1

t5 <- c()

t4 <- subset (newTPMdata, newTPMdata[,1] == TPMmax10[1])

t4 <- t4

for (i in 2:L1) {

  t5 <- subset (newTPMdata, newTPMdata[,1] ==  TPMmax10[i], )

  t4 <- rbind (t4, t5)

}

newTPMdata<-t4

FC<-log2(1.5)

print(FC)

for (j in 1:n) {

  terms3<-terms2[,j]

  terms4<-append("con",terms3)

  print(terms4)

  res<-results(dds, contrast=terms4)

  res$TPM <-newTPMdata

  tpm<-THtpm

  print(tpm)

  data2 <- as.data.frame(res)

  data2upAll <- subset(data2, (data2$pad<=0.01 & (data2$log2FoldChange>=FC )))

  outfilename <- paste0("DESeq2_result_", terms3[1],"vs",terms3[2],"_TPMmax10_1.5upAll.txt")

  out_f <- paste(outDir, outfilename, sep="/");

  write.table(data2upAll, out_f, sep="\t", append=F, quote=F, row.names=T, col.names=T)

  data2downAll <- subset(data2, (data2$pad<=0.01 & (data2$log2FoldChange<=-FC )))

  outfilename <- paste0("DESeq2_result_", terms3[1],"vs",terms3[2],"_TPMmax10_1.5downAll.txt")

  out_f <- paste(outDir, outfilename, sep="/");

  write.table(data2downAll, out_f, sep="\t", append=F, quote=F, row.names=T, col.names=T)

}

**(H-7) DESeq2_F.R**

#PCA(VST)&DESeq2

options(warn=1)

options(scipen=100)

options( java.parameters = "-Xmx64g" )

library( DESeq2 )

packageVersion("DESeq2")

library(xlsx)

library("genefilter")

library("gplots")

my.col1 <- colorRampPalette(c("blue","white","magenta"))

args <- commandArgs(T)

workDir   <- args[1]

data      <- args[2]

name    <- args[3]

gtfFile   <- args[4]

gene_type <- args[5]

subName   <- ifelse(gene_type=="default","",paste0(".",gene_type))

markers   <- c()

TPMdata <- args[6]

newTPMdata<-args[7]

THtpm <- args[8]

infile3 <- args[9];

outdir <- workDir

outDir    <- file.path(workDir)

outFile   <- file.path(outDir, paste0("TPM",".xlsx"))

outPCA    <- file.path(outDir, paste0("PCA",subName,".xlsx"))

outBAR    <- file.path(outDir, paste0("barplot",subName,".pdf"))

saveData  <- file.path(outDir, paste0("DESeq2_HISAT2",subName,".Rdata"))

name1 <- read.delim(name,header=F, sep=",")

name1<-as.vector(name1)

Group <- data.frame(con = factor(name1))

data<-read.table(data,header=T,row.names=1,sep="\t")

#TPM>10

TPMdata<-read.delim(TPMdata,header=T,sep="\t")

TPMdata1<-as.matrix(TPMdata)

TPMmax10<-TPMdata1[,1]

print(head(TPMdata1))

print(head(TPMmax10))

L1 <- length(TPMmax10)

L1

t5 <- c()

t4 <- subset (data, row.names(data) == TPMmax10[1])

t4 <- t4

for (i in 2:L1) {

  t5 <- subset (data, row.names(data) ==  TPMmax10[i], )

  t4 <- rbind (t4, t5)

}

data<-t4

data1<-row.names(data)

data2<-data[6:ncol(data)]

data3<-cbind(data1, data2)

#DEseq2

dds <- DESeqDataSetFromMatrix(countData=data2, colData=Group, design=~ con)

genelength <- matrix( unlist(data [5]), ncol = 1)

mcols(dds)$basepairs <- as.numeric(genelength)

dds <- estimateSizeFactors(dds)

dds <- estimateDispersions(dds)

dds <- nbinomWaldTest(dds)

#vst_value_PCA_plot(Fig.5B)________________________

vst <- vst(dds, blind=FALSE)

head(assay(vst), 3)

res <- prcomp(t(assay(vst)), scale=T)

v <- res$sdev^2

v <- v / sum(v)

v[1]<-round(v[1]*100,1)

v[2]<-round(v[2]*100,1)

v[3]<-round(v[3]*100,1)

v[4]<-round(v[4]*100,1)

v[5]<-round(v[5]*100,1)

v[6]<-round(v[6]*100,1)

v[7]<-round(v[7]*100,1)

v[8]<-round(v[8]*100,1)

v[9]<-round(v[9]*100,1)

v[1]

v[2]

v[3]

v[4]

v[5]

PC1 <- res$x[, 1]

PC2 <- res$x[, 2]

PC3 <- res$x[, 3]

PC4 <- res$x[, 4]

PC5 <- res$x[, 5]

PC6 <- res$x[, 6]

PC7 <- res$x[, 7]

PC8 <- res$x[, 8]

PC9 <- res$x[, 9]

PC1_9<- res$x[,1:9]

outfile <- paste(outdir, "Score_VST_PCA_prcomp.txt", sep="/")

write.table(PC1_9, file=outfile, sep="\t")

col <- c("skyblue3","skyblue3","skyblue3","skyblue3","skyblue3","skyblue3","skyblue3","skyblue3","skyblue3","skyblue3","skyblue3","skyblue3","palevioletred1","palevioletred1","palevioletred1","palevioletred1","palevioletred1","palevioletred1","palevioletred1","palevioletred1","palevioletred1","palevioletred1","palevioletred1","palevioletred1")

pch <- c(0, 0, 0, 0, 0, 0, 0, 0, 0, 0, 0, 0,0, 0, 0, 0, 0, 0, 0, 0, 0, 0, 0, 0, 1, 1, 1, 1, 1, 1, 1, 1, 1, 1, 1, 1, 1, 1, 1, 1, 1, 1, 1, 1, 1, 1, 1, 1)

label3 <- c("I", "I", "I", "II", "II", "II", "III", "III","III", "IV", "IV", "IV", "I", "I","I", "II", "II", "II", "III", "III", "III", "IV", "IV", "IV")

outfile <- paste(outdir, "VST_PCA_prcomp_PC1-2_Fig.5B.pdf", sep="/");

pdf(file=outfile,onefile=FALSE,paper="special",height=15,width=15,family="Times",pointsize=20)

xname <- paste("PC1_", v[1], "%", sep="");

yname <- paste("PC2_", v[2], "%", sep="");

plot(PC1, PC2, pch=1, cex=6, col = col,xlab=xname, ylab=yname, main="VSA_PCA_prcomp_PC1-PC2")

text(PC1, PC2, label3, col = col, cex=2.5)

dev.off();

#loading factor

fc.l2<-sweep(res$rotation, MARGIN=2, res$sdev, FUN="*")

newTPMdata<-read.delim(newTPMdata,header=T,sep="\t")

colname <- newTPMdata[,1];

colname <- as.matrix(colname)

nfu_G <- data3[,1]

nfu_zeb<-read.delim(infile3, sep="\t",header=F);

nfu_zeb <- as.matrix(nfu_zeb)

L1 <- length(nfu_G)

L2 <- L1+1

L2

t5 <- c()

t4 <- subset (nfu_zeb, nfu_zeb[,1] == nfu_G[1], 4)

t4 <- t4[1]

for (i in 2:L1) {

  t5 <- subset (nfu_zeb, nfu_zeb[,1] ==  nfu_G[i], 4)

  t6 <- t5[1]

  t4 <- c (t4, t6)

}

zebraID <- t4

zebraID <- as.matrix(zebraID)

colnames(zebraID)<-c("zebra_geneID")

length(zebraID)

t5 <- c()

t4 <- subset (nfu_zeb, nfu_zeb[,1] == nfu_G[1], 5)

t4 <- t4[1]

for (i in 2:L1) {

  t5 <- subset (nfu_zeb, nfu_zeb[,1] ==  nfu_G[i], 5)

  t6 <- t5[1]

  t4 <- c (t4, t6)

}

zebraGName <- t4

zebraGName <- as.matrix(zebraGName)

colnames(zebraGName)<-c("zebra_geneName")

t5 <- c()

t4 <- subset (nfu_zeb, nfu_zeb[,1] == nfu_G[1], 6)

t4 <- t4[1]

for (i in 2:L1) {

  t5 <- subset (nfu_zeb, nfu_zeb[,1] ==  nfu_G[i], 6)

  t6 <- t5[1]

  t4 <- c (t4, t6)

}

zebraGene <- t4

zebraGene <- as.matrix(zebraGene)

colnames(zebraGene)<-c("zebra_gene")

fc.l<-cbind(data3,zebraID,zebraGName,zebraGene,fc.l2)

outfile <- paste(outdir, "FactorLoadings_VST_PCA_prcomp.txt", sep="/")

write.table(fc.l, file=outfile, sep="\t")

#______________________

#DEGs(For Supplementary Fig.3)

norm.dt   <- data.frame(counts(dds, normalized=T), check.names=F)

fpkm.dt   <- data.frame(fpkm(dds, robust=F), check.names=F)

tpm.dt    <- data.frame(sweep(fpkm.dt*1e6, 2, colSums(fpkm.dt),"/"), check.names=F)

cpm.dt    <- data.frame(fpm(dds, robust=F), check.names=F)

outfile <- paste(outDir, "DESeq2_results.Rdata", sep="/")

wb <- createWorkbook(type="xlsx")

sheet  <- createSheet(wb, sheet="TPM")

row.names(tpm.dt)<-row.names(data)

colnames(tpm.dt)<-name1

addDataFrame(tpm.dt, sheet, row.names=T)

saveWorkbook(wb, file=outFile)

outfile <- paste(outDir, "TPM.txt", sep="/")

write.table(tpm.dt, file=outfile, row.names=T)

dds <- DESeqDataSetFromMatrix(countData=data2, colData=Group, design=~ con)

genelength <- matrix( unlist(data [5]), ncol = 1)

mcols(dds)$basepairs <- as.numeric(genelength)

dds <- DESeq(dds)

terms<-c("S11w","S8w","S5w","S3w","G11w","G10w","G8w","G5w")

terms2<-combn(terms,2)

terms2

n<-ncol(terms2)

#newTPMdata(TPM>10)

newTPMdata<-as.matrix(newTPMdata)

L1 <- length(TPMmax10)

L1

#TPM

t5 <- c()

t4 <- subset (newTPMdata, newTPMdata[,1] == TPMmax10[1])

t4 <- t4

for (i in 2:L1) {

  t5 <- subset (newTPMdata, newTPMdata[,1] ==  TPMmax10[i], )

  t4 <- rbind (t4, t5)

}

newTPMdata<-t4

FC<-log2(2)

print(FC)

for (j in 1:n) {

  terms3<-terms2[,j]

  terms4<-append("con",terms3)

  print(terms4)

  res<-results(dds, contrast=terms4)

  res$TPM <-newTPMdata

  tpm<-THtpm

  data2 <- as.data.frame(res)

  data2upAll <- subset(data2, (data2$pad<=0.01 & (data2$log2FoldChange>=FC )))

  outfilename <- paste0("DESeq2_result_", terms3[1],"vs",terms3[2],"_TPMmax10_2.0upAll.txt")

  out_f <- paste(outDir, outfilename, sep="/");

  write.table(data2upAll, out_f, sep="\t", append=F, quote=F, row.names=T, col.names=T)

  data2downAll <- subset(data2, (data2$pad<=0.01 & (data2$log2FoldChange<=-FC )))

  outfilename <- paste0("DESeq2_result_", terms3[1],"vs",terms3[2],"_TPMmax10_2.0downAll.txt")

  out_f <- paste(outDir, outfilename, sep="/");

  write.table(data2downAll, out_f, sep="\t", append=F, quote=F, row.names=T, col.names=T)

}

**(H-8) ID-TPMv2.R**

#TPM

comm <- commandArgs(trailingOnly = T)

infile1 <- comm[1]

infile2 <- comm[2]

infile3 <- comm[3]

outdir <- comm[4]

TPM <- read.delim(infile3, sep="\t")

TPM <- as.matrix(TPM)

downcomID <- read.delim(infile1,header=F,sep=",")

downcomID <-as.matrix(downcomID)

downcomID

L1 <- length(downcomID)

L1

#TPM

t5 <- c()

t4 <- subset (TPM, TPM[,1] == downcomID[1])

t4 <- t4

for (i in 2:L1) {

   t5 <- subset (TPM, TPM[,1] ==  downcomID[i], )

   t4 <- rbind (t4, t5)

}

head(t4)

outfile <- paste(outdir, infile2, sep="/")

write.table(t4, file=outfile, sep="\t")

**(H-9) Fig5C-testis.R**

#heatmap

library(lattice)

library(latticeExtra)

library(gplots)

library(matrixStats)

library(genefilter)

library(dplyr)

my.col1 <- colorRampPalette(c("blue","white","magenta"))

comm <- commandArgs(trailingOnly = T)

infile1 <- comm[1]

outdir <- comm[2]

data<-read.delim(infile1,header=T, sep="\t")

x<-data[,3:25]

x <- as.matrix(x)

x_a<-x/rowMeans(x)

log_x_a<-log(x_a,base=2)

outfile <- paste(outdir, "Fig5C-testis.pdf", sep="/")

pdf(file=outfile,onefile=FALSE,paper="special",height=15,width=15,family="Helvetica",pointsize=20)

heatmap.2(as.matrix(log_x_a),col=my.col1(269),Colv=NA, Rowv=NA,scale="none",key=TRUE, symm=F,symkey=F,symbreaks=T, breaks=c(seq(-0.5,0.5,length=270)), trace="none",distfun = function(x) {dist(x, method="euclidean")},hclustfun = function(x) {hclust(x, method="ward.D2")})

dev.off()

**(H-10) ID-TPMv2.R**

#TPM

comm <- commandArgs(trailingOnly = T);

infile1 <- comm[1]

infile2 <- comm[2]

infile3 <- comm[3]

outdir <- comm[4]

TPM <- read.delim(infile3, sep="\t");

TPM <- as.matrix(TPM)

downcomID <- read.delim(infile1,header=F,sep=",")

downcomID <-as.matrix(downcomID)

downcomID

L1 <- length(downcomID)

L1

#TPM

t5 <- c()

t4 <- subset (TPM, TPM[,1] == downcomID[1])

t4 <- t4

for (i in 2:L1) {

   t5 <- subset (TPM, TPM[,1] ==  downcomID[i], )

   t4 <- rbind (t4, t5)

}

head(t4)

outfile <- paste(outdir, infile2, sep="/")

write.table(t4, file=outfile, sep="\t")

**(H-11) Fig5C-ovary.R**

#heatmap

library(lattice)

library(latticeExtra)

library(gplots)

library(matrixStats)

library(genefilter)

library(dplyr)

my.col1 <- colorRampPalette(c("blue","white","magenta"))

comm <- commandArgs(trailingOnly = T)

infile1 <- comm[1]

outdir <- comm[2]

indata <- read.delim(infile1, header=T, sep="\t")

data<-read.delim("ovaryGOterms-nfu-TPM.txt",header=T, sep="\t")

x<-data[,3:26]

x <- as.matrix(x)

x_a<-x/rowMeans(x)

log_x_a<-log(x_a, base=2)

outfile <- paste(outdir, "Fig5C-ovary.pdf", sep="/")

pdf(file=outfile,onefile=FALSE,paper="special",height=15,width=15,family="Helvetica",pointsize=20)

heatmap.2(as.matrix(log_x_a),col=my.col1(269),Colv=NA, Rowv=NA,scale="none",key=TRUE, symm=F,symkey=F,symbreaks=T, breaks=c(seq(-1,1,length=270)), trace="none",distfun = function(x) {dist(x, method="euclidean")},hclustfun = function(x) {hclust(x, method="ward.D2")})

dev.off()

**(H-12) blast4.R**

#Making nfu_zeb nfu_medaka table

args <- commandArgs(T)

nn   <- args[1] #nfu_nfu

nz   <- args[2] #nfu_zeb

nm   <- args[3]#nfu_medaka

outDir <- args[4] #output

nn <- read.delim(nn,header=F,sep="\t")

nn <- as.matrix(nn)

nz <- read.delim(nz,header=F,sep="\t")

nz <- as.matrix(nz)

nm <- read.delim(nm,header=F,sep="\t")

nm <- as.matrix(nm)

#Getting zebra information from Nfu Protein ID

nn_P <- nn[,1]

nn_P <- as.matrix(nn_P)

L1 <- length(nn_P[,1])

t4 <- c()

t4 <- as.matrix(subset (nz, nz[,1] == nn_P[1]))

t4

t4 <- t4[1,]

t4

t5 <- c()

t6 <- c()

t7 <- c()

for (i in 2:L1) {

  t5 <- as.matrix(subset (nz, nz[,1] ==  nn_P[i]))

  t5 <- as.matrix(t5[!is.na(t5[,1]),])

  if (length(t5) < 17) {

    t6 <-t5[1:16]

  } else {

    t6 <- t5[1,]

  }

  t7 <- rbind (t4, t6)

  t4 <- t7

}

outfile <- paste(outDir, "nfu_zeb_t4.txt", sep="/")

write.table(t4, file=outfile, row.names=F, sep = "\t", quote=F)

#Getting medaka information from Nfu Protein ID

t4 <- as.matrix(t4)

head(t4)

length(t4[,1])

L1

t8 <- c()

t8 <- as.matrix(subset (nm, nm[,1] == nn_P[1]))

t8 <- t8[1,]

t8

t9 <- c()

t10 <- c()

t11 <- c()

for (i in 2:L1) {

  t9 <- as.matrix(subset (nm, nm[,1] ==  nn_P[i]))

  t9 <- as.matrix(t9[!is.na(t9[,1]),])

  if (length(t9) < 17) {

    t10 <-t9[1:16]

  } else {

    t10 <- t9[1,]

  }

  t11 <- rbind (t8, t10)

  t8 <- t11

}

t8 <- as.matrix(t8)

head(t8)

length(t8[,1])

L1

#Getting nfu information from Nfu Protein ID

t12 <- c()

t12 <- subset (nn, nn[,1] == nn_P[1])

t12 <- t12[1,]

t12

t13 <- c()

t14 <- c()

t15 <- c()

for (i in 2:L1) {

  t13 <- as.matrix(subset (nn, nn[,1] ==  nn_P[i]))

  t13 <- as.matrix(t13[!is.na(t13[,1]),])

  if (length(t13) < 17) {

    t14 <-t13[1:16]

  } else {

    t14 <- t13[1,]

  }

  t15 <- rbind (t12, t14)

  t12 <- t15

}

t12 <- as.matrix(t12)

head(t12)

length(t12[,1])

L1

length(t12[,16])

length(t12[,14:15])

length(t4[,16])

length(t4[,14:15])

length(t4[,3])

length(t8[,16])

length(t8[,14:15])

length(t8[,3])

outfile <- paste(outDir, "nfu_gID.txt", sep="/")

write.table(t12[,16] , file=outfile, row.names=F, sep = "\t", quote=F)

outfile <- paste(outDir, "nfu_gNameDis.txt", sep="/")

write.table(t12[,14:15] , file=outfile, row.names=F, sep = "\t", quote=F)

outfile <- paste(outDir, "zeb_gID.txt", sep="/")

write.table(t4[,16] , file=outfile, row.names=F, sep = "\t", quote=F)

outfile <- paste(outDir, "zeb_gNameDis.txt", sep="/")

write.table(t4[,14:15] , file=outfile, row.names=F, sep = "\t", quote=F)

outfile <- paste(outDir, "zeb_homology.txt", sep="/")

write.table(t4[,3] , file=outfile, row.names=F, sep = "\t", quote=F)

outfile <- paste(outDir, "medaka_gID.txt", sep="/")

write.table(t8[,16], file=outfile, row.names=F, sep = "\t", quote=F)

outfile <- paste(outDir, "medaka_gNameDis.txt", sep="/")

write.table(t8[,14:15] , file=outfile, row.names=F, sep = "\t", quote=F)

outfile <- paste(outDir, "medaka_homology.txt", sep="/")

write.table(t8[,3] , file=outfile, row.names=F, sep = "\t", quote=F)

nzm <-cbind(t12[,16],t12[,14:15],t4[,16],t4[,14:15],t4[,3],t8[,16],t8[,14:15],t8[,3])

outfile <- paste(outDir, "nfu_zeb_medaka.txt", sep="/")

write.table(nzm , file=outfile, row.names=F, sep = "\t", quote=F)
