# Supplementary material for: Single housing of juveniles accelerates early-stage growth but extends adult lifespan in African turquoise killifish
Source: Aging (Albany NY). 2024 Sep 16;16(18):12443–72. doi: 10.18632/aging.206111 (PMC11466477; doi:10.18632/aging.206111)
Supplement: Supplementary Material 2 [file aging-16-206111-s005.docx]

**Supplementary Material 2. Script for livers analysis (Figure 6).**

**(A) Linux commands**

#0 Analysis environment

Ubuntu 18.04.6 LTS

#1. Making directories

workDir=/mnt/d/takahashi/Livers

mkdir $workDir

scDir=/mnt/d/takahashi/Livers/00_reference

mkdir $scDir&& cd $_

#Copying sample information file to 00_reference

cp /mnt/d/takahashi/sample.csv $_

#Making directories from the information of the sample file

cat sample.csv | awk -F "," '{print $2}'  > filename.txt

cat sample.csv | awk -F "," '{print $3}'  > workDir.txt

paste -d "/" workDir.txt filename.txt > FilenameDir.txt

cat FilenameDir.txt | {

while read dir1

  do

      mkdir -p $dir1 && cd $_

      mkdir 01_raw-seq 02_fastqc 03_valid-seq 04_abundant-check 05_mapping 06_count 07_clustering 08_DESeq2

  done

}

cd $workDir

tree

#2. Making index for HISAT2

cd $scDir

wget ftp://ftp.ensembl.org/pub/release-108/fasta/nothobranchius_furzeri/dna/Nothobranchius_furzeri.Nfu_20140520.dna.toplevel.fa.gz

gzip -d Nothobranchius_furzeri.Nfu_20140520.dna.toplevel.fa.gz

hisat2-build Nothobranchius_furzeri.Nfu_20140520.dna.toplevel.fa Nothobranchius_furzeri.Nfu_20140520.dna.toplevel

cd $scDir

hisat2-build nfu.abundant.fa nfu.abundant

#3-1. Download of Genome Data from Ensembl

cd $scDir

wget ftp://ftp.ensembl.org/pub/release-108/gtf/nothobranchius_furzeri/Nothobranchius_furzeri.Nfu_20140520.108.gtf.gz

gzip -d Nothobranchius_furzeri.Nfu_20140520.108.gtf.gz

#3-2. Making homologues list

#See Supplementary Figure 8(A) Linux commands #3-2. Making homologues list (pages 2-4)

#4. check of raw data in 01_rawdata

workDir=/mnt/d/takahashi/Livers

scDir=/mnt/d/takahashi/Livers/00_reference

cd $scDir

cat FilenameDir.txt | {

while read dir1

  do

    cd $dir1/01_raw-seq

    for d in `find ./ -type d`;

        do echo $dir1,`ls "$d" | wc -l`;

    done

    md5sum Lib*gz > sum.txt

  done

}

#5. RUN FastQC

workDir=/mnt/d/takahashi/Livers

scDir=/mnt/d/takahashi/Livers/00_reference

cd $scDir

cat FilenameDir.txt | {

  while read dir1

  do

      fastqc -t 8 --nogroup $dir1/01_raw-seq/Lib*.gz -o $dir1/02_fastqc

      multiqc $dir1/02_fastqc

      mv ./multiqc_report*.html $dir1/02_fastqc/

  done

}

#6. Processing sequence data with Trim galore

workDir=/mnt/d/takahashi/Livers

scDir=/mnt/d/takahashi/Livers/00_reference

cd $scDir

cat FilenameDir.txt | {

  while read dir1

  do

    Max=$((`find $dir1/01_raw-seq -type f | wc -l`/2))

    for i in `seq -f "%02g" $Max`

    do

    trim_galore -q 30 --length 35 \

    --paired $dir1/01_raw-seq/Lib${i}*R1_001.fastq.gz $dir1/01_raw-seq/Lib${i}*R2_001.fastq.gz \

    -o $dir1/03_valid-seq \

    &> $dir1/03_valid-seq/fastq${i}.trim-galore.log;

    done

  done

}

#7 genome mapping with HISAT2

workDir=/mnt/d/takahashi/Livers

scDir=/mnt/d/takahashi/Livers/00_reference

cd $scDir

cat FilenameDir.txt | {

  while read dir1

  do

  Max=$((`find $dir1/01_raw-seq -type f | wc -l`/2))

  for i in `seq -f "%02g" $Max`

  do

    hisat2 -p 8 -x $scDir/nfu.abundant \

      -1 $dir1/03_valid-seq/Lib${i}_*val_1.fq.gz \

      -2 $dir1/03_valid-seq/Lib${i}_*val_2.fq.gz \

      -S $dir1/04_abundant-check/Lib${i}.abundant.sam \

      --no-unal --no-hd \

      2>$dir1/04_abundant-check/Lib${i}.abundant.log;

  done

  done

}

workDir=/mnt/d/takahashi/Livers

scDir=/mnt/d/takahashi/Livers/00_reference

cd $scDir

cat FilenameDir.txt | {

  while read dir1

  do

    echo -e "library\tchrM\teGFP\tphiX174\trRNA\tmRNA" > $dir1/04_abundant-check/abundant.summary.log;

    Max=$((`find $dir1/01_raw-seq -type f | wc -l`/2))

    echo $Max;

  done

}

cat FilenameDir.txt | {

  while read dir1

  do

    Max=$((`find $dir1/01_raw-seq -type f | wc -l`/2))

    for i in `seq -f "%02g" $Max`

    do

    paste <(echo $dir1/04_abundant-check/Lib${i}) \

    <(grep -c chrM    $dir1/04_abundant-check/Lib${i}.abundant.sam) \

    <(grep -c eGFP    $dir1/04_abundant-check/Lib${i}.abundant.sam) \

    <(grep -c phiX174 $dir1/04_abundant-check/Lib${i}.abundant.sam) \

    <(grep -c EU780557    $dir1/04_abundant-check/Lib${i}.abundant.sam) \

    <(sed -n 3P $dir1/04_abundant-check/Lib${i}.abundant.log | cut -f 5 -d ' ') \

    >> $dir1/04_abundant-check/abundant.summary.log;

    done

  done

}

cat FilenameDir.txt | {

  while read dir1

  do

    R --no-save --args $dir1/04_abundant-check/abundant.summary.log $dir1/04_abundant-check/abundant < $scDir/logPlot.R

  done

}

workDir=/mnt/d/takahashi/Livers

scDir=/mnt/d/takahashi/Livers/00_reference

cd $scDir

cat FilenameDir.txt | {

  while read dir1

  do

    Max=$((`find $dir1/01_raw-seq -type f | wc -l`/2))

    for i in `seq -f "%02g" $Max`

    do

    hisat2 -p 8 -x $scDir/Nothobranchius_furzeri.Nfu_20140520.dna.toplevel --rna-strandness RF \

    -1 $dir1/03_valid-seq/Lib${i}*val_1.fq.gz -2 $dir1/03_valid-seq/Lib${i}*val_2.fq.gz \

    --dta \

    -S $dir1/05_mapping/Lib${i}.sam 2> $dir1/05_mapping/Lib${i}.mapping.log;

    done

  done

}

workDir=/mnt/d/takahashi/Livers

scDir=/mnt/d/takahashi/Livers/00_reference

cd $scDir

cat FilenameDir.txt | {

  while read dir1

  do

    Max=$((`find $dir1/01_raw-seq -type f | wc -l`/2))

    for i in `seq -f "%02g" $Max`

    do

    samtools sort -@ 8 $dir1/05_mapping/Lib${i}.sam > $dir1/05_mapping/Lib${i}.bam;

    samtools index -@ 8 $dir1/05_mapping/Lib${i}.bam;

    done

  done

}

workDir=/mnt/d/takahashi/Livers

scDir=/mnt/d/takahashi/Livers/00_reference

cd $scDir

cat FilenameDir.txt | {

  while read dir1

  do

    echo -e "library\tunmapped\tmulti-mapped\tuniq-mapped" > $dir1/05_mapping/mapping.summary.log;

    Max=$((`find $dir1/01_raw-seq -type f | wc -l`/2))

    for i in `seq -f "%02g" $Max`

    do

        paste <(echo $dir1/05_mapping/Lib${i}) \

        <(sed -n 3P $dir1/05_mapping/Lib${i}.mapping.log | cut -f 5 -d ' ') \

        <(sed -n 5P $dir1/05_mapping/Lib${i}.mapping.log | cut -f 5 -d ' ') \

        <(sed -n 4P $dir1/05_mapping/Lib${i}.mapping.log | cut -f 5 -d ' ') \

        >> $dir1/05_mapping/mapping.summary.log;

    done

    R --no-save --args $dir1/05_mapping/mapping.summary.log $dir1/05_mapping/mapping <  $scDir/logPlot.R

    done

}

#9.Counting with fetureCounts

workDir=/mnt/d/takahashi/Livers

scDir=/mnt/d/takahashi/Livers/00_reference

cd $scDir

cat FilenameDir.txt | {

    while read dir1

    do

    featureCounts -T 8 -p -B -C -t exon -g gene_id -a $scDir/Nothobranchius_furzeri.Nfu_20140520.108.gtf -o $dir1/06_count/all.featurecounts.txt $dir1/05_mapping/Lib*.bam;

  done

}

#Manually delete the first line of the text file "all.featurecounts.txt",  and rename it to "all.featurecounts2.txt".

workDir=/mnt/d/takahashi/Livers

scDir=/mnt/d/takahashi/Livers/00_reference

cd $scDir

cat FilenameDir.txt | {

    while read dir1

    do

    R --no-save --args $dir1/06_count/all.featurecounts2.txt $dir1/06_count/ $dir1/06_count/colnames.txt <  $scDir/counts_to_tpm2.R #H-2 script

    R --no-save --args $dir1/06_count/TPMfeaturecounts.txt $dir1/06_count $dir1/06_count/colnames.txt<  $scDir/TH.R #H-3 script

    R --no-save --args /mnt/d/takahashi/blast/ncbi-blast-2.13.0+-src/c++/nfu_zeb_medaka.txt $dir1/06_count/TPMfeaturecounts.txt $dir1/06_count/ <  /mnt/d/takahashi/blast/ncbi-blast-2.13.0+-src/c++/blast3.R #H-9 script

    R --no-save --args $dir1/06_count/TPMfeaturecounts_max10.txt $dir1/06_count  <  $dir1/06_count/Fig6B.R #H-4 script

  done

}

#10. DEseq2

#Manually delete ' of the text file "newTPMfeaturecounts.txt" in Excel (microsoft),  and rename it to "newTPMfeaturecounts2.txt".

workDir=/mnt/d/takahashi/Livers/male

scDir=/mnt/d/takahashi/Livers/00_reference

wdir=$workDir/08_DESeq2/

data=$workDir/06_count/all.featurecounts2.txt

INDEX=$workDir/08_DESeq2/samplenames.txt

GTF=$scDir/Nothobranchius_furzeri.Nfu_20140520.108.gtf

default=default

TPMdata=$workDir/06_count/TPMfeaturecounts_max10.txt

newTPMdata=$workDir/06_count/newTPMfeaturecounts2.txt

THtpm=10

ID=/mnt/d/takahashi/blast/ncbi-blast-2.13.0+-src/c++/nfu_zeb_medaka.txt

R --no-save --args $wdir $data $INDEX $GTF defalt $TPMdata $newTPMdata $THtpm $ID< $workDir/08_DESeq2/DESeq2.R #H-5 script

#11. Clustering of 1588_age_related_genes

workDir=/mnt/d/takahashi/Livers/male/06_count

cd $workDir

R --no-save --args ./1588_age_related_genes.csv 1588_age_related_genes-TPM.txt ./newTPMfeaturecounts2.txt ./ <  ./ID-TPMv1.R #H-6 script

workDir=/mnt/d/takahashi/Livers/male

scDir=/mnt/d/takahashi/Livers/00_reference

cd $workDir/07_clustering

R --no-save --args $workDir/07_clustering/1588_age_related_genes-TPM.txt $workDir/07_clustering <  $workDir/07_clustering/Fig6C.R #H-7 script

#12. graphs of the expression change of cell scenesence-related genes

workDir=/mnt/d/takahashi/Livers/male/06_count

cd $workDir

R --no-save --args ./liver_graph.txt liver_graph-TPM.txt ./newTPMfeaturecounts2.txt ./ <  ./ID-TPMv1.R  #H-6 script

R --no-save --args liver_graph-TPM.txt ./ liver_graph-TPM# < ./Fig6D.R #H-8 script

**(B) sample.csv**

1,male,/mnt/d/takahashi/Livers,

**(C) nfu.abundant.fa**

See Supplementary Figure 8(C) nfu.abundant.fa (pages 11-17)

**(D) colnames.txt**

geneID,GeneLength,G7w_1,G7w_2,G7w_3,G7w_4,G7w_5,G7w_6,G7w_7,G7w_8,G7w_9,G7w_10,S7w_1,S7w_2,S7w_3,S7w_4,S7w_5,S7w_6,S7w_7,S7w_8,S7w_9,S7w_10,G14w_2,G14w_3,G14w_4,G14w_5,G14w_6,G14w_7,G14w_8,G14w_9,G14w_10,S14w_1,S14w_2,S14w_3,S14w_4,S14w_5,S14w_6,S14w_7,S14w_8,S14w_9,S14w_10

**(E) samplenames.txt**

G7w,G7w,G7w,G7w,G7w,G7w,G7w,G7w,G7w,G7w,S7w,S7w,S7w,S7w,S7w,S7w,S7w,S7w,S7w,S7w,G14w,G14w,G14w,G14w,G14w,G14w,G14w,G14w,G14w,S14w,S14w,S14w,S14w,S14w,S14w,S14w,S14w,S14w,S14w

**(F)** **1588_age_related_genes.csv**

ENSNFUG00015000772

ENSNFUG00015000843

ENSNFUG00015000960

ENSNFUG00015001123

ENSNFUG00015001228

ENSNFUG00015001233

ENSNFUG00015001437

ENSNFUG00015001617

ENSNFUG00015001635

ENSNFUG00015001689

ENSNFUG00015001762

ENSNFUG00015002061

ENSNFUG00015002471

ENSNFUG00015003275

ENSNFUG00015003337

ENSNFUG00015003758

ENSNFUG00015003956

ENSNFUG00015004128

ENSNFUG00015004196

ENSNFUG00015004389

ENSNFUG00015004396

ENSNFUG00015004485

ENSNFUG00015004651

ENSNFUG00015004961

ENSNFUG00015005562

ENSNFUG00015005723

ENSNFUG00015006102

ENSNFUG00015007473

ENSNFUG00015008635

ENSNFUG00015010592

ENSNFUG00015011950

ENSNFUG00015012230

ENSNFUG00015013022

ENSNFUG00015013833

ENSNFUG00015013978

ENSNFUG00015019376

ENSNFUG00015023111

ENSNFUG00015023259

ENSNFUG00015000248

ENSNFUG00015000262

ENSNFUG00015000765

ENSNFUG00015000830

ENSNFUG00015001104

ENSNFUG00015001111

ENSNFUG00015001224

ENSNFUG00015001247

ENSNFUG00015001349

ENSNFUG00015001395

ENSNFUG00015001485

ENSNFUG00015001681

ENSNFUG00015001750

ENSNFUG00015002179

ENSNFUG00015002333

ENSNFUG00015002672

ENSNFUG00015002701

ENSNFUG00015002840

ENSNFUG00015003271

ENSNFUG00015003351

ENSNFUG00015003622

ENSNFUG00015003795

ENSNFUG00015004047

ENSNFUG00015004349

ENSNFUG00015004753

ENSNFUG00015005988

ENSNFUG00015006460

ENSNFUG00015006536

ENSNFUG00015007397

ENSNFUG00015007556

ENSNFUG00015007712

ENSNFUG00015011445

ENSNFUG00015011619

ENSNFUG00015012163

ENSNFUG00015012229

ENSNFUG00015012910

ENSNFUG00015013813

ENSNFUG00015014427

ENSNFUG00015017855

ENSNFUG00015018683

ENSNFUG00015019083

ENSNFUG00015020606

ENSNFUG00015020686

ENSNFUG00015020954

ENSNFUG00015021050

ENSNFUG00015025184

ENSNFUG00015000548

ENSNFUG00015000838

ENSNFUG00015001388

ENSNFUG00015001893

ENSNFUG00015002145

ENSNFUG00015002387

ENSNFUG00015002425

ENSNFUG00015002710

ENSNFUG00015002854

ENSNFUG00015002962

ENSNFUG00015003024

ENSNFUG00015003360

ENSNFUG00015003378

ENSNFUG00015003768

ENSNFUG00015003902

ENSNFUG00015003922

ENSNFUG00015004149

ENSNFUG00015004262

ENSNFUG00015005770

ENSNFUG00015006652

ENSNFUG00015007234

ENSNFUG00015012586

ENSNFUG00015012711

ENSNFUG00015012739

ENSNFUG00015012877

ENSNFUG00015015976

ENSNFUG00015017390

ENSNFUG00015018533

ENSNFUG00015023972

ENSNFUG00015024451

ENSNFUG00015025159

ENSNFUG00015025241

ENSNFUG00015000474

ENSNFUG00015000520

ENSNFUG00015000665

ENSNFUG00015000932

ENSNFUG00015001155

ENSNFUG00015001402

ENSNFUG00015001408

ENSNFUG00015001464

ENSNFUG00015001534

ENSNFUG00015001790

ENSNFUG00015002674

ENSNFUG00015003305

ENSNFUG00015003313

ENSNFUG00015003441

ENSNFUG00015003479

ENSNFUG00015003488

ENSNFUG00015003491

ENSNFUG00015003820

ENSNFUG00015004861

ENSNFUG00015005612

ENSNFUG00015009049

ENSNFUG00015009113

ENSNFUG00015009973

ENSNFUG00015010069

ENSNFUG00015010496

ENSNFUG00015011900

ENSNFUG00015012290

ENSNFUG00015012293

ENSNFUG00015012295

ENSNFUG00015012799

ENSNFUG00015013734

ENSNFUG00015013807

ENSNFUG00015016298

ENSNFUG00015016407

ENSNFUG00015019132

ENSNFUG00015023040

ENSNFUG00015023520

ENSNFUG00015024370

ENSNFUG00015024416

ENSNFUG00015000103

ENSNFUG00015000317

ENSNFUG00015000425

ENSNFUG00015000499

ENSNFUG00015000710

ENSNFUG00015000882

ENSNFUG00015001521

ENSNFUG00015001827

ENSNFUG00015002121

ENSNFUG00015002131

ENSNFUG00015002305

ENSNFUG00015002327

ENSNFUG00015002638

ENSNFUG00015002713

ENSNFUG00015002917

ENSNFUG00015002933

ENSNFUG00015002976

ENSNFUG00015003037

ENSNFUG00015003414

ENSNFUG00015003681

ENSNFUG00015004091

ENSNFUG00015004142

ENSNFUG00015004144

ENSNFUG00015004278

ENSNFUG00015004300

ENSNFUG00015004309

ENSNFUG00015004569

ENSNFUG00015005364

ENSNFUG00015006147

ENSNFUG00015006209

ENSNFUG00015006755

ENSNFUG00015007563

ENSNFUG00015007620

ENSNFUG00015008204

ENSNFUG00015008271

ENSNFUG00015008597

ENSNFUG00015008618

ENSNFUG00015008674

ENSNFUG00015010534

ENSNFUG00015011707

ENSNFUG00015011800

ENSNFUG00015014789

ENSNFUG00015015424

ENSNFUG00015017287

ENSNFUG00015020230

ENSNFUG00015022132

ENSNFUG00015000125

ENSNFUG00015000523

ENSNFUG00015000581

ENSNFUG00015000624

ENSNFUG00015000651

ENSNFUG00015000835

ENSNFUG00015001183

ENSNFUG00015001572

ENSNFUG00015002204

ENSNFUG00015002610

ENSNFUG00015002614

ENSNFUG00015002816

ENSNFUG00015003371

ENSNFUG00015003372

ENSNFUG00015003375

ENSNFUG00015003489

ENSNFUG00015004027

ENSNFUG00015004164

ENSNFUG00015004570

ENSNFUG00015004688

ENSNFUG00015006585

ENSNFUG00015006589

ENSNFUG00015007706

ENSNFUG00015010233

ENSNFUG00015010942

ENSNFUG00015010958

ENSNFUG00015010964

ENSNFUG00015010981

ENSNFUG00015013943

ENSNFUG00015015054

ENSNFUG00015015059

ENSNFUG00015015209

ENSNFUG00015016532

ENSNFUG00015017980

ENSNFUG00015019674

ENSNFUG00015020192

ENSNFUG00015020297

ENSNFUG00015020426

ENSNFUG00015020430

ENSNFUG00015022769

ENSNFUG00015024501

ENSNFUG00015024660

ENSNFUG00015024733

ENSNFUG00015001364

ENSNFUG00015003798

ENSNFUG00015003878

ENSNFUG00015004394

ENSNFUG00015004647

ENSNFUG00015005613

ENSNFUG00015006258

ENSNFUG00015007012

ENSNFUG00015007298

ENSNFUG00015007690

ENSNFUG00015008874

ENSNFUG00015010218

ENSNFUG00015011622

ENSNFUG00015017484

ENSNFUG00015017826

ENSNFUG00015018792

ENSNFUG00015019247

ENSNFUG00015020743

ENSNFUG00015023046

ENSNFUG00015004398

ENSNFUG00015004822

ENSNFUG00015005910

ENSNFUG00015006411

ENSNFUG00015006602

ENSNFUG00015006938

ENSNFUG00015007711

ENSNFUG00015009546

ENSNFUG00015010404

ENSNFUG00015011169

ENSNFUG00015011184

ENSNFUG00015011190

ENSNFUG00015011873

ENSNFUG00015012375

ENSNFUG00015012928

ENSNFUG00015012936

ENSNFUG00015012947

ENSNFUG00015013329

ENSNFUG00015014351

ENSNFUG00015015123

ENSNFUG00015015726

ENSNFUG00015019164

ENSNFUG00015019551

ENSNFUG00015020026

ENSNFUG00015020755

ENSNFUG00015021708

ENSNFUG00015022366

ENSNFUG00015022783

ENSNFUG00015023162

ENSNFUG00015023357

ENSNFUG00015023858

ENSNFUG00015024126

ENSNFUG00015005017

ENSNFUG00015006015

ENSNFUG00015006124

ENSNFUG00015007373

ENSNFUG00015008323

ENSNFUG00015008558

ENSNFUG00015008595

ENSNFUG00015009386

ENSNFUG00015011464

ENSNFUG00015012362

ENSNFUG00015013021

ENSNFUG00015013378

ENSNFUG00015013669

ENSNFUG00015013981

ENSNFUG00015016258

ENSNFUG00015018822

ENSNFUG00015020742

ENSNFUG00015020812

ENSNFUG00015021324

ENSNFUG00015021373

ENSNFUG00015022116

ENSNFUG00015022818

ENSNFUG00015022911

ENSNFUG00015023235

ENSNFUG00015023248

ENSNFUG00015025232

ENSNFUG00015004698

ENSNFUG00015005064

ENSNFUG00015005986

ENSNFUG00015006013

ENSNFUG00015006050

ENSNFUG00015006067

ENSNFUG00015006550

ENSNFUG00015006835

ENSNFUG00015007704

ENSNFUG00015007925

ENSNFUG00015007950

ENSNFUG00015008369

ENSNFUG00015008591

ENSNFUG00015009233

ENSNFUG00015010162

ENSNFUG00015010583

ENSNFUG00015011206

ENSNFUG00015012929

ENSNFUG00015013578

ENSNFUG00015013878

ENSNFUG00015014553

ENSNFUG00015014582

ENSNFUG00015015382

ENSNFUG00015016424

ENSNFUG00015017956

ENSNFUG00015018071

ENSNFUG00015018825

ENSNFUG00015018942

ENSNFUG00015018959

ENSNFUG00015019091

ENSNFUG00015019155

ENSNFUG00015019437

ENSNFUG00015019633

ENSNFUG00015019939

ENSNFUG00015020163

ENSNFUG00015020541

ENSNFUG00015020617

ENSNFUG00015020684

ENSNFUG00015020810

ENSNFUG00015020832

ENSNFUG00015021082

ENSNFUG00015021189

ENSNFUG00015022111

ENSNFUG00015022530

ENSNFUG00015022746

ENSNFUG00015022888

ENSNFUG00015023595

ENSNFUG00015023968

ENSNFUG00015023987

ENSNFUG00015025135

ENSNFUG00015004999

ENSNFUG00015005105

ENSNFUG00015005444

ENSNFUG00015005456

ENSNFUG00015005506

ENSNFUG00015005757

ENSNFUG00015005871

ENSNFUG00015006235

ENSNFUG00015006284

ENSNFUG00015006391

ENSNFUG00015006407

ENSNFUG00015006445

ENSNFUG00015006450

ENSNFUG00015006466

ENSNFUG00015006559

ENSNFUG00015007889

ENSNFUG00015009292

ENSNFUG00015009397

ENSNFUG00015009450

ENSNFUG00015009741

ENSNFUG00015009770

ENSNFUG00015011498

ENSNFUG00015012453

ENSNFUG00015013287

ENSNFUG00015013595

ENSNFUG00015013703

ENSNFUG00015013919

ENSNFUG00015013971

ENSNFUG00015016981

ENSNFUG00015019650

ENSNFUG00015021063

ENSNFUG00015021118

ENSNFUG00015021759

ENSNFUG00015022599

ENSNFUG00015023503

ENSNFUG00015024209

ENSNFUG00015024478

ENSNFUG00015024513

ENSNFUG00015025089

ENSNFUG00015004865

ENSNFUG00015005599

ENSNFUG00015008800

ENSNFUG00015011631

ENSNFUG00015013469

ENSNFUG00015013622

ENSNFUG00015013677

ENSNFUG00015013739

ENSNFUG00015014030

ENSNFUG00015014763

ENSNFUG00015015801

ENSNFUG00015015856

ENSNFUG00015019055

ENSNFUG00015020154

ENSNFUG00015022121

ENSNFUG00015022209

ENSNFUG00015023880

ENSNFUG00015024206

ENSNFUG00015004933

ENSNFUG00015005102

ENSNFUG00015005471

ENSNFUG00015006805

ENSNFUG00015008829

ENSNFUG00015010628

ENSNFUG00015011852

ENSNFUG00015013499

ENSNFUG00015013520

ENSNFUG00015015326

ENSNFUG00015017752

ENSNFUG00015018102

ENSNFUG00015018210

ENSNFUG00015018748

ENSNFUG00015018838

ENSNFUG00015018920

ENSNFUG00015022329

ENSNFUG00015022572

ENSNFUG00015023247

ENSNFUG00015023513

ENSNFUG00015025177

ENSNFUG00015006129

ENSNFUG00015006247

ENSNFUG00015006293

ENSNFUG00015007042

ENSNFUG00015007977

ENSNFUG00015008091

ENSNFUG00015008098

ENSNFUG00015008285

ENSNFUG00015008358

ENSNFUG00015009577

ENSNFUG00015010586

ENSNFUG00015011416

ENSNFUG00015013046

ENSNFUG00015014191

ENSNFUG00015016189

ENSNFUG00015017001

ENSNFUG00015017068

ENSNFUG00015021307

ENSNFUG00015022262

ENSNFUG00015022429

ENSNFUG00015023300

ENSNFUG00015023839

ENSNFUG00015023923

ENSNFUG00015024701

ENSNFUG00015024804

ENSNFUG00015025210

ENSNFUG00015004816

ENSNFUG00015005929

ENSNFUG00015008827

ENSNFUG00015010571

ENSNFUG00015014022

ENSNFUG00015014228

ENSNFUG00015014433

ENSNFUG00015014501

ENSNFUG00015015456

ENSNFUG00015016037

ENSNFUG00015016847

ENSNFUG00015017215

ENSNFUG00015017277

ENSNFUG00015017540

ENSNFUG00015019993

ENSNFUG00015020286

ENSNFUG00015021182

ENSNFUG00015021593

ENSNFUG00015021745

ENSNFUG00015021896

ENSNFUG00015022245

ENSNFUG00015022289

ENSNFUG00015022556

ENSNFUG00015022684

ENSNFUG00015022938

ENSNFUG00015023511

ENSNFUG00015023649

ENSNFUG00015023812

ENSNFUG00015024359

ENSNFUG00015024965

ENSNFUG00015005847

ENSNFUG00015008490

ENSNFUG00015008875

ENSNFUG00015010290

ENSNFUG00015011665

ENSNFUG00015014190

ENSNFUG00015015206

ENSNFUG00015015261

ENSNFUG00015015956

ENSNFUG00015018035

ENSNFUG00015018284

ENSNFUG00015018401

ENSNFUG00015019882

ENSNFUG00015020050

ENSNFUG00015020347

ENSNFUG00015020955

ENSNFUG00015021689

ENSNFUG00015021690

ENSNFUG00015021693

ENSNFUG00015021721

ENSNFUG00015022744

ENSNFUG00015022830

ENSNFUG00015022928

ENSNFUG00015022940

ENSNFUG00015023239

ENSNFUG00015006128

ENSNFUG00015008202

ENSNFUG00015009561

ENSNFUG00015010402

ENSNFUG00015010621

ENSNFUG00015016915

ENSNFUG00015017432

ENSNFUG00015021408

ENSNFUG00015023558

ENSNFUG00015023560

ENSNFUG00015023570

ENSNFUG00015025021

ENSNFUG00015025127

ENSNFUG00015025312

ENSNFUG00015025381

ENSNFUG00015025406

ENSNFUG00015025420

ENSNFUG00015025446

ENSNFUG00015025458

ENSNFUG00015006052

ENSNFUG00015014094

ENSNFUG00015014995

ENSNFUG00015015276

ENSNFUG00015015302

ENSNFUG00015017060

ENSNFUG00015017071

ENSNFUG00015017290

ENSNFUG00015018482

ENSNFUG00015020932

ENSNFUG00015022932

ENSNFUG00015023067

ENSNFUG00015023611

ENSNFUG00015024098

ENSNFUG00015024106

ENSNFUG00015024201

ENSNFUG00015024223

ENSNFUG00015011242

ENSNFUG00015013419

ENSNFUG00015018249

ENSNFUG00015018254

ENSNFUG00015018467

ENSNFUG00015020075

ENSNFUG00015020771

ENSNFUG00015021569

ENSNFUG00015022868

ENSNFUG00015017465

ENSNFUG00015015250

ENSNFUG00015017413

ENSNFUG00015017425

ENSNFUG00015018016

ENSNFUG00015014621

ENSNFUG00015016082

ENSNFUG00015015025

ENSNFUG00015019720

ENSNFUG00015021522

ENSNFUG00015014333

ENSNFUG00015019328

ENSNFUG00015020308

ENSNFUG00015015862

ENSNFUG00015014839

ENSNFUG00015018454

ENSNFUG00015012740

ENSNFUG00015015374

ENSNFUG00015019160

ENSNFUG00015015940

ENSNFUG00015014336

ENSNFUG00015016136

ENSNFUG00015016166

ENSNFUG00015020889

ENSNFUG00015018459

ENSNFUG00015014206

ENSNFUG00015021545

ENSNFUG00015021546

ENSNFUG00015021551

ENSNFUG00015012127

ENSNFUG00015015963

ENSNFUG00015017958

ENSNFUG00015018032

ENSNFUG00015019707

ENSNFUG00015023469

ENSNFUG00015011681

ENSNFUG00015011531

ENSNFUG00015011984

ENSNFUG00015020423

ENSNFUG00015010977

ENSNFUG00015009511

ENSNFUG00015010460

ENSNFUG00015010466

ENSNFUG00015020233

ENSNFUG00015018677

ENSNFUG00015014131

ENSNFUG00015014717

ENSNFUG00015015223

ENSNFUG00015018655

ENSNFUG00015019802

ENSNFUG00015019819

ENSNFUG00015019926

ENSNFUG00015011758

ENSNFUG00015011968

ENSNFUG00015013519

ENSNFUG00015013033

ENSNFUG00015012445

ENSNFUG00015016104

ENSNFUG00015016768

ENSNFUG00015010322

ENSNFUG00015012826

ENSNFUG00015017020

ENSNFUG00015009143

ENSNFUG00015009154

ENSNFUG00015009242

ENSNFUG00015013178

ENSNFUG00015015290

ENSNFUG00015009109

ENSNFUG00015012953

ENSNFUG00015018342

ENSNFUG00015015870

ENSNFUG00015000009

ENSNFUG00015000039

ENSNFUG00015015293

ENSNFUG00015015303

ENSNFUG00015015307

ENSNFUG00015008969

ENSNFUG00015015188

ENSNFUG00015015681

ENSNFUG00015015819

ENSNFUG00015013764

ENSNFUG00015002818

ENSNFUG00015003662

ENSNFUG00015004375

ENSNFUG00015004781

ENSNFUG00015009311

ENSNFUG00015009898

ENSNFUG00015000944

ENSNFUG00015001905

ENSNFUG00015005645

ENSNFUG00015010808

ENSNFUG00015017401

ENSNFUG00015001033

ENSNFUG00015002173

ENSNFUG00015002182

ENSNFUG00015003343

ENSNFUG00015003473

ENSNFUG00015003683

ENSNFUG00015004612

ENSNFUG00015004874

ENSNFUG00015011598

ENSNFUG00015013181

ENSNFUG00015016605

ENSNFUG00015017288

ENSNFUG00015000347

ENSNFUG00015001398

ENSNFUG00015001509

ENSNFUG00015002147

ENSNFUG00015002410

ENSNFUG00015007546

ENSNFUG00015007598

ENSNFUG00015007606

ENSNFUG00015007666

ENSNFUG00015019435

ENSNFUG00015003420

ENSNFUG00015016301

ENSNFUG00015003986

ENSNFUG00015004324

ENSNFUG00015011783

ENSNFUG00015021982

ENSNFUG00015008712

ENSNFUG00015009269

ENSNFUG00015012309

ENSNFUG00015015633

ENSNFUG00015006095

ENSNFUG00015009619

ENSNFUG00015009835

ENSNFUG00015011862

ENSNFUG00015023011

ENSNFUG00015006505

ENSNFUG00015007198

ENSNFUG00015008246

ENSNFUG00015009940

ENSNFUG00015011168

ENSNFUG00015014414

ENSNFUG00015015748

ENSNFUG00015016247

ENSNFUG00015016466

ENSNFUG00015020628

ENSNFUG00015020646

ENSNFUG00015023615

ENSNFUG00015007629

ENSNFUG00015007791

ENSNFUG00015008009

ENSNFUG00015008179

ENSNFUG00015021675

ENSNFUG00015023760

ENSNFUG00015008794

ENSNFUG00015015970

ENSNFUG00015016338

ENSNFUG00015022023

ENSNFUG00015024287

ENSNFUG00015024901

ENSNFUG00015025162

ENSNFUG00015018313

ENSNFUG00015020146

ENSNFUG00015022763

ENSNFUG00015024340

ENSNFUG00015014174

ENSNFUG00015015072

ENSNFUG00015019849

ENSNFUG00015004926

ENSNFUG00015006808

ENSNFUG00015010738

ENSNFUG00015016485

ENSNFUG00015017354

ENSNFUG00015020601

ENSNFUG00015024390

ENSNFUG00015024401

ENSNFUG00015006971

ENSNFUG00015007362

ENSNFUG00015010343

ENSNFUG00015017851

ENSNFUG00015009620

ENSNFUG00015021074

ENSNFUG00015022906

ENSNFUG00015023557

ENSNFUG00015008542

ENSNFUG00015017155

ENSNFUG00015020311

ENSNFUG00015023644

ENSNFUG00015025319

ENSNFUG00015013123

ENSNFUG00015015259

ENSNFUG00015016035

ENSNFUG00015020295

ENSNFUG00015019837

ENSNFUG00015018229

ENSNFUG00015018235

ENSNFUG00015016991

ENSNFUG00015017004

ENSNFUG00015017331

ENSNFUG00015020551

ENSNFUG00015016573

ENSNFUG00015017210

ENSNFUG00015012209

ENSNFUG00015011597

ENSNFUG00015013879

ENSNFUG00015001315

ENSNFUG00015001876

ENSNFUG00015002266

ENSNFUG00015002514

ENSNFUG00015002592

ENSNFUG00015003005

ENSNFUG00015003628

ENSNFUG00015003656

ENSNFUG00015003918

ENSNFUG00015004230

ENSNFUG00015007817

ENSNFUG00015013006

ENSNFUG00015022671

ENSNFUG00015023683

ENSNFUG00015023887

ENSNFUG00015000759

ENSNFUG00015001743

ENSNFUG00015001977

ENSNFUG00015002643

ENSNFUG00015003041

ENSNFUG00015003513

ENSNFUG00015004344

ENSNFUG00015004373

ENSNFUG00015004622

ENSNFUG00015007377

ENSNFUG00015007820

ENSNFUG00015007824

ENSNFUG00015008032

ENSNFUG00015009075

ENSNFUG00015009818

ENSNFUG00015010238

ENSNFUG00015012249

ENSNFUG00015012863

ENSNFUG00015016623

ENSNFUG00015017832

ENSNFUG00015019334

ENSNFUG00015021429

ENSNFUG00015021900

ENSNFUG00015024420

ENSNFUG00015000838

ENSNFUG00015002804

ENSNFUG00015003048

ENSNFUG00015004149

ENSNFUG00015006497

ENSNFUG00015006747

ENSNFUG00015011139

ENSNFUG00015014954

ENSNFUG00015015052

ENSNFUG00015018360

ENSNFUG00015018375

ENSNFUG00015018533

ENSNFUG00015023617

ENSNFUG00015025118

ENSNFUG00015000941

ENSNFUG00015001155

ENSNFUG00015001209

ENSNFUG00015002150

ENSNFUG00015002156

ENSNFUG00015002598

ENSNFUG00015003502

ENSNFUG00015004793

ENSNFUG00015010659

ENSNFUG00015010830

ENSNFUG00015011006

ENSNFUG00015012111

ENSNFUG00015012119

ENSNFUG00015016280

ENSNFUG00015024583

ENSNFUG00015000234

ENSNFUG00015000267

ENSNFUG00015000370

ENSNFUG00015000594

ENSNFUG00015001518

ENSNFUG00015001775

ENSNFUG00015002608

ENSNFUG00015003051

ENSNFUG00015003626

ENSNFUG00015004108

ENSNFUG00015004984

ENSNFUG00015005318

ENSNFUG00015007719

ENSNFUG00015010314

ENSNFUG00015010629

ENSNFUG00015011009

ENSNFUG00015020150

ENSNFUG00015000581

ENSNFUG00015002368

ENSNFUG00015002418

ENSNFUG00015002437

ENSNFUG00015002524

ENSNFUG00015002527

ENSNFUG00015002610

ENSNFUG00015004493

ENSNFUG00015008498

ENSNFUG00015019989

ENSNFUG00015020426

ENSNFUG00015020430

ENSNFUG00015021056

ENSNFUG00015024029

ENSNFUG00015024685

ENSNFUG00015002892

ENSNFUG00015003301

ENSNFUG00015003416

ENSNFUG00015005270

ENSNFUG00015005918

ENSNFUG00015007184

ENSNFUG00015022081

ENSNFUG00015022971

ENSNFUG00015010464

ENSNFUG00015010627

ENSNFUG00015010952

ENSNFUG00015012171

ENSNFUG00015012314

ENSNFUG00015012318

ENSNFUG00015012791

ENSNFUG00015015237

ENSNFUG00015017767

ENSNFUG00015018827

ENSNFUG00015019412

ENSNFUG00015020147

ENSNFUG00015023325

ENSNFUG00015006058

ENSNFUG00015006867

ENSNFUG00015007336

ENSNFUG00015008669

ENSNFUG00015012091

ENSNFUG00015014216

ENSNFUG00015016093

ENSNFUG00015019233

ENSNFUG00015019621

ENSNFUG00015019943

ENSNFUG00015020323

ENSNFUG00015020742

ENSNFUG00015021457

ENSNFUG00015023308

ENSNFUG00015005137

ENSNFUG00015006508

ENSNFUG00015007840

ENSNFUG00015008381

ENSNFUG00015013018

ENSNFUG00015013047

ENSNFUG00015013175

ENSNFUG00015015180

ENSNFUG00015018935

ENSNFUG00015019214

ENSNFUG00015022111

ENSNFUG00015023679

ENSNFUG00015025143

ENSNFUG00015006525

ENSNFUG00015008299

ENSNFUG00015009428

ENSNFUG00015011653

ENSNFUG00015012771

ENSNFUG00015015879

ENSNFUG00015017409

ENSNFUG00015021719

ENSNFUG00015022238

ENSNFUG00015024469

ENSNFUG00015024644

ENSNFUG00015024647

ENSNFUG00015004780

ENSNFUG00015005028

ENSNFUG00015005961

ENSNFUG00015006238

ENSNFUG00015006349

ENSNFUG00015008007

ENSNFUG00015008015

ENSNFUG00015008862

ENSNFUG00015012996

ENSNFUG00015022211

ENSNFUG00015024115

ENSNFUG00015024143

ENSNFUG00015024148

ENSNFUG00015024343

ENSNFUG00015004933

ENSNFUG00015006701

ENSNFUG00015012607

ENSNFUG00015013417

ENSNFUG00015018610

ENSNFUG00015019393

ENSNFUG00015023172

ENSNFUG00015023505

ENSNFUG00015024575

ENSNFUG00015005561

ENSNFUG00015005742

ENSNFUG00015007547

ENSNFUG00015008045

ENSNFUG00015008360

ENSNFUG00015014270

ENSNFUG00015016830

ENSNFUG00015017231

ENSNFUG00015025201

ENSNFUG00015014254

ENSNFUG00015014536

ENSNFUG00015017931

ENSNFUG00015018465

ENSNFUG00015022140

ENSNFUG00015022459

ENSNFUG00015022476

ENSNFUG00015022994

ENSNFUG00015023763

ENSNFUG00015024874

ENSNFUG00015008226

ENSNFUG00015020834

ENSNFUG00015022907

ENSNFUG00015023281

ENSNFUG00015008202

ENSNFUG00015010019

ENSNFUG00015015607

ENSNFUG00015017297

ENSNFUG00015024996

ENSNFUG00015025121

ENSNFUG00015006948

ENSNFUG00015012931

ENSNFUG00015013418

ENSNFUG00015022134

ENSNFUG00015023018

ENSNFUG00015024123

ENSNFUG00015014104

ENSNFUG00015014942

ENSNFUG00015016213

ENSNFUG00015016746

ENSNFUG00015017393

ENSNFUG00015018946

ENSNFUG00015018952

ENSNFUG00015018960

ENSNFUG00015019477

ENSNFUG00015019628

ENSNFUG00015022287

ENSNFUG00015022351

ENSNFUG00015019109

ENSNFUG00015021530

ENSNFUG00015014853

ENSNFUG00015021167

ENSNFUG00015021994

ENSNFUG00015014823

ENSNFUG00015013473

ENSNFUG00015013659

ENSNFUG00015022264

ENSNFUG00015023147

ENSNFUG00015012578

ENSNFUG00015017533

ENSNFUG00015011710

ENSNFUG00015021612

ENSNFUG00015016478

ENSNFUG00015017274

ENSNFUG00015017281

ENSNFUG00015019850

ENSNFUG00015012540

ENSNFUG00015012597

ENSNFUG00015010411

ENSNFUG00015010927

ENSNFUG00015011825

ENSNFUG00015013178

ENSNFUG00015015383

ENSNFUG00015010645

ENSNFUG00015012644

ENSNFUG00015017951

ENSNFUG00015014253

ENSNFUG00015000003

ENSNFUG00015000006

ENSNFUG00015000014

ENSNFUG00015010074

ENSNFUG00015015485

ENSNFUG00015014477

ENSNFUG00015000126

ENSNFUG00015000127

ENSNFUG00015000214

ENSNFUG00015000629

ENSNFUG00015000988

ENSNFUG00015001655

ENSNFUG00015001764

ENSNFUG00015002359

ENSNFUG00015002400

ENSNFUG00015002500

ENSNFUG00015003259

ENSNFUG00015004007

ENSNFUG00015004106

ENSNFUG00015004156

ENSNFUG00015004249

ENSNFUG00015004260

ENSNFUG00015004679

ENSNFUG00015005010

ENSNFUG00015005513

ENSNFUG00015006241

ENSNFUG00015011657

ENSNFUG00015012745

ENSNFUG00015013097

ENSNFUG00015014436

ENSNFUG00015016363

ENSNFUG00015018205

ENSNFUG00015023853

ENSNFUG00015024146

ENSNFUG00015000241

ENSNFUG00015000243

ENSNFUG00015000288

ENSNFUG00015000460

ENSNFUG00015000658

ENSNFUG00015000885

ENSNFUG00015000886

ENSNFUG00015001049

ENSNFUG00015001232

ENSNFUG00015001961

ENSNFUG00015003136

ENSNFUG00015003652

ENSNFUG00015003799

ENSNFUG00015003912

ENSNFUG00015004315

ENSNFUG00015004407

ENSNFUG00015004432

ENSNFUG00015004599

ENSNFUG00015004605

ENSNFUG00015004648

ENSNFUG00015005328

ENSNFUG00015005399

ENSNFUG00015005947

ENSNFUG00015006005

ENSNFUG00015006345

ENSNFUG00015007847

ENSNFUG00015008416

ENSNFUG00015009493

ENSNFUG00015012158

ENSNFUG00015012951

ENSNFUG00015012975

ENSNFUG00015012978

ENSNFUG00015012981

ENSNFUG00015012986

ENSNFUG00015018398

ENSNFUG00015018689

ENSNFUG00015018692

ENSNFUG00015019104

ENSNFUG00015019680

ENSNFUG00015019863

ENSNFUG00015020791

ENSNFUG00015020918

ENSNFUG00015020971

ENSNFUG00015021407

ENSNFUG00015021929

ENSNFUG00015024667

ENSNFUG00015001688

ENSNFUG00015001804

ENSNFUG00015002448

ENSNFUG00015002483

ENSNFUG00015002731

ENSNFUG00015003085

ENSNFUG00015003728

ENSNFUG00015003781

ENSNFUG00015003825

ENSNFUG00015003904

ENSNFUG00015005182

ENSNFUG00015005526

ENSNFUG00015006486

ENSNFUG00015007148

ENSNFUG00015007839

ENSNFUG00015012944

ENSNFUG00015013190

ENSNFUG00015013924

ENSNFUG00015017170

ENSNFUG00015017623

ENSNFUG00015017627

ENSNFUG00015022029

ENSNFUG00015023943

ENSNFUG00015025280

ENSNFUG00015000075

ENSNFUG00015000321

ENSNFUG00015000788

ENSNFUG00015000791

ENSNFUG00015000793

ENSNFUG00015001099

ENSNFUG00015001117

ENSNFUG00015001211

ENSNFUG00015001428

ENSNFUG00015001737

ENSNFUG00015001748

ENSNFUG00015001901

ENSNFUG00015001991

ENSNFUG00015002057

ENSNFUG00015002164

ENSNFUG00015002194

ENSNFUG00015002596

ENSNFUG00015002605

ENSNFUG00015003221

ENSNFUG00015003447

ENSNFUG00015003457

ENSNFUG00015003471

ENSNFUG00015003639

ENSNFUG00015003724

ENSNFUG00015003830

ENSNFUG00015003917

ENSNFUG00015004851

ENSNFUG00015005045

ENSNFUG00015005116

ENSNFUG00015005619

ENSNFUG00015005816

ENSNFUG00015006926

ENSNFUG00015007170

ENSNFUG00015007293

ENSNFUG00015008981

ENSNFUG00015009210

ENSNFUG00015009231

ENSNFUG00015009378

ENSNFUG00015009748

ENSNFUG00015011026

ENSNFUG00015012174

ENSNFUG00015016284

ENSNFUG00015016359

ENSNFUG00015018408

ENSNFUG00015023045

ENSNFUG00015000145

ENSNFUG00015000249

ENSNFUG00015001263

ENSNFUG00015001487

ENSNFUG00015001496

ENSNFUG00015001503

ENSNFUG00015002461

ENSNFUG00015002737

ENSNFUG00015003285

ENSNFUG00015004390

ENSNFUG00015004510

ENSNFUG00015004619

ENSNFUG00015005286

ENSNFUG00015007566

ENSNFUG00015009256

ENSNFUG00015010963

ENSNFUG00015011254

ENSNFUG00015011512

ENSNFUG00015012024

ENSNFUG00015012094

ENSNFUG00015012778

ENSNFUG00015014578

ENSNFUG00015015063

ENSNFUG00015018679

ENSNFUG00015019428

ENSNFUG00015019934

ENSNFUG00015020177

ENSNFUG00015020240

ENSNFUG00015000261

ENSNFUG00015001249

ENSNFUG00015001839

ENSNFUG00015002170

ENSNFUG00015002371

ENSNFUG00015004024

ENSNFUG00015004399

ENSNFUG00015007232

ENSNFUG00015007549

ENSNFUG00015008048

ENSNFUG00015008063

ENSNFUG00015010167

ENSNFUG00015010524

ENSNFUG00015011346

ENSNFUG00015016600

ENSNFUG00015020019

ENSNFUG00015021797

ENSNFUG00015003580

ENSNFUG00015003606

ENSNFUG00015003607

ENSNFUG00015003666

ENSNFUG00015003986

ENSNFUG00015007125

ENSNFUG00015008502

ENSNFUG00015010440

ENSNFUG00015021071

ENSNFUG00015021982

ENSNFUG00015022989

ENSNFUG00015004576

ENSNFUG00015004583

ENSNFUG00015006543

ENSNFUG00015007253

ENSNFUG00015007908

ENSNFUG00015009025

ENSNFUG00015010985

ENSNFUG00015011320

ENSNFUG00015012103

ENSNFUG00015012110

ENSNFUG00015012322

ENSNFUG00015014754

ENSNFUG00015015308

ENSNFUG00015015897

ENSNFUG00015016849

ENSNFUG00015018903

ENSNFUG00015020140

ENSNFUG00015020157

ENSNFUG00015005773

ENSNFUG00015005774

ENSNFUG00015006026

ENSNFUG00015006875

ENSNFUG00015007388

ENSNFUG00015008484

ENSNFUG00015009079

ENSNFUG00015009087

ENSNFUG00015009823

ENSNFUG00015010154

ENSNFUG00015010614

ENSNFUG00015010941

ENSNFUG00015011221

ENSNFUG00015011627

ENSNFUG00015012028

ENSNFUG00015012035

ENSNFUG00015012785

ENSNFUG00015013369

ENSNFUG00015016143

ENSNFUG00015016259

ENSNFUG00015018829

ENSNFUG00015018862

ENSNFUG00015019661

ENSNFUG00015019712

ENSNFUG00015020070

ENSNFUG00015020609

ENSNFUG00015020795

ENSNFUG00015021486

ENSNFUG00015021924

ENSNFUG00015022015

ENSNFUG00015023109

ENSNFUG00015023112

ENSNFUG00015024212

ENSNFUG00015024255

ENSNFUG00015024988

ENSNFUG00015004713

ENSNFUG00015005055

ENSNFUG00015005139

ENSNFUG00015005973

ENSNFUG00015006538

ENSNFUG00015008619

ENSNFUG00015008831

ENSNFUG00015009564

ENSNFUG00015010008

ENSNFUG00015010795

ENSNFUG00015011034

ENSNFUG00015011262

ENSNFUG00015014367

ENSNFUG00015015017

ENSNFUG00015015194

ENSNFUG00015016281

ENSNFUG00015016401

ENSNFUG00015017320

ENSNFUG00015020824

ENSNFUG00015021065

ENSNFUG00015021511

ENSNFUG00015022105

ENSNFUG00015023197

ENSNFUG00015023220

ENSNFUG00015004702

ENSNFUG00015004929

ENSNFUG00015005944

ENSNFUG00015007120

ENSNFUG00015007157

ENSNFUG00015007615

ENSNFUG00015008009

ENSNFUG00015008179

ENSNFUG00015008279

ENSNFUG00015008783

ENSNFUG00015009569

ENSNFUG00015010388

ENSNFUG00015013974

ENSNFUG00015017697

ENSNFUG00015018034

ENSNFUG00015018539

ENSNFUG00015019009

ENSNFUG00015021029

ENSNFUG00015021231

ENSNFUG00015021709

ENSNFUG00015023747

ENSNFUG00015024069

ENSNFUG00015024499

ENSNFUG00015024664

ENSNFUG00015004707

ENSNFUG00015005608

ENSNFUG00015006207

ENSNFUG00015007674

ENSNFUG00015008540

ENSNFUG00015008622

ENSNFUG00015009357

ENSNFUG00015013284

ENSNFUG00015013386

ENSNFUG00015013610

ENSNFUG00015013855

ENSNFUG00015015144

ENSNFUG00015015827

ENSNFUG00015015970

ENSNFUG00015017619

ENSNFUG00015018613

ENSNFUG00015018821

ENSNFUG00015023099

ENSNFUG00015024474

ENSNFUG00015024901

ENSNFUG00015004768

ENSNFUG00015005732

ENSNFUG00015006558

ENSNFUG00015006630

ENSNFUG00015006670

ENSNFUG00015008325

ENSNFUG00015009617

ENSNFUG00015010793

ENSNFUG00015011789

ENSNFUG00015013054

ENSNFUG00015013067

ENSNFUG00015014004

ENSNFUG00015014832

ENSNFUG00015015532

ENSNFUG00015015636

ENSNFUG00015016305

ENSNFUG00015016776

ENSNFUG00015018266

ENSNFUG00015018635

ENSNFUG00015018657

ENSNFUG00015018718

ENSNFUG00015019732

ENSNFUG00015022393

ENSNFUG00015022763

ENSNFUG00015023168

ENSNFUG00015023408

ENSNFUG00015023836

ENSNFUG00015024038

ENSNFUG00015024173

ENSNFUG00015024180

ENSNFUG00015024234

ENSNFUG00015024689

ENSNFUG00015024887

ENSNFUG00015006483

ENSNFUG00015008529

ENSNFUG00015011171

ENSNFUG00015011705

ENSNFUG00015014174

ENSNFUG00015014258

ENSNFUG00015015343

ENSNFUG00015016754

ENSNFUG00015017521

ENSNFUG00015018394

ENSNFUG00015019834

ENSNFUG00015020451

ENSNFUG00015022959

ENSNFUG00015023393

ENSNFUG00015023584

ENSNFUG00015024876

ENSNFUG00015025154

ENSNFUG00015005321

ENSNFUG00015005846

ENSNFUG00015006872

ENSNFUG00015007558

ENSNFUG00015007578

ENSNFUG00015007865

ENSNFUG00015008174

ENSNFUG00015008191

ENSNFUG00015008399

ENSNFUG00015008563

ENSNFUG00015010763

ENSNFUG00015015639

ENSNFUG00015015773

ENSNFUG00015016525

ENSNFUG00015016591

ENSNFUG00015016829

ENSNFUG00015016834

ENSNFUG00015018525

ENSNFUG00015019481

ENSNFUG00015019836

ENSNFUG00015023318

ENSNFUG00015023676

ENSNFUG00015023783

ENSNFUG00015023785

ENSNFUG00015024324

ENSNFUG00015005262

ENSNFUG00015006971

ENSNFUG00015007002

ENSNFUG00015007037

ENSNFUG00015010593

ENSNFUG00015011340

ENSNFUG00015012618

ENSNFUG00015014348

ENSNFUG00015014558

ENSNFUG00015016091

ENSNFUG00015017565

ENSNFUG00015017851

ENSNFUG00015018076

ENSNFUG00015018811

ENSNFUG00015020083

ENSNFUG00015020103

ENSNFUG00015020307

ENSNFUG00015021095

ENSNFUG00015022087

ENSNFUG00015022187

ENSNFUG00015022647

ENSNFUG00015022767

ENSNFUG00015023007

ENSNFUG00015023275

ENSNFUG00015024093

ENSNFUG00015024179

ENSNFUG00015024205

ENSNFUG00015007376

ENSNFUG00015008747

ENSNFUG00015010348

ENSNFUG00015010982

ENSNFUG00015015291

ENSNFUG00015016990

ENSNFUG00015019491

ENSNFUG00015021000

ENSNFUG00015021002

ENSNFUG00015021433

ENSNFUG00015023562

ENSNFUG00015023659

ENSNFUG00015023788

ENSNFUG00015024777

ENSNFUG00015024799

ENSNFUG00015024854

ENSNFUG00015024990

ENSNFUG00015025174

ENSNFUG00015025297

ENSNFUG00015025392

ENSNFUG00015006952

ENSNFUG00015007071

ENSNFUG00015007151

ENSNFUG00015008452

ENSNFUG00015009884

ENSNFUG00015010015

ENSNFUG00015010206

ENSNFUG00015011487

ENSNFUG00015011645

ENSNFUG00015014663

ENSNFUG00015016452

ENSNFUG00015016668

ENSNFUG00015017130

ENSNFUG00015017381

ENSNFUG00015017725

ENSNFUG00015018292

ENSNFUG00015018603

ENSNFUG00015019346

ENSNFUG00015021267

ENSNFUG00015022006

ENSNFUG00015023532

ENSNFUG00015015259

ENSNFUG00015017696

ENSNFUG00015019142

ENSNFUG00015019166

ENSNFUG00015019804

ENSNFUG00015017598

ENSNFUG00015014219

ENSNFUG00015018668

ENSNFUG00015021155

ENSNFUG00015021335

ENSNFUG00015014384

ENSNFUG00015014900

ENSNFUG00015015048

ENSNFUG00015015168

ENSNFUG00015019149

ENSNFUG00015017531

ENSNFUG00015014704

ENSNFUG00015016556

ENSNFUG00015016567

ENSNFUG00015018027

ENSNFUG00015018768

ENSNFUG00015021039

ENSNFUG00015021041

ENSNFUG00015014679

ENSNFUG00015012364

ENSNFUG00015014798

ENSNFUG00015014886

ENSNFUG00015019297

ENSNFUG00015022055

ENSNFUG00015022138

ENSNFUG00015010602

ENSNFUG00015018229

ENSNFUG00015022267

ENSNFUG00015014585

ENSNFUG00015015200

ENSNFUG00015015387

ENSNFUG00015023193

ENSNFUG00015023240

ENSNFUG00015023362

ENSNFUG00015013729

ENSNFUG00015012647

ENSNFUG00015010953

ENSNFUG00015011290

ENSNFUG00015011974

ENSNFUG00015020477

ENSNFUG00015009112

ENSNFUG00015009701

ENSNFUG00015010182

ENSNFUG00015014006

ENSNFUG00015011846

ENSNFUG00015020551

ENSNFUG00015020580

ENSNFUG00015014467

ENSNFUG00015019339

ENSNFUG00015015381

ENSNFUG00015017146

ENSNFUG00015017210

ENSNFUG00015020718

ENSNFUG00015020938

ENSNFUG00015008925

ENSNFUG00015010501

ENSNFUG00015012166

ENSNFUG00015013072

ENSNFUG00015013192

ENSNFUG00015012672

ENSNFUG00015012677

ENSNFUG00015014897

ENSNFUG00015014965

ENSNFUG00015015753

ENSNFUG00015010769

ENSNFUG00015012411

ENSNFUG00015012639

ENSNFUG00015009227

ENSNFUG00015011921

ENSNFUG00015012012

ENSNFUG00015014908

ENSNFUG00015015442

ENSNFUG00015015850

ENSNFUG00015014499

ENSNFUG00015000022

ENSNFUG00015000023

ENSNFUG00015015179

ENSNFUG00015015555

ENSNFUG00015015482

ENSNFUG00015013551

ENSNFUG00015013558

ENSNFUG00015013879

**(G)** **liver_graph.txt**

ENSNFUG00015005973

ENSNFUG00015001875

ENSNFUG00015020077

ENSNFUG00015012785

ENSNFUG00015000759

ENSNFUG00015004576

ENSNFUG00015023318

ENSNFUG00015009231

ENSNFUG00015001509

ENSNFUG00015022816

ENSNFUG00015013104

ENSNFUG00015007257

ENSNFUG00015002864

ENSNFUG00015022746

ENSNFUG00015019454

ENSNFUG00015002243

ENSNFUG00015007233

ENSNFUG00015005194

**(H) R scripts**

**(H-1) logPlot.R**

See Supplementary Figure 8(H-1) logPlot.R (pages 18-19)

**(H-2) counts_to_tpm2.R**

See Supplementary Figure 8(H-1) counts_to_tpm2.R (page 19)

**(H-3) TH.R**

See Supplementary Figure 8(H-1) counts_to_tpm2.R (pages 19-20)

**(H-4) Fig6B.R**

#Correlation coefficients between samples in the gene sets with TPM > 10 in at least one sample.

library(lattice)

library(latticeExtra)

library(gplots)

library(matrixStats)

library(genefilter)

library(dplyr)

my.col1 <- colorRampPalette(c("blue","white","magenta"))

comm <- commandArgs(trailingOnly = T)

head (comm)

infile1 <- comm[1]

outdir <- comm[2]

indata <- read.delim(infile1, header=T, sep="\t")

x<-indata[,3:41]

x<-x+1

x <- as.matrix(x)

log_x<-log(x,base=2)

cor<-cor(log_x)

cor2<-round(cor, digits = 2)

correlationPlot <- function(x) {

   cor<-cor(log_x);

   fig<-levelplot(cor, col.regions=colorRampPalette(c("white","yellow", "red"), space = "rgb")(120), scales=list(x=list(rot=55)), main="TPMlibirary_Correlation plot")

   fig

}

outfile <- paste(outdir, "Fig6B.pdf", sep="/")

pdf(file=outfile,onefile=FALSE,paper="special",height=15,width=15,family="Helvetica",pointsize=15)

correlationPlot(log_x)

dev.off()

**(H-5) DESeq2.R**

#DESeq2

options(warn=1)

options(scipen=100)

options( java.parameters = "-Xmx64g" )

library( DESeq2 )

packageVersion("DESeq2")

library("dplyr")

library(xlsx)

library("genefilter")

library("gplots")

my.col1 <- colorRampPalette(c("blue","white","magenta"))

args <- commandArgs(T)

workDir   <- args[1]

data      <- args[2]

head(data)

name    <- args[3]

gtfFile   <- args[4]

gene_type <- args[5]

subName   <- ifelse(gene_type=="default","",paste0(".",gene_type))

markers   <- c()

TPMdata <- args[6]

newTPMdata<-args[7]

THtpm <- args[8]

outDir    <- file.path(workDir)

outDir

outFile   <- file.path(outDir, paste0("TPM",".xlsx"))

outPCA    <- file.path(outDir, paste0("PCA",subName,".xlsx"))

outBAR    <- file.path(outDir, paste0("barplot",subName,".pdf"))

saveData  <- file.path(outDir, paste0("DESeq2_HISAT2",subName,".Rdata"))

name1 <- read.delim(name,header=F, sep=",")

name1<-as.vector(name1)

Group <- data.frame(con = factor(name1))

head(Group)

typeof(Group)

mode(Group)

class(Group)

data<-read.table(data,header=T,row.names=1,sep="\t")

head(data)

#TPM>10

TPMdata<-read.delim(TPMdata,header=T,sep="\t")

TPMdata1<-as.matrix(TPMdata)

TPMmax10<-TPMdata1[,1]

print(head(TPMdata1))

print(head(TPMmax10))

L1 <- length(TPMmax10)

L1

t5 <- c()

t4 <- subset (data, row.names(data) == TPMmax10[1])

t4 <- t4

for (i in 2:L1) {

  t5 <- subset (data, row.names(data) ==  TPMmax10[i], )

  t4 <- rbind (t4, t5)

}

head(t4)

data<-t4

head(data)

data1<-row.names(data)

data2<-data[6:ncol(data)]

head(data2)

data3<-cbind(data1, data2)

#DEseq2�ŉ��

dds <- DESeqDataSetFromMatrix(countData=data2, colData=Group, design=~ con)

genelength <- matrix( unlist(data [5]), ncol = 1)

mcols(dds)$basepairs <- as.numeric(genelength)

dds <- estimateSizeFactors(dds)

dds <- estimateDispersions(dds)

dds <- nbinomWaldTest(dds)

norm.dt   <- data.frame(counts(dds, normalized=T), check.names=F)

fpkm.dt   <- data.frame(fpkm(dds, robust=F), check.names=F)

tpm.dt    <- data.frame(sweep(fpkm.dt*1e6, 2, colSums(fpkm.dt),"/"), check.names=F)

cpm.dt    <- data.frame(fpm(dds, robust=F), check.names=F)

head(norm.dt)

outfile <- paste(outDir, "DESeq2_results.Rdata", sep="/")

#TPM

wb <- createWorkbook(type="xlsx")

sheet  <- createSheet(wb, sheet="TPM")

row.names(tpm.dt)<-row.names(data)

colnames(tpm.dt)<-name1

addDataFrame(tpm.dt, sheet, row.names=T)

saveWorkbook(wb, file=outFile)

outfile <- paste(outDir, "TPM.txt", sep="/")

write.table(tpm.dt, file=outfile, row.names=T)

dds <- DESeqDataSetFromMatrix(countData=data2, colData=Group, design=~ con)

head(dds)

genelength <- matrix( unlist(data [5]), ncol = 1)

mcols(dds)$basepairs <- as.numeric(genelength)

dds <- DESeq(dds)

head(dds)

res<-results(dds)

head(res)

terms<-c("S14w","S7w","G14w","G7w")

terms2<-combn(terms,2)

terms2

n<-ncol(terms2)

DEGall_FC1_fdr0.05<-c()

DEGall_FC1_fdr0.01<-c()

DEGall_FC2_fdr0.05<-c()

DEGall_FC2_fdr0.01<-c()

DEGall_FC3_fdr0.05<-c()

DEGall_FC3_fdr0.01<-c()

print(resultsNames(dds))

#newTPMdata(TPM>10)

newTPMdata<-read.delim(newTPMdata,header=T,sep="\t")

newTPMdata<-as.matrix(newTPMdata)

L1 <- length(TPMmax10)

L1

#TPM

t5 <- c()

t4 <- subset (newTPMdata, newTPMdata[,1] == TPMmax10[1])

t4 <- t4

for (i in 2:L1) {

  t5 <- subset (newTPMdata, newTPMdata[,1] ==  TPMmax10[i], )

  t4 <- rbind (t4, t5)

}

head(t4)

newTPMdata<-t4

head(newTPMdata)

FC<-log2(1.5)

print(FC)

for (j in 1:n) {

  terms3<-terms2[,j]

  terms4<-append("con",terms3)

  print(terms4)

  res<-results(dds, contrast=terms4)

  res$TPM <-newTPMdata

  tpm<-THtpm

  print(tpm)

  data2 <- as.data.frame(res)

  data2upAll <- subset(data2, (data2$pad<=0.01 & (data2$log2FoldChange>=FC )))

  outfilename <- paste0("DESeq2_result_", terms3[1],"vs",terms3[2],"_TPMmax10_1.5upAll.txt")

  out_f <- paste(outDir, outfilename, sep="/");

  write.table(data2upAll, out_f, sep="\t", append=F, quote=F, row.names=T, col.names=T)

  data2downAll <- subset(data2, (data2$pad<=0.01 & (data2$log2FoldChange<=-FC )))

  outfilename <- paste0("DESeq2_result_", terms3[1],"vs",terms3[2],"_TPMmax10_1.5downAll.txt")

  out_f <- paste(outDir, outfilename, sep="/");

  write.table(data2downAll, out_f, sep="\t", append=F, quote=F, row.names=T, col.names=T)

}

**(H-6) ID-TPMv1.R**

#ID->TPM

comm <- commandArgs(trailingOnly = T)

infile1 <- comm[1]

infile2 <- comm[2]

infile3 <- comm[3]

outdir <- comm[4]

TPM <- read.delim(infile3, sep="\t");

TPM <- as.matrix(TPM)

downcomID <- read.delim(infile1,header=F,sep=",")

downcomID <-as.matrix(downcomID)

downcomID

L1 <- length(downcomID)

L1

#TPM

t5 <- c()

t4 <- subset (TPM, TPM[,1] == downcomID[1])

t4 <- t4

for (i in 2:L1) {

   t5 <- subset (TPM, TPM[,1] ==  downcomID[i], )

   t4 <- rbind (t4, t5)

}

head(t4)

outfile <- paste(outdir, infile2, sep="/")

write.table(t4, file=outfile, sep="\t")

**(H-7) Fig6C.R**

#Correlation coefficients between samples in the gene sets with TPM > 10 in at least one sample.

library(lattice)

library(latticeExtra)

library(gplots)

library(matrixStats)

library(genefilter)

library(dplyr)

my.col1 <- colorRampPalette(c("blue","white","magenta"))

comm <- commandArgs(trailingOnly = T);

head (comm)

infile1 <- comm[1]

outdir <- comm[2]

data <- read.delim(infile1, header=T, sep="\t")

x<-data[,3:41]

x<-x+1

x <- as.matrix(x)

x_a<-x/rowMeans(x)

log_x_a<-log(x_a,base=2)

outfile <- paste(outdir, "Fig6C.pdf", sep="/")

pdf(file=outfile,onefile=FALSE,paper="special",height=15,width=15,family="Helvetica",pointsize=20)

heatmap.2(as.matrix(log_x_a),col=my.col1(269), scale="none",key=TRUE, symm=F,symkey=F,symbreaks=T, breaks=c(seq(-2,2,length=270)), trace="none",distfun = function(x) {dist(x, method="euclidean")},hclustfun = function(x) {hclust(x, method="ward.D2")})

dev.off()

**(H-8) Fig6D.R**

library(beeswarm)

args <- commandArgs(T)

TPMdata <- args[1]

outDir <- args[2]

name <- args[3]

TPMdata <- read.delim(TPMdata, header=T, sep="\t");

TPMdata <- as.matrix(TPMdata)

head(TPMdata)

N<-matrix(NA,nrow(TPMdata),1)

head(N)

print(nrow(TPMdata))

print(nrow(N))

head(TPMdata[,3:22])

head(TPMdata[,23:41])

dx <- cbind(TPMdata[,3:22],N,TPMdata[,23:41])

dx<-as.matrix(dx)

label<-c(1,1,1,1,1,1,1,1,1,1,2,2,2,2,2,2,2,2,2,2,3,3,3,3,3,3,3,3,3,3,4,4,4,4,4,4,4,4,4,4)

X = ceiling(nrow(dx)/30)

X

print(X)

i <- 1

for(i in 1:X){

  print(i)

  filename <- paste0(name,i,".pdf")

  outfile <- paste(outDir, filename, sep="/");

  pdf(file=outfile,onefile=FALSE,paper="special",height=15,width=15,family="Helvetica",pointsize=8)

  H <- ceiling(nrow(dx)/2)

  H

  par(mfrow=c(5,6))

  a<-30*(i-1)+1

  if(i==X){

    b<-nrow(dx)

  }else{

    b<-30*i

  }

  for(j in a:b){

    y1<-c(as.numeric(dx[j,1:10]))

    y2<-c(as.numeric(dx[j,11:20]))

    y3<-c(as.numeric(dx[j,21:30]))

    y4<-c(as.numeric(dx[j,31:40]))

    col1<-c("#00A0E9","#00A0E9","#00A0E9","#00A0E9","#00A0E9","#00A0E9","#00A0E9","#00A0E9","#00A0E9","#00A0E9")

    col2<-c("#FFB2A4", "#FFB2A4", "#FFB2A4", "#FFB2A4","#FFB2A4", "#FFB2A4", "#FFB2A4", "#FFB2A4", "#FFB2A4", "#FFB2A4")

    v1<-cbind.data.frame(y1,col1)

    colnames(v1)<-c("TPM","col")

    v2<-cbind.data.frame(y2,col2)

    colnames(v2)<-c("TPM","col")

    v3<-cbind.data.frame(y3,col1)

    colnames(v3)<-c("TPM","col")

    v4<-cbind.data.frame(y4,col2)

    colnames(v4)<-c("TPM","col")

    y<-rbind.data.frame(v1,v2,v3,v4)

    dx2<-cbind.data.frame(label,y)

    title=TPMdata[j,42]

    print(title)

    max=max(as.numeric(TPMdata[j,c(3:41)]))+10

    if(!is.na(max)){

      beeswarm(TPM~label, data=dx2,pch=16,cex=1.5, yaxs="i", las = 1, xlab = "", cex.lab  = 1.4, cex.axis=1.5, cex.names=1.5, main=title,pwcol=c("#00A0E9","#00A0E9","#00A0E9","#00A0E9","#00A0E9","#00A0E9","#00A0E9","#00A0E9","#00A0E9","#00A0E9","#FFB2A4", "#FFB2A4", "#FFB2A4", "#FFB2A4","#FFB2A4", "#FFB2A4", "#FFB2A4", "#FFB2A4", "#FFB2A4", "#FFB2A4","#00A0E9","#00A0E9","#00A0E9","#00A0E9","#00A0E9","#00A0E9","#00A0E9","#00A0E9","#00A0E9","#00A0E9","#FFB2A4", "#FFB2A4", "#FFB2A4", "#FFB2A4","#FFB2A4", "#FFB2A4", "#FFB2A4", "#FFB2A4", "#FFB2A4", "#FFB2A4"),labels = c("G7","S7","G14","S14"),ylim=c(0,max))

      t1<-as.numeric(dx[j,1:10])

      t2<-as.numeric(dx[j,11:20])

      t3<-as.numeric(dx[j,21:30])

      t4<-as.numeric(dx[j,31:40])

      dx3<-cbind(t1,t2,t3,t4)

      m <- apply(dx3, 2, mean, na.rm = TRUE)

      s <- apply(dx3, 2, sd, na.rm = TRUE)

      b <- c(1,2,3,4)

      b2 <- c(1,2,2,3,3,4,4,5,6,6,7,7)

      arrows (b, m,

              b, m + s,

              length = 0.08,

              angle = 90,

              col=c("#00A0E9","#FFB2A4","#00A0E9","#FFB2A4", "#00A0E9","#FFB2A4",  "#00A0E9", "#FFB2A4"))

      arrows (b, m,

              b, m - s,

              length = 0.08,

              angle = 90,

              col=c("#00A0E9","#FFB2A4","#00A0E9","#FFB2A4", "#00A0E9","#FFB2A4",  "#00A0E9", "#FFB2A4"))

      arrows (b, m,

              b+0.2, m,

              length = 0,

              angle = 90,

              col=c("#00A0E9","#FFB2A4","#00A0E9","#FFB2A4", "#00A0E9","#FFB2A4",  "#00A0E9", "#FFB2A4"))

      arrows (b, m,

              b-0.2, m,

              length = 0,

              angle = 90,

              col=c("#00A0E9","#FFB2A4","#00A0E9","#FFB2A4", "#00A0E9","#FFB2A4",  "#00A0E9", "#FFB2A4"))

    }else{

    }

  }

  dev.off();

}
