# Supplementary material for: Single housing of juveniles accelerates early-stage growth but extends adult lifespan in African turquoise killifish
Source: Aging (Albany NY). 2024 Sep 16;16(18):12443–72. doi: 10.18632/aging.206111 (PMC11466477; doi:10.18632/aging.206111)
Supplement: Supplementary Material 3 [file aging-16-206111-s006.docx]

**Supplementary Material 3. Script for juveniles analysis (Figure 7).**

**(A) Linux commands**

#0 Analysis environment

Ubuntu 18.04.6 LTS

#1. Making directories

workDir=/mnt/d/takahashi/Juveniles

mkdir $workDir

scDir=/mnt/d/takahashi/Juveniles/00_reference

mkdir $scDir&& cd $_

#Copying sample information file to 00_reference

cp /mnt/d/takahashi/sample.csv $_

#Making directories from the information of the sample file

cat sample.csv | awk -F "," '{print $2}'  > filename.txt

cat sample.csv | awk -F "," '{print $3}'  > workDir.txt

paste -d "/" workDir.txt filename.txt > FilenameDir.txt

cat FilenameDir.txt | {

while read dir1

  do

      mkdir -p $dir1 && cd $_

      mkdir 01_raw-seq 02_fastqc 03_valid-seq 04_abundant-check 05_mapping 06_count 07_clustering 08_DESeq2

  done

}

cd $workDir

tree

#2. Making index for HISAT2

cd $scDir

wget ftp://ftp.ensembl.org/pub/release-108/fasta/nothobranchius_furzeri/dna/Nothobranchius_furzeri.Nfu_20140520.dna.toplevel.fa.gz

gzip -d Nothobranchius_furzeri.Nfu_20140520.dna.toplevel.fa.gz

hisat2-build Nothobranchius_furzeri.Nfu_20140520.dna.toplevel.fa Nothobranchius_furzeri.Nfu_20140520.dna.toplevel

cd $scDir

hisat2-build nfu.abundant.fa nfu.abundant

#3-1. Download of Genome Data from Ensembl

cd $scDir

wget ftp://ftp.ensembl.org/pub/release-108/gtf/nothobranchius_furzeri/Nothobranchius_furzeri.Nfu_20140520.108.gtf.gz

gzip -d Nothobranchius_furzeri.Nfu_20140520.108.gtf.gz

#3-2. Making homologues list

#See Supplementary Figure 8(A) Linux commands #3-2. Making homologues list (pages 2-4)

#4. check of raw data in 01_rawdata

workDir=/mnt/d/takahashi/Juveniles

scDir=/mnt/d/takahashi/Juveniles/00_reference

cd $scDir

cat FilenameDir.txt | {

while read dir1

  do

    cd $dir1/01_raw-seq

    for d in `find ./ -type d`;

        do echo $dir1,`ls "$d" | wc -l`;

    done

    md5sum Lib*gz > sum.txt

  done

}

#5. RUN FastQC

workDir=/mnt/d/takahashi/Juveniles

scDir=/mnt/d/takahashi/Juveniles/00_reference

cd $scDir

cat FilenameDir.txt | {

  while read dir1

  do

      fastqc -t 8 --nogroup $dir1/01_raw-seq/Lib*.gz -o $dir1/02_fastqc

      multiqc $dir1/02_fastqc

      mv ./multiqc_report*.html $dir1/02_fastqc/

  done

}

#6. Processing sequence data with Trim galore

workDir=/mnt/d/takahashi/Juveniles

scDir=/mnt/d/takahashi/Juveniles/00_reference

cd $scDir

cat FilenameDir.txt | {

  while read dir1

  do

  Max=$((`find $dir1/01_raw-seq -type f | wc -l`/2))

  for i in `seq -f "%02g" $Max`

  do

  trim_galore -q 30 --length 35 \

  --paired $dir1/01_raw-seq/Lib${i}*R1_001.fastq.gz $dir1/01_raw-seq/Lib${i}*R2_001.fastq.gz \

  -o $dir1/03_valid-seq \

  &> $dir1/03_valid-seq/fastq${i}.trim-galore.log;

  done

  done

}

#7 genome mapping with HISAT2

workDir=/mnt/d/takahashi/Juveniles

scDir=/mnt/d/takahashi/Juveniles/00_reference

cd $scDir

cat FilenameDir.txt | {

  while read dir1

  do

  Max=$((`find $dir1/01_raw-seq -type f | wc -l`/2))

  for i in `seq -f "%02g" $Max`

  do

    hisat2 -p 8 -x $scDir/nfu.abundant \

      -1 $dir1/03_valid-seq/Lib${i}*val_1.fq.gz \

      -2 $dir1/03_valid-seq/Lib${i}*val_2.fq.gz \

      -S $dir1/04_abundant-check/Lib${i}.abundant.sam \

      --no-unal --no-hd \

      2>$dir1/04_abundant-check/Lib${i}.abundant.log;

  done

  done

}

workDir=/mnt/d/takahashi/Juveniles

scDir=/mnt/d/takahashi/Juveniles/00_reference

cd $scDir

cat FilenameDir.txt | {

  while read dir1

  do

  echo -e "library\tchrM\teGFP\tphiX174\trRNA\tmRNA" > $dir1/04_abundant-check/abundant.summary.log;

  Max=$((`find $dir1/01_raw-seq -type f | wc -l`/2))

  echo $Max;

  done

}

cat FilenameDir.txt | {

  while read dir1

  do

  Max=$((`find $dir1/01_raw-seq -type f | wc -l`/2))

  for i in `seq -f "%02g" $Max`

  do

  paste <(echo $dir1/04_abundant-check/Lib${i}) \

  <(grep -c chrM    $dir1/04_abundant-check/Lib${i}.abundant.sam) \

  <(grep -c eGFP    $dir1/04_abundant-check/Lib${i}.abundant.sam) \

  <(grep -c phiX174 $dir1/04_abundant-check/Lib${i}.abundant.sam) \

  <(grep -c EU780557    $dir1/04_abundant-check/Lib${i}.abundant.sam) \

  <(sed -n 3P $dir1/04_abundant-check/Lib${i}.abundant.log | cut -f 5 -d ' ') \

  >> $dir1/04_abundant-check/abundant.summary.log;

  done

  done

}

cat FilenameDir.txt | {

  while read dir1

  do

  R --no-save --args $dir1/04_abundant-check/abundant.summary.log $dir1/04_abundant-check/abundant < $scDir/logPlot.R #H-1 script

  done

}

workDir=/mnt/d/takahashi/Juveniles

scDir=/mnt/d/takahashi/Juveniles/00_reference

cd $scDir

cat FilenameDir.txt | {

  while read dir1

  do

  Max=$((`find $dir1/01_raw-seq -type f | wc -l`/2))

  for i in `seq -f "%02g" $Max`

  do

  hisat2 -p 8 -x $scDir/Nothobranchius_furzeri.Nfu_20140520.dna.toplevel --rna-strandness RF \

  -1 $dir1/03_valid-seq/Lib${i}*val_1.fq.gz -2 $dir1/03_valid-seq/Lib${i}*val_2.fq.gz \

  --dta \

  -S $dir1/05_mapping/Lib${i}.sam 2> $dir1/05_mapping/Lib${i}.mapping.log;

  done

  done

}

workDir=/mnt/d/takahashi/Juveniles

scDir=/mnt/d/takahashi/Juveniles/00_reference

cd $scDir

cat FilenameDir.txt | {

  while read dir1

  do

  Max=$((`find $dir1/01_raw-seq -type f | wc -l`/2))

  for i in `seq -f "%02g" $Max`

  do

  samtools sort -@ 8 $dir1/05_mapping/Lib${i}.sam > $dir1/05_mapping/Lib${i}.bam;

  samtools index -@ 8 $dir1/05_mapping/Lib${i}.bam;

  done

  done

}

workDir=/mnt/d/takahashi/Juveniles

scDir=/mnt/d/takahashi/Juveniles/00_reference

cd $scDir

cat FilenameDir.txt | {

  while read dir1

  do

    echo -e "library\tunmapped\tmulti-mapped\tuniq-mapped" > $dir1/05_mapping/mapping.summary.log;

    Max=$((`find $dir1/01_raw-seq -type f | wc -l`/2))

    for i in `seq -f "%02g" $Max`

    do

        paste <(echo $dir1/05_mapping/Lib${i}) \

        <(sed -n 3P $dir1/05_mapping/Lib${i}.mapping.log | cut -f 5 -d ' ') \

        <(sed -n 5P $dir1/05_mapping/Lib${i}.mapping.log | cut -f 5 -d ' ') \

        <(sed -n 4P $dir1/05_mapping/Lib${i}.mapping.log | cut -f 5 -d ' ') \

        >> $dir1/05_mapping/mapping.summary.log;

    done

    R --no-save --args $dir1/05_mapping/mapping.summary.log $dir1/05_mapping/mapping <  $scDir/logPlot.R #H-1 script

  done

}

#9.Counting with fetureCounts

workDir=/mnt/d/takahashi/Juveniles

scDir=/mnt/d/takahashi/Juveniles/00_reference

cd $scDir

cat FilenameDir.txt | {

    while read dir1

    do

    featureCounts -T 8 -p -B -C -t exon -g gene_id -a $scDir/Nothobranchius_furzeri.Nfu_20140520.108.gtf -o $dir1/06_count/all.featurecounts.txt $dir1/05_mapping/Lib*.bam;

  done

}

#Manually delete the first line of the text file "all.featurecounts.txt",  and rename it to "all.featurecounts2.txt".

workDir=/mnt/d/takahashi/Juveniles

scDir=/mnt/d/takahashi/Juveniles/00_reference

cd $scDir

cat FilenameDir.txt | {

    while read dir1

    do

    R --no-save --args $dir1/06_count/all.featurecounts2.txt $dir1/06_count/ $dir1/06_count/colnames.txt <  $scDir/counts_to_tpm2.R #H-2 script

    R --no-save --args $dir1/06_count/TPMfeaturecounts.txt $dir1/06_count $dir1/06_count/colnames.txt<  $scDir/TH.R #H-3 script

    R --no-save --args /mnt/d/takahashi/blast/ncbi-blast-2.13.0+-src/c++/nfu_zeb_medaka.txt $dir1/06_count/TPMfeaturecounts.txt $dir1/06_count/ <  /mnt/d/takahashi/blast/ncbi-blast-2.13.0+-src/c++/blast3.R #H-11 script

    R --no-save --args $dir1/06_count/TPMfeaturecounts_max10.txt $dir1/06_count  <  $dir1/06_count/Fig7A.R #H-4 script

  done

}

#10. PCA(Fig.7B) & DEseq2 (Supplementary Fig.4-5)

workDir=/mnt/d/takahashi/Juveniles

scDir=/mnt/d/takahashi/Juveniles/00_reference

cd $workDir

cd $scDir

cat FilenameDir.txt | {

  while read dir1

  do

  wdir=$dir1/08_DESeq2

  data=$dir1/06_count/all.featurecounts2.txt

  INDEX=$dir1/08_DESeq2/samplenames.txt

  GTF=$scDir/Nothobranchius_furzeri.Nfu_20140520.108.gtf

  default=default

  TPMdata=$dir1/06_count/TPMfeaturecounts_max10.txt

  newTPMdata=$dir1/06_count/newTPMfeaturecounts2.txt

  THtpm=10

  ID=/mnt/d/takahashi/blast/ncbi-blast-2.13.0+-src/c++/nfu_zeb_medaka.txt

  R --no-save --args $wdir $data $INDEX $GTF defalt $TPMdata $newTPMdata $THtpm $ID < $dir1/08_DESeq2/DESeq2.R #H-5 script

  done

  done

}

#11. Heatmap and clustering of DEGs between stage1/2 and stage6 (Fig.7C)

workDir=/mnt/d/takahashi/Juveniles

scDir=/mnt/d/takahashi/Juveniles/00_reference

cd $workDir

cd $scDir

cat FilenameDir.txt | {

    while read dir1

    do

      R --no-save --args $dir1/07_clustering/commonDEGs.csv commonDEGs-TPM.txt $dir1/06_count/newTPMfeaturecounts2.txt $dir1/07_clustering <  $dir1/07_clustering/ID-TPMv1.R #H-6 script

      R --no-save --args $dir1/07_clustering/commonDEGs-TPM.txt $dir1/07_clustering  <  $dir1/07_clustering/Fig7C.R #H-7 script

    done

}

#12. graphs of the expression change of cell scenesence-related genes (Fig.7D, Supplementary Fig.6)

workDir=/mnt/d/takahashi/Juveniles

scDir=/mnt/d/takahashi/Juveniles/00_reference

cd $workDir

cd $scDir

cat FilenameDir.txt | {

    while read dir1

    do

    R --no-save --args $dir1/06_count/graph.txt graph-TPM.txt $dir1/06_count/newTPMfeaturecounts2.txt $dir1/06_count <  $dir1/07_clustering/ID-TPMv1.R #H-6 script

    R --no-save --args $dir1/06_count/graph-TPM.txt $dir1/06_count graph-TPM# < $dir1/06_count/beeswarm2.R #H-8 script

    done

}

#13. DEGs number (Fig.7E)

workDir=/mnt/d/takahashi/Juveniles

scDir=/mnt/d/takahashi/Juveniles/00_reference

cd $scDir

cat FilenameDir.txt | {

  while read dir1

  do

  wdir=$dir1/08_DESeq2

  data=$dir1/06_count/all.featurecounts2.txt

  INDEX=$dir1/08_DESeq2/samplenames.txt

  GTF=$scDir/Nothobranchius_furzeri.Nfu_20140520.108.gtf

  default=default

  TPMdata=$dir1/06_count/TPMfeaturecounts_max50.txt

  newTPMdata=$dir1/06_count/newTPMfeaturecounts2.txt

  THtpm=50

  R --no-save --args $wdir $data $INDEX $GTF defalt $TPMdata $newTPMdata $THtpm < $dir1/08_DESeq2/DESeq2v4-2.R #H-9 script

  done

}

#14. Heatmap for Fig7F

workDir=/mnt/d/takahashi/Juveniles

scDir=/mnt/d/takahashi/Juveniles/00_reference

cd $workDir

cd $scDir

cat FilenameDir.txt | {

    while read dir1

    do

      wdir=$dir1/08_DESeq2

      data=$dir1/08_DESeq2/DESeq2_result_DEGall_FC4_fdr0.01_TPMmax50.txt

      INDEX=$dir1/08_DESeq2/samplenames.txt

      newTPMdata=$dir1/06_count/newTPMfeaturecounts2.txt

      name="allDEGsFC4_fdr0.01_TPMmax50_TPMcorrelation_All.txt"

      R --no-save --args $wdir $data $INDEX $newTPMdata $name < $scDir/correlation3.R #H-10 script

      R --no-save --args $dir1/08_DESeq2/allDEGsFC4_fdr0.01_TPMmax50_TPMcorrelation_All.txt $dir1/08_DESeq2 <  $dir1/08_DESeq2/Fig7F.R #H-11 script

    done

}

#Supplementary Fig.4-5

workDir=/mnt/d/takahashi/Juveniles

scDir=/mnt/d/takahashi/Juveniles/00_reference

cd $workDir

cd $scDir

cat FilenameDir.txt | {

    while read dir1

    do

    R --no-save --args $dir1/06_count/up.csv up-TPM.txt $dir1/06_count/newTPMfeaturecounts2.txt ./ <  .$dir1/06_count/ID-TPMv1.R #H-6 script

    R --no-save --args $dir1/06_count/down.csv down-TPM.txt $dir1/06_count/newTPMfeaturecounts2.txt ./ <  $dir1/06_count/ID-TPMv1.R #H-6 script

    done

}

#DEGS (S2-4 vs G1-4)_Supplementary Fig.7-8

workDir=/mnt/d/takahashi/Juveniles

scDir=/mnt/d/takahashi/Juveniles/00_reference

cd $scDir

cat FilenameDir.txt | {

  while read dir1

  do

  wdir=$dir1/08_DESeq2

  data=$dir1/06_count/all.featurecounts2.txt

  INDEX=$dir1/08_DESeq2/samplenames2.txt

  GTF=$scDir/Nothobranchius_furzeri.Nfu_20140520.108.gtf

  default=default

  TPMdata=$dir1/06_count/TPMfeaturecounts_max10.txt

  newTPMdata=$dir1/06_count/newTPMfeaturecounts2.txt

  THtpm=10

  ID=/mnt/d/takahashi/blast/ncbi-blast-2.13.0+-src/c++/nfu_zeb_medaka.txt

  R --no-save --args $wdir $data $INDEX $GTF defalt $TPMdata $newTPMdata $THtpm $ID $INDEX2< $dir1/08_DESeq2/DESeq2v6.R #H-12 script

  done

}

**(B) sample.csv**

1,male,/mnt/d/takahashi/Juveniles,

2,female,/mnt/d/takahashi/Juveniles,

**(C) nfu.abundant.fa**

See Supplementary Figure 8(C) nfu.abundant.fa (pages 11-17)

**(D) colnames.txt**

geneID,GeneLength,G9d_1,G9d_2,G9d_3,G9d_4,G13d_1,G13d_2,G13d_3,G13d_4,G17d_1,G17d_2,G17d_3,G17d_4,G21d_1,G21d_2,G21d_3,G21d_4,G28d_1,G28d_2,G28d_3,G28d_4,G35d_1,G35d_2,G35d_3,G35d_4,S9d_1,S9d_2,S9d_3,S9d_4,S10d_1,S10d_2,S10d_3,S10d_4,S11d_1,S11d_2,S11d_3,S11d_4,S17d_1,S17d_2,S17d_3,S17d_4,S21d_1,S21d_2,S21d_3,S21d_4

**(E) samplenames.txt**

(E-1) samplenames.txt

G9d,G9d,G9d,G9d,G13d,G13d,G13d,G13d,G17d,G17d,G17d,G17d,G21d,G21d,G21d,G21d,G28d,G28d,G28d,G28d,G35d,G35d,G35d,G35d,S9d,S9d,S9d,S9d,S10d,S10d,S10d,S10d,S11d,S11d,S11d,S11d,S17d,S17d,S17d,S17d,S21d,S21d,S21d,S21d

(E-2) samplenames2.txt

Gj,Gj,Gj,Gj,Gj,Gj,Gj,Gj,Gj,Gj,Gj,Gj,Gj,Gj,Gj,Gj,G28d,G28d,G28d,G28d,G35d,G35d,G35d,G35d,Sj,Sj,Sj,Sj,Sj,Sj,Sj,Sj,Sj,Sj,Sj,Sj,S17d,S17d,S17d,S17d,S21d,S21d,S21d,S21d

**(F)** **commonDEGs.csv**

**(F-1) male >** **07_clustering > commonDEGs.csv**

ENSNFUG00015013763

ENSNFUG00015016999

ENSNFUG00015004461

ENSNFUG00015011555

ENSNFUG00015007621

ENSNFUG00015016896

ENSNFUG00015020193

ENSNFUG00015017486

ENSNFUG00015006758

ENSNFUG00015018841

ENSNFUG00015010429

ENSNFUG00015013536

ENSNFUG00015009758

ENSNFUG00015010595

ENSNFUG00015006779

ENSNFUG00015017910

ENSNFUG00015010711

ENSNFUG00015025289

ENSNFUG00015013330

ENSNFUG00015012483

ENSNFUG00015006914

ENSNFUG00015003233

ENSNFUG00015001595

ENSNFUG00015013426

ENSNFUG00015013464

ENSNFUG00015003476

ENSNFUG00015016057

ENSNFUG00015012290

ENSNFUG00015019695

ENSNFUG00015003981

ENSNFUG00015003776

ENSNFUG00015025307

ENSNFUG00015000894

ENSNFUG00015008466

ENSNFUG00015023790

ENSNFUG00015016486

ENSNFUG00015014629

ENSNFUG00015007153

ENSNFUG00015021139

ENSNFUG00015008878

ENSNFUG00015004991

ENSNFUG00015014299

ENSNFUG00015020269

ENSNFUG00015020573

ENSNFUG00015001304

ENSNFUG00015009909

ENSNFUG00015021422

ENSNFUG00015005810

ENSNFUG00015004739

ENSNFUG00015002339

ENSNFUG00015018028

ENSNFUG00015006628

ENSNFUG00015021545

ENSNFUG00015008767

ENSNFUG00015010722

ENSNFUG00015015054

ENSNFUG00015020032

ENSNFUG00015018114

ENSNFUG00015008089

ENSNFUG00015010862

ENSNFUG00015000800

ENSNFUG00015007052

ENSNFUG00015007574

ENSNFUG00015023555

ENSNFUG00015007657

ENSNFUG00015018780

ENSNFUG00015010818

ENSNFUG00015007832

ENSNFUG00015024586

ENSNFUG00015025049

ENSNFUG00015015333

ENSNFUG00015011388

ENSNFUG00015014135

ENSNFUG00015005872

ENSNFUG00015002229

ENSNFUG00015022509

ENSNFUG00015022812

ENSNFUG00015021366

ENSNFUG00015006905

ENSNFUG00015007443

ENSNFUG00015017825

ENSNFUG00015021543

ENSNFUG00015017867

ENSNFUG00015021546

ENSNFUG00015025279

ENSNFUG00015018989

ENSNFUG00015007370

ENSNFUG00015022400

ENSNFUG00015015479

ENSNFUG00015023021

ENSNFUG00015004017

ENSNFUG00015007595

ENSNFUG00015016689

ENSNFUG00015015449

ENSNFUG00015024742

ENSNFUG00015000547

ENSNFUG00015016621

ENSNFUG00015023557

ENSNFUG00015018687

ENSNFUG00015024735

ENSNFUG00015010772

ENSNFUG00015021921

ENSNFUG00015020816

ENSNFUG00015010844

ENSNFUG00015020525

ENSNFUG00015012293

ENSNFUG00015003894

ENSNFUG00015000041

ENSNFUG00015022100

ENSNFUG00015014569

ENSNFUG00015020545

ENSNFUG00015007841

ENSNFUG00015010245

ENSNFUG00015003143

ENSNFUG00015018291

ENSNFUG00015022168

ENSNFUG00015012345

ENSNFUG00015024441

ENSNFUG00015003973

ENSNFUG00015007919

ENSNFUG00015021264

ENSNFUG00015003036

ENSNFUG00015000814

ENSNFUG00015013595

ENSNFUG00015002566

ENSNFUG00015007656

ENSNFUG00015020984

ENSNFUG00015007630

ENSNFUG00015009959

ENSNFUG00015018650

ENSNFUG00015016492

ENSNFUG00015024320

ENSNFUG00015007611

ENSNFUG00015020333

ENSNFUG00015018786

ENSNFUG00015018686

ENSNFUG00015001018

ENSNFUG00015009627

ENSNFUG00015014701

ENSNFUG00015002319

ENSNFUG00015021358

ENSNFUG00015007590

ENSNFUG00015015003

ENSNFUG00015006859

ENSNFUG00015020348

ENSNFUG00015003964

ENSNFUG00015003976

ENSNFUG00015025432

ENSNFUG00015020809

ENSNFUG00015010160

ENSNFUG00015018651

ENSNFUG00015002083

ENSNFUG00015006413

ENSNFUG00015020256

ENSNFUG00015022933

ENSNFUG00015007213

ENSNFUG00015015838

ENSNFUG00015018722

ENSNFUG00015002697

ENSNFUG00015006801

ENSNFUG00015017802

ENSNFUG00015007588

ENSNFUG00015011925

ENSNFUG00015018234

ENSNFUG00015009255

ENSNFUG00015010960

ENSNFUG00015011241

ENSNFUG00015006757

ENSNFUG00015010085

ENSNFUG00015017471

ENSNFUG00015007564

ENSNFUG00015007691

ENSNFUG00015017780

ENSNFUG00015019128

ENSNFUG00015009349

ENSNFUG00015003574

ENSNFUG00015014768

ENSNFUG00015018188

ENSNFUG00015019234

ENSNFUG00015015059

ENSNFUG00015004588

ENSNFUG00015023064

ENSNFUG00015021686

ENSNFUG00015017985

ENSNFUG00015014159

ENSNFUG00015016044

ENSNFUG00015007720

ENSNFUG00015021094

ENSNFUG00015022729

ENSNFUG00015015495

ENSNFUG00015013469

ENSNFUG00015010294

ENSNFUG00015014325

ENSNFUG00015010908

ENSNFUG00015021289

ENSNFUG00015006246

ENSNFUG00015020047

ENSNFUG00015009729

ENSNFUG00015024043

ENSNFUG00015021222

ENSNFUG00015004893

ENSNFUG00015005255

ENSNFUG00015012982

ENSNFUG00015007833

ENSNFUG00015017761

ENSNFUG00015022621

ENSNFUG00015018152

ENSNFUG00015015941

ENSNFUG00015007651

ENSNFUG00015007939

ENSNFUG00015007592

ENSNFUG00015011078

ENSNFUG00015021667

ENSNFUG00015012237

ENSNFUG00015013044

ENSNFUG00015010666

ENSNFUG00015005871

ENSNFUG00015008312

ENSNFUG00015010229

ENSNFUG00015015819

ENSNFUG00015001966

ENSNFUG00015021535

ENSNFUG00015016322

ENSNFUG00015023558

ENSNFUG00015007717

ENSNFUG00015010142

ENSNFUG00015000712

ENSNFUG00015015766

ENSNFUG00015006360

ENSNFUG00015022978

ENSNFUG00015023198

ENSNFUG00015015630

ENSNFUG00015006172

ENSNFUG00015023977

ENSNFUG00015009586

ENSNFUG00015012502

ENSNFUG00015021052

ENSNFUG00015016470

ENSNFUG00015023940

ENSNFUG00015012612

ENSNFUG00015007273

ENSNFUG00015016793

ENSNFUG00015000204

ENSNFUG00015009805

ENSNFUG00015000743

ENSNFUG00015002021

ENSNFUG00015007208

ENSNFUG00015017664

ENSNFUG00015001213

ENSNFUG00015000767

ENSNFUG00015005140

ENSNFUG00015010698

ENSNFUG00015024394

ENSNFUG00015009494

ENSNFUG00015006143

ENSNFUG00015022811

ENSNFUG00015018080

ENSNFUG00015012115

ENSNFUG00015011926

ENSNFUG00015019779

ENSNFUG00015011785

ENSNFUG00015024512

ENSNFUG00015003400

ENSNFUG00015005520

ENSNFUG00015023553

ENSNFUG00015013279

ENSNFUG00015010883

ENSNFUG00015018852

ENSNFUG00015018551

ENSNFUG00015007594

ENSNFUG00015014747

ENSNFUG00015017417

ENSNFUG00015025051

ENSNFUG00015011382

ENSNFUG00015012768

ENSNFUG00015001682

ENSNFUG00015013421

ENSNFUG00015001043

ENSNFUG00015000514

ENSNFUG00015006722

ENSNFUG00015007568

ENSNFUG00015005495

ENSNFUG00015022596

ENSNFUG00015012084

ENSNFUG00015000103

ENSNFUG00015009064

ENSNFUG00015025156

ENSNFUG00015013089

ENSNFUG00015017323

ENSNFUG00015011193

ENSNFUG00015001736

ENSNFUG00015020829

ENSNFUG00015012839

ENSNFUG00015014981

ENSNFUG00015023739

ENSNFUG00015004926

ENSNFUG00015023611

ENSNFUG00015011872

ENSNFUG00015023232

ENSNFUG00015003709

ENSNFUG00015004887

ENSNFUG00015024066

ENSNFUG00015018058

ENSNFUG00015012295

ENSNFUG00015003128

ENSNFUG00015017740

ENSNFUG00015016531

ENSNFUG00015007102

ENSNFUG00015008728

ENSNFUG00015014522

ENSNFUG00015018635

ENSNFUG00015000562

ENSNFUG00015004381

ENSNFUG00015010809

ENSNFUG00015022988

ENSNFUG00015024522

ENSNFUG00015003569

ENSNFUG00015008169

ENSNFUG00015008993

ENSNFUG00015005275

ENSNFUG00015013458

ENSNFUG00015020979

ENSNFUG00015019050

ENSNFUG00015017823

ENSNFUG00015023270

ENSNFUG00015018972

ENSNFUG00015002106

ENSNFUG00015015295

ENSNFUG00015001718

ENSNFUG00015000835

ENSNFUG00015016626

ENSNFUG00015019962

ENSNFUG00015016800

ENSNFUG00015024296

ENSNFUG00015014977

ENSNFUG00015014155

ENSNFUG00015011020

ENSNFUG00015014769

ENSNFUG00015007729

ENSNFUG00015021622

ENSNFUG00015009227

ENSNFUG00015010015

ENSNFUG00015017938

ENSNFUG00015017520

ENSNFUG00015012669

ENSNFUG00015020147

ENSNFUG00015013077

ENSNFUG00015010155

ENSNFUG00015019285

ENSNFUG00015019852

ENSNFUG00015022487

ENSNFUG00015023235

ENSNFUG00015010050

ENSNFUG00015004919

ENSNFUG00015005554

ENSNFUG00015017198

ENSNFUG00015024401

ENSNFUG00015017012

ENSNFUG00015008591

ENSNFUG00015022208

ENSNFUG00015022246

ENSNFUG00015024322

ENSNFUG00015010600

ENSNFUG00015022293

ENSNFUG00015002592

ENSNFUG00015013958

ENSNFUG00015013216

ENSNFUG00015009242

ENSNFUG00015019137

ENSNFUG00015011177

ENSNFUG00015024040

ENSNFUG00015019646

ENSNFUG00015010888

ENSNFUG00015011974

ENSNFUG00015011171

ENSNFUG00015007826

ENSNFUG00015000477

ENSNFUG00015002002

ENSNFUG00015008952

ENSNFUG00015003372

ENSNFUG00015019943

ENSNFUG00015012496

ENSNFUG00015001575

ENSNFUG00015018903

ENSNFUG00015000820

ENSNFUG00015000336

ENSNFUG00015019268

ENSNFUG00015006598

ENSNFUG00015005758

ENSNFUG00015013910

ENSNFUG00015000759

ENSNFUG00015021179

ENSNFUG00015014349

ENSNFUG00015017725

ENSNFUG00015004344

ENSNFUG00015024015

ENSNFUG00015017497

ENSNFUG00015018297

ENSNFUG00015020995

ENSNFUG00015004714

ENSNFUG00015020088

ENSNFUG00015024316

ENSNFUG00015011825

ENSNFUG00015002174

ENSNFUG00015003845

ENSNFUG00015022351

ENSNFUG00015003457

ENSNFUG00015020938

ENSNFUG00015023535

ENSNFUG00015020580

ENSNFUG00015013192

ENSNFUG00015013764

ENSNFUG00015019830

ENSNFUG00015022641

ENSNFUG00015015249

ENSNFUG00015003267

ENSNFUG00015003458

ENSNFUG00015011022

ENSNFUG00015003379

ENSNFUG00015008974

ENSNFUG00015000252

ENSNFUG00015013485

ENSNFUG00015016228

ENSNFUG00015000071

ENSNFUG00015021900

ENSNFUG00015017257

ENSNFUG00015021811

ENSNFUG00015017546

ENSNFUG00015016320

ENSNFUG00015012644

ENSNFUG00015004411

ENSNFUG00015003461

ENSNFUG00015007083

ENSNFUG00015012322

ENSNFUG00015000043

ENSNFUG00015013454

ENSNFUG00015001941

ENSNFUG00015007612

ENSNFUG00015019771

ENSNFUG00015010961

ENSNFUG00015014257

ENSNFUG00015019454

ENSNFUG00015004311

ENSNFUG00015014686

ENSNFUG00015017397

ENSNFUG00015003306

ENSNFUG00015016383

ENSNFUG00015007541

ENSNFUG00015019311

ENSNFUG00015004136

ENSNFUG00015014696

ENSNFUG00015009928

ENSNFUG00015007233

ENSNFUG00015010644

ENSNFUG00015019893

ENSNFUG00015004973

ENSNFUG00015018689

ENSNFUG00015000143

ENSNFUG00015001899

ENSNFUG00015014758

ENSNFUG00015014962

ENSNFUG00015006338

ENSNFUG00015002610

ENSNFUG00015020489

ENSNFUG00015003384

ENSNFUG00015000471

ENSNFUG00015013643

ENSNFUG00015021065

ENSNFUG00015017533

ENSNFUG00015003180

ENSNFUG00015012094

ENSNFUG00015011056

ENSNFUG00015019950

ENSNFUG00015013926

ENSNFUG00015014709

ENSNFUG00015014308

ENSNFUG00015020740

ENSNFUG00015023269

ENSNFUG00015003513

ENSNFUG00015000269

ENSNFUG00015025287

ENSNFUG00015014400

ENSNFUG00015023325

ENSNFUG00015013605

ENSNFUG00015015485

ENSNFUG00015017565

ENSNFUG00015000503

ENSNFUG00015003847

ENSNFUG00015003606

ENSNFUG00015022180

ENSNFUG00015021141

ENSNFUG00015010993

ENSNFUG00015018236

ENSNFUG00015007820

ENSNFUG00015006015

ENSNFUG00015007824

ENSNFUG00015018290

ENSNFUG00015022953

ENSNFUG00015010138

ENSNFUG00015005386

ENSNFUG00015016280

ENSNFUG00015014552

ENSNFUG00015021417

ENSNFUG00015022234

ENSNFUG00015018952

ENSNFUG00015016857

ENSNFUG00015009050

ENSNFUG00015014743

ENSNFUG00015023543

ENSNFUG00015007277

ENSNFUG00015008534

ENSNFUG00015022282

ENSNFUG00015009233

ENSNFUG00015003039

ENSNFUG00015005936

ENSNFUG00015003110

ENSNFUG00015001761

ENSNFUG00015003502

ENSNFUG00015001594

ENSNFUG00015023749

ENSNFUG00015012689

ENSNFUG00015006349

ENSNFUG00015019915

ENSNFUG00015007783

ENSNFUG00015021329

ENSNFUG00015001053

ENSNFUG00015005532

ENSNFUG00015013589

ENSNFUG00015019996

ENSNFUG00015011805

ENSNFUG00015020168

ENSNFUG00015017418

ENSNFUG00015009099

ENSNFUG00015016004

ENSNFUG00015003282

ENSNFUG00015013378

ENSNFUG00015022893

ENSNFUG00015005265

ENSNFUG00015012314

ENSNFUG00015009085

ENSNFUG00015017902

ENSNFUG00015014865

ENSNFUG00015014942

ENSNFUG00015003376

ENSNFUG00015007142

ENSNFUG00015013492

ENSNFUG00015014585

ENSNFUG00015012005

ENSNFUG00015015642

ENSNFUG00015018946

ENSNFUG00015001183

ENSNFUG00015009493

ENSNFUG00015000270

ENSNFUG00015020177

ENSNFUG00015014679

ENSNFUG00015018409

ENSNFUG00015015442

ENSNFUG00015000508

ENSNFUG00015006164

ENSNFUG00015002590

ENSNFUG00015025172

ENSNFUG00015008971

ENSNFUG00015003471

ENSNFUG00015001558

ENSNFUG00015013505

ENSNFUG00015014725

ENSNFUG00015024390

ENSNFUG00015005742

ENSNFUG00015011184

ENSNFUG00015003382

ENSNFUG00015011034

ENSNFUG00015000240

ENSNFUG00015003447

ENSNFUG00015003177

ENSNFUG00015007666

ENSNFUG00015020583

ENSNFUG00015018054

ENSNFUG00015016245

ENSNFUG00015016503

ENSNFUG00015002154

ENSNFUG00015014995

ENSNFUG00015016849

ENSNFUG00015005415

ENSNFUG00015012942

ENSNFUG00015024552

ENSNFUG00015002464

ENSNFUG00015003851

ENSNFUG00015016281

ENSNFUG00015021709

ENSNFUG00015013245

ENSNFUG00015013219

ENSNFUG00015008380

ENSNFUG00015022329

ENSNFUG00015023343

ENSNFUG00015022759

ENSNFUG00015008655

ENSNFUG00015001391

ENSNFUG00015016260

ENSNFUG00015023009

ENSNFUG00015016739

ENSNFUG00015007755

ENSNFUG00015004407

ENSNFUG00015008299

ENSNFUG00015015383

ENSNFUG00015017177

ENSNFUG00015004258

ENSNFUG00015008015

ENSNFUG00015017627

ENSNFUG00015018748

ENSNFUG00015006867

ENSNFUG00015004277

ENSNFUG00015013516

ENSNFUG00015019537

ENSNFUG00015002715

ENSNFUG00015017001

ENSNFUG00015002839

ENSNFUG00015014614

ENSNFUG00015024014

ENSNFUG00015009731

ENSNFUG00015023275

ENSNFUG00015016616

ENSNFUG00015018960

ENSNFUG00015016401

ENSNFUG00015011644

ENSNFUG00015020695

ENSNFUG00015003607

ENSNFUG00015010424

ENSNFUG00015022793

ENSNFUG00015015870

ENSNFUG00015014856

ENSNFUG00015012298

ENSNFUG00015007606

ENSNFUG00015010583

ENSNFUG00015018292

ENSNFUG00015022858

ENSNFUG00015023298

ENSNFUG00015024882

ENSNFUG00015020063

ENSNFUG00015011347

ENSNFUG00015001487

ENSNFUG00015017681

ENSNFUG00015024506

ENSNFUG00015001540

ENSNFUG00015001496

ENSNFUG00015020824

ENSNFUG00015001503

ENSNFUG00015022354

ENSNFUG00015013610

ENSNFUG00015012931

ENSNFUG00015019444

ENSNFUG00015002376

ENSNFUG00015000248

ENSNFUG00015008831

ENSNFUG00015016997

ENSNFUG00015017623

ENSNFUG00015019552

ENSNFUG00015015117

ENSNFUG00015011727

ENSNFUG00015013974

ENSNFUG00015021245

ENSNFUG00015024760

ENSNFUG00015008429

ENSNFUG00015009007

ENSNFUG00015005016

ENSNFUG00015011389

ENSNFUG00015013054

ENSNFUG00015006281

ENSNFUG00015016124

ENSNFUG00015001604

ENSNFUG00015023344

ENSNFUG00015008636

ENSNFUG00015001340

ENSNFUG00015022868

ENSNFUG00015010982

ENSNFUG00015020115

ENSNFUG00015008007

ENSNFUG00015009087

ENSNFUG00015023887

ENSNFUG00015014336

ENSNFUG00015021231

ENSNFUG00015018774

ENSNFUG00015021039

ENSNFUG00015022162

ENSNFUG00015022764

ENSNFUG00015024791

ENSNFUG00015021268

ENSNFUG00015021183

ENSNFUG00015010677

ENSNFUG00015012600

ENSNFUG00015006993

ENSNFUG00015004493

ENSNFUG00015015179

ENSNFUG00015007130

ENSNFUG00015000591

ENSNFUG00015000476

ENSNFUG00015003917

ENSNFUG00015018285

ENSNFUG00015004704

ENSNFUG00015004413

ENSNFUG00015017907

ENSNFUG00015003140

ENSNFUG00015008440

ENSNFUG00015018041

ENSNFUG00015018700

ENSNFUG00015003602

ENSNFUG00015017607

ENSNFUG00015002150

ENSNFUG00015022838

ENSNFUG00015004227

ENSNFUG00015007736

ENSNFUG00015009781

ENSNFUG00015005351

ENSNFUG00015003877

ENSNFUG00015007598

**(F-2) female >** **07_clustering > commonDEGs.csv**

ENSNFUG00015004461

ENSNFUG00015011555

ENSNFUG00015015154

ENSNFUG00015007621

ENSNFUG00015016896

ENSNFUG00015020193

ENSNFUG00015004896

ENSNFUG00015018841

ENSNFUG00015010429

ENSNFUG00015009758

ENSNFUG00015010595

ENSNFUG00015006779

ENSNFUG00015010711

ENSNFUG00015005422

ENSNFUG00015015303

ENSNFUG00015025289

ENSNFUG00015012483

ENSNFUG00015010661

ENSNFUG00015006914

ENSNFUG00015001595

ENSNFUG00015003233

ENSNFUG00015003476

ENSNFUG00015007216

ENSNFUG00015016057

ENSNFUG00015015851

ENSNFUG00015012290

ENSNFUG00015003776

ENSNFUG00015016486

ENSNFUG00015014629

ENSNFUG00015021139

ENSNFUG00015000936

ENSNFUG00015014299

ENSNFUG00015020269

ENSNFUG00015020573

ENSNFUG00015001304

ENSNFUG00015021422

ENSNFUG00015014636

ENSNFUG00015002339

ENSNFUG00015002701

ENSNFUG00015018028

ENSNFUG00015021545

ENSNFUG00015008767

ENSNFUG00015010722

ENSNFUG00015015054

ENSNFUG00015022403

ENSNFUG00015008089

ENSNFUG00015010862

ENSNFUG00015007574

ENSNFUG00015023555

ENSNFUG00015007657

ENSNFUG00015010818

ENSNFUG00015024586

ENSNFUG00015025049

ENSNFUG00015015333

ENSNFUG00015011388

ENSNFUG00015014135

ENSNFUG00015005872

ENSNFUG00015017831

ENSNFUG00015002229

ENSNFUG00015022812

ENSNFUG00015021366

ENSNFUG00015006905

ENSNFUG00015007443

ENSNFUG00015021543

ENSNFUG00015021546

ENSNFUG00015025279

ENSNFUG00015018989

ENSNFUG00015015954

ENSNFUG00015007595

ENSNFUG00015015449

ENSNFUG00015016689

ENSNFUG00015016621

ENSNFUG00015023557

ENSNFUG00015018687

ENSNFUG00015024735

ENSNFUG00015020816

ENSNFUG00015010844

ENSNFUG00015020525

ENSNFUG00015012293

ENSNFUG00015014569

ENSNFUG00015020545

ENSNFUG00015007841

ENSNFUG00015010245

ENSNFUG00015003143

ENSNFUG00015003973

ENSNFUG00015007919

ENSNFUG00015021264

ENSNFUG00015003036

ENSNFUG00015000814

ENSNFUG00015007656

ENSNFUG00015024320

ENSNFUG00015016492

ENSNFUG00015018650

ENSNFUG00015007611

ENSNFUG00015018686

ENSNFUG00015009627

ENSNFUG00015017960

ENSNFUG00015014701

ENSNFUG00015002319

ENSNFUG00015007590

ENSNFUG00015006859

ENSNFUG00015015003

ENSNFUG00015003964

ENSNFUG00015003976

ENSNFUG00015010160

ENSNFUG00015023985

ENSNFUG00015002083

ENSNFUG00015020256

ENSNFUG00015007213

ENSNFUG00015003686

ENSNFUG00015002697

ENSNFUG00015015033

ENSNFUG00015007588

ENSNFUG00015011925

ENSNFUG00015010960

ENSNFUG00015018977

ENSNFUG00015006757

ENSNFUG00015010085

ENSNFUG00015007564

ENSNFUG00015007691

ENSNFUG00015009349

ENSNFUG00015011567

ENSNFUG00015018188

ENSNFUG00015019234

ENSNFUG00015015059

ENSNFUG00015004588

ENSNFUG00015023064

ENSNFUG00015016044

ENSNFUG00015007720

ENSNFUG00015011844

ENSNFUG00015021094

ENSNFUG00015022729

ENSNFUG00015013469

ENSNFUG00015010294

ENSNFUG00015021289

ENSNFUG00015006246

ENSNFUG00015009729

ENSNFUG00015024043

ENSNFUG00015021665

ENSNFUG00015005255

ENSNFUG00015007833

ENSNFUG00015022621

ENSNFUG00015018152

ENSNFUG00015015941

ENSNFUG00015007651

ENSNFUG00015007939

ENSNFUG00015007592

ENSNFUG00015011078

ENSNFUG00015012237

ENSNFUG00015021667

ENSNFUG00015013044

ENSNFUG00015010666

ENSNFUG00015005871

ENSNFUG00015008312

ENSNFUG00015001966

ENSNFUG00015016322

ENSNFUG00015023558

ENSNFUG00015016842

ENSNFUG00015007717

ENSNFUG00015010142

ENSNFUG00015000712

ENSNFUG00015002658

ENSNFUG00015015766

ENSNFUG00015015630

ENSNFUG00015009586

ENSNFUG00015012502

ENSNFUG00015021551

ENSNFUG00015012612

ENSNFUG00015007273

ENSNFUG00015016793

ENSNFUG00015000204

ENSNFUG00015013084

ENSNFUG00015009805

ENSNFUG00015002021

ENSNFUG00015009310

ENSNFUG00015007208

ENSNFUG00015017664

ENSNFUG00015000767

ENSNFUG00015005140

ENSNFUG00015010698

ENSNFUG00015009494

ENSNFUG00015012115

ENSNFUG00015018080

ENSNFUG00015019779

ENSNFUG00015011785

ENSNFUG00015003400

ENSNFUG00015005520

ENSNFUG00015013279

ENSNFUG00015018852

ENSNFUG00015007594

ENSNFUG00015025051

ENSNFUG00015001682

ENSNFUG00015013421

ENSNFUG00015000514

ENSNFUG00015006722

ENSNFUG00015007568

ENSNFUG00015012084

ENSNFUG00015000103

ENSNFUG00015014448

ENSNFUG00015017323

ENSNFUG00015011193

ENSNFUG00015001736

ENSNFUG00015020829

ENSNFUG00015012839

ENSNFUG00015014981

ENSNFUG00015004926

ENSNFUG00015023611

ENSNFUG00015011872

ENSNFUG00015003709

ENSNFUG00015004887

ENSNFUG00015024066

ENSNFUG00015009203

ENSNFUG00015018058

ENSNFUG00015012295

ENSNFUG00015003128

ENSNFUG00015002655

ENSNFUG00015014522

ENSNFUG00015022988

ENSNFUG00015024522

ENSNFUG00015003794

ENSNFUG00015013458

ENSNFUG00015019050

ENSNFUG00015023270

ENSNFUG00015002106

ENSNFUG00015022896

ENSNFUG00015007626

ENSNFUG00015007589

ENSNFUG00015001144

ENSNFUG00015006328

ENSNFUG00015002395

ENSNFUG00015008398

ENSNFUG00015005356

ENSNFUG00015022904

ENSNFUG00015002872

ENSNFUG00015018108

ENSNFUG00015022217

ENSNFUG00015018351

ENSNFUG00015019621

ENSNFUG00015006496

ENSNFUG00015008045

ENSNFUG00015000835

ENSNFUG00015019962

ENSNFUG00015016800

ENSNFUG00015024296

ENSNFUG00015011020

ENSNFUG00015014769

ENSNFUG00015007729

ENSNFUG00015023207

ENSNFUG00015022599

ENSNFUG00015022911

ENSNFUG00015020147

ENSNFUG00015019285

ENSNFUG00015010050

ENSNFUG00015005554

ENSNFUG00015024401

ENSNFUG00015015639

ENSNFUG00015013958

ENSNFUG00015013216

ENSNFUG00015009242

ENSNFUG00015019137

ENSNFUG00015011177

ENSNFUG00015024040

ENSNFUG00015019646

ENSNFUG00015011974

ENSNFUG00015005176

ENSNFUG00015008375

ENSNFUG00015019943

ENSNFUG00015005048

ENSNFUG00015001748

ENSNFUG00015000336

ENSNFUG00015010952

ENSNFUG00015004984

ENSNFUG00015006598

ENSNFUG00015013910

ENSNFUG00015003488

ENSNFUG00015000759

ENSNFUG00015021179

ENSNFUG00015009228

ENSNFUG00015014349

ENSNFUG00015024114

ENSNFUG00015020699

ENSNFUG00015020995

ENSNFUG00015025381

ENSNFUG00015005137

ENSNFUG00015020088

ENSNFUG00015000694

ENSNFUG00015016101

ENSNFUG00015022351

ENSNFUG00015015120

ENSNFUG00015009961

ENSNFUG00015008063

ENSNFUG00015020580

ENSNFUG00015013192

ENSNFUG00015013764

ENSNFUG00015003458

ENSNFUG00015000252

ENSNFUG00015000071

ENSNFUG00015001518

ENSNFUG00015002707

ENSNFUG00015021811

ENSNFUG00015012576

ENSNFUG00015011710

ENSNFUG00015004411

ENSNFUG00015003772

ENSNFUG00015001227

ENSNFUG00015007083

ENSNFUG00015018379

ENSNFUG00015001941

ENSNFUG00015019771

ENSNFUG00015007612

ENSNFUG00015002576

ENSNFUG00015019454

ENSNFUG00015004311

ENSNFUG00015006003

ENSNFUG00015017397

ENSNFUG00015016383

ENSNFUG00015007541

ENSNFUG00015004136

ENSNFUG00015010247

ENSNFUG00015014696

ENSNFUG00015002311

ENSNFUG00015014758

ENSNFUG00015002610

ENSNFUG00015020489

ENSNFUG00015000471

ENSNFUG00015017533

ENSNFUG00015003180

ENSNFUG00015024254

ENSNFUG00015014709

ENSNFUG00015014308

ENSNFUG00015009031

ENSNFUG00015013530

ENSNFUG00015003513

ENSNFUG00015015259

ENSNFUG00015014400

ENSNFUG00015019534

ENSNFUG00015006636

ENSNFUG00015003606

ENSNFUG00015021141

ENSNFUG00015011169

ENSNFUG00015002890

ENSNFUG00015010993

ENSNFUG00015019289

ENSNFUG00015006158

ENSNFUG00015007824

ENSNFUG00015018290

ENSNFUG00015022953

ENSNFUG00015010138

ENSNFUG00015005386

ENSNFUG00015014552

ENSNFUG00015018454

ENSNFUG00015021417

ENSNFUG00015016857

ENSNFUG00015023543

ENSNFUG00015008255

ENSNFUG00015022282

ENSNFUG00015003039

ENSNFUG00015014081

ENSNFUG00015003502

ENSNFUG00015007925

ENSNFUG00015020694

ENSNFUG00015021943

ENSNFUG00015015115

ENSNFUG00015021329

ENSNFUG00015001053

ENSNFUG00015017418

ENSNFUG00015016004

ENSNFUG00015024487

ENSNFUG00015003282

ENSNFUG00015005265

ENSNFUG00015021229

ENSNFUG00015017902

ENSNFUG00015008747

ENSNFUG00015014942

ENSNFUG00015003376

ENSNFUG00015013492

ENSNFUG00015007142

ENSNFUG00015014585

ENSNFUG00015006747

ENSNFUG00015012005

ENSNFUG00015023097

ENSNFUG00015009493

ENSNFUG00015021804

ENSNFUG00015020177

ENSNFUG00015015442

ENSNFUG00015025017

ENSNFUG00015002590

ENSNFUG00015011657

ENSNFUG00015013505

ENSNFUG00015014725

ENSNFUG00015024390

ENSNFUG00015020697

ENSNFUG00015005742

ENSNFUG00015007666

ENSNFUG00015018054

ENSNFUG00015014995

ENSNFUG00015005415

ENSNFUG00015016202

ENSNFUG00015013132

ENSNFUG00015021002

ENSNFUG00015008032

ENSNFUG00015024552

ENSNFUG00015025346

ENSNFUG00015016281

ENSNFUG00015013219

ENSNFUG00015023343

ENSNFUG00015022759

ENSNFUG00015006322

ENSNFUG00015008655

ENSNFUG00015009448

ENSNFUG00015002603

ENSNFUG00015007755

ENSNFUG00015004974

ENSNFUG00015008015

ENSNFUG00015006867

ENSNFUG00015003723

ENSNFUG00015019537

ENSNFUG00015017001

ENSNFUG00015002715

ENSNFUG00015024014

ENSNFUG00015009731

ENSNFUG00015018960

ENSNFUG00015020695

ENSNFUG00015010424

ENSNFUG00015003607

ENSNFUG00015024482

ENSNFUG00015021678

ENSNFUG00015014049

ENSNFUG00015009802

ENSNFUG00015007606

ENSNFUG00015003870

ENSNFUG00015022858

ENSNFUG00015011347

ENSNFUG00015017681

ENSNFUG00015024506

ENSNFUG00015001496

ENSNFUG00015001503

ENSNFUG00015012931

ENSNFUG00015013610

ENSNFUG00015018243

ENSNFUG00015020601

ENSNFUG00015017623

ENSNFUG00015015117

ENSNFUG00015024760

ENSNFUG00015008429

ENSNFUG00015019309

ENSNFUG00015013054

ENSNFUG00015016124

ENSNFUG00015018171

ENSNFUG00015002862

ENSNFUG00015008657

ENSNFUG00015001340

ENSNFUG00015007224

ENSNFUG00015020115

ENSNFUG00015022784

ENSNFUG00015000553

ENSNFUG00015007184

ENSNFUG00015009087

ENSNFUG00015003351

ENSNFUG00015024126

ENSNFUG00015007174

ENSNFUG00015021039

ENSNFUG00015018774

ENSNFUG00015022162

ENSNFUG00015021268

ENSNFUG00015012600

ENSNFUG00015022548

ENSNFUG00015023943

ENSNFUG00015000591

ENSNFUG00015002138

ENSNFUG00015003917

ENSNFUG00015000476

ENSNFUG00015003140

ENSNFUG00015008440

ENSNFUG00015018700

ENSNFUG00015005718

ENSNFUG00015007736

ENSNFUG00015023746

ENSNFUG00015007598

ENSNFUG00015001718

ENSNFUG00015016626

ENSNFUG00015006385

ENSNFUG00015014155

ENSNFUG00015021622

ENSNFUG00015010015

ENSNFUG00015017938

ENSNFUG00015017520

ENSNFUG00015012669

ENSNFUG00015005268

ENSNFUG00015010155

ENSNFUG00015024469

ENSNFUG00015019852

ENSNFUG00015012582

ENSNFUG00015003901

ENSNFUG00015022487

ENSNFUG00015004919

ENSNFUG00015010290

ENSNFUG00015017198

ENSNFUG00015015699

ENSNFUG00015017012

ENSNFUG00015022246

ENSNFUG00015022208

ENSNFUG00015024322

ENSNFUG00015002469

ENSNFUG00015012566

ENSNFUG00015023026

ENSNFUG00015010600

ENSNFUG00015017019

ENSNFUG00015022293

ENSNFUG00015002592

ENSNFUG00015005672

ENSNFUG00015010888

ENSNFUG00015014179

ENSNFUG00015000477

ENSNFUG00015023365

ENSNFUG00015002002

ENSNFUG00015012267

ENSNFUG00015003372

ENSNFUG00015012496

ENSNFUG00015012588

ENSNFUG00015001575

ENSNFUG00015018903

ENSNFUG00015001130

ENSNFUG00015012215

ENSNFUG00015019268

ENSNFUG00015005758

ENSNFUG00015016922

ENSNFUG00015004344

ENSNFUG00015012611

ENSNFUG00015008908

ENSNFUG00015004714

ENSNFUG00015019792

ENSNFUG00015024316

ENSNFUG00015011825

ENSNFUG00015009097

ENSNFUG00015003845

ENSNFUG00015007566

ENSNFUG00015020938

ENSNFUG00015008615

ENSNFUG00015001071

ENSNFUG00015019830

ENSNFUG00015015249

ENSNFUG00015003267

ENSNFUG00015005939

ENSNFUG00015023018

ENSNFUG00015011022

ENSNFUG00015003379

ENSNFUG00015013998

ENSNFUG00015007457

ENSNFUG00015013485

ENSNFUG00015017257

ENSNFUG00015010230

ENSNFUG00015015926

ENSNFUG00015025264

ENSNFUG00015010284

ENSNFUG00015004574

ENSNFUG00015007237

ENSNFUG00015009598

ENSNFUG00015012644

ENSNFUG00015021272

ENSNFUG00015003461

ENSNFUG00015000043

ENSNFUG00015009557

ENSNFUG00015018125

ENSNFUG00015013454

ENSNFUG00015010961

ENSNFUG00015003306

ENSNFUG00015018355

ENSNFUG00015010644

ENSNFUG00015004973

ENSNFUG00015018689

ENSNFUG00015000143

ENSNFUG00015017084

ENSNFUG00015003384

ENSNFUG00015013643

ENSNFUG00015021065

ENSNFUG00015016301

ENSNFUG00015022721

ENSNFUG00015012094

ENSNFUG00015019950

ENSNFUG00015011056

ENSNFUG00015013926

ENSNFUG00015020740

ENSNFUG00015023269

ENSNFUG00015020451

ENSNFUG00015004958

ENSNFUG00015000269

ENSNFUG00015011622

ENSNFUG00015023325

ENSNFUG00015013605

ENSNFUG00015015485

ENSNFUG00015017565

ENSNFUG00015003847

ENSNFUG00015006015

ENSNFUG00015024297

ENSNFUG00015018751

ENSNFUG00015018055

ENSNFUG00015018952

ENSNFUG00015009050

ENSNFUG00015007277

ENSNFUG00015009233

ENSNFUG00015005936

ENSNFUG00015001594

ENSNFUG00015015740

ENSNFUG00015022701

ENSNFUG00015019915

ENSNFUG00015017241

ENSNFUG00015007783

ENSNFUG00015010007

ENSNFUG00015005532

ENSNFUG00015013589

ENSNFUG00015019996

ENSNFUG00015020168

ENSNFUG00015009099

ENSNFUG00015011852

ENSNFUG00015009511

ENSNFUG00015009085

ENSNFUG00015016273

ENSNFUG00015014223

ENSNFUG00015007037

ENSNFUG00015022908

ENSNFUG00015012597

ENSNFUG00015010498

ENSNFUG00015015642

ENSNFUG00015018946

ENSNFUG00015016756

ENSNFUG00015018908

ENSNFUG00015020617

ENSNFUG00015014679

ENSNFUG00015018409

ENSNFUG00015014396

ENSNFUG00015025172

ENSNFUG00015008971

ENSNFUG00015001558

ENSNFUG00015001480

ENSNFUG00015011184

ENSNFUG00015011034

ENSNFUG00015003382

ENSNFUG00015024877

ENSNFUG00015000240

ENSNFUG00015003177

ENSNFUG00015020583

ENSNFUG00015023747

ENSNFUG00015016849

ENSNFUG00015013259

ENSNFUG00015005736

ENSNFUG00015004106

ENSNFUG00015005248

ENSNFUG00015014798

ENSNFUG00015013245

ENSNFUG00015002984

ENSNFUG00015012949

ENSNFUG00015012559

ENSNFUG00015001391

ENSNFUG00015016260

ENSNFUG00015023009

ENSNFUG00015021994

ENSNFUG00015004407

ENSNFUG00015008299

ENSNFUG00015004258

ENSNFUG00015017627

ENSNFUG00015018748

ENSNFUG00015004277

ENSNFUG00015023976

ENSNFUG00015000234

ENSNFUG00015023275

ENSNFUG00015012757

ENSNFUG00015005433

ENSNFUG00015007157

ENSNFUG00015016401

ENSNFUG00015011644

ENSNFUG00015025036

ENSNFUG00015022793

ENSNFUG00015015870

ENSNFUG00015014856

ENSNFUG00015012298

ENSNFUG00015010583

ENSNFUG00015001721

ENSNFUG00015018292

ENSNFUG00015013325

ENSNFUG00015024882

ENSNFUG00015020063

ENSNFUG00015001487

ENSNFUG00015020824

ENSNFUG00015022354

ENSNFUG00015004935

ENSNFUG00015020771

ENSNFUG00015002376

ENSNFUG00015012103

ENSNFUG00015009842

ENSNFUG00015011727

ENSNFUG00015022029

ENSNFUG00015009007

ENSNFUG00015005016

ENSNFUG00015022086

ENSNFUG00015006281

ENSNFUG00015001604

ENSNFUG00015023344

ENSNFUG00015008636

ENSNFUG00015020679

ENSNFUG00015022868

ENSNFUG00015010982

ENSNFUG00015023887

ENSNFUG00015021231

ENSNFUG00015008081

ENSNFUG00015010677

ENSNFUG00015017561

ENSNFUG00015004493

ENSNFUG00015015179

ENSNFUG00015018285

ENSNFUG00015004413

ENSNFUG00015017907

ENSNFUG00015018041

ENSNFUG00015021000

ENSNFUG00015022838

ENSNFUG00015020698

ENSNFUG00015009781

ENSNFUG00015005351

**(G)** **graph.txt**

**(G-1) male >** **06_count > graph.txt**

ENSNFUG00015017902

ENSNFUG00015003177

ENSNFUG00015002592

ENSNFUG00015014290

ENSNFUG00015010294

ENSNFUG00015019695

ENSNFUG00015010245

ENSNFUG00015024488

ENSNFUG00015015851

ENSNFUG00015017155

ENSNFUG00015021982

**(G-2) female >** **06_count > graph.txt**

ENSNFUG00015017902

ENSNFUG00015003177

ENSNFUG00015002592

ENSNFUG00015014290

ENSNFUG00015010294

ENSNFUG00015019695

ENSNFUG00015010245

ENSNFUG00015022764

ENSNFUG00015021077

ENSNFUG00015017155

ENSNFUG00015019719

**(H) R scripts**

**(H-1) logPlot.R**

See Supplementary Figure 8(H-1) logPlot.R (pages 18-19)

**(H-2) counts_to_tpm2.R**

See Supplementary Figure 8(H-1) counts_to_tpm2.R (page 19)

**(H-3) TH.R**

See Supplementary Figure 8(H-1) counts_to_tpm2.R (pages 19-20)

**(H-4) Fig7A.R**

#Heatmap

#Correlation coefficients between samples in the gene sets with TPM > 10 in at least one sample.

library(lattice)

library(latticeExtra)

library(gplots)

library(matrixStats)

library(genefilter)

library(dplyr)

my.col1 <- colorRampPalette(c("blue","white","magenta"))

comm <- commandArgs(trailingOnly = T)

head (comm)

infile1 <- comm[1]

outdir <- comm[2]

indata <- read.delim(infile1, header=T, sep="\t")

x<-indata[,3:46]

x<-x+1

x <- as.matrix(x)

log_x<-log(x,base=2)

cor<-cor(log_x)

cor2<-round(cor, digits = 2)

correlationPlot <- function(x) {

  cor<-cor(log_x);

  fig<-levelplot(cor, col.regions=colorRampPalette(c("yellow", "red"), space = "rgb")(120), scales=list(x=list(rot=55)), main="TPMlibirary_Correlation plot")

  fig

}

outfile <- paste(outdir, "Fig7A.pdf", sep="/")

pdf(file=outfile,onefile=FALSE,paper="special",height=15,width=15,family="Helvetica",pointsize=15)

correlationPlot(log_x)

dev.off()

**(H-5) DESeq2.R**

#PCA(VST)&DESeq2

options(warn=1)

options(scipen=100)

options( java.parameters = "-Xmx64g" )

library( DESeq2 )

packageVersion("DESeq2")

library("dplyr")

library(xlsx)

library("genefilter")

library("gplots")

my.col1 <- colorRampPalette(c("blue","white","magenta"))

args <- commandArgs(T)

workDir   <- args[1]

data      <- args[2]

head(data)

name    <- args[3]

gtfFile   <- args[4]

gene_type <- args[5]

subName   <- ifelse(gene_type=="default","",paste0(".",gene_type))

markers   <- c()

TPMdata <- args[6]

#nzm<-args[7]

newTPMdata<-args[7]

THtpm <- args[8]

infile3 <- args[9];#zebraID�ϊ��\

outdir <- workDir

outDir    <- file.path(workDir)

outDir

outFile   <- file.path(outDir, paste0("TPM",".xlsx"))

outPCA    <- file.path(outDir, paste0("PCA",subName,".xlsx"))

outBAR    <- file.path(outDir, paste0("barplot",subName,".pdf"))

saveData  <- file.path(outDir, paste0("DESeq2_HISAT2",subName,".Rdata"))

name1 <- read.delim(name,header=F, sep=",")

name1<-as.vector(name1)

Group <- data.frame(con = factor(name1))

data<-read.table(data,header=T,row.names=1,sep="\t")

head(data)

#data TPM>10

TPMdata<-read.delim(TPMdata,header=T,sep="\t")

TPMdata1<-as.matrix(TPMdata)

TPMmax10<-TPMdata1[,1]

print(head(TPMdata1))

print(head(TPMmax10))

L1 <- length(TPMmax10)

L1

#TPM

t5 <- c()

t4 <- subset (data, row.names(data) == TPMmax10[1])

t4 <- t4

for (i in 2:L1) {

  t5 <- subset (data, row.names(data) ==  TPMmax10[i], )

  t4 <- rbind (t4, t5)

}

head(t4)

data<-t4

data1<-row.names(data)

data2<-data[6:ncol(data)]

data3<-cbind(data1, data2)

#DESeq2

dds <- DESeqDataSetFromMatrix(countData=data2, colData=Group, design=~ con)

genelength <- matrix( unlist(data [5]), ncol = 1)

mcols(dds)$basepairs <- as.numeric(genelength)

dds <- estimateSizeFactors(dds)

dds <- estimateDispersions(dds)

dds <- nbinomWaldTest(dds)

#vst_value_PCA_plot(Fig.7B)________________________

vst <- vst(dds, blind=FALSE)

head(assay(vst), 3)

vst1<-assay(vst)

colnames(vst1) <- c("G9d_1", "G9d_2", "G9d_3", "G9d_4", "G13d_1", "G13d_2", "G13d_3", "G13d_4","G17d_1", "G17d_2", "G17d_3", "G17d_4", "G21d_1", "G21d_2", "G21d_3", "G21d_4", "G28d_1", "G28d_2", "G28d_3", "G28d_4", "G35d_1", "G35d_2", "G35d_3", "G35d_4", "S9d_1", "S9d_2", "S9d_3", "S9d_4",  "S10d_1", "S10d_2", "S10d_3", "S10d_4", "S11d_1", "S11d_2", "S11d_3", "S11d_4", "S17d_1", "S17d_2", "S17d_3", "S17d_4",  "S21d_1", "S21d_2", "S21d_3", "S21d_4")

outfile <- paste(outdir, "VST_value.txt", sep="/")

write.table(vst1, file=outfile, sep="\t")

res <- prcomp(t(assay(vst)), scale=T)

v <- res$sdev^2

v <- v / sum(v)

v[1]<-round(v[1]*100,1)

v[2]<-round(v[2]*100,1)

v[3]<-round(v[3]*100,1)

v[4]<-round(v[4]*100,1)

v[5]<-round(v[5]*100,1)

v[6]<-round(v[6]*100,1)

v[7]<-round(v[7]*100,1)

v[8]<-round(v[8]*100,1)

v[9]<-round(v[9]*100,1)

v[1]

v[2]

v[3]

v[4]

v[5]

PC1 <- res$x[, 1]

PC2 <- res$x[, 2]

PC3 <- res$x[, 3]

PC4 <- res$x[, 4]

PC5 <- res$x[, 5]

PC6 <- res$x[, 6]

PC7 <- res$x[, 7]

PC8 <- res$x[, 8]

PC9 <- res$x[, 9]

PC1_9<- res$x[,1:9]

outfile <- paste(outdir, "Score_VST_PCA_prcomp.txt", sep="/")

write.table(PC1_9, file=outfile, sep="\t")

col <- c("skyblue3","skyblue3","skyblue3","skyblue3","skyblue3","skyblue3","skyblue3","skyblue3","skyblue3","skyblue3","skyblue3","skyblue3","skyblue3","skyblue3","skyblue3","skyblue3","skyblue3","skyblue3","skyblue3","skyblue3","skyblue3","skyblue3","skyblue3","skyblue3","palevioletred1","palevioletred1","palevioletred1","palevioletred1","palevioletred1","palevioletred1","palevioletred1","palevioletred1","palevioletred1","palevioletred1","palevioletred1","palevioletred1","palevioletred1","palevioletred1","palevioletred1","palevioletred1","palevioletred1","palevioletred1","palevioletred1","palevioletred1","palevioletred1","palevioletred1","palevioletred1","palevioletred1")

pch <- c(0, 0, 0, 0, 0, 0, 0, 0, 0, 0, 0, 0,0, 0, 0, 0, 0, 0, 0, 0, 0, 0, 0, 0, 1, 1, 1, 1, 1, 1, 1, 1, 1, 1, 1, 1, 1, 1, 1, 1, 1, 1, 1, 1, 1, 1, 1, 1)

label3 <- c("1", "1", "1", "1", "2", "2", "2", "2","3", "3", "3", "3", "4", "4", "4", "4", "5", "5", "5", "5", "6", "6", "6", "6", "2", "2", "2", "2", "3", "3", "3", "3", "4", "4", "4", "4",  "5", "5", "5", "5",  "6", "6", "6", "6")

outfile <- paste(outdir, "VST_PCA_prcomp_PC1-2_#2.pdf", sep="/");

pdf(file=outfile,onefile=FALSE,paper="special",height=15,width=15,family="Times",pointsize=20)

xname <- paste("PC1_", v[1], "%", sep="");

yname <- paste("PC2_", v[2], "%", sep="");

plot(PC1, PC2, pch=1, cex=6, col = col,xlab=xname, ylab=yname, main="TPMlibirary_PCA_prcomp_PC1-2")

text(PC1, PC2, label3, col = col, cex=2.5)

dev.off();

#loading factors

fc.l2<-sweep(res$rotation, MARGIN=2, res$sdev, FUN="*")

newTPMdata<-read.delim(newTPMdata,header=T,sep="\t")

colname <- newTPMdata[,1];

colname <- as.matrix(colname)

nfu_G <- data3[,1]

head(nfu_G)

nfu_zeb<-read.delim(infile3, sep="\t",header=F);

nfu_zeb <- as.matrix(nfu_zeb)

head(nfu_zeb)

L1 <- length(nfu_G)

L2 <- L1+1

L2

t5 <- c()

t4 <- subset (nfu_zeb, nfu_zeb[,1] == nfu_G[1], 4)

t4 <- t4[1]

for (i in 2:L1) {

  t5 <- subset (nfu_zeb, nfu_zeb[,1] ==  nfu_G[i], 4)

  t6 <- t5[1]

  t4 <- c (t4, t6)

}

zebraID <- t4

zebraID <- as.matrix(zebraID)

colnames(zebraID)<-c("zebra_geneID")

length(zebraID)

head(zebraID)

t5 <- c()

t4 <- subset (nfu_zeb, nfu_zeb[,1] == nfu_G[1], 5)

t4 <- t4[1]

for (i in 2:L1) {

  t5 <- subset (nfu_zeb, nfu_zeb[,1] ==  nfu_G[i], 5)

  t6 <- t5[1]

  t4 <- c (t4, t6)

}

zebraGName <- t4

zebraGName <- as.matrix(zebraGName)

colnames(zebraGName)<-c("zebra_geneName")

t5 <- c()

t4 <- subset (nfu_zeb, nfu_zeb[,1] == nfu_G[1], 6)

t4 <- t4[1]

for (i in 2:L1) {

  t5 <- subset (nfu_zeb, nfu_zeb[,1] ==  nfu_G[i], 6)

  t6 <- t5[1]

  t4 <- c (t4, t6)

}

zebraGene <- t4

zebraGene <- as.matrix(zebraGene)

colnames(zebraGene)<-c("zebra_gene")

fc.l<-cbind(data3,zebraID,zebraGName,zebraGene,fc.l2)

outfile <- paste(outdir, "FactorLoadings_VST_PCA_prcomp.txt", sep="/")

write.table(fc.l, file=outfile, sep="\t")

#______________________

#DEGs(For Supplementary Fig.5-6)

norm.dt   <- data.frame(counts(dds, normalized=T), check.names=F)

fpkm.dt   <- data.frame(fpkm(dds, robust=F), check.names=F)

tpm.dt    <- data.frame(sweep(fpkm.dt*1e6, 2, colSums(fpkm.dt),"/"), check.names=F)

cpm.dt    <- data.frame(fpm(dds, robust=F), check.names=F)

outfile <- paste(outDir, "DESeq2_results.Rdata", sep="/")

#TPM

wb <- createWorkbook(type="xlsx")

sheet  <- createSheet(wb, sheet="TPM")

row.names(tpm.dt)<-row.names(data)

colnames(tpm.dt)<-name1

addDataFrame(tpm.dt, sheet, row.names=T)

saveWorkbook(wb, file=outFile)

outfile <- paste(outDir, "TPM.txt", sep="/")

write.table(tpm.dt, file=outfile, row.names=T)

dds <- DESeqDataSetFromMatrix(countData=data2, colData=Group, design=~ con)

head(dds)

genelength <- matrix( unlist(data [5]), ncol = 1)

mcols(dds)$basepairs <- as.numeric(genelength)

dds <- DESeq(dds)

head(dds)

res<-results(dds)

head(res)

terms<-c("S21d","S17d","S11d","S10d","S9d","G35d","G28d","G21d","G17d","G13d","G9d")

terms2<-combn(terms,2)

terms2

n<-ncol(terms2)

newTPMdata<-as.matrix(newTPMdata)

L1 <- length(TPMmax10)

L1

#TPM

t5 <- c()

t4 <- subset (newTPMdata, newTPMdata[,1] == TPMmax10[1])

t4 <- t4

for (i in 2:L1) {

  t5 <- subset (newTPMdata, newTPMdata[,1] ==  TPMmax10[i], )

  t4 <- rbind (t4, t5)

}

head(t4)

newTPMdata<-t4

head(newTPMdata)

FC<-log2(3)

print(FC)

for (j in 1:n) {

  terms3<-terms2[,j]

  terms4<-append("con",terms3)

  print(terms4)

  res<-results(dds, contrast=terms4)

  res$TPM <-newTPMdata

  tpm<-THtpm

  print(tpm)

  data2 <- as.data.frame(res)

  outfilename <- paste0("DESeq2_result_", terms3[1],"vs",terms3[2],"_TPMmax10_All.txt")

  out_f <- paste(outDir, outfilename, sep="/");

  write.table(data2, out_f, sep="\t", append=F, quote=F, row.names=T, col.names=T)

  data2upAll <- subset(data2, (data2$pad<=0.01 & (data2$log2FoldChange>=FC )))

  outfilename <- paste0("DESeq2_result_", terms3[1],"vs",terms3[2],"_TPMmax10_3.0upAll.txt")

  out_f <- paste(outDir, outfilename, sep="/");

  write.table(data2upAll, out_f, sep="\t", append=F, quote=F, row.names=T, col.names=T)

  data2downAll <- subset(data2, (data2$pad<=0.01 & (data2$log2FoldChange<=-FC )))

  outfilename <- paste0("DESeq2_result_", terms3[1],"vs",terms3[2],"_TPMmax10_3.0downAll.txt")

  out_f <- paste(outDir, outfilename, sep="/");

  write.table(data2downAll, out_f, sep="\t", append=F, quote=F, row.names=T, col.names=T)

}

**(H-6) ID-TPMv1.R**

#ID->TPM

comm <- commandArgs(trailingOnly = T)

infile1 <- comm[1]

infile2 <- comm[2]

infile3 <- comm[3]

outdir <- comm[4]

TPM <- read.delim(infile3, sep="\t");

TPM <- as.matrix(TPM)

downcomID <- read.delim(infile1,header=F,sep=",")

downcomID <-as.matrix(downcomID)

downcomID

L1 <- length(downcomID)

L1

#TPM

t5 <- c()

t4 <- subset (TPM, TPM[,1] == downcomID[1])

t4 <- t4

for (i in 2:L1) {

   t5 <- subset (TPM, TPM[,1] ==  downcomID[i], )

   t4 <- rbind (t4, t5)

}

head(t4)

outfile <- paste(outdir, infile2, sep="/")

write.table(t4, file=outfile, sep="\t")

**(H-7) Fig7C.R**

library(lattice)

library(latticeExtra)

library(gplots)

library(matrixStats)

library(genefilter)

library(dplyr)

my.col1 <- colorRampPalette(c("blue","white","magenta"))

comm <- commandArgs(trailingOnly = T);

head (comm)

infile1 <- comm[1];

outdir <- comm[2];

data <- read.delim(infile1, header=T, sep="\t");

x<-data[,3:46]

x<-x+1

x <- as.matrix(x)

m1<-rowMeans(x[,1:4])

m2<-rowMeans(x[,5:8])

m3<-rowMeans(x[,9:12])

m4<-rowMeans(x[,13:16])

m5<-rowMeans(x[,17:21])

m6<-rowMeans(x[,22:24])

m7<-rowMeans(x[,25:28])

m8<-rowMeans(x[,29:32])

m9<-rowMeans(x[,33:36])

m10<-rowMeans(x[,37:40])

m11<-rowMeans(x[,41:44])

mx<-cbind(m1,m7,m2,m8,m3,m9,m4,m10,m5,m11,m6)

mx_a<-mx/rowMeans(mx)

log_mx_a<-log(mx_a, base=2)

outfile <- paste(outdir, "Fig7C_heatmap.pdf", sep="/");

pdf(file=outfile,onefile=FALSE,paper="special",height=15,width=15,family="Helvetica",pointsize=20)

heatmap.2(as.matrix(log_mx_a),col=my.col1(269),Colv=NA, scale="none",key=TRUE, symm=F,symkey=F,symbreaks=T, breaks=c(seq(-2,2,length=270)), trace="none",distfun = function(x) {dist(x, method="euclidean")},hclustfun = function(x) {hclust(x, method="ward.D2")})

dev.off();

outfile <- paste(outdir, "Fig7C_clustering.pdf", sep="/");

pdf(file=outfile,onefile=FALSE,paper="special",height=15,width=15,family="Helvetica",pointsize=20)

heatmap.2(as.matrix(log_mx_a),col=my.col1(269),scale="none",key=TRUE, symm=F,symkey=F,symbreaks=T, breaks=c(seq(-2,2,length=270)), trace="none",distfun = function(x) {dist(x, method="euclidean")},hclustfun = function(x) {hclust(x, method="ward.D2")})

dev.off();

**(H-8) beeswarm2.R**

args <- commandArgs(T)

TPMdata <- args[1]

outDir <- args[2]

name <- args[3]

TPMdata <- read.delim(TPMdata, header=T, sep="\t", row.names=1)

TPMdata <- as.matrix(TPMdata)

N<-matrix(NA,nrow(TPMdata),4)

head(N)

print(nrow(TPMdata))

print(nrow(N))

head(TPMdata[,3:26])

head(TPMdata[,27:46])

dx <- cbind(TPMdata[,3:26],N,TPMdata[,27:46])

head(dx)

label<-c(1,1,1,1,1,1,1,1,2,2,2,2,2,2,2,2,3,3,3,3,3,3,3,3,4,4,4,4,4,4,4,4,5,5,5,5,5,5,5,5,6,6,6,6,6,6,6,6)

label2<-c(1,1,1,1,2,2,2,2,2,2,2,2,3,3,3,3,3,3,3,3,4,4,4,4,4,4,4,4,5,5,5,5,6,6,6,6,6,6,6,6,7,7,7,7,7,7,7,7)

X = ceiling(nrow(dx)/30)

X

print(X)

i <- 1

for(i in 1:X){

  print(i)

  filename <- paste0(name,"bw",i,".pdf")

  outfile <- paste(outDir, filename, sep="/");

  pdf(file=outfile,onefile=FALSE,paper="special",height=15,width=15,family="Helvetica",pointsize=8)

  H <- ceiling(nrow(dx)/2)

  H

  par(mfrow=c(5,6))

  a<-30*(i-1)+1

  if(i==X){

    b<-nrow(dx)

  }else{

    b<-30*i

  }

  for(j in a:b){

    y1<-c(as.numeric(dx[j,1:4]), as.numeric(dx[j,25:28]))

    y2<-c(as.numeric(dx[j,5:8]), as.numeric(dx[j,29:32]))

    y3<-c(as.numeric(dx[j,9:12]),as.numeric(dx[j,33:36]))

    y4<-c(as.numeric(dx[j,13:16]),as.numeric(dx[j,37:40]))

    y5<-c(as.numeric(dx[j,17:20]),as.numeric(dx[j,41:44]))

    y6<-c(as.numeric(dx[j,21:24]),as.numeric(dx[j,45:48]))

    col<-c("#00A0E9","#00A0E9","#00A0E9","#00A0E9","#FFB2A4", "#FFB2A4", "#FFB2A4", "#FFB2A4")

    v1<-cbind.data.frame(y1,col)

    colnames(v1)<-c("TPM","col")

    v2<-cbind.data.frame(y2,col)

    colnames(v2)<-c("TPM","col")

    v3<-cbind.data.frame(y3,col)

    colnames(v3)<-c("TPM","col")

    v4<-cbind.data.frame(y4,col)

    colnames(v4)<-c("TPM","col")

    v5<-cbind.data.frame(y5,col)

    colnames(v5)<-c("TPM","col")

    v6<-cbind.data.frame(y6,col)

    colnames(v6)<-c("TPM","col")

    y<-rbind.data.frame(v1,v2,v3,v4,v5,v6)

    dx2<-cbind.data.frame(label,y)

    title=TPMdata[j,55:56]

    max=max(as.numeric(dx[j,c(1:24,29:48)]))+10

    print(max)

    if(!is.na(max)){

      beeswarm(TPM~label, data=dx2,pch=16,cex=1.5, yaxs="i", las = 1, xlab = "", cex.lab  = 1.4, cex.axis=1.5, cex.names=1.5, las = 2, main=title,pwcol=c("#00A0E9","#00A0E9","#00A0E9", "#00A0E9","#FFB2A4", "#FFB2A4", "#FFB2A4","#FFB2A4","#00A0E9","#00A0E9","#00A0E9", "#00A0E9","#FFB2A4", "#FFB2A4", "#FFB2A4","#FFB2A4","#00A0E9","#00A0E9","#00A0E9", "#00A0E9","#FFB2A4", "#FFB2A4", "#FFB2A4","#FFB2A4","#00A0E9","#00A0E9","#00A0E9", "#00A0E9","#FFB2A4", "#FFB2A4", "#FFB2A4","#FFB2A4","#00A0E9","#00A0E9","#00A0E9", "#00A0E9","#FFB2A4", "#FFB2A4", "#FFB2A4","#FFB2A4","#00A0E9","#00A0E9","#00A0E9", "#00A0E9","#FFB2A4", "#FFB2A4", "#FFB2A4","#FFB2A4"),labels = c("G9", "G13S9", "G17S10", "G21S11","G28S17","G35S21"),ylim=c(0,max))

      t1<-as.numeric(dx[j,1:4])

      t2<-as.numeric(dx[j,25:28])

      t3<-as.numeric(dx[j,5:8])

      t4<-as.numeric(dx[j,29:32])

      t5<-as.numeric(dx[j,9:12])

      t6<-as.numeric(dx[j,33:36])

      t7<-as.numeric(dx[j,13:16])

      t8<-as.numeric(dx[j,37:40])

      t9<-as.numeric(dx[j,17:20])

      t10<-as.numeric(dx[j,41:44])

      t11<-as.numeric(dx[j,21:24])

      t12<-as.numeric(dx[j,45:48])

      dx3<-cbind(t1,t2,t3,t4,t5,t6,t7,t8,t9,t10,t11,t12)

      m <- apply(dx3, 2, mean, na.rm = TRUE)

      s <- apply(dx3, 2, sd, na.rm = TRUE)

      b <- c(1,1,2,2,3,3,4,4,5,5,6,6)

      arrows (b, m,

              b, m + s,

              length = 0.08,

              angle = 90,

              col=c("#00A0E9","#FFB2A4","#00A0E9","#FFB2A4", "#00A0E9","#FFB2A4",  "#00A0E9", "#FFB2A4"))

      arrows (b, m,

              b, m - s,

              length = 0.08,

              angle = 90,

              col=c("#00A0E9","#FFB2A4","#00A0E9","#FFB2A4", "#00A0E9","#FFB2A4",  "#00A0E9", "#FFB2A4"))

      arrows (b, m,

              b+0.2, m,

              length = 0,

              angle = 90,

              col=c("#00A0E9","#FFB2A4","#00A0E9","#FFB2A4", "#00A0E9","#FFB2A4",  "#00A0E9", "#FFB2A4"))

      arrows (b, m,

              b-0.2, m,

              length = 0,

              angle = 90,

              col=c("#00A0E9","#FFB2A4","#00A0E9","#FFB2A4", "#00A0E9","#FFB2A4",  "#00A0E9", "#FFB2A4"))

    }else{

    }

  }

  dev.off();

}

**(H-9) DESeq2v4-2.R**

#DEGs TPM50, FC4 (FDR0.01)

options(warn=1)

options(scipen=100)

options( java.parameters = "-Xmx64g" )

library( DESeq2 )

packageVersion("DESeq2")

library("dplyr")

library(xlsx)

library("genefilter")

library("gplots")

my.col1 <- colorRampPalette(c("blue","white","magenta"))

args <- commandArgs(T)

workDir   <- args[1]

data      <- args[2]

head(data)

name    <- args[3]

gtfFile   <- args[4]

gene_type <- args[5]

subName   <- ifelse(gene_type=="default","",paste0(".",gene_type))

markers   <- c()

TPMdata <- args[6]

#nzm<-args[7]

newTPMdata<-args[7]

THtpm <- args[8]

outDir    <- file.path(workDir)

outDir

outFile   <- file.path(outDir, paste0("TPM",".xlsx"))

outPCA    <- file.path(outDir, paste0("PCA",subName,".xlsx"))

outBAR    <- file.path(outDir, paste0("barplot",subName,".pdf"))

saveData  <- file.path(outDir, paste0("DESeq2_HISAT2",subName,".Rdata"))

name1 <- read.delim(name,header=F, sep=",")

name1<-as.vector(name1)

Group <- data.frame(con = factor(name1))

data<-read.table(data,header=T,row.names=1,sep="\t")

TPMdata<-read.delim(TPMdata,header=T,sep="\t")

TPMdata1<-as.matrix(TPMdata)

TPMmax50<-TPMdata1[,1]

print(head(TPMdata1))

print(head(TPMmax50))

L1 <- length(TPMmax50)

L1

#TPM

t5 <- c()

t4 <- subset (data, row.names(data) == TPMmax50[1])

t4 <- t4

for (i in 2:L1) {

  t5 <- subset (data, row.names(data) ==  TPMmax50[i], )

  t4 <- rbind (t4, t5)

}

head(t4)

data<-t4

data1<-row.names(data)

data2<-data[6:ncol(data)]

data3<-cbind(data1, data2)

#DEseq2

dds <- DESeqDataSetFromMatrix(countData=data2, colData=Group, design=~ con)

genelength <- matrix( unlist(data [5]), ncol = 1)

mcols(dds)$basepairs <- as.numeric(genelength)

dds <- estimateSizeFactors(dds)

dds <- estimateDispersions(dds)

dds <- nbinomWaldTest(dds)

norm.dt   <- data.frame(counts(dds, normalized=T), check.names=F)

fpkm.dt   <- data.frame(fpkm(dds, robust=F), check.names=F)

tpm.dt    <- data.frame(sweep(fpkm.dt*1e6, 2, colSums(fpkm.dt),"/"), check.names=F)

cpm.dt    <- data.frame(fpm(dds, robust=F), check.names=F)

outfile <- paste(outDir, "DESeq2_results_FC2_0.01.Rdata", sep="/")

#TPM

wb <- createWorkbook(type="xlsx")

sheet  <- createSheet(wb, sheet="TPM")

row.names(tpm.dt)<-row.names(data)

colnames(tpm.dt)<-name1

addDataFrame(tpm.dt, sheet, row.names=T)

saveWorkbook(wb, file=outFile)

outfile <- paste(outDir, "TPM_FC2_0.01.txt", sep="/")

write.table(tpm.dt, file=outfile, row.names=T)

dds <- DESeqDataSetFromMatrix(countData=data2, colData=Group, design=~ con)

head(dds)

genelength <- matrix( unlist(data [5]), ncol = 1)

mcols(dds)$basepairs <- as.numeric(genelength)

dds <- DESeq(dds)

res<-results(dds)

terms<-c("S21d","S17d","S11d","S10d","S9d","G35d","G28d","G21d","G17d","G13d","G9d")

terms2<-combn(terms,2)

terms2

n<-ncol(terms2)

newTPMdata<-read.delim(newTPMdata,header=T,sep="\t")

newTPMdata<-as.matrix(newTPMdata)

L1 <- length(TPMmax50)

L1

#TPM

t5 <- c()

t4 <- subset (newTPMdata, newTPMdata[,1] == TPMmax50[1])

t4 <- t4

for (i in 2:L1) {

  t5 <- subset (newTPMdata, newTPMdata[,1] ==  TPMmax50[i], )

  t4 <- rbind (t4, t5)

}

newTPMdata<-t4

DEGall_FC4_fdr0.01<-c()

for (j in 1:n) {

  terms3<-terms2[,j]

  terms4<-append("con",terms3)

  print(terms4)

  res<-results(dds, contrast=terms4)

  res$TPM <-newTPMdata

  tpm<-THtpm

  print(tpm)

  data2 <- as.data.frame(res)

  data2upAll <- subset(data2, (data2$pad<=0.01 & (data2$log2FoldChange>=2 )))

  DEG<-rownames(data2upAll)

  DEGall_FC4_fdr0.01<-unique(c(DEG, DEGall_FC4_fdr0.01))

  outfilename <- paste0("DESeq2_result_", terms3[1],"vs",terms3[2],"_TPMmax50_upAll_FC4_0.01.txt")

  out_f <- paste(outDir, outfilename, sep="/");

  write.table(data2upAll, out_f, sep="\t", append=F, quote=F, row.names=T, col.names=T)

  data2downAll <- subset(data2, (data2$pad<=0.01 & (data2$log2FoldChange<=-2 )))

  DEG<-rownames(data2downAll)

  DEGall_FC4_fdr0.01<-unique(c(DEG, DEGall_FC4_fdr0.01))

  outfilename <- paste0("DESeq2_result_", terms3[1],"vs",terms3[2],"_TPMmax50_downAll_FC4_0.01.txt")

  out_f <- paste(outDir, outfilename, sep="/");

  write.table(data2downAll, out_f, sep="\t", append=F, quote=F, row.names=T, col.names=T)

}

#Add TPM data

head(DEGall_FC4_fdr0.01)

data <- args[2]

data<-read.delim(data,header=T,row.names=1,sep="\t")

data1<-row.names(data)

data2<-data[6:ncol(data)]

data3<-cbind(data1, data2)

newTPMdata<-args[7]

newTPMdata<-read.delim(newTPMdata,header=T,row.names=1,sep="\t")

newTPMdata1<-row.names(newTPMdata)

newTPMdata3<-cbind(newTPMdata1, newTPMdata)

L1<-length(DEGall_FC4_fdr0.01)

L1

DEGallTPM1<-matrix()

DEGallTPM2<-matrix()

DEGallcount1<-matrix()

DEGallcount2<-matrix()

DEGallTPM1<-subset(newTPMdata, newTPMdata3[,1]==DEGall_FC4_fdr0.01[1])

DEGallTPM1

DEGallcount1<-subset(data, data3[,1]==DEGall_FC4_fdr0.01[1])

DEGallcount1

for (i in 2:L1){

  DEGallTPM2<-subset(newTPMdata, newTPMdata3[,1]==DEGall_FC4_fdr0.01[i])

  DEGallTPM1<-rbind(DEGallTPM1,DEGallTPM2)

  DEGallcount2<-subset(data, data3[,1]==DEGall_FC4_fdr0.01[i])

  DEGallcount1<-rbind(DEGallcount1,DEGallcount2)

}

outfilename <- paste0("DESeq2_result_DEGall_FC4_fdr0.01","_TPMmax50.txt")

out_f <- paste(outDir, outfilename, sep="/");

write.table(DEGallTPM1, out_f, sep="\t", append=F, quote=F, row.names=T, col.names=T)

outfilename <- paste0("DESeq2_result_DEGall_FC4_fdr0.01","_countRAWmax50.txt")

out_f <- paste(outDir, outfilename, sep="/");

write.table(DEGallcount1, out_f, sep="\t", append=F, quote=F, row.names=T, col.names=T)

**(H-10)** **correlation3.R**

#Fig.7C

options(warn=1)

options(scipen=100)

options( java.parameters = "-Xmx64g" )

library(xlsx)

args <- commandArgs(T)

workDir   <- args[1]

data      <- args[2]

name    <- args[3]

newTPMdata <- args[4]

newTPMdata<-read.delim(newTPMdata,header=T,row.names=1,sep="\t")

outname <- args[5]

outDir    <- file.path(workDir)

name1 <- read.delim(name,header=F, sep=",")

name1<-as.vector(name1)

data<-read.delim(data,header=T,row.names=1,sep="\t")

data1<-row.names(data)

data2<-data[2:45]

data3<-cbind(data1, data2)

data3<-as.data.frame(data3)

colnames(data2)<-name1

data2<-as.data.frame(data2)

terms<-name1

n<-ncol(terms)

n2<-n/4

k<-0

Average<-c()

for (j in 1:n2) {

  print(j)

  m<-1+(4*k)

  i<-m+3

  Mean<-rowMeans(data2[,m:i])

  Mean<-as.matrix(Mean)

  print(head(Mean))

  print(ncol(Mean))

  print(length(data1))

  print(nrow(Mean))

  A<-Mean[,1]

  Average<-cbind(Average,A)

  k<-k+1

}

log<-log(Average+1, base=2)

cor1<-c()

n3<-length(data1)

for (j in 1:n3) {

  cor2<-cor(log[j,1:5],log[j,6:10])

  cor1<-rbind(cor1,cor2)

}

data3$cor<-cor1

Alldata<-cbind(data1,data3$cor,log,data)

outfile <- paste(workDir, outname, sep="/")

write.table(Alldata, file=outfile, sep="\t", append=F, quote=F, row.names=T, col.names=T)

**(H-10)** **Fig7F.R**

#Figure7F, correlation heatmap

library(lattice)

library(latticeExtra)

library(gplots)

library(matrixStats)

library(genefilter)

library(dplyr)

my.col1 <- colorRampPalette(c("blue","white","magenta"))

comm <- commandArgs(trailingOnly = T);

infile1 <- comm[1];

outdir <- comm[2];

data<-read.delim(infile1, header=T, sep="\t")

x<-data[,15:58]

x<-x+1

x <- as.matrix(x)

m1<-rowMeans(x[,1:4])

m2<-rowMeans(x[,5:8])

m3<-rowMeans(x[,9:12])

m4<-rowMeans(x[,13:16])

m5<-rowMeans(x[,17:21])

m6<-rowMeans(x[,22:24])

m7<-rowMeans(x[,25:28])

m8<-rowMeans(x[,29:32])

m9<-rowMeans(x[,33:36])

m10<-rowMeans(x[,37:40])

m11<-rowMeans(x[,41:44])

mx<-cbind(m1,m2,m3,m4,m5,m6,m7,m8,m9,m10,m11)

log_mx<-log(mx, base=2)

cor<-cor(log_mx);

cor2<-round(cor, digits = 2)

correlationPlot <- function(x) {

  cor<-cor(log_mx);

  fig<-levelplot(cor, col.regions=colorRampPalette(c("white","yellow", "red"), space = "rgb")(120), scales=list(x=list(rot=55)), main="Correlation Plot")

  fig+layer(panel.text(x,y,labels=cor2))

}

outfile <- paste(outdir, "Fig7F.pdf", sep="/");

pdf(file=outfile,onefile=FALSE,paper="special",height=15,width=15,family="Helvetica",pointsize=20)

correlationPlot(log_mx);

dev.off();

**(H-11) DESeq2v6.R**

options(warn=1)

options(scipen=100)

options( java.parameters = "-Xmx64g" )

library( DESeq2 )

packageVersion("DESeq2")

#1.28.1

library("dplyr")

library(xlsx)

library("genefilter")

library("gplots")

my.col1 <- colorRampPalette(c("blue","white","magenta"))

args <- commandArgs(T)

workDir   <- args[1]

data      <- args[2]

head(data)

name    <- args[3]

gtfFile   <- args[4]

gene_type <- args[5]

subName   <- ifelse(gene_type=="default","",paste0(".",gene_type))

markers   <- c()

TPMdata <- args[6]

newTPMdata<-args[7]

THtpm <- args[8]

infile3 <- args[9]

outdir <- workDir

outDir    <- file.path(workDir)

outDir

outFile   <- file.path(outDir, paste0("TPM",".xlsx"))

outPCA    <- file.path(outDir, paste0("PCA",subName,".xlsx"))

outBAR    <- file.path(outDir, paste0("barplot",subName,".pdf"))

saveData  <- file.path(outDir, paste0("DESeq2_HISAT2",subName,".Rdata"))

name1 <- read.delim(name,header=F, sep=",")

name1<-as.vector(name1)

Group <- data.frame(con = factor(name1))

data<-read.table(data,header=T,row.names=1,sep="\t")

#data TPM>10

TPMdata<-read.delim(TPMdata,header=T,sep="\t")

TPMdata1<-as.matrix(TPMdata)

TPMmax10<-TPMdata1[,1]

print(head(TPMdata1))

print(head(TPMmax10))

L1 <- length(TPMmax10)

L1

#TPM

t5 <- c()

t4 <- subset (data, row.names(data) == TPMmax10[1])

t4 <- t4

for (i in 2:L1) {

  t5 <- subset (data, row.names(data) ==  TPMmax10[i], )

  t4 <- rbind (t4, t5)

}

data<-t4

data1<-row.names(data)

data2<-data[6:ncol(data)]

data3<-cbind(data1, data2)

#DEseq2

dds <- DESeqDataSetFromMatrix(countData=data2, colData=Group, design=~ con)

genelength <- matrix( unlist(data [5]), ncol = 1)

mcols(dds)$basepairs <- as.numeric(genelength)

dds <- estimateSizeFactors(dds)

dds <- estimateDispersions(dds)

dds <- nbinomWaldTest(dds)

norm.dt   <- data.frame(counts(dds, normalized=T), check.names=F)

fpkm.dt   <- data.frame(fpkm(dds, robust=F), check.names=F)

tpm.dt    <- data.frame(sweep(fpkm.dt*1e6, 2, colSums(fpkm.dt),"/"), check.names=F)

cpm.dt    <- data.frame(fpm(dds, robust=F), check.names=F)

outfile <- paste(outDir, "DESeq2_results.Rdata", sep="/")

#TPM

wb <- createWorkbook(type="xlsx")

sheet  <- createSheet(wb, sheet="TPM")

row.names(tpm.dt)<-row.names(data)

colnames(tpm.dt)<-name1

addDataFrame(tpm.dt, sheet, row.names=T)

saveWorkbook(wb, file=outFile)

outfile <- paste(outDir, "TPM.txt", sep="/")

write.table(tpm.dt, file=outfile, row.names=T)

dds <- DESeqDataSetFromMatrix(countData=data2, colData=Group, design=~ con)

genelength <- matrix( unlist(data [5]), ncol = 1)

mcols(dds)$basepairs <- as.numeric(genelength)

dds <- DESeq(dds)

res<-results(dds)

terms<-c("S21d","S17d","Sj","G35d","G28d","Gj")

terms2<-combn(terms,2)

terms2

n<-ncol(terms2)

print(resultsNames(dds))

#newTPMdata TPM>10

newTPMdata<-read.delim(newTPMdata,header=T,sep="\t")

newTPMdata<-as.matrix(newTPMdata)

L1 <- length(TPMmax10)

L1

#TPM

t5 <- c()

t4 <- subset (newTPMdata, newTPMdata[,1] == TPMmax10[1])

t4 <- t4

for (i in 2:L1) {

  t5 <- subset (newTPMdata, newTPMdata[,1] ==  TPMmax10[i], )

  t4 <- rbind (t4, t5)

}

head(t4)

newTPMdata<-t4

head(newTPMdata)

FC<-log2(1.5)

for (j in 1:n) {

  terms3<-terms2[,j]

  terms4<-append("con",terms3)

  print(terms4)

  res<-results(dds, contrast=terms4)

  res$TPM <-newTPMdata

  tpm<-THtpm

  data2 <- as.data.frame(res)

  data2upAll <- subset(data2, (data2$pad<=0.05 & (data2$log2FoldChange>=FC )))

  print(head(data2upAll))

  outfilename <- paste0("DESeq2_result_", terms3[1],"vs",terms3[2],"_TPMmax10_upAll_1.5.txt")

  out_f <- paste(outDir, outfilename, sep="/");

  write.table(data2upAll, out_f, sep="\t", append=F, quote=F, row.names=T, col.names=T)

  data2downAll <- subset(data2, (data2$pad<=0.05 & (data2$log2FoldChange<=-FC )))

  outfilename <- paste0("DESeq2_result_", terms3[1],"vs",terms3[2],"_TPMmax10_downAll_1.5.txt")

  out_f <- paste(outDir, outfilename, sep="/");

  write.table(data2downAll, out_f, sep="\t", append=F, quote=F, row.names=T, col.names=T)

}
